# Triassic stem caecilian supports dissorophoid origin of living amphibians

**DOI:** 10.1038/s41586-022-05646-5

**Published:** 2023-01-25

**Authors:** Ben T. Kligman, Bryan M. Gee, Adam D. Marsh, Sterling J. Nesbitt, Matthew E. Smith, William G. Parker, Michelle R. Stocker

**Affiliations:** 1 Department of Resource Management and Science, Petrified Forest National Park, Petrified Forest, AZ USA; 2 Department of Geosciences, Virginia Tech, Blacksburg, VA USA; 3 Burke Museum and Department of Biology, University of Washington, Seattle, WA USA

**Keywords:** Palaeontology, Palaeoecology, Herpetology, Phylogenetics

## Abstract

Living amphibians (Lissamphibia) include frogs and salamanders (Batrachia) and the limbless worm-like caecilians (Gymnophiona). The estimated Palaeozoic era gymnophionan–batrachian molecular divergence^1^ suggests a major gap in the record of crown lissamphibians prior to their earliest fossil occurrences in the Triassic period^2–6^. Recent studies find a monophyletic Batrachia within dissorophoid temnospondyls^7–10^, but the absence of pre-Jurassic period caecilian fossils^11,12^ has made their relationships to batrachians and affinities to Palaeozoic tetrapods controversial^1,8,13,14^. Here we report the geologically oldest stem caecilian—a crown lissamphibian from the Late Triassic epoch of Arizona, USA—extending the caecilian record by around 35 million years. These fossils illuminate the tempo and mode of early caecilian morphological and functional evolution, demonstrating a delayed acquisition of musculoskeletal features associated with fossoriality in living caecilians, including the dual jaw closure mechanism^15,16^, reduced orbits^17^ and the tentacular organ^18^. The provenance of these fossils suggests a Pangaean equatorial origin for caecilians, implying that living caecilian biogeography reflects conserved aspects of caecilian function and physiology^19^, in combination with vicariance patterns driven by plate tectonics^20^. These fossils reveal a combination of features that is unique to caecilians alongside features that are shared with batrachian and dissorophoid temnospondyls, providing new and compelling evidence supporting a single origin of living amphibians within dissorophoid temnospondyls.

## Abstract (web summary)

Analysis of fossils of the oldest known caecilian provide insights into the origin and morphological and functional evolution of caecilians.

## Main

Of the nine tetrapod lineages surviving from the Triassic to the present day^21^, caecilians have the most depauperate fossil record, with only 11 total occurrences^22^; of these, only *Rubricacaecilia monbaroni*^23^ and *Eocaecilia micropodia*^11,12^ represent unambiguous stem caecilians. The estimated Permo–Carboniferous origin of caecilians leaves a gap exceeding 70 million years between putative Palaeozoic relatives and *Eocaecilia*^1^. The absence of a pre-Jurassic caecilian record provides little evidence informing the pattern of morphological transformations leading to the specialized caecilian body plan, the timing and pattern of caecilian origins and diversification, the functional and ecological origins of extant caecilians, and caecilian palaeobiogeography. Furthermore, this gap has resulted in longstanding disagreement regarding the relationships of living amphibian groups to each other and to other tetrapods with multiple mutually exclusive hypotheses proposed^8,14^. With the discovery of *Gerobatrachus hottoni*^7^, an early Permian dissorophoid bearing a combination of batrachian and amphibamiform features, the monophyly of Batrachia nested within amphibamiform dissorophoids reached near-consensus opinion^8^, demonstrating the crucial nature of new fossil evidence to questions of lissamphibian origins. Despite the improved understanding of batrachian origins, the origins of Lissamphibia remain contentious, now hinging on the relationships of caecilians to batrachians and Palaeozoic tetrapods^1,8,13,14^. Therefore, consensus on lissamphibian origins can be resolved only with the addition of new caecilian fossils filling the morphological gap between *Eocaecilia* and Palaeozoic tetrapods.

**Extended Data Fig. 1:**
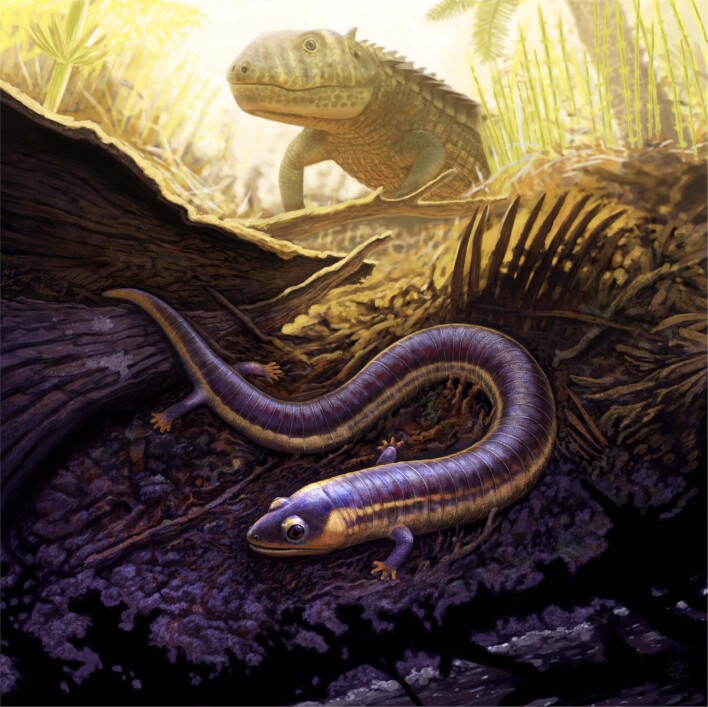
Life reconstruction of *Funcusvermis gilmorei* (lower) and *Acaenasuchus geoffreyi* (upper) in a paleoenvironmental reconstruction of PFV 456. Illustration by Andrey Atuchin.

**Extended Data Fig. 2:**
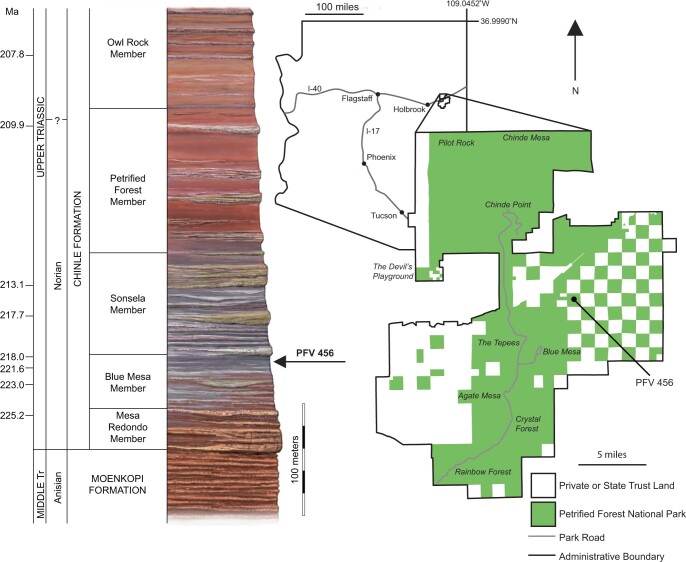
Geographic and stratigraphic context of PFV 456 (Thunderstorm Ridge), the type locality of *Funcusvermis gilmorei* in the upper Blue Mesa Member of the Chinle Formation (Late Triassic) at Petrified Forest National Park, AZ, USA. U-Pb radioisotopic ages derived from refs. ^24,25^.

Here we approach such consensus by reporting the discovery of a new stem caecilian from a multitaxic microvertebrate and macrovertebrate bonebed in the Upper Triassic Chinle Formation of Petrified Forest National Park (PEFO), Arizona, USA (Extended Data Figs. 1 and 2). This material represents the most abundant caecilian-bearing fossil locality known, with at least 76 individuals consisting of isolated three-dimensional skeletal elements that we infer to belong to the same taxon, including elements from the upper and lower jaws, and postcrania (Supplementary Information, section 1).

## Systematic palaeontology

Lissamphibia Haeckel, 1866

Gymnophionomorpha Marjanović and Laurin, 2008

*Funcusvermis gilmorei* gen. et sp. nov.

**Etymology**. *Funcus*, Latinized form of the English word funky (funk is an upbeat, rhythmic form of dance music); *vermis*, worm (Latin); in honour of the 1972 song Funky Worm from the album *Pleasure* by the Ohio Players. The species name honours N. Gilmore, collections manager at the Academy of Natural Sciences of Drexel University in Philadelphia, PA, USA.

**Fig. 1:**
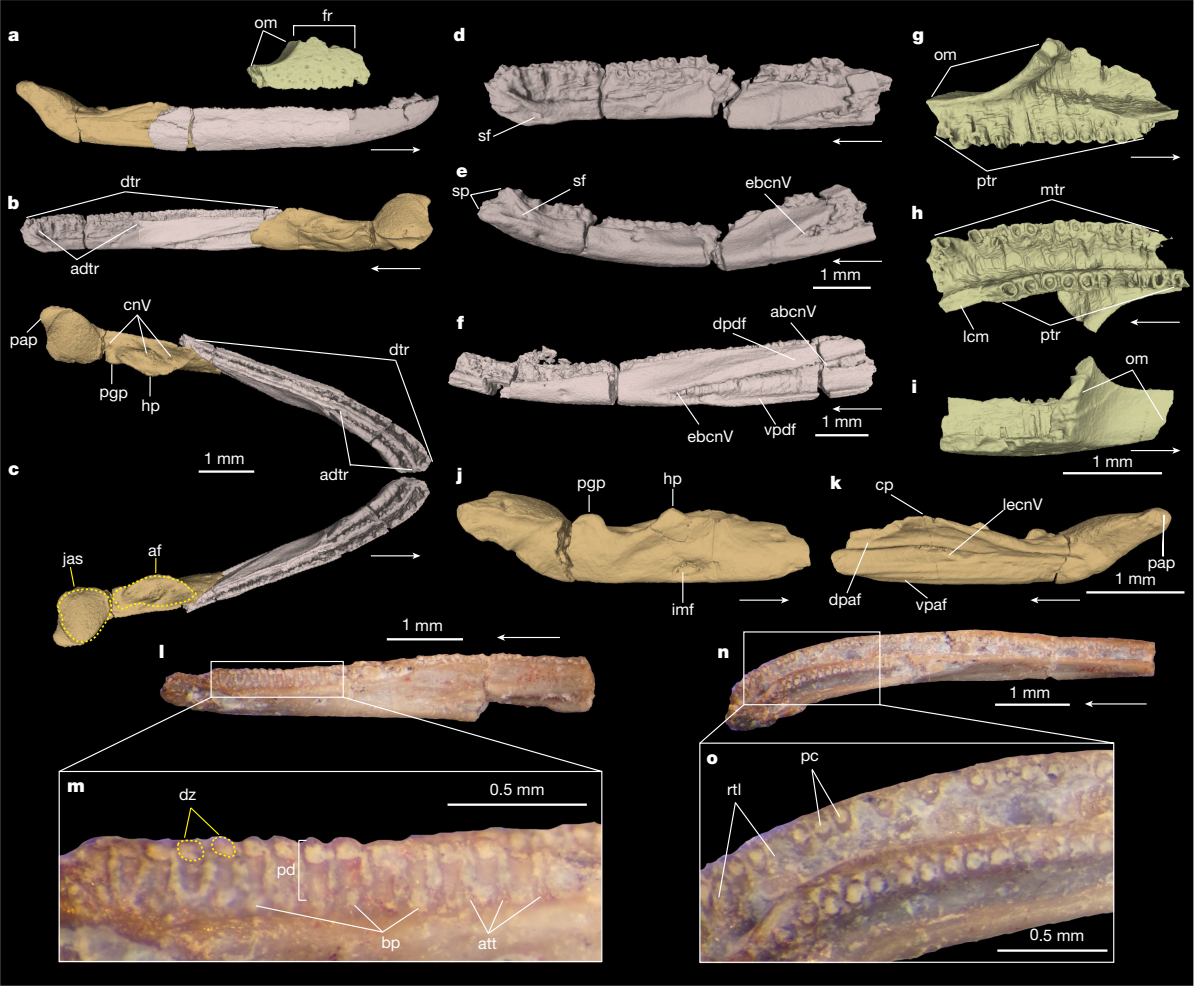
Digital renderings of holotype, paratype, and referred specimens of *F. gilmorei*. **a**–**c**, Composite reconstruction of craniomandibular elements in lateral (**a**), medial (**b**) and dorsal (**c**) views. **d**,**e**, Holotype right pseudodentary (PEFO 43891) in medial and ventral views. **f**, Paratype right pseudodentary (PEFO 46284) in medial view. **g**–**i**, Referred left maxillopalatine (PEFO 46481) in medial (**g**), ventral (**h**) and dorsal (**i**) views. **j**,**k**, Referred left pseudoangular (PEFO 46480) in medial and lateral views. **l**–**o**, Paratype right pseudodentary (PEFO 45800) in medial (**l**; expanded view in **m**) and dorsal (**n**; expanded view in **o**) views. abcnV, alveolar branch cranial nerve V; adtr, adsymphyseal tooth row; af, adductor fossa; att, attachment tissue; bp, basal pore; cnV, cranial nerve V insertions; cp, coronoid process; dpaf, dorsal pseudoangular facet; dpdf, dorsal pseudodentary facet; dtr, dentary tooth row; dz, dividing zone; ebcnV, external branch cranial nerve V; fr, facial ramus; hp, hamate process; imf, intramandibular foramen; jas, jaw articulation surface; lcm, lateral choanal margin; lecnV, lateral exit cranial nerve V; mtr, maxillary tooth row; om, orbital margin; pap, posterior pseudoangular process; pc, pulp cavity; pd, pedicel; pgp, preglenoid process; ptr, palatal tooth row; rtl, replacement tooth locus; sf, symphyseal foramen; sp, symphyseal prongs; vpaf, ventral pseudoangular facet; vpdf, ventral pseudodentary facet. Arrows indicate anterior direction.

**Extended Data Fig. 3:**
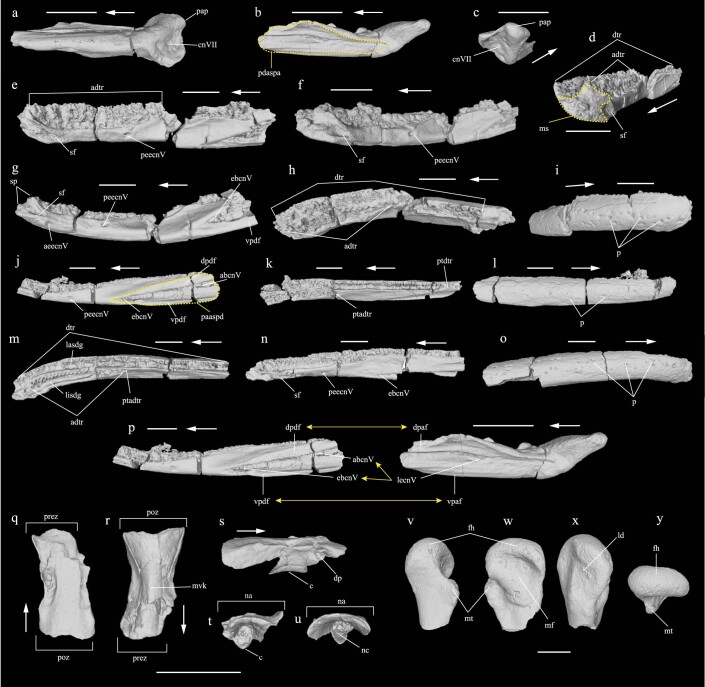
Anatomy of *Funcusvermis gilmorei* based on reconstructed 3D surface meshes from segmented micro-computed tomographic scan data. **a**–**c**, Referred left pseudoangular (PEFO 46480) in ventral, lateral, and posterior views. **e**–**i**, Holotype right pseudodentary (PEFO 43891) in medial, anteromedial, anterior, ventral, dorsal, and anterolateral views. **j**–**l**, Paratype right pseudodentary (PEFO 46284) in medial, dorsal, and lateral views. **m**–**o**, Paratype right pseudodentary (PEFO 45800) in dorsal, medial, and lateral views. **p**, Paratype right pseudodentary (PEFO 46284) and referred left pseudoangular (PEFO 46480) with yellow arrows showing matching facet surfaces and neurovascular canals. **q**–**u**, Referred postatlantal vertebra (PEFO 45810) in dorsal, ventral, right lateral, anterior, and posterior views. **v**–**y**, Referred right femur (PEFO 43811) in dorsal, ventrolateral, lateral, and proximal views. abcnV, alveolar branch cranial nerve V; adtr, adsymphyseal tooth row; aeecnV, anterior exit external branch of cranial nerve V; c, centrum; cnVII, cranial nerve VII insertion; dp, diapophysis; dpaf, dorsal pseudoangular facet; dpdf, dorsal pseudodentary facet; dtr, dentary tooth row; ebcnV, external branch cranial nerve V; fh, femoral head; lasdg, labial subdental groove; ld, lateral depression; lecnV, lateral exit cranial nerve V; lisdg, lingual subdental groove; mf, medial fossa; mkv; midventral keel; ms, mandibular symphysis; mt, medial trochanter; na, neural arch; nc, neural canal; p, pit; paaspd, pseudoangular attachment surface of the pseudodentary; pap, posterior pseudoangular process; pdaspa, pseudodentary attachment surface of the pseudoangular; peecnV, posterior exit external branch cranial nerve V; poz, postzygapophysis; prez, prezygapophysis; ptadtr, posterior terminus adsymphyseal tooth row; ptdtr, posterior terminus dentary tooth row; sf, symphyseal foramen; sp, symphyseal prongs; vpaf, ventral pseudoangular facet; vpdf, ventral pseudodentary facet. Arrows point in anterior direction. Scale bars equal 1 mm.

**Extended Data Fig. 4:**
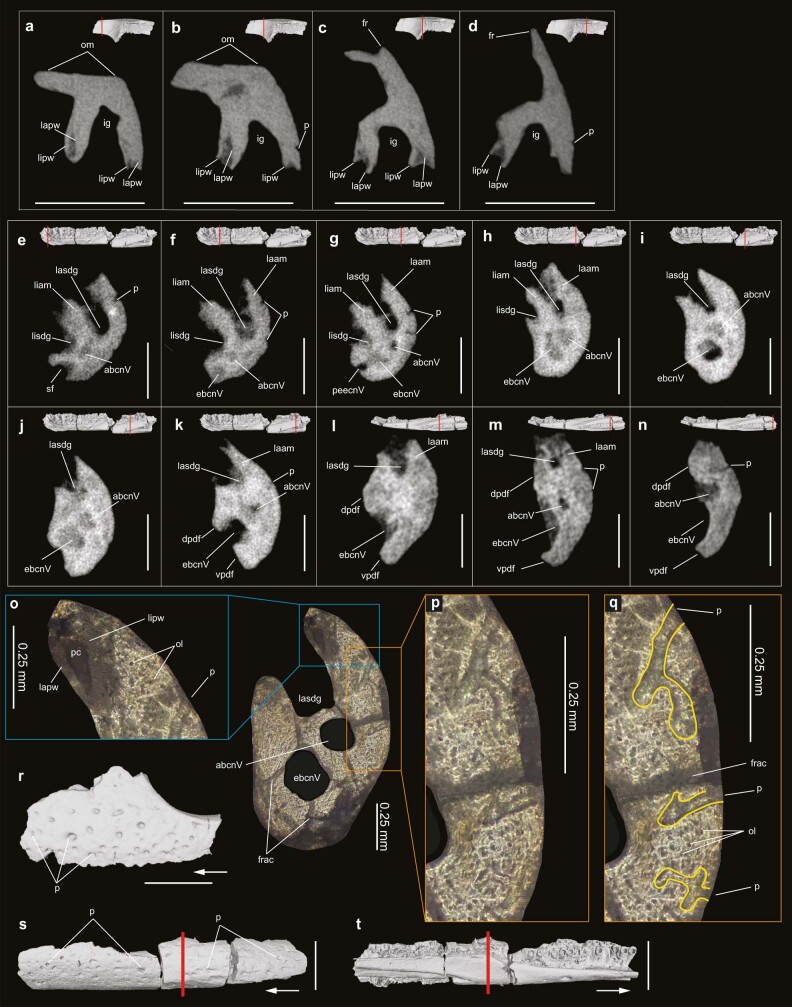
Cross sectional views of *Funcusvermis gilmorei* dentigerous elements from micro-computed tomographic scan data and osteohistological sectioning. **a**–**d**, Coronal cross sections of referred left maxillopalatine (PEFO 46481). **e**–**k**, Coronal cross sections of holotype right pseudodentary (PEFO 43891). **l**–**n**, Coronal cross sections of paratype right pseudodentary (PEFO 46284). **o**–**p**, Microanatomy from osteohistological section of paratype right pseudodentary (PEFO 44432) in coronal view. **r**, Referred left maxillopalatine (PEFO 46481) in lateral view. Paratype right pseudodentary (PEFO 44432) in lateral (**s**) and medial (**t**) views. abcnV, alveolar branch cranial nerve V; dpdf, dorsal pseudodentary facet; ebcnV, external branch cranial nerve V; frac, fractures; fr, facial ramus; ig, interdental groove; laam, labial alveolar margin; lapw, labial pedicel wall; lasdg, labial subdental groove; liam, lingal alveolar margin; lipw, lingual pedicel wall; lisdg, lingual subdental groove; oc, osteocyte lacunae; om, orbital margin; p, pit; pc, pulp cavity; peecnV, posterior exit external branch cranial nerve V; sf, symphyseal foramen; vpdf, ventral pseudodentary facet. Red lines indicate locations of cross sections. Yellow outlines denote margins of canals connected to external pits. Scale bars equal 1 mm unless otherwise noted.

**Holotype.** PEFO 43891, right pseudodentary (Fig. 1 and Extended Data Figs. 3 and 4), accessioned at Petrified Forest National Park, Arizona, USA.

**Paratypes.** PEFO 44432, PEFO 45800 and PEFO 46284 (all right pseudodentaries; Fig. 1 and Extended Data Figs. 3 and 4). Additional paratypes are listed in Supplementary Information, section 1.

**Referred material.** PEFO 46481, left maxillopalatine (Fig. 1 and Extended Data Fig. 4); PEFO 46480, left pseudoangular (Fig. 1 and Extended Data Fig. 3); PEFO 45810 (postatlantal vertebra), PEFO 43811 (right femur) (Extended Data Fig. 3). Additional referred specimens are listed in Supplementary Information, section 1.

**Type locality and horizon.** PFV 456, Thunderstorm Ridge, PEFO, Arizona, USA (Extended Data Fig. 2), within the upper Blue Mesa Member, Chinle Formation (Late Triassic: Norian); 223.036 ± 0.059 Ma (ref. ^24^ to 218.08 ± 0.037 Ma (ref. ^25^), or ~221 Ma (ref. ^26^); Adamanian estimated holochron^27^).

**Diagnosis.** A gymnophionomorph diagnosed by the following unique combination of features found in the holotype and paratype and referred specimens (asterisk denotes autapomorphies): symphyseal foramen* and notch subdividing the mandibular symphysis into medial and lateral processes*; at least 50 and at least 22 tooth pedicels in the dentary and adsymphyseal tooth rows, respectively. Further diagnosed by features found in referred specimens: co-ossified maxilla and palatine (compound maxillopalatine); palatal dentition of maxillopalatine terminated anteriorly by the lateral choanal margin*; maxillopalatine without osteological correlate of the tentacular organ*; absence of internal and retroarticular processes of the pseudoangular*; jaw articulation surface of pseudoangular formed by a subcircular flat pad; pseudoangular bearing a dorsally exposed adductor chamber occupying more than 30% of pseudoangular length*; three cranial nerve V insertions in pseudoangular*; femur present. Differential diagnosis in Supplementary Information, section 2.

## Phylogenetic relationships

**Extended Data Fig. 5:**
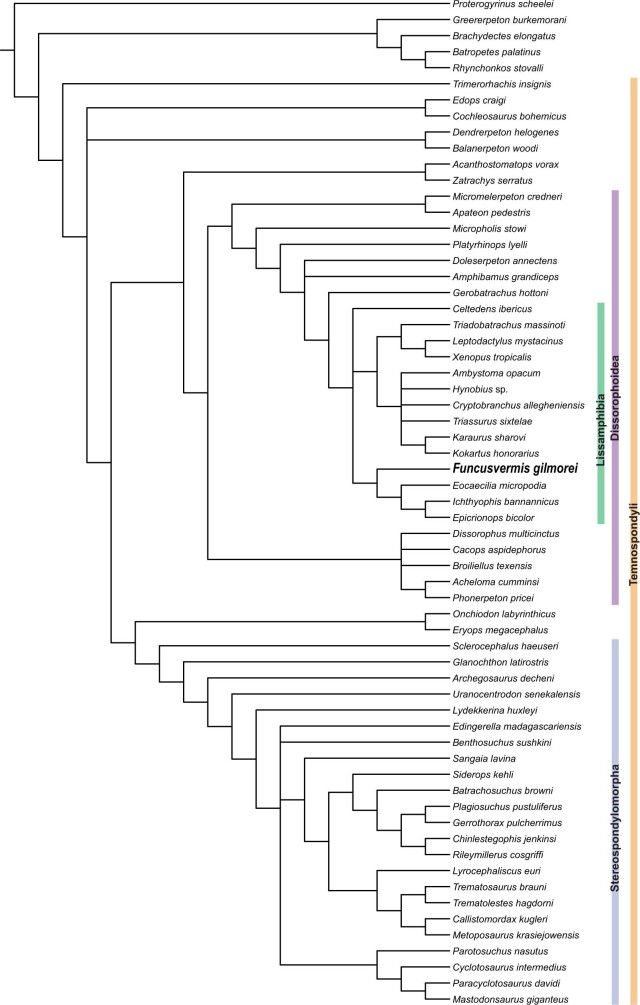
Phylogenetic tree from parsimony analysis. Strict consensus tree of 71 most parsimonious trees (1,468 steps each) from TNT parsimony analysis (Methods). CI = 0.287, RI = 0.675.

**Extended Data Fig. 6:**
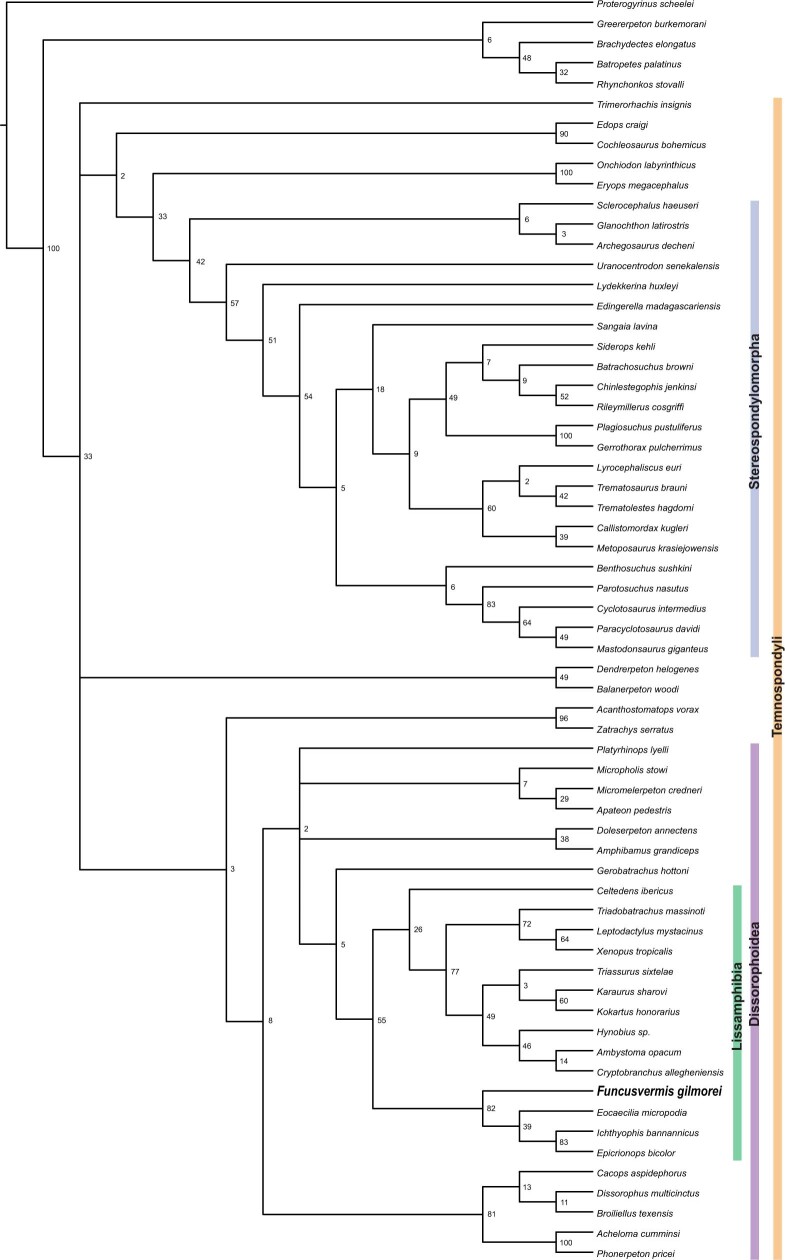
Phylogenetic tree from parsimony analysis showing bootstrap support. Strict consensus topology of trees produced via 1,000 bootstrap replicates resampled with replacement (Methods). Node values indicate bootstrap support.

**Extended Data Fig. 7:**
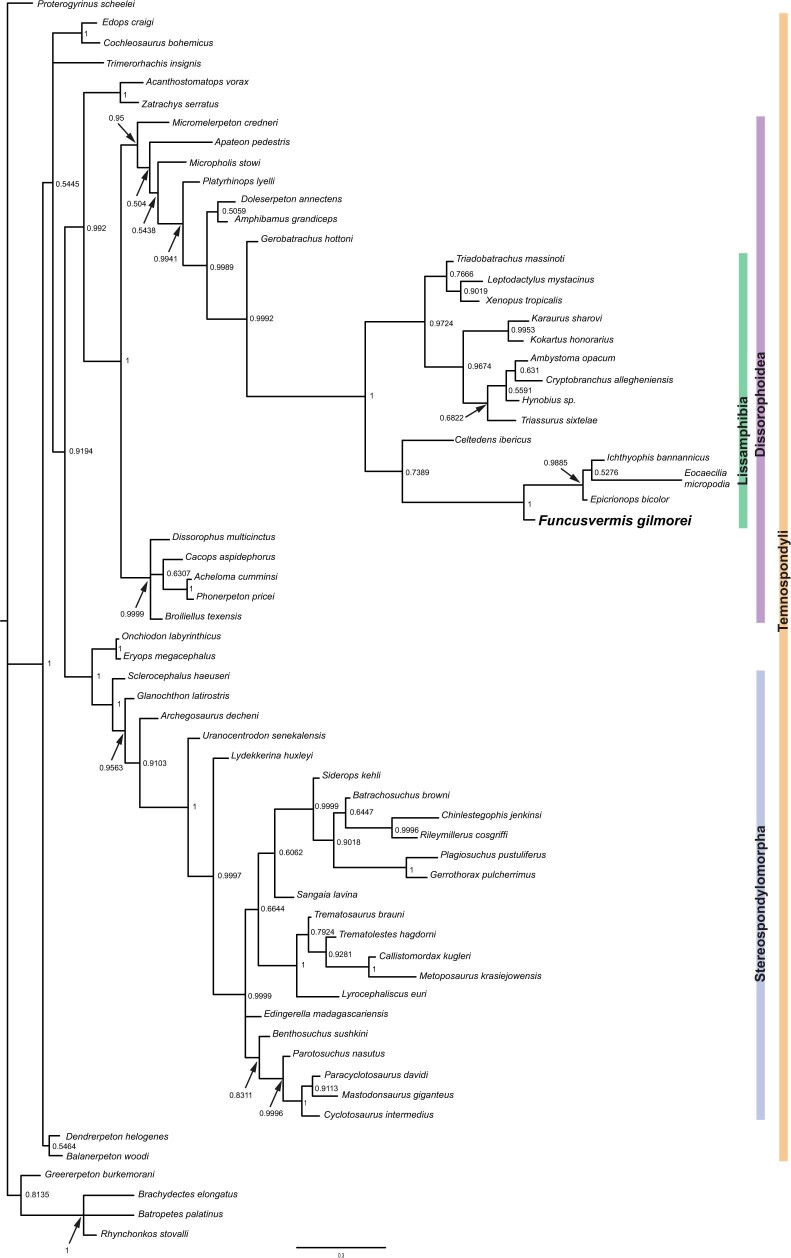
Topology from Bayesian inference phylogenetic analysis. Majority-rule consensus tree from Bayesian inference analysis (Methods). Node labels show posterior probability values.

**Extended Data Fig. 8:**
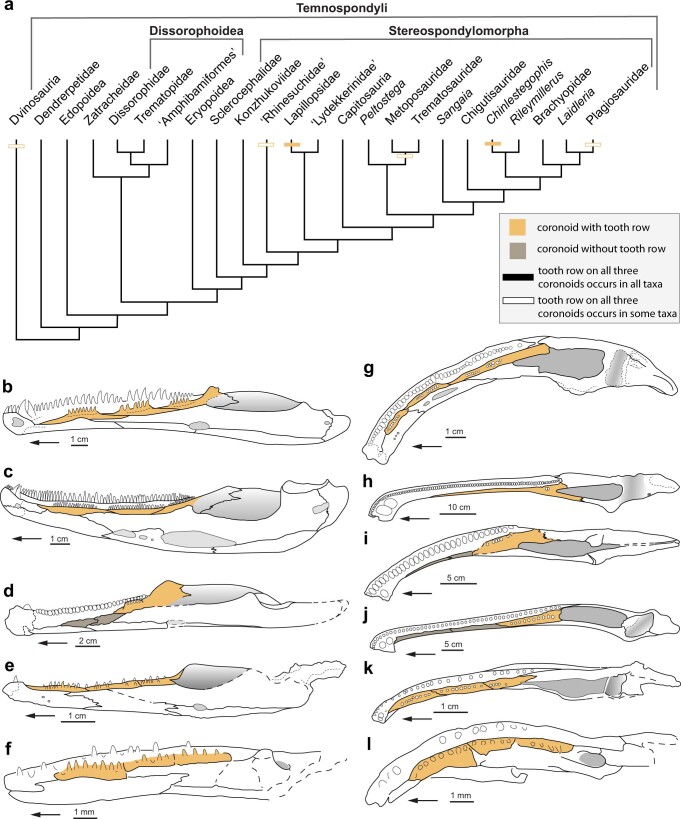
Phylogenetic distribution and comparative morphology of a single row of coronoid teeth in Temnospondyli. **a**, Phylogenetic topology (derived from refs. ^14,55^) with presence of a semi-continuous row of teeth across all three coronoids (See Supplementary Information 3 for further discussion of this feature). **b**–**k**, Comparative silhouettes of lower jaws in lingual (**b**–**g**), and dorsal (**h**–**m**), views. **b**, The dvinosaur *Dvinosaurus primus*^56^. **c**, The trematosaur *Almasaurus habbazi*^57^. **d**, The rhinesuchid *Rastosuchus hammeri*^58^. **e**, The brachyopid *Vanastega plurimidens*^59^. **f**, The stereospondyl *Chinlestegophis jenkinsi*^14^. **g**, The plagiosaurid *Gerrothorax pulcherrimus*^60^ (Illustration adapted from ref. ^60^ under CC BY 3.0 license). **h**, The capitosaur *Mastodonsaurus giganteus*^61^. **i**, The brachyopid *Hadrokkosaurus bradyi*^62^. **j**, The trematosaur *Benthosuchus sushkini*^63^. **k**, *Chinlestegophis jenkinsi*^14^. Arrows indicate anterior direction.

**Extended Data Fig. 9:**
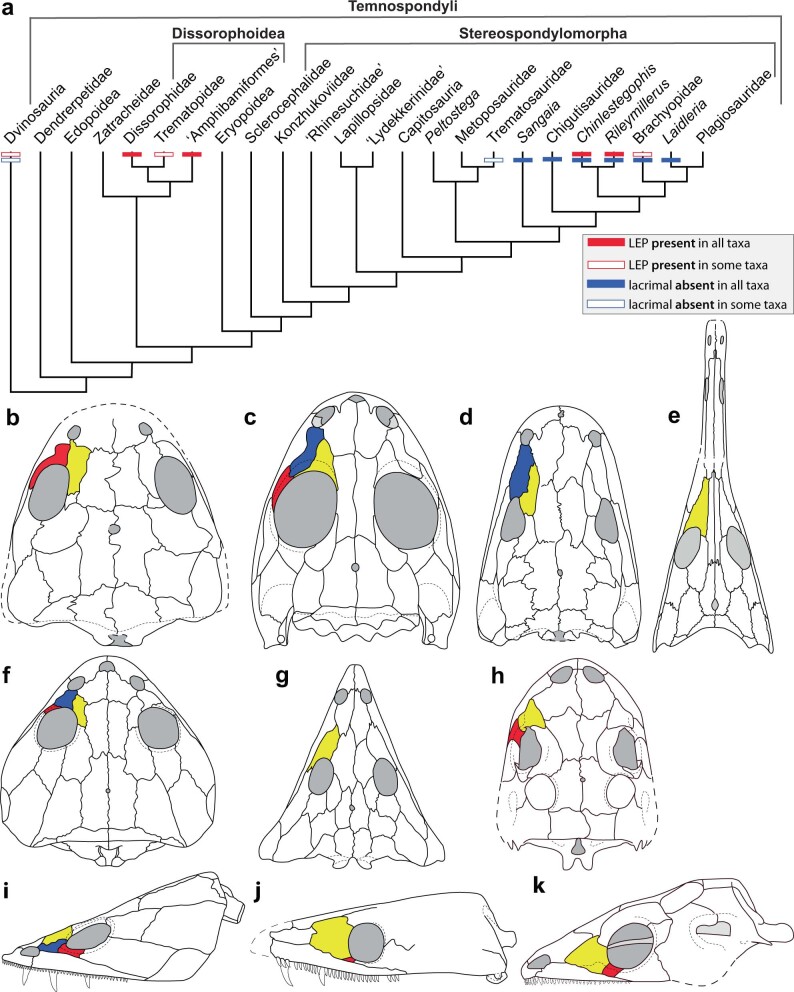
Phylogenetic distribution and comparative morphology of a lateral exposure of the palatine (LEP) and the loss of the lacrimal in Temnospondyli. **a**, Phylogenetic topology (derived from refs. ^14,55^) with variable presence of the lacrimal and the LEP (See Supplementary Information 3 for further discussion of this feature). Comparative silhouettes of skulls in dorsal **b**–**h** and lateral **i**–**k** views; the prefrontal is coloured yellow here as it is the main element to positionally compensate for an absent lacrimal. **b**, The dvinosaur *Thabanchuia oomie*^64^ (Illustration reproduced with permission from Cambridge University Press from ref. ^64^). **c**, The amphibamid *Doleserpeton annectens*^9^ (Illustration adapted from ref. ^9^ under copyright © Society of Vertebrate Paleontology, www.vertpaleo.org, reprinted by permission of Informa UK Limited, trading as Taylor & Francis Group, www.tandfonline.com on behalf of Society of Vertebrate Paleontology, www.vertpaleo.org. **d**, The ‘dendrerpetid’ *Dendrerpeton helogenes*^65^, which represents the plesiomorphic condition (Illustration adapted from ref. ^65^. under copyright © Society of Vertebrate Paleontology, www.vertpaleo.org, reprinted by permission of Informa UK Limited, trading as Taylor & Francis Group, www.tandfonline.com on behalf of Society of Vertebrate Paleontology, www.vertpaleo.org). **e**, The trematosaur *Wantzosaurus elongatus*^66^. **f**, The dvinosaur *Acroplous vorax*^67^ (Illustration adapted from ref. ^67^ under copyright © Society of Vertebrate Paleontology, www.vertpaleo.org, reprinted by permission of Informa UK Limited, trading as Taylor & Francis Group, www.tandfonline.com on behalf of Society of Vertebrate Paleontology, www.vertpaleo.org). **g**, the rhytidosteid *Laidleria gracilis*^68^ (Illustration adapted from ref. ^68^. by permission of the Zoological Journal of the Linnean Society). **h**, *Chinlestegophis jenkinsi*^14^. **i**, *Acroplous vorax*^67^ in lateral view. **j**, *Rileymillerus cosgriffi*^69^ in lateral view. **k**, *Chinlestegophis jenkinsi*^14^ in lateral view. The prefrontal is also coloured yellow here as it is the main element to positionally compensate for an absent lacrimal. Silhouettes are not to scale.

**Extended Data Fig. 10:**
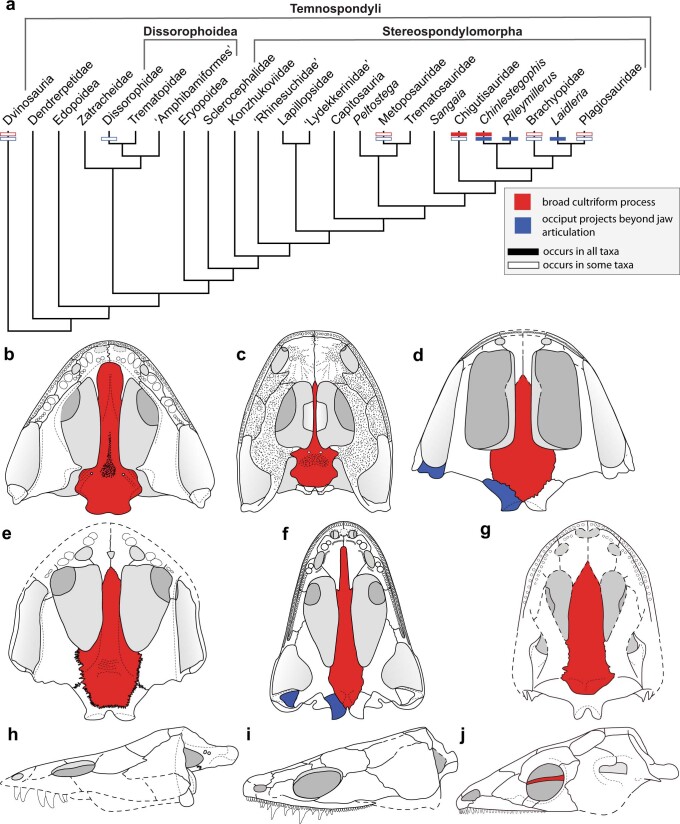
Phylogenetic distribution and comparative morphology of a broad cultriform process and a posteriorly situated occiput in Temnospondyli. **a**, Phylogenetic topology (derived from refs. ^14,55^) with presence of a broad cultriform process and a posteriorly situated occiput (See Supplementary Information 3 for further discussion of this feature). **b**–**j**, Comparative silhouettes of skulls in lateral **i**–**k**, and ventral **b**–**h**, views. **b**, The dvinosaur *Dvinosaurus primus*^56^. **c**, The ‘dendrerpetid’ *Balanerepeton woodi*^70,71^ (Illustration adapted from ref. ^70^. with permission from Cambridge University Press), which represents the plesiomorphic condition. **d**, The plagiosaurid *Plagiosuchus pustuliferus*^72^ (Illustration adapted from ref. ^72^. by permission of the Zoological Journal of the Linnean Society). **e**, The brachyopid *Batrachosuchus browni*^73^. **f**, The metoposaurid *Metoposaurus krasiejowensis*^74^. **g**, *Chinlestegophis jenkinsi*^14^. **h**, *Batrachosuchus browni*^73^. **i**, The dvinosaur *Thabanchuia oomie*^64^ (Illustration reproduced with permission from Cambridge University Press from ref. ^64^). **j**, *Chinlestegophis jenkinsi*^14^. Silhouettes are not to scale.

We tested the relationships of *Funcusvermis gilmorei* in a modified dataset^6^ of 63 terminal taxa including stem tetrapods, stem and crown amniotes, and temnospondyl amphibians including stereospondyls and lissamphibians (Methods). Using both maximum parsimony and Bayesian inference optimality criteria (Methods), our phylogenetic analyses robustly support *Funcusvermis* as the earliest-diverging gymnophionomorph, sister taxon to the clade including *Eocaecilia, Rubricacaecilia* and Gymnophiona. All analyses unambiguously recovered a monophyletic Lissamphibia nested within amphibamiform dissorophoids, with *Gerobatrachus* and *Doleserpeton annectens* as successive outgroups to Lissamphibia (Fig. 3 and Extended Data Figs. 5–7). Our parsimony analysis recovered Lissamphibia consisting of a polytomous trichotomy of Gymnophionomorpha, Batrachia and Albanerpetontidae (Extended Data Fig. 5), whereas our Bayesian analysis recovered Lissamphibia consisting of a sister group relationship between Batrachia and a clade comprised of a sister group relationship between Gymnophionomorpha and Albanerpetontidae (Extended Data Fig. 7). The varying position of albanerpetontids in these and other recent analyses^28^ highlights the ghost lineage from 150 million years ago (Ma) preceding their earliest occurrences in the Middle Jurassic epoch^29^ as an outstanding gap obscuring conclusive resolution of relationships amongst major lissamphibian lineages. These results suggest that the caecilian-like anatomy in *Chinlestegophis jenkinsi* (a Late Triassic diminutive burrowing stereospondyl also found in the Chinle Formation^14^) is convergent with that of gymnophionomorphs such as *Eocaecilia* because of adaptations facilitating fossoriality (further discussed in Supplementary Information, section 3 and Extended Data Figs. 8–10).

## Origins of the lissamphibian jaw apparatus

*Funcusvermis* indicates that many features of the lissamphibian mandibular ramus appeared initially in amphibamiform dissorophoids and were later lost or modified in batrachians, albanerpetontids and gymnophionomorphs (Fig. 3). All dentition in *Funcusvermis* (Fig. 1) is pedicellate—the oldest known example of this distinctive tooth form in crown Lissamphibia—reinforcing hypotheses that pedicellate teeth are derived in amphibamiform dissorophoids^10^, conserved in gymnophionomorphs and batrachians^30^, and lost in albanerpetontids^29^. The rod-like pseudodentary of *Funcusvermis* resembles that of *Eocaecilia*^12^ and the dentary of *Doleserpeton*^9^ in the presence of tightly packed homodont tooth pedicels in parallel labial (dentary) and lingual (adsymphyseal) rows. The symphyseal foramen, of similar form and position to those of albanerpetontids^31^, suggests that the Meckel’s cartilage never ossified at the mandibular symphysis, probably a conservation of the ancestral condition of temnospondyls^32^, and differing from the ossified condition of this element that forms a closed mandibular symphysis in batrachians and other gymnophionomorphs. As in *Doleserpeton*^9^, a vertical notch bisects the mandibular symphysis between the anterior termini of the dentary and adsymphyseal tooth rows forming medial and lateral processes in *Funcusvermis* (Fig. 1 and Extended Data Fig. 3); these are similar to the more pronounced symphyseal prongs of albanerpetontids (for example, in refs. ^28,29,31^), indicating that this feature may be ancestral to Lissamphibia and later lost in Batrachia and the common ancestor of *Eocaecilia* and Gymnophiona.

In *Funcusvermis*, the presence of 22 or more teeth in the adsymphyseal tooth row is similar to the more than 20 teeth reported in *Eocaecilia*^12^, suggesting a transformation of the condition exhibited in *Doleserpeton* (5–7 teeth^9^) through distal expansion via addition of new teeth. In living caecilians, the lingual tooth row forms embryonically on a distinct anlage that later ossifies to the medial surface of the dentary forming the medial part of the mandibular symphysis and the lingual tooth row of adult caecilians^33^. Recent identifications of a dorsally facing tooth-bearing adsymphyseal (equivalent to the parasymphyseal (plate)) medial to (and separate from) the dentary at the mandibular symphysis in early branching tetrapods^34,35^, juvenile temnospondyls^36^ and dissorophoid temnospondyls^37^ suggests that in taxa that appear to bear a lingual tooth row at the mandibular symphysis of the ‘dentary’ (for example, *Doleserpeton*, *Funcusvermis* and other gymnophionomorphs), the ‘dentary’ is actually composed of a tooth-bearing adsymphyseal (forming the lingual tooth row) co-ossified lingually to the dentary, and not a coronoid as previously thought^14,33^ (Fig. 3 and Extended Data Fig. 8; see Supplementary Information, section 2 for discussion of adsymphyseal homology).

The pseudoangular of *Funcusvermis* is highly similar to the postdentary morphology of dissorophoids exemplified by the amphibamid *Doleserpeton*^9^ (Fig. 3 and Extended Data Fig. 3); as in *Doleserpeton*, batrachians, and albanerpetontids, *Funcusvermis* lacks retroarticular and internal processes, suggesting their initial acquisition in the common ancestor of *Eocaecilia* and Gymnophiona. The absence of the retroarticular process and presence of a dorsally facing adductor fossa (insertion site of the m. adductor mandibulae complex (mAM)) occupying more than 30% of pseudoangular length (Supplementary Table 1) in the pseudoangular of *Funcusvermis* (Fig. 1) illuminate a major transformation from the ancestral lissamphibian condition to the unique musculoskeletal architecture of living gymnophionans. Jaw closure driven primarily by the mAM is ancestral for tetrapods, and is retained in batrachians, albanerpetontids and *Funcusvermis*, differing from the condition of all other gymnophionomorphs, which exhibit the distinctive caecilian dual jaw closure mechanism^15^ (DJCM). The DJCM is driven primarily by the hyobranchial muscle *m. interhyoidus posterior* (mIHP), and secondarily by the mAM; the mIHP inserts onto the ventral side of the retroarticular process and extends posteroventrally, acting as a first-order lever causing the anterior component of the lower jaw to pivot upwards with respect to the quadrate during jaw closure^15^. Acquisition of DJCM is hypothesized to be an adaptation for fossoriality: the mIHP contribution to bite force allows for reduction of the mAM and therefore compaction of the skull roof^15,16^, a suite of transformations shown to be acquired by the common ancestor of *Eocaecilia* and Gymnophiona. Although the skull roof of *Funcusvermis* is unknown, absence of the DJCM and our phylogenetic results suggest that it probably retained the plesiomorphic condition of cheek emargination (gymnokrotaphy, as in *Gerobatrachus*, batrachians, albanerpetontids and presumably the common ancestor of Lissamphibia) to accommodate the mAM, rather than a closed skull roof with large interpterygoid vacuities^13^ (stegokrotaphy, as in dissorophoids).

The obtuse angle of the orbital margin in the *Funcusvermis* maxillopalatine (Fig. 1) may suggest the presence of large orbits as in dissorophoids, batrachians and albanerpetontids (differing from the reduced orbits of other gymnophionomorphs); however, the incomplete orbital margin in the single maxillopalatine specimen (PEFO 46481) prohibits conclusive assessment of this feature. The orbital margin of *Funcusvermis* lacks a tentacular fossa or aperture (osteological correlates for the chemosensory tentacle organ^18^), suggesting its absence in early gymnophionomorphs and later derivation by the common ancestor of *Eocaecilia* and Gymnophiona^12^. The presence of a co-ossified maxilla and palatine (maxillopalatine) in *Funcusvermis* is shared with gymnophionans and differs from that of amphibamiforms, albanerpetontids and batrachians, evidence of maxillopalatine consolidation early in gymnophionomorph evolution; however, these bones are possibly separate in *Eocaecilia*^12^ and *Rubricacaecilia*^23^. Ventrally, the maxillopalatine of *Funcusvermis* bears parallel maxillary and palatal rows of tightly packed pedicellate teeth of similar size to those in the pseudodentary, seemingly intermediate between the condition of these dentitions in *Doleserpeton* and *Eocaecilia*, sharing an anterior truncation of the palatal tooth row by the internal nares with the former, and mesiodistal distal extension (through addition of new teeth) of the palatal row with the latter. A comprehensive comparative description of the *Funcusvermis* skull and postcranial elements is included in Supplementary Information, section 2.

## Evolution of caecilian fossoriality

Given our phylogenetic results, the ecological habits of *Funcusvermis* may be transitional between terrestrial amphibamid dissorophoids and fossorial gymnophionans. The compound bones in the compact skull of fossorial gymnophionans are thought to withstand the forces associated with head-first burrowing^17^, and at least some are present in *Funcusvermis* (for example, maxillopalatine). Small pits covering the lateral surfaces of the pseudodentary and maxillopalatine in *Funcusvermis* are also found in *Eocaecilia*^12^, *Rubricacaecilia*^23^ and gymnophionans^38^. External structure and internal microanatomy of these pits revealed by osteohistological sectioning of a *Funcusvermis* pseudodentary (PEFO 44432) show a marked resemblance to those of studied living caecilians (Extended Data Fig. 4), in which these pits act as anchor sites for collagen networks forming a tight skin-to-bone attachment and house glands that produce a lubricating mucus secretion, functions thought to aid in subterranean burrowing^39^. The dorsally flattened neural arch of the *Funcusvermis* postatlantal pleurocentrum (PEFO 45810; Extended Data Fig. 3) resembles those of *Rubricacaecilia*^23^, suggesting the acquisition of a tubular trunk, a feature crucial for underground locomotion in living caecilians^40^. These morphologies in *Funcusvermis* illustrate acquisition (by at least the Late Triassic) of some features that now facilitate fossoriality in living caecilians, later followed by acquisition of the DJCM and tentacular organ in *Eocaecilia*, and finally loss of the appendicular skeleton in gymnophionans.

## Biogeography of early caecilians

**Fig. 2:**
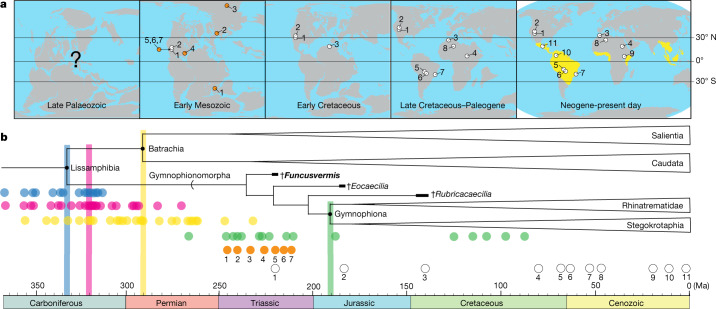
Spatiotemporal history of Lissamphibia and Gymnophionomorpha. **a**, Biogeographic history of Gymnophionomorpha and Triassic batrachians; yellow indicates living caecilian distribution. **b**, Time-calibrated topology of lissamphibian relationships showing major divergences (topology derived from refs. ^6,23,38^). Estimated molecular divergence dates for major divergences are shown as blue circles (Gymnophionomopha–Batrachia divergence without *Gerobatrachus* calibration; Supplementary Table 4), pink circles (Gymnophionomopha–Batrachia divergence with *Gerobatrachus* calibration; Supplementary Table 5), yellow circles (Salientia–Caudata divergence; Supplementary Table 6) and green circles (Rhinatrematidae–Stegokrotaphia divergence; Supplementary Table 7); coloured vertical bars show the average for each set of divergence estimates. Numbered white and orange circles correspond to occurrences in Supplementary Tables 2 and 3, respectively. Crosses indicate extinct taxa.

The spatiotemporal occurrence of *Funcusvermis* empirically establishes lissamphibian geographic origins on the Pangaean supercontinent before its fragmentation^20^, and the similar palaeogeography of *Eocaecilia*^12^ to *Funcusvermis* suggests the non-gymnophionan gymnophionomorph origin may lie in the early Mesozoic era of equatorial central Pangaea. The occurrence of *Rubricacaecilia* in the Early Cretaceous epoch of equatorial Gondwana may further support this hypothesis, suggesting non-gymnophionan gymnophionomorph distribution across both Laurasian and Gondwanan components of Pangaea in the early Mesozoic prior to its breakup^23^. The equatorial provenance of *Funcusvermis* adds to an exclusively equatorial pattern of gymnophionomorph distribution: all fossil occurrences fall between a minimum of approximately 16° N and 27° S (Fig. 2 and Supplementary Table 2), and living caecilians are restricted to equatorial latitudes^19^ between 27° N and 34° S. The tropical distribution of extant gymnophionans is notably disjunct from non-gymnophionan gymnophionomorph fossil occurrences in present-day western North America and Morocco (Fig. 2). Drift of the North American and African plates during the Mesozoic^41^ may explain the extirpation of gymnophionomorphs from these areas later in the Phanerozoic as these previously humid palaeotropical regions moved north into the arid subtropics. Concurrently, the northern drift of Gondwana into the palaeotropics may have expanded suitable terrestrial habitats, consistent with molecular evidence of an early Mesozoic Gondwanan origin of gymnophionans^20^.

**Fig. 3:**
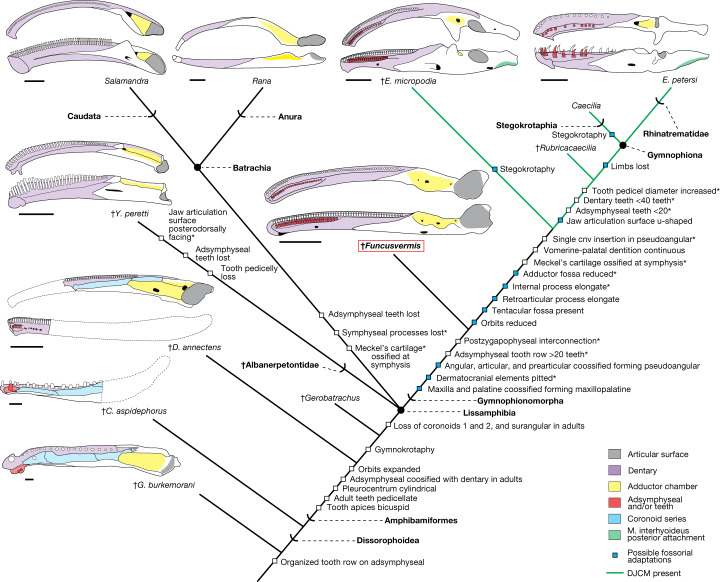
Evolutionary history of the lissamphibian mandibular ramus. Squares denote important apomorphies (including non-mandibular features); apomorphies are optimized computationally unless followed by an asterisk, which denotes an apomorphy suggested by our results but lacking sufficient sampling to optimize computationally. Topology is derived from parsimony results (Extended Data Fig. 5); *Yaksha peretti*, *Salamandra* and *Rana* approximate conditions are found in taxa sampled in the analysis. Illustrations represent right mandibles in medial (bottom) and dorsal (top) views for *Doleserpeton annectens*^9^, *Eocaecilia micropodia*^12^ (Illustration adapted from ref. ^12^, with the permission of Museum of Comparative Zoology, Harvard University), *Epicrionops petersi*^12^, *Funcusvermis gilmorei*, *Rana*, *Salamandra* and *Y. peretti*^28^, excepting *Greererpeton burkemorani*^35^ (dorsal only) and *Cacops aspidephorus*^37^ (medial only). All scale bars are 2 mm except for *G. burkemorani* (2 cm) and *C. aspidephorus* (2 cm). Brackets on the branches indicate stem groups, whereas circles indicate node groups. Crosses indicate extinct taxa.

The earliest batrachians hail from the Triassic of southern^2^, equatorial^4,5^ and northern^3,6^ Pangaea (Supplementary Table 3), indicating extensive latitudinal dispersal by at least the Middle Triassic epoch; this pattern is further reflected in the subsequent batrachian fossil record and their extant distribution. Unlike in extant batrachians, evaporative water loss is found to be a critical physiological constraint in living caecilians, limiting their distribution to humid environments near the equator^19^. The contrasting spatiotemporal histories of batrachians and gymnophionomorphs suggest a divergence of physiological constraints linked to humidity prior to the Triassic; conserved physiological traits in these groups may explain subsequent patterns of dispersal reflected in present-day lissamphibian biogeography.

## Timing of lissamphibian origins

Prior to the results of this study, the chronology of lissamphibian origins remained unresolved owing to the reliance of molecular clock estimates on different node minima derived from competing phylogenetic hypotheses that include extinct taxa^1^. *Funcusvermis* lends novel and strong support for a monophyletic origin of living amphibians within dissorophoid temnospondyls^30,42^ (the ‘classic’ temnospondyl hypothesis), and thus the molecular clock estimates of caecilian–batrachian divergence using the temnospondyl hypothesis. Additionally, the recovery of *Gerobatrachus* as the sister taxon to Lissamphibia in our analysis suggests that taxon may not be a stem batrachian^7,8^ and should be used with caution as a minimum age calibration for Lissamphibia. Molecular clock estimates using the temnospondyl hypothesis topology unconstrained by *Gerobatrachus* as the minimum age calibration of Lissamphibia may result in the most accurate estimates of the caecilian–batrachian divergence, and studies following these criteria show divergence time estimates ranging from the Late Devonian (367.0 Ma) to Middle Pennsylvanian (314.8 Ma) epochs, with a mean in the Middle Mississippian (333.5 Ma) and a median in the Late Mississippian (325.6 Ma) epoch (Fig. 2b and Supplementary Table 4). Our results refocus the timeframe of lissamphibian origins to the Mississippian subperiod, older than previous estimates of a Pennsylvanian-Permian divergence based on calibrations using *Gerobatrachus* or *Amphibamus grandiceps*^1^ and those considering Gymnophionomorpha as the sister group to the stereospondyl *Chinlestegophis*^1,14^ (Fig. 2 and Supplementary Table 5).

## Methods

### New phylogenetic definition

Gymnophionomorpha Marjanović and Laurin 2008

**Remarks**. Gymnophionomorpha is defined here as the total group consisting of *Caecilia tentaculata* and all taxa that share a more recent common ancestor with it than with *Salamandra* *salamandra* Linnaeus, 1758, *Rana temporaria* Linnaeus, 1758, and *Albanerpeton inexpectatum*, Estes and Hoffstetter 1976. This newly proposed stem-based definition of Gymnophionomorpha is modified after that originally proposed^43^.

### Assignment of elements

Although all specimens assigned to *Funcusvermis* were found as isolated, dissociated elements, their assignment to a single gymnophionomorph taxon is supported by: (1) specimens bearing a suite of features present exclusively in gymnophionomorphs to the exclusion of all other tetrapods (Supplementary Information, section 2); (2) skeletal elements represented by multiple specimens (77 pseudodentaries and 8 pseudoangulars) where all are identical in morphology, varying only in size (Supplementary Information, sections 1 and 2); (3) the pseudodentary and pseudoangular bear complementary facets where they would overlap when in articulation (Extended Data Fig. 3).

### Geological framework

The blue-coloured strata of the upper Blue Mesa Member of the Chinle Formation were deposited in a northwest-flowing fluviolacustrine system on the western margin of central Pangaea at a palaeolatitude of 5° to 15° N in a humid monsoonal climate^26^. Detrital zircon U-Pb radiometric ages provide robust geochronologic constraints on the Chinle Formation, bracketing deposition of the upper Blue Mesa Member^24,25^ to ~223–218 Ma (Extended Data Fig. 2). The gymnophionomorph fossils described herein were collected from the Thunderstorm Ridge locality (PFV 456) near the Puerco River in PEFO, Arizona, USA (Extended Data Fig. 2). The fossiliferous unit is a 15-cm-thick, poorly sorted siltstone horizon, bearing a dense concentration of carbonate nodules, angular intraformational clasts, micro- and macrovertebrate bones and coprolites. PFV 456 has yielded a diverse assemblage of vertebrates including chondrichthyans, actinopterygians, dipnoans, coelacanths, metoposaurids, salentians^5^, drepanosauromorphs^44^, lepidosauromorphs, archosauromorphs^45^, pseudosuchian archosaurs^46^, dinosauromorphs^47^ and cynodonts^48^. The lack of abrasion and polishing and the exceptional three-dimensional preservation of extremely delicate microvertebrate bones indicates initial deposition in a low-energy setting, followed by brief reworking and redeposition in a channel avulsion event that incorporated angular intraformational clasts and carbonate nodules into the fossiliferous layer. This sedimentological evidence in combination with the presence of abundant spinicaudatan exoskeletons, unionid bivalve steinkerns and obligate-aquatic, amphibious and fully terrestrial vertebrates indicates initial deposition in a marginal lacustrine palaeoenvironment occupied by a diverse vertebrate community.

### Collection and preparation methods

The hypodigm and all referred specimens were collected by screenwashing fossiliferous matrix from PFV 456 (9 out of 11 fossil gymnophionomorph occurrences were recovered using screenwashing; Supplementary Table 2). Blocks of matrix weighing approximately 1.8–3.2 kg were individually disaggregated in water and subsequently washed through a series of wire mesh screens with a minimum screen opening of 0.5 mm (no. 35 mesh). Dividing the fossiliferous concentrate from each block into smaller fractions in this way accelerated the process of picking. The resulting concentrate fractions were picked using a dissecting microscope resulting in the identification and separation of all *Funcusvermis* specimens. Importantly, through processing individual blocks of matrix, *Funcusvermis* elements that fragmented into multiple parts during the screenwashing process could be re-associated after microscopic sorting. Elements found as multiple broken pieces were subsequently reassembled by adhering matching fractured surfaces using cyanoacrylate, typically a low viscosity PaleoBOND or Loctite brand. To facilitate rapidly and precisely adhering these miniscule fragments together we created a mechanism that combines aspects of a jeweler’s block ball vice, and a hobbyist tool, sometimes called a third hand or helping hand. It combines a socket made of wood or closed cell polyethylene foam and a hemispherical wooden ball to create a pivot that can turn or tilt in all directions. This is topped with a small rectangle of wood with a small concave arch cut into it to provide a workspace. Insect pins are slid through channels in the wood filled with soft microcrystalline wax, which allows the pins freedom of movement, but the resistance needed to precisely position the fossil fragments. The fragments are temporarily adhered to the pin tips with more microcrystalline wax. Adhesive was applied to the joint between fragments as a microdroplet suspended on a single filament such as a cotton fibre and drawn into the joint via capillary action leaving a minimum of excess residue. Reassembly took place under a variety of Leica and Wild binocular microscopes, primarily MZ6, MZ12 and M8 models, varying in power from a maximum of ×40–×80 magnification.

To reveal the details of the pseudodentary dentition of *Funcusvermis*, matrix covering the dentition and other anatomy of PEFO 45800 was prepared through the following process. Melted cyclododecane (CDD) was poured into a shallow ceramic watch glass and allowed to harden. A small trench the size of the specimen was excavated, and the specimen was placed in the trench in the desired orientation. A Ukrainian *kistky* (a wax pen), was used to melt the CDD around the specimen and allowed it to adhere to and support the specimen. Then matrix was removed using a 1/32 inch (0.79375 mm) carbide-needle in a pin vice primarily under high magnification under a Leica MZ12 and MZ6 microscope. The point of the needle was ground to a superfine conical point at about 10°–15° parallel to the shaft and flattened briefly along one side to provide an edge to remove adhesives. Some of the softer clay particles were removed with a porcupine quill. When needed, the specimen was consolidated with a very dilute solution of polyvinyl butyral (Butvar B-76) in acetone; the solution was mixed by eye, applying a bit to another vertebrate bone fragment and looking for sheen upon drying. Any excess Butvar film was removed by abrasion with the porcupine quill. The specimen was rotated in the CDD by trenching around the specimen until it was loose, shifting it, and then remelting the resulting CDD powder with the *kistky*. After all matrix was removed, the specimen was trenched out a final time and set aside in the fume hood to allow the CDD to sublimate.

### Digital photography methods

Photographs of PEFO 45800 in Fig. 1 were acquired using a Leica MZ67 stereomicroscope and a Sony NEX-5T digital camera. Image stacking was conducted in Adobe Photoshop CC (https://www.adobe.com/products/photoshop.html).

### Micro-computed tomographic scan methods

PEFO 44432, PEFO 45800, PEFO 45910, PEFO 46284, PEFO 46480 and PEFO 46481 were CT scanned with a Skyscan 1172 Microfocus X-radiographic Scanner at the Virginia Tech Institute for Critical Technology and Applied Science (ICTAS). PEFO 43891 was scanned with a Nikon XTH 225 ST High-Resolution X-ray Computed Tomography Scanner in the Shared Materials Instrumentation Facility at Duke University. Micro-computed scan parameters (resolution, source voltage, source current and scanning equipment type) for each scanned specimen included in Supplementary Table 8. Surface volume files (3D meshes) of specimens figured in Fig. 1 and Extended Data Figs. 3 and 4 are available for download under project 000382289 at Morphosource.org (https://www.morphosource.org/projects/000382289?locale=en).

### 3D segmentation methods

Scan datasets were processed using Dragonfly 2020.2 (http://www.theobjects.com/dragonfly) to produce 3D virtual reconstructions. PEFO 43891, PEFO 46284, and PEFO 46481 were segmented in Dragonfly 2020.2 to digitally remove matrix covering parts of the specimens.

Images of 3D surface meshes were produced using Meshlab 2021.07 (https://www.meshlab.net/).

### Digital reconstruction methods

A composite reconstruction of a partial skull of *Funcusvermis* (Fig. 1) was produced using Meshmixer 3.5 (https://meshmixer.com). Digital 3D surface meshes representing the anterior (PEFO 43891; light pink in Fig. 1a–c) and posterior (PEFO 46284; dark pink in Fig. 1a–c) portions of a pseudodentary were scaled to the same dorsoventral height, and both specimens were overlapped to form a composite reconstruction of a complete pseudodentary. The pseudoangular (PEFO 46480) and maxillopalatine (PEFO 46481) were scaled to match the size of the reconstructed pseudodentary, and anatomically positioned relative to the pseudodentary to approximate their position in an articulated three-dimensional skull. A surface volume file (3D mesh) of the composite skull reconstruction is available for download under project 000382289 on Morphosource.org (https://www.morphosource.org/projects/000382289?locale=en).

### Osteohistology methods

PEFO 44432 (right pseudodentary) was embedded in clear epoxy (Castolite AP), cut into 1 mm sections, and then ground to a ~100 µm thickness in the Virginia Tech Fossil Preparation Lab. Images of the histologically sectioned pseudodentary slide used in Extended Data Fig. 4 were acquired using a Sony NEX-5T digital camera mounted on a Nikon OPTIPHOT-POL Polarizing microscope. Fracturing of the specimen occurred during osteohistological preparation, causing fracture planes apparent in histological imaging (Extended Data Fig. 4).

### Phylogenetic methods

See ‘Code availability’ to access and download phylogenetic matrix and analysis scripts.

### Taxon sampling

Recent analyses recovered gymnophionomorphs at variable positions within Tetrapoda dependent on character and taxon sampling, including: (1) as ‘microsaur’ ‘lepospondyls’^49^ (note that taxa formerly included in ‘Lepospondyli’ are now understood as polyphyletic^50^); (2) as stereospondyl temnospondyls forming the sister group to *C. jenkinsi*^14^; (3) as ‘microsaurian’ or aïstopod ‘lepospondyls’^51^; and [4] as amphibamiform dissorophoid temnospondyls forming the sister group to batrachians^6,13^. The matrix of Schoch et al. (2020), recently used to hypothesize the phylogenetic position of the stem salamander *Triassurus sixtelae* and the origin of lissamphibians, was selected to test the phylogenetic relationships of *F. gilmorei* given its comprehensive sampling of taxa proposed to be sister groups to Gymnophionomorpha including stem and crown amniotes, stereospondyl and dissorophoid temnospondyl amphibians, batrachians, gymnophionomorphs and albanerpetontids. *F. gilmorei* was coded into the modified Schoch et al. (2020) matrix, for a total of 63 sampled terminal taxa. See Supplementary Information, section 4 for discussion of taxon sampling.

### Character sampling and scoring

Modifications to the Schoch et al. (2020) matrix are detailed in Supplementary Information, section 4 and include addition of new characters, modification of preexisting characters, exclusion of preexisting characters, and recodings of preexisting character states. *Funcusvermis* was coded for 29 characters in total based on currently known skeletal material (Supplementary Table 9). The final matrix includes 355 morphological characters (Full character list in Supplementary Information, section 8; see ‘Code availability’ to access and download phylogenetic matrix and analysis scripts).

### Maximum parsimony and Bayesian analysis

All characters were equally weighted and unordered in both analyses following previous versions^6,14^. The character–taxon matrix was first analysed in the phylogenetic analysis software package TNT 1.5 (ref. ^52^) using New Technology Search options with the following parameters: ratchet (1,000 iterations), sectoral search (1,000 rounds), tree fusing (100 rounds), and random additional sequence (1,000 replicates). A total of 71 most parsimonious trees of 1,468 steps each were recovered (consistency index = 0.287; retention index = 0.675). A strict consensus tree calculated from the most parsimonious trees is presented in Extended Data Fig. 5. Bootstrap support values were obtained using TNT 1.5, and a strict consensus topology of trees produced via 1,000 bootstrap replicates resampled with replacement is presented in Extended Data Fig. 6. A Bayesian inference analysis of the character–taxon matrix was conducted in the phylogenetic software package MrBayes v.3.2.6 (ref. ^53^) with the Mkv^54^ model and gamma rate variation and the following parameters: four runs (six Markov chain Monte Carlo chains each), sampled every 1,000 generations, for 10 million generations with a relative burn-in of 0.25. Convergence of independent runs was assessed using Tracer v.1.76.1 (http://beast.bio.ed.ac.uk/Tracer). A consensus cladogram with mapped posterior probability values is presented in Extended Data Fig. 7.

### Nomenclatural acts

The Life Science Identifiers (LSID) for the new genus and species are registered with Zoobank (http://zoobank.org) under the identifiers urn:lsid:zoobank.org:pub:A2A6C7AD-2077-413B-9004-2E841270A289.

### Reporting summary

Further information on research design is available in the Nature Portfolio Reporting Summary linked to this article.

## Online content

Any methods, additional references, Nature Portfolio reporting summaries, source data, extended data, supplementary information, acknowledgements, peer review information; details of author contributions and competing interests; and statements of data and code availability are available at 10.1038/s41586-022-05646-5.

## Supplementary information

Supplementary Information This supplementary information file contains the following sections: 1. Complete list of material assigned to *F. gilmorei*. 2. Expanded description of *F. gilmorei*. 3. Comparisons to stereospondyl temnospondyls and *C. jenkinsi*. 4. Revisions and additions to phylogenetic dataset. 5. Supplementary Tables 1–9. 6. Institutional abbreviations. 7. Supplementary references. 8. Character list and phylogenetic datasets.

Reporting Summary

## Data Availability

The holotype, paratypes and referred specimens of *F. gilmorei* are catalogued and available for study to qualified researchers at PEFO. Computed tomographic scan data, including surface volume files (3D meshes) and raw CT data of *Funcusvermis* specimens mentioned in the main text and extended data figures (including the holotype, paratypes and referred specimens), as well as a surface volume file of the composite skull reconstruction of *Funcusvermis* are available for download under project 000382289 on Morphosource.org (https://www.morphosource.org/projects/000382289?locale=en).

## Code availability

Code for TNT and MrBayes scripts used in the phylogenetic analyses conducted herein are available in Supplementary Information, section 8; the matrix is available for download under project 4166 on Morphobank.org (http://morphobank.org/permalink/?P4166).
