# Supplementary material for: Triassic stem caecilian supports dissorophoid origin of living amphibians
Source: Nature. 2023 Jan 25;614(7946):102–7. doi: 10.1038/s41586-022-05646-5 (PMC9892002; doi:10.1038/s41586-022-05646-5)
Supplement: Supplementary file 1 — This supplementary information file contains the following sections: 1. Complete list of material assigned to F. gilmorei. 2. Expanded description of F. gilmorei. 3. Comparisons to stereospondyl temnospondyls and C. jenkinsi. 4. Revisions and additions to phylogenetic dataset. 5. Supplementary Tables 1–9. 6. Institutional abbreviations. 7. Supplementary references. 8. Character list and phylogenetic datasets. [file 41586_2022_5646_MOESM1_ESM.docx]

**Supplementary Information**

**Triassic stem caecilian supports dissorophoid origin of living amphibians**

Ben T. Kligman, Bryan M. Gee, Adam D. Marsh, Sterling J. Nesbitt, Matthew E. Smith, William G. Parker, Michelle R. Stocker

**Table of Contents**

1. **Complete list of material assigned to *Funcusvermis gilmorei*** [pp. 2–3]
2. **Expanded description of *Funcusvermis gilmorei*** [pp. 3–17]
3. **Comparisons to stereospondyl temnospondyls and *Chinlestegophis jenkinsi*** [pp. 18– 32]
4. **Revisions and additions to phylogenetic dataset** [pp. 33–106]
5. **Supplementary Tables** [pp. 108–116]

**1. Adductor fossa to pseudoangular length ratios** [p. 108]

**2. Fossil gymnophionomorph occurrence data** [p. 109]

**3. Triassic batrachian occurrence data** [p. 110]

**4. Molecular clock estimates of Gymnophionomorpha-Batrachia divergence without *Gerobatrachus* calibration** [p. 111]

**5. Gymnophionomorpha-Batrachia divergence data with *Gerobatrachus* calibration** [p. 112]

**6. Molecular clock estimates of Salientia-Caudata divergence** [p. 113]

**7. Rhinatrematidae-Stegokrotaphia divergence data** [p. 114]

**8. Micro-computed tomographic scan parameters** [p. 114]

**9. Character and character state coding for *Funcusvermis gilmorei*** [p. 115]

1. **Institutional abbreviations** [p. 116]
2. **Supplementary references** [pp. 117–138]
3. **Character list and phylogenetic datasets** [pp. 138–182]

**1.** **Complete list of material assigned to *Funcusvermis gilmorei***

**Holotype.** PEFO 43891, right pseudodentary (Fig. 1, Extended Data Figs. 3–4).

**Paratypes.** PEFO 44432, right pseudodentary; PEFO 45800, right pseudodentary; PEFO 46284, right pseudodentary (Fig. 1, Extended Data Figs. 3–4).

**Paratypes not mentioned in main text.** PEFO 43804, right pseudodentary; PEFO 43889, right pseudodentary; PEFO 44425, right pseudodentary; PEFO 44432, right pseudodentary; PEFO 45643, right pseudodentary; PEFO 45768, right pseudodentary; PEFO 45800, right pseudodentary; PEFO 45982, right pseudodentary; PEFO 46084, right pseudodentary; PEFO 46112, right pseudodentary; PEFO 46138, right pseudodentary; PEFO 46243, right pseudodentary; PEFO 46284, right pseudodentary; PEFO 46477, right pseudodentary; PEFO 46478, right pseudodentary; PEFO 46485, right pseudodentary; PEFO 46486, right pseudodentary; PEFO 46487, right pseudodentary; PEFO 46488, right pseudodentary; PEFO 46489, partial pseudodentary; PEFO 46491, right pseudodentary; PEFO 46493, right pseudodentary; PEFO 46495, right pseudodentary; PEFO 46497, right pseudodentary; PEFO 46498, right pseudodentary; PEFO 46499, partial pseudodentary; PEFO 46636, right pseudodentary; PEFO 46730, right pseudodentary; PEFO 47675, right pseudodentary; PEFO 47676, right pseudodentary; PEFO 47677, right pseudodentary; PEFO 47678, right pseudodentary; PEFO 47679, right pseudodentary; PEFO 47680, right pseudodentary; PEFO 47849, right pseudodentary; PEFO 47850, right pseudodentary; PEFO 47851, right pseudodentary; PEFO 47852, right pseudodentary; PEFO 47853, right pseudodentary; PEFO 47854, left pseudodentary; PEFO 47855, right pseudodentary; PEFO 47856, right pseudodentary; PEFO 47857, right pseudodentary; PEFO 47858, right pseudodentary; PEFO 47859, right pseudodentary; PEFO 47860, right pseudodentary; PEFO 47861, right pseudodentary; PEFO 47862, right pseudodentary; PEFO 47863, right pseudodentary; PEFO 47864, left pseudodentary; PEFO 47869, right pseudodentary; PEFO 47871, right pseudodentary; PEFO 47873, right pseudodentary; PEFO 48872, right pseudodentary; PEFO 49495, right pseudodentary; PEFO 49522, right pseudodentary; PEFO 49535, right pseudodentary; PEFO 49620, right pseudodentary; PEFO 49737, right pseudodentary; PEFO 5023, right pseudodentary; PEFO 50339, right pseudodentary; PEFO 50348, right pseudodentary; PEFO 50403, right pseudodentary; PEFO 50425, right pseudodentary; PEFO 50469, right pseudodentary; PEFO 50543, right pseudodentary; PEFO 50544, right pseudodentary; PEFO 50569, right pseudodentary; PEFO 50572, right pseudodentary; PEFO 50719, right pseudodentary; PEFO 51047, right pseudodentary; PEFO 51187, right psuedodentary; PEFO 51300, right pseudodentary; PEFO 51382, right pseudodentary; PEFO 51465, right pseudodentary; PEFO 51509, right pseudodentary; PEFO 49738, left pseudodentary. All pseudodentaries are partial due to fragmentation.

**Referred material.** PEFO 46481, left maxillopalatine (Fig. 1, Extended Data Fig. 4); PEFO 46479, partial right pseudoangular; PEFO 46480, left pseudoangular (Fig. 1, Extended Data Fig. 3); PEFO 46482, partial right pseudoangular; PEFO 47865, partial left pseudoangular; PEFO 47866, nearly complete left pseudoangular; PEFO 48687, partial pseudoangular; PEFO 49376, partial right pseudoangular; PEFO 51188, partial left pseudoangular; PEFO 51380, partial left pseudoangular; PEFO 45810, postatlantal vertebra (Extended Data Fig. 3), PEFO 4381l, proximal end of right femur (Extended Data Fig. 3).

**Minimum number of individuals.** Pseudodentaries are the most common element assigned to *Funcusvermis*, with a minimum number of 76 individuals (MNI) represented by right pseudodentaries; this abundance is unprecedented in the gymnophionomorph fossil record, where only *Eocaecilia* and *Rubricacaecilia* are known from an MNI greater than one, a MNI of 11 and two, respectively (Supplementary Table 2).

**Pseudodentary size variation.** Dorsoventral height of the pseudodentary at the posterior terminus of the lingual tooth row ranges from 0.92 mm (PEFO 47863) to 1.43 mm (PEFO 44425). This may be a result of ontogeny or some other form of intraspecific variation.

**2. Expanded description of *Funcusvermis gilmorei***

**Differential diagnosis.** A gymnophionomorph diagnosed by a unique combination of features and autapomorphies (listed in the main text). *Funcusvermis gilmorei* is differentiated from closely related taxa based on a unique combination of character states: (** denotes features found in holotype and paratype pseudodentary specimens, all other features are found in referred specimens).

Differentiated from non-lissamphibian amphibamiform dissorophoids in the coossification of postdentary elements forming the pseudoangular, coossification of the maxilla and palatine into a compound maxillopalatine, expansion of the adsymphyseal tooth row to include >20 teeth**, and absence of coronoid**, splenial**, and surangular elements.

Differentiated from adult batrachians and albanerpetontids in the presence of palatal and adsymphyseal** tooth rows in the upper and lower jaws, respectively; further differentiated from batrachians in the presence of heavily pitted dermatocranial elements, a symphyseal foramen** in adults (indicating unossified mentomeckelian cartilage at the mandibular symphysis), and postzygaphophysial interconnection in postatlantal vertebrae. Differentiated from albanerpetontids in the presence of pedicellate dentition**, and presence of a dorsally facing jaw articulation surface of the lower jaw.

Differentiated from *Eocaecilia micropodia* and *Rubricacaecilia* *monbaroni* in the presence of a coossified maxilla and palatine. Differentiated from *E. micropodia*, *R. monbaroni*, and gymnophionans in the absence of osteological correlates for the tentacular organ, absence of continuous vomerine-palatal dentition, absence of the retroarticular and internal processes of the pseudoangular, presence of an enlarged (occupying >30% pseudoangular length) adductor fossa of the pseudoangular, presence of three insertions for cranial nerve V (CN V) in the pseudoangular, and presence of a symphyseal foramen in the pseudodentary**. Differentiated from *R. monbaroni* and gymnophionans in the absence of a transversely aligned deeply inset U-shaped jaw articulation surface of the pseudoangular, and >40 and >20 tooth pedicels in the labial and lingual tooth rows of the pseudodentary**, respectively (excepting *Praslinia cooperi*). Differentiated from gymnophionans in the presence of hind limbs.

**Maxillopalatine***.* PEFO 46481 (Fig. 1, Extended Data Fig. 4) represents a partial left maxillopalatine missing its dorsal, posterior, and anterior surfaces due to breakage during collection. The medial and ventral surfaces are obscured in rock matrix and were digitally segmented from CT data to reveal the underlying morphology (Methods). The element is mediolaterally broad, accommodating the parallel maxillary and palatal tooth rows on its ventral surface. The maxillopalatine in *Funcusvermis gilmorei* is formed by a coossification of the maxilla to the palatine, differing from the condition in *Eocaecilia micropodia* (Jenkins and Walsh, 1993; Jenkins et al., 2007) and *Rubricacaecilia* *monbaroni* (Evans & Sigogneau-Russel, 2001) where these elements are separate (i.e., non-coossified) ossifications. Given the earliest-diverging position of *F. gilmorei* among gymnophionomorphs as recovered in our phylogenetic analyses, coossification of the maxilla and palatine occurred early in gymnophionomorph evolution and is present by the common ancestor of *F. gilmorei* and Gymnophiona. The separate maxilla and palatine ossifications reported in *E. micropodia* and *R. monbaroni* could represent: [1] a homoplastic reversal to the ancestral state exhibited in dissorophoids, suggesting coossification of the maxilla and palatine only became canalized in the common ancestor of Gymnophiona; or [2] coossification of the maxilla and palatine was present in *E. micropodia* and *R. monbaroni*, but taphonomic breakage caused these elements to appear separate. The compound maxillopalatine of *F. gilmorei* is shared with all gymnophionans.

Studies demonstrate the maxillopalatine of living caecilians is formed during development by coossification of the medial side of the maxilla to the lateral side of the palatine (Wake & Hanken, 1982; Reiss, 1996; Müller et al., 2005; Müller 2006; Theska et al., 2019), however another smaller ossification fuses with the maxillopalatine during embryonic development in some taxa, including *Dermophis mexicanus*, *Indiocranium russeli*, *Gegeneophis ramaswamii*, and *Hypogeophis rostratus* where a small ossification coossifies to the maxillopalatine dorsally (Müller et al., 2005; Müller 2006; Theska et al., 2019); this ossification has uncertain homology and may be homologous with the prefrontal or lacrimal. In *I. russeli*, *G. ramaswamii*, and *H. rostratus*, this ossification is pierced by the nasolacrimal duct (= ‘tentacle canal’), allowing the inference of its homology with the lacrimal (Theska et al., 2019). Whether this ossification participates in the maxillopalatine of rhinatrematid gymnophionans (the earliest-diverging clade of living caecilians; Wilkinson and Nussbaum, 1996; Wilkinson, 1997; San Mauro et al., 2014; Wilkinson et al., 2021) is unknown in absence of studies considering their embryonic development; only two ossifications (maxilla and palatine) are present in larvae of *Epicrionops bicolor* and *Epicrionops petersi,* in which these elements coossify during metamorphosis (Reiss, 1996). Whether the lacrimal participates in the maxillopalatine ossification in *Funcusvermis gilmorei* is uncertain; there are no insertions evident on or near the orbital margin where the nasolacrimal canal enters the lacrimal in temnospondyls and in dissorophoids specifically, such as *Doleserpeton annectens* (Bolt, 1977; Sigurdsen & Bolt 2010), *Pasawioops mayi* (Anderson & Bolt, 2013), and *Tersomius dolesensis* (Anderson & Bolt, 2013). The ancestral condition of the lacrimal ossification in batrachians is uncertain; the lacrimal is absent in the earliest diverging salientian *Triadobatrachus massinoti* (Ascarrunz et al., 2016) and variably present in early diverging caudates, where it is absent in *Triassurus sixtelae* (Schoch et al., 2020), but present in *Kokartus honorarius* and *Karaurus sharovi* (Ivachnenko, 1978; Skutschas & Martin 2011). The lacrimal is present in the stem lissamphibian *Gerobatrachus hottoni* (Anderson et al., 2008b) and all other dissorophoids.

The posterodorsal surface of the maxillopalatine is floored by a fossa formed by a mediolaterally-broad, smooth-surfaced shelf, representing the anteroventral margin of the orbit (Fig. 1). Although the complete orbital margin of *Funcusvermis gilmorei* is not present, the preserved portion forms a wide arc, suggesting the complete orbit may have been relatively large, differing from the condition of *Eocaecilia micropodia* (Jenkins et al., 2007) and gymnophionans (Wake, 1985; Wilkinson et al., 2011) where the orbits are reduced or completely absent. Similarly enlarged orbits are a derived feature of dissorophoids (e.g., Bolt, 1969, 1979; Anderson et al., 2008b; Sigurdsen & Bolt, 2010; Schoch, 2019a), and their likely presence in *Funcusvermis* suggests enlarged orbits to be the ancestral gymnophionan condition and a conservation of the orbital form in dissorophoids.

There is no insertion apparent for the nasolacrimal duct on the surface of the orbital margin. However, it is possible this duct entered the orbital margin in the maxillopalatine at a point dorsal to what is preserved in this specimen**,** or in a more dorsally placed element (e.g., lacrimal or prefrontal). In living gymnophionans, the chemosensory tentacle organ exits an aperture or fossa housed by the maxillopalatine anterior to the orbit through a duct coopted from the nasolacrimal duct (Billo & Wake, 1987); this aperture is inset into the anteroventral margin of the orbit in the early-branching rhinatrematids (Nussbaum, 1977). In *Eocaecilia micropodia*, a shallow fossa traverses the dorsal surface of the palatine and maxilla at the anteroventral corner of the orbit, and it is hypothesized to be an osteological correlate for the duct that housed the tentacular organ (Jenkins et al., 2007). There is no sign of an aperture or fossa at a similar location to other gymnophionomorphs in the orbital margin of *Funcusvermis gilmorei*, suggesting that the tentacular organ was not present in *F. gilmorei* (Fig. 1 Extended Data Fig. 4). We hypothesize that osteological expression of the tentacular organ and therefore the tentacular organ itself was gained after the divergence of *F. gilmorei* in the common ancestor of *E. micropodia* and gymnophionans.

A mediolaterally thin lamina of bone extends dorsally anterior to the orbit in *Funcusvermis gilmorei*, forming a tall facial ramus (Fig. 1, Extended Data Fig. 4). The facial ramus curves dorsomedially towards the roof of the skull, overhanging medially beyond the lateral margin of the palatal tooth row. The tall facial ramus of the maxillopalatine in *F. gilmorei* differs from the condition in dissorophoids (Schoch, 2019a) and *Eocaecilia micropodia* (Jenkins et al., 2007), in which the maxilla is dorsoventrally low anterior to the orbit. It is more like the condition in gymnophionans where the facial process is dorsally tall and curved medially towards the skull roof. A tall facial ramus of the maxilla is present in early-diverging urodelans (Schoch et al., 2020) but absent in early diverging salientians such as *Prosalirus bitis* (Shubin & Jenkins, 1995). It is also absent in the putative stem-batrachian *Gerobatrachus* *hottoni* (Anderson et al., 2008b) but present in the highly nested amphibamiform *Doleserpeton* *annectens* (Sigurdsen & Bolt, 2010), which was long considered the closest relative of lissamphibians prior to the discovery of *G. hottoni* (e.g., Bolt, 1969).

The maxillary and palatal tooth rows bear 17 and 11 tooth pedicels, respectively, and these two rows bear tooth pedicels of similar size and shape (Fig. 1). Due to anterior and posterior breakage, the extent of the maxillary tooth row and total number of teeth in both maxillary and palatal tooth rows is unknown; however, the palatal tooth row appears to naturally terminate prior to the anterior end of the element. Immediately medial to the palatal tooth row is a smooth laterally concave margin of bone representing the lateral choanal margin. The form of the choanal margin and palatal portion of the maxillopalatine suggests that the palatine ossification of *Funcusvermis gilmorei* was a thin rod-like strut, likely a conservation of the derived condition in amphibamiform dissorophoids (Schoch, 2019a). The palatal tooth row terminates prior to the anterior end of the maxillopalatine in *F. gilmorei* such that the palatal and vomerine tooth rows would not have formed a continuous tooth row lateral to the choana, unlike the condition in *Eocaecilia* *micropodia* and other gymnophionomorphs where the palatal tooth row continues anteriorly meeting the tooth row on the vomer. Instead, here the anteriorly foreshortened palatal tooth row resembles the condition seen in amphibamids like *Doleserpeton annectens* (e.g., Sigurdsen & Bolt, 2010). In amphibamids, a short row of five to seven pedicellate teeth of similar size to the marginal teeth is present on the palatine, and this tooth row is oriented parallel to the marginal row and is separated from the tooth row of the vomer by the choana (Bolt, 1974b, 1977; Sigurdsen & Bolt, 2010; Schoch, 2019a). We hypothesize that the palatal tooth row of *F. gilmorei* represents a transitional state of the gymnophionomorph palate between the condition of *D. annectens* and gymnophionans; the maxilla and palatine are coossified as in gymnophionans, but as in *D. annectens* the palatal tooth row is anteriorly terminal and separated from the vomerine tooth row by the lateral choanal margin.

In the maxillopalatine of *Funcusvermis gilmorei*, the maxillary and palatal tooth rows are parallel to each other and separated by a deep groove (Fig. 1). This condition resembles the pattern in *Eocaecilia micropodia* where maxillary and palatine tooth rows form two closely spaced parallel rows of tooth pedicels. The close mediolateral spacing and parallel arrangement of the tooth rows in *E. micropodia* and *F. gilmorei* differ from the condition in living caecilians, where space between the palatal and maxillary dentitions increases anteriorly (e.g., Wilkinson et al., 2011).

**Mandible***.* The mandible of *Funcusvermis gilmorei* is composed of two compound bones, the pseudoangular and pseudodentary, which were articulated in life along an extensive overlapping sutural surface present on the medial side of the pseudodentary and lateral side of the pseudoangular (Fig. 1, Extended Data Fig. 3). This bipartite composition of the mandible in *F. gilmorei* is a derived condition shared with all other gymnophionomorphs (*Eocaecilia micropodia*, *Rubricacaecilia monbaroni*, and gymnophionans). The identity of lower jaw ossification centers in living gymnophionans is best known from developmental studies of embryonic stages of *Dermophis mexicanus* (Wake & Hanken, 1982), *Geneophis ramaswamii* (Müller et al., 2005), *Hypogeophis rostratus* (Müller, 2006), and *Indiocranium russeli* (Theska et al., 2019). The pseudodentary is formed by coossification of the mentomeckelian, dentary, and tooth-bearing ‘coronoid’ (a likely homologue of the adsymphyseal, see discussion below) in *G. ramaswamii* (Müller et al., 2005)*, H. rostratus* (Müller, 2006), and *I. russeli* (Theska et al., 2019); however, two additional ossifications of uncertain homology participate in the pseudodentary of *D. mexicanus*: the ‘coronoid’ and ‘complementare’ (Wake & Hanken, 1982; Theska et al., 2019). The pseudoangular is composed of the angular and articular in *H. rostratus* (Müller, 2006), *D. mexicanus* (Wake & Hanken, 1982), and *I. russeli* (Theska et al., 2019); however, the prearticular is additionally present in *G. ramaswamii* (Müller et al., 2005). Lack of developmental series prohibits conclusive assertions of homology of the elements comprising the mandibular ramus in *F. gilmorei*; however, distinctive differences in its mandibular anatomy compared to that of dissorophoids, non-gymnophionomorph lissamphibians, and other gymnophionomorphs allows for novel inferences of homology made in the following description.

**Pseudodentary.** The pseudodentary forms the anterior tooth-bearing portion of the mandible in *Funcusvermis* and all other gymnophionomorphs. In life, it articulated medially along an oblique suture to the lateral side of the pseudoangular. The pseudodentary is the most common element referred to *Funcusvermis gilmorei* recovered from PFV 456, represented by at least 61 partial to nearly complete specimens (including both the right and left sides), although no single specimen preserves the entire element from its anterior to posterior ends. Although there is some variation in the size of pseudodentary specimens, they show little variation in overall morphology. PEFO 43891 and PEFO 46284 are the best preserved anterior and posterior portions of the element, and they preserve overlapping portions of the pseudodentary allowing for composite reconstruction of the entire element (Fig. 1; Methods). The following description is based on those two specimens, as well as PEFO 45800 for its preservation of the dentition (Fig. 1). The overall form of the pseudodentary is rod-like, curving medially towards the mandibular symphysis, resembling the condition of the dentary bone in dissorophoids, batrachians, albanerpetontids, and other gymnophionomorphs.

The dentary (=labial) tooth row extends distally from the mandibular symphysis nearly the entire length of the element, terminating where the dorsal facet for attachment of the pseudoangular overlaps the dorsal crest of the pseudodentary (Fig. 1, Extended Data Fig. 3). No single specimen preserves the entire dentary tooth row, prohibiting a full count of tooth pedicels, but the tooth number in two nearly complete specimens (PEFO 46284: 54; PEFO 45800: 52) is evidence that this row contained at least 52 teeth. The number of teeth in the dentary tooth row of *Funcusvermis gilmorei* is slightly higher than that of *Eocaecilia* (40–43 teeth; Jenkins et al., 2007), and slightly less than in amphibamiform dissorophoids like *Doleserpeton annectens* (>60 teeth; Sigurdsen & Bolt, 2010). The tooth number in the dentary tooth row of *F. gilmorei* and *E. micropodia* is greater than in *Rubricacaecilia monbaroni* (28 teeth), and about twice that of nearly all gymnophionans excepting *Praslinia cooperi*. (Boulenger 1909; Taylor, 1977; Jenkins et al., 2007). This suggests that the high tooth count in the dentary tooth row of *F. gilmorei* and *E. micropodia* represents a slight reduction from the dissorophoid condition, and a more major reduction in tooth count occurred in the common ancestor of *R. monbaroni* and Gymnophiona.

The adsymphyseal (=lingual) tooth row extends from the mandibular symphysis to a point slightly anterior to the anterodorsal terminus of the tapering pseudoangular facet of the pseudodentary (Fig. 1, Extended Data Fig. 3). Several specimens preserve the complete adsymphyseal tooth row, showing it to include at least 22 tooth pedicels (PEFO43891: 22; PEFO 45800: 23). An adsymphyseal row of pedicellate teeth arranged parallel to the labial tooth row at the mandibular symphysis is shared with *Doleserpeton annectens*, *Eocaecilia micropodia*, *Rubricacaecilia monbaroni*, and gymnophionans (excepting the derived loss of the lingual tooth row in some gymnophionan taxa; e.g., Nussbaum, 1985; Wilkinson et al., 2011), however the number of teeth in this row varies between these taxa (*D. annectens*: 5–7; Sigurdsen & Bolt, 2010); *E. micropodia*: >20; *R. monbaroni* and Gymnophiona (excepting *Praslinia cooperi*; see discussion in Jenkins et al., 2007:p. 330): <10 (Taylor, 1977; Evans & Sigogneau-Russel, 2001; Jenkins et al., 2007). We hypothesize that the number of adsymphyseal teeth in *F. gilmorei* represents a transformation of the condition exhibited in *D. annectens* through addition of new teeth distally, yielding a tooth count of at least 20 in the common ancestor of *F. gilmorei* and *E. micropodia*. A reduction of tooth number to less than 10 occurred in the common ancestor of *R. monbaroni* and Gymnophiona.

A deep subdental groove located medial to the dentary tooth row extends from the posterior tooth row terminus to the mandibular symphysis of *Funcusvermis gilmorei*, separating the dentary and adsymphyseal tooth rows at the anterior of the element where both are present. Another subdental groove is present medial to the adsymphyseal tooth row extending from the mandibular symphysis to the posterior terminus of the adsymphyseal tooth row. Similar subdental grooves are found in the dentary of *Doleserpeton annectens* and the pseudodentary of gymnophionomorphs.

The medially facing surface of the pseudodentary for attachment to the lateral side of the pseudoangular is best preserved in PEFO 46284; this wedge-shaped facet tapers anteriorly to its terminus approximately halfway along the longitudinal length of the pseudodentary (Fig. 1, Extended Data Fig. 3). Inset into this surface are two longitudinally oriented facets that fit with matching facets on the lateral surface of the pseudoangular (Extended Data Fig. 3); the ventral facet is dorsoventrally thin, whereas the dorsal facet expands in dorsoventral height in the posterior direction. At its posterior end, the dorsal facet overhangs a longitudinal fossa confluent with an anteriorly directed foramen for insertion of the alveolar branch of CN V and associated vasculature, which continues anteriorly towards the mandibular symphysis below to the labial tooth row. Ventral to the aforementioned fossa is a medially concave, longitudinally directed fossa that housed the external branch of CN V, associated vasculature, and presumably a cartilaginous Meckel’s cartilage, and this fossa becomes fully enclosed by the pseudodentary at the anterior terminus of the pseudoangular attachment surface. This canal continues anteriorly through the ramus, where two branches exit the ventral surface through small foramina posterior to the symphysis (Extended Data Figs. 3–4). Similar exits for branches of this neurovasculature are present in *Eocaecilia micropodia*, *Rubricacaecilia monbaroni*, and most gymnophionans (Evans & Sigogneau-Russel, 2001; Jenkins et al., 2007). This canal opens at the mandibular symphysis through a conspicuous symphyseal foramen (Fig. 1, Extended Data Fig. 3); the presence of an open foramen in this position suggests the mentomeckelian cartilage was unossified at the symphysis in *Funcusvermis gilmorei*. The mandibular symphysis is closed in other fossil gymnophionomorphs (Jenkins et al., 2007; Evans & Sigogneau-Russel, 2001), suggesting the presence of an ossified mentomeckelian closing the mandibular symphysis as in living caecilians (e.g., Theska et al., 2019). The mentomeckelian is present as an ossified element at the symphysis (separate from the dentary) in batrachians (e.g., Carroll, 2007). The symphyseal foramen in *F. gilmorei* resembles a foramen of similar form and position found in the dentary of all albanerpetontids (Gardner & Averianov, 1998:p. 459) that represents the anterior opening of the Meckelian canal (Sweetman & Gardner, 2012:p. 310). Ossification of the Meckel’s cartilage at the mandibular symphysis forming the mentomeckelian (as in lissamphibians) is hypothesized to be absent in non-lissamphibian temnospondyls (Bolt, 1991; Carroll, 2007), and the presence of an open Meckelian canal at the mandibular symphysis of dissorophoids including *Eoscopus lockardi* (Daly, 1994), *Tersomius texensis* (Carroll, 1964), and *Doleserpeton* *annectens* (Bolt, 1991) suggests conservation of this condition in albanerpetontids and *F. gilmorei* (and therefore possibly in the common ancestor of Lissamphibia). Accordingly, this suggests independent acquisitions of the mentomeckelian ossification in batrachians and the common ancestor of *E. micropodia* and gymnophionans (Fig. 3).

A fossa confluent with the symphyseal foramen bisects the symphyseal surface between the anterior termini of the dentary and adsymphyseal tooth rows at the mandibular symphysis, forming medial and lateral processes (Fig. 1, Extended Data Fig. 3). The presence of a symphyseal foramen and confluent fossa bisecting the anteroposteriorly flared mandibular symphysis in *Funcusvermis* *gilmorei* differs from the mandibular symphysis of *Eocaecilia micropodia*, *Rubricacaecilia monbaroni*, and gymnophionans, in which the mandibular symphysis is fully closed and exhibits no anterior expansion or processes. The medial and lateral processes at the mandibular symphysis of *F. gilmorei* resembles the condition of *Doleserpeton annectens* (Sigurdsen & Bolt, 2010) and the symphyseal prongs of albanerpetontids (e.g., Gardner & Averianov, 1998; Gardner, 2000, 2001; Gardner et al., 2003; Matsumoto & Evans, 2018; Daza, 2020), although they are less pronounced in *F. gilmorei* and *D. annectens*, opening the possibility that this feature is present in the common ancestor of Lissamphibia, conserved in early gymnophionomorphs, and lost in batrachians and later gymnophionomorphs.

*Homology of the gymnophionomorph lingual tooth row.* The homology of the gymnophionomorph lingual tooth row has remained uncertain due to the conflicting topologies of gymnophionomorph relationships to other tetrapod groups, as well as uncertainty in the homology of ossifications forming the pseudodentary in living caecilians. Developmental studies of the gymnophionan mandible indicate the presence of a separate tooth bearing *anlage* medial to the anterior end of the dentary *anlage* in embryonic stages; later in development this bone coossifies to the medial surface of the dentary at the mandibular symphysis, forming the lingual row of dentition (Müller et al., 2005; Müller, 2006; Theska et al., 2019). More specifically, it has been assumed that this is coronoid 1 (the anteriormost coronoid in temnospondyls) based on the position of this element (see discussion by Müller et al., 2005; Pardo et al., 2017a). If this interpretation is correct, this would place a premium on identifying fossil tetrapods with a single row of teeth on coronoid 1, as in *Chinlestegophis jenkinsi* (but also in a number of other unrelated temnospondyls). It would also offer a line of evidence against a monophyletic origin of caecilians within amphibamiform dissorophoids, as all dissorophoids retain the plesiomorphic shagreen of denticles on the coronoid series.

An alternative consideration for the homologue of the element bearing the gymnophionomorph lingual tooth row is another element, sometimes considered part of the coronoid series but not bearing the coronoid name: the adsymphyseal (sometimes termed the ‘parasymphyseal’ or the ‘parasymphyseal plate’). The presence of an adsymphyseal bearing an organized row of teeth is derived in stem tetrapods (e.g., Ahlberg & Clack, 1998; Bolt & Lombard, 2001). It occurs at and participates in the mandibular symphysis, separating coronoid 1 from this region, and usually bears dentition in some form, ranging from a patch of denticles to a continuous tooth row with enlarged ‘fangs.’ It is classified as part of the coronoid series by some workers based on its position and similar dentition to the coronoids proper (e.g., Jessen, 1965; Shishkin, 1967; 1973; Bolt & Lombard, 2001, 2006). The presence of the adsymphyseal as an ossification separate from the dentary is exceedingly rare in crown tetrapods and was historically used as a feature to exclude temnospondyls from consideration in the identification of isolated lower jaws in late Carboniferous-early Permian deposits (e.g., Milner & Lindsay, 1998). However, an adsymphyseal has been identified in at least two dissorophids (Anderson, 2005; Reisz et al., 2009; Anderson et al., 2020) and possibly in an amphibamiform (Anderson et al., 2008a), a plagiosaurid (Damiani et al., 2009), and a trematosaur (Novikov, 2012a); this is a wide taxon bracket encompassing the majority of Rhachitomi. Furthermore, Shishkin and Sulej (2009) reported the presence of an adsymphyseal incompletely coossified to the dentary in juvenile specimens of the capitosaurid *Parotosuchus speleus*, suggesting its homology to the adsymphyseal in stem tetrapods and indicating that the adsymphyseal tooth row of temnospondyls may coossify with the dentary later in their development (p. 69 therein). The apparent paucity of the adsymphyseal as a distinct ossification among temnospondyls may be interpreted as evidence that this bone could not be the homologue of the lingual tooth-bearing element in gymnophionomorphs. However, it must be considered whether the element has simply been misidentified due to preservation, tight sutural articulation, or coossification with the dentary, and that the long-held perception that this element was absent across Temnospondyli was mistaken (having only been identified in this clade by Anderson, 2005).

Plesiomorphically in dissorophoids, three denticulate coronoid bones articulate with the medial surface of the dentary, positioned ventral to the dentition and dorsal to the Meckelian canal (Extended Data Fig. 8). The anteriormost coronoid (coronoid 1) has historically been interpreted to lie at the mandibular symphysis in the absence of an adsymphyseal, followed posteriorly by coronoids 2 and 3 (e.g., Carroll, 1964; Schoch & Rubidge, 2005; Clack & Milner, 2010). However, it is important to note that most historic characterizations are reconstructions that cannot identify the actual sutures. Often, in the absence of discernible sutures, temnospondyl workers assume that the dentigerous surface lingual to the dentary belongs to the coronoids proper (e.g., Clack & Milner, 2010) and make no mention of an adsymphyseal. The dorsal and lingual exposures of most amphibamiform lower jaws are in fact unknown, either due to a paucity of specimens or to articulation with the skull; for this reason, characterizations of the lower jaw of most branchiosaurids and non-branchiosaurid amphibamiforms are generic; some phylogenetic analyses do not even include any lower jaw characters (e.g., Schoch & Milner, 2008). Therefore, we present the possibility here that (1) an adsymphyseal ossification is much more widely present among temnospondyls; and (2) that its ossification and subsequent coossification with the dentary may have produced the lingual tooth row of gymnophionomorphs. Neontologists may not have considered the possibility that this ossification, rather than coronoids 1, 2, or 3 (or some combination of the three), forms the lingual tooth row in gymnophionomorphs because of its perceived rarity among crown tetrapods. Main Text Fig. 3 illustrates the phylogenetic distribution and comparative morphology of the adsymphyseal and the adsymphyseal tooth row in the colosteid stem-tetrapod *Greererpeton burkemorani*, the dissorophoid *Cacops aspidephorus*, the amphibamiform *Doleserpeton annectens*, and a selection of extinct and extant lissamphibians including *Funcusvermis gilmorei*. Extended Data Fig. 8 illustrates the phylogenetic distribution and comparative morphology of a single row of coronoid teeth in Temnospondyli.

*Patterns of homology in the lissamphibian mandibular ramus.* Dissorophoids (e.g., *Acheloma cumminsi*, *Cacops aspidephorus*; Gee et al., 2019; Anderson et al., 2020), including amphibamiforms (e.g., *Tersomius texensis*; Carroll, 1964), retain the plesiomorphic condition of temnospondyls (Jupp & Warren, 1986) in which the mandible is composed of at least nine dermal bones including the dentary, three coronoids, splenial, postsplenial, prearticular, angular, prearticular, articular, and surangular. As previously discussed, the dissorophoid dentary likely represents a compound bone formed by the dentary and adsymphyseal; additionally, the mentomeckelian is absent in this group.

The revised homology of the adsymphyseal tooth row of gymnophionomorphs and lack of the mentomeckelian ossification in *Funcusvermis gilmorei* refines understandings of homology in the evolution of the lissamphibian mandibular ramus. The mentomeckelian in batrachians and *Eocaecilia micropodia* + Gymnophiona appears to be independently derived and was therefore likely absent in the common ancestor of Lissamphibia. Besides the adsymphyseal, other bones in the coronoid series appear to be lost in gymnophionomorphs, salientians, and albanerpetontids. However, a single tooth bearing coronoid (likely homologous to the posterior coronoid (=coronoid 3) of dissorophoids) is present medial to the posterior end of the dentary tooth row in some early-branching caudates (e.g., Gao & Shubin, 2001; Jia & Gao, 2016a, b), and is variably present in living caudates where it typically resorbs during metamorphosis causing its absence in adults (e.g., Jia & Gao, 2019). Therefore, evolutionary patterns regarding the coronoid bones in lissamphibians include the loss of at least the anterior two coronoids (not including the adsymphyseal) in the common ancestor of Lissamphibia, loss of all coronoids (including the adsymphyseal) in salientians and albanerpetontids, loss of coronoids (not including the adsymphyseal) in gymnophionomorphs, and retention of a posterior coronoid in caudates. The surangular, present in dissorophoids (e.g., *Doleserpeton annectens*, Sigurdsen & Bolt, 2010), is absent in all lissamphibians, suggesting its loss is a shared derived feature of the clade (Trueb & Cloutier, 1991). Generally, these inferences demonstrate a shared pattern of mandibular simplification in the common ancestor of Lissamphibia driven by loss of mandibular ossifications present in dissorophoids, and subsequent patterns in the four major lissamphibian lineages of further loss and fusion of ossifications. Improved understandings of the relationship of albanerpetontids to batrachians and gymnophionomorphs may further elucidate the stepwise acquisition lissamphibian mandibular features, however the current uncertain placement of albanerpetontids largely obscures whether features of the albanerpetontid mandible represent lissamphibian plesiomorphies, lissamphibian synapomorphies, albanerpetontid autapomorphies, or homologies with either batrachians or gymnophionomorphs.

**Pseudoangular.** The pseudoangular of *Funcusvermis gilmorei* is represented by four specimens (PEFO 46480, PEFO 46482, PEFO 47866, PEFO 47865) that share similar morphological features and vary only in size. PEFO 46480 is a left pseudoangular that is complete except for its anteriormost end (Fig. 1, Extended Data Fig. 3); it represents the most complete pseudoangular recovered from PFV 456, and therefore it is the basis for this description.

The jaw articulation surface is positioned near the posterior terminus of the element, and is formed by a subcircular, dorsomedially facing, slightly convex pad of bone (Fig. 1, Extended Data Fig. 3). The slightly porous surface of the jaw articulation surface gives it a rugose texture, distinct from the smooth external bone present on the rest of the element. The location of the jaw articulation surface in *Funcusvermis gilmorei* (at the posterior terminus of the pseudoangular), differs from the condition in all other gymnophionomorphs, in which it is located midway along the element anterior to the retroarticular process and lateral to the internal process. In *F. gilmorei* the jaw articulation surface is more similar in both morphology and position to the condition in dissorophoids and batrachians, where the jaw articulation surface is formed by a planar, dorsally facing subcircular pad occupying the posterior terminus of the lower jaw. The form of the jaw articulation surface in *F. gilmorei* is especially like that of the amphibamiform dissorophoid *Doleserpeton annectens* (FMNH UR1335; Sigurdsen & Bolt, 2010, fig. 3C), suggesting they may also share similarities in their quadrate morphology. The jaw articulation surface morphology of *Eocaecilia micropodia* is similar to that of *F. gilmorei*, differing from the transversely oriented deeply inset U-shaped fossa forming the jaw articulation surface in *Rubricacaecilia monbaroni* and all gymnophionans (e.g. Nussbaum 1983; Bemis et al., 1983; Evans & Sigogneau-Russel, 2001; Jenkins et al., 2007; Wilkinson et al., 2011)., where the condyle of the quadrate-squamosal complex fits tightly into the fossa of the jaw joint, increasing the stability of the jaw closure mechanism (Bemis et al., 1983; Jenkins et al., 2007). The morphology of the jaw articulation surface morphology in *F. gilmorei* and *E. micropodia* suggests jaw closure may have been destabilized relative to living gymnophionans, allowing propalinal movement (Jenkins et al., 2007) as in batrachians and presumably dissorophoids.

A small subtriangular process projects (=posterior pseudoangular process; Fig. 1, Extended Data Fig. 3) posteriorly from the posterolateral margin of the jaw articulation surface, forming the posteriormost extent of the lower jaw. The articular of *Doleserpeton annectens* bears a small posterolaterally projecting process situated on the posterolateral corner of the jaw articulation surface, strongly resembling the posterior pseudoangular process in *Funcusvermis gilmorei*. This resemblance suggests this process may be homologous in *F. gilmorei* and *D. annectens*. In *F. gilmorei*, this process may represent a posterior expansion of the pseudoangular that later becomes the elongate retroarticular process in the common ancestor of *Eocaecilia micropodia* and Gymnophiona. Unlike all other gymnophionomorphs, and similar to dissorophoids, batrachians, and albanerpetontids, the elongate retroarticular process is absent in *F. gilmorei*, suggesting the ancestral gymnophionomorph condition lacks this feature.

The adductor fossa is formed by a deep fossa that occupies most of the dorsal surface of the pseudoangular anterior to the jaw articulation surface, occupying > 30% of the total pseudoangular length (Supplementary Table 1). Ancestrally in tetrapods the *m. adductor mandibulae* complex (mAM) inserts into the adductor fossa and acts as the main jaw closure musculature (e.g., Carroll & Holmes, 1980; Kleinteich & Haas, 2007). This condition is present ancestrally in batrachians, albanerpetontids, and dissorophoids, which all bear a correspondingly large, dorsally opening adductor fossa for insertion of the mAM. *Funcusvermis gilmorei* bears similar adductor fossa morphology to these groups, suggesting it conserved the ancestral tetrapod jaw closure mechanism driven primarily by the mAM (Nussbaum, 1983; Kleinteich & Haas, 2007; Johnston, 2011). *Eocaecilia micropodia*, *Rubricacaecilia monbaroni*, and gymnophionans exhibit a significantly reduced adductor fossa relative to *F. gilmorei*. The small adductor fossa (< 20% pseudoangular length; Supplementary Table 1) in living caecilians is associated with the derived caecilian-type ‘dual-jaw closure mechanism’ (sensu Nussbaum, 1983), which is driven by two muscular systems, including: [1] the *m. adductor mandibulae* (mAM) complex, which extends from the temporal region of the cranium to its insertion in the mandibular fossa and dorsal surface of the pseudoangular just anterior to the jaw joint, acting as a third-order lever pulling up on the lower jaw (Nussbaum, 1983; Bemis et al., 1983; Kleinteich et al., 2008); and, [2] a derived component of the hyobranchial muscle *m. interhyoidus posterior* (mIHP), which inserts on the ventral side of the retroarticular process and extends posteroventrally, acting as a first-order lever pulling posteroventrally, causing the anterior component of the lower jaw to pivot upwards with respect to the quadrate (Nussbaum, 1983; Bemis et al., 1983; Summers & Wake, 2005; Kleinteich et al., 2008). In the caecilian type dual jaw closure mechanism, the mIHP acts as the primary jaw closure musculature, while the reduced mAM plays a minor role. This derived muscle arrangement is hypothesized to be an adaptation for fossoriality (Nussbaum, 1983); in this scenario, compaction of the temporal region and enlargement of the squamosal limited and reduced the size of the mAM, necessitating use of the mIHP as an additional jaw-closing muscle to compensate for loss of bite force through reduction of the mAM (Nussbaum, 1983). This dual-jaw closure apparatus is present in all living gymnophionans, but its condition in rhinatrematids differs from that of all other gymnophionans. In rhinatrematids the adductor fossa is both unroofed and larger than in all other gymnophionans (Wake, 1993), and the mAM is larger while the mIHP is smaller than in other gymnophionans, allowing the mAM muscle to contribute more to bite force (Nussbaum, 1983). The open temporal fossae of zygokrotaphic rhinatrematids accommodates the cranial attachment of the mAM, differing from the condition of all other gymnophionans (including both zygokrotaphic and stegokrotaphic forms) where the reduced mAM attaches to bones on the ventral surface of the closed skull roof (Nussbaum, 1983). Given the early-branching position of rhinatrematids (sister group to all other gymnophionans), their jaw-closing mechanism has been hypothesized to represent the ancestral condition for gymnophionans (Nussbaum, 1983): in this scenario the less-reduced mAM of rhinatrematids is a conservation of the ancestral tetrapod condition, and the common ancestor of non-rhinatrematid gymnophionans (stegokrotaphians) acquired a derived condition where the mIHP is enlarged and is the dominant jaw-closing muscle, while the mAM is reduced (and its attachment to the skull roof accommodated by the temporal fenestra is lost). Nussbaum (1983) predicted that the ancestral mAM in ‘protocaecilians’ would have been even larger than in rhinatrematids and would likely have been accommodated by open temporal fossae. The adductor fossa of *F. gilmorei* is significantly larger than that of rhinatrematids and *E. micropodia* (Supplementary Table 1)*,* seemingly confirming this prediction. Furthermore, this suggests mIHP component of the gymnophionan jaw closure mechanism was absent in the common ancestor of Lissamphibia, confirming hypothesis A of Kleinteich & Haas (2007:fig. 9). Although the skull roof of *F. gilmorei* is unknown, it likely possessed larger accommodation space for attachment of the mAM than in all other gymnophionomorphs, suggesting it may have either conserved the skull roofing condition of dissorophoids, or acquired open temporal fossae homologous with those in zygokrotaphic rhinatrematids. The zygokrotaphic skull roof of *E. micropodia*, which retains many dermal bones of the temporal region present ancestrally in dissorophoids like *Doleserpeton annectens* (and absent in gymnophionans), may represent: [1] a modified form of the closed skull roof of dissorophoids (suggesting *F. gilmorei* may have shared this morphology); or, [2] an autapomorphic reversal to stegokrotaphy (if *F. gilmorei* had temporal fossae homologous to those of rhinatrematids) (Carroll & Homes, 1980; Maddin et al., 2012).

The medial margin of the adductor fossa is framed by a dorsally-oriented lamina bearing two dorsally directed processes, including: [1] the preglenoid process (preglenoid positioned just anterior to the jaw articulation surface); and, [2] the hamate process positioned anterior to a low saddle separating it from the preglenoid process (Fig. 1, Extended Data Fig. 3). An internal process similar to that of all gymnophionans is absent in *Funcusvermis gilmorei*, however it is possible either the preglenoid ridge or hamate process is homologous to the internal process, requiring medial elongation of one of these processes in the common ancestor of *Eocaecilia micropodia* and Gymnophiona. Anterior to the hamate process the medial margin of the adductor fossa sweeps laterally forming a low ridge that frames the anteromedial margin of the adductor fossa. This ridge meets the coronoid process on the lateral margin of the pseudoangular, causing the anteriorly tapering adductor fossa to terminate acutely at this junction. The coronoid process is anteroposteriorly elongate, rising in dorsal height in the anterior direction from the adductor fossa to its dorsal apex near the anterior terminus of the adductor fossa, forming the lateral margin of the adductor fossa. The coronoid process of *F. gilmorei* is similar to the elongate coronoid process derived in dissorophoids, where it is formed by coronoid 3 (e.g., Schoch & Rubidge, 2005), suggesting *F. gilmorei* conserves this dissorophoid condition. A dorsally expanded eminence is present at the same location as the coronoid process of *F. gilmorei* in *E. micropodia*, *Rubricacaecilia monbaroni*, and gymnophionans, indicating conservation of this morphology in all gymnophionomorphs.

Three foramina pierce the adductor fossa in *Funcusvermis gilmorei*, representing insertions for branches of neurovasculature associated with the mandibular branch of CN V, including (Fig. 1, Extended Data Fig. 3): [1] A small dorsally placed foramen located at the posteriormost end of the adductor fossa; [2] a large foramen located on the floor of the adductor fossa; and [3] a small foramen located on the raised surface of the adductor fossa anterior between the coronoid and hamate processes. A foramen is present on the ventral surface of the posterior end of the pseudoangular opposite the jaw articulation surface, likely the insertion point of neurovasculature associated with CN VII. The intramandibular foramen forms a large anteroposteriorly oblong foramen on the medial surface of the element, serving as the exit for neurovasculature associated with ramus intramandibularis of CN V. The neurovascular arrangement in the pseudoangular of *F. gilmorei* is largely similar to that of *Eocaecilia micropodia*, *Rubricacaecilia monbaroni*, and gymnophionans, including the following: insertion of the mandibular branches of CN V into the lower jaw via a foramen in the floor of the adductor fossa; a large intramandibular foramen on the medial surface of the pseudoangular for exit of the intramandibular branch of CN V; insertion of the alveolar branch of CN VII on the ventral or medial surface of the pseudoangular ventral to the jaw articulation surface. The presence of more than three foramina for insertion of the mandibular branch of CN V in the floor of the adductor fossa differs from the condition in all other gymnophionomorphs where a single insertion is present.

In *Funcusvermis gilmorei*, the lateral articulation surface of the pseudoangular for attachment to the medial side of the pseudodentary is formed by a vertically oriented and laterally facing inset fossa occupying the lateral side of the pseudoangular from its anterior end to a position ventral to the anterior end of the jaw articulation surface (Extended Data Fig. 3). Two longitudinally oriented facets are present on the dorsal and ventral margins of the pseudodentary articulation surface of the pseudoangular, and these facets match partner facets on the medial surface of the pseudodentary (Extended Data Fig. 3). An anteriorly directed foramen emerges midway along the pseudodentary attachment surface of the pseudoangular, representing the medial exit of neurovasculature associated with CN V, confluent with a longitudinally oriented fossa that extends through the anterior end of the element. This fossa forms a closed canal when the pseudodentary and pseudoangular are in articulation, carrying CN V neurovasculature anteriorly along the mandibular ramus into the pseudodentary (Extended Data Fig. 3). The anteriorly tapering dorsal and ventral margins of the anterior third of the pseudodentary attachment surface of the pseudoangular forms a wedge that fits into the matching anteriorly tapering fossa on the medial surface of the pseudodentary (Extended Data Fig. 3). The pseudoangular-pseudodentary attachment morphology of *F. gilmorei* is shared with all other gymnophionomorphs, indicating this morphology was present ancestrally in Gymnophionomorpha.

**Pedicellate dentition.** The maxillopalatine and pseudodentary are the only dentigerous elements known from *Funcusvermis gilmorei* (Fig. 1, Extended Data Figs 3–4). The basal tooth structure is consistent with the pedicellate dentition seen in other gymnophionomorphs and many lissamphibians, where each tooth is bipartite and consists of a basal dentine cylinder and apical crown separated by an unmineralized dividing zone of fibrous tissue (Parker & Dunn, 1956; Parsons and Williams, 1962, 1963; Lawson, 1965a,b; Davit‐Béal et al., 2007; Chuliver & Scanferla, 2019). The absence of tooth crowns in *F. gilmorei* is due to separation of the crown from the pedicel along the weak unmineralized dividing zone prior to fossilization, and the uniformity of the dividing zone in each pedicel apex suggests this dividing zone was well-developed in *F. gilmorei* (e.g., Parsons and Williams, 1962; Lawson, 1965; Davit‐Béal et al., 2007; Chuliver & Scanferla, 2019). Isolated tooth crowns consistent in morphology to *Eocaecilia micropodia* have not been found from several hundred kilograms of even the finest fractions of washed and picked concentrate from PFV 456.

Each pedicel of *Funcusvermis gilmorei* forms a hollow cylinder attached to tissue of the dentary on its labial side, and free on its lingual side (Fig. 1, Extended Data Fig. 4). Many pedicels are open lingually at their bases, serving as an opening for neurovasculature to innervate the pulp cavity (Casey & Lawson, 1981). Struts of attachment tissue frame the basal pore and attach the bases of the mesial and distal pedicel walls to the underlying dentary bone (Lawson, 1954a, b; Casey & Lawson, 1981; Chuliver & Scanferla, 2019). The basal pore varies in size, suggesting stages in the process of pedicel resorption as the basal pore expands, eventually resorbing the entire lingual wall and leaving only the labial wall intact (Lawson, 1965b; Casey & Lawson, 1981). Resorbed tooth loci that retain only the crescent-shaped lingual pedicel wall can be observed in most pseudodentaries of *Funcusvermis gilmorei* (Fig. 1). Tooth pedicels are of approximately uniform diameter in both labial and lingual tooth rows of each pseudodentary. Pedicles among pseudodentary specimens range in labiolingual diameter at the pedicel apex from a minimum of 0.07 mm (PEFO 47859) to a maximum of 0.09 mm (PEFO 45800). The morphology of the tooth pedicels in *F. gilmorei* closely resembles that of *Doleserpeton annectens* (Sigurdsen and Bolt, 2010), *Eocaecilia micropodia* (Jenkins et al., 2007), *Rubricacaecilia monbaroni* (Evans & Sigogneau-Russel, 2001), and gymnophionans (e.g., Parker & Dunn, 1956; Parsons and Williams, 1962, 1963; Lawson, 1965a,b; Davit‐Béal et al., 2007; Chuliver & Scanferla, 2019), which all exhibit cylindrical pedicels bearing uniform dividing zones, basal pores, basal attachment tissue, and resorbed tooth loci. The labiolingual diameter of tooth pedicels at the dividing zone in *E. micropodia* ranges from 0.09–0.13 mm (Jenkins et al., 2007), and those at the anterior end of the dentary in *D. annectens* are approximately 0.11 mm (Sigurdsen & Bolt, 2010; Fig. 6). The labiolingual diameter of the tooth pedicels in *D. annectens, E. micropodia*, and *F. gilmorei* are smaller than that of *R. monbaroni* (~0.3 mm; Evans and Sigogneau-Russel, 2001; Jenkins et al., 2007), and gymnophionans (0.16–0.50 mm; based on a selection of 9 gymnophionan taxa measured in Wake and Wurst, 1979: table 1), suggesting acquisition of larger tooth pedicels occurred in the common ancestor of gymnophionans after the divergence of stem gymnophionomorphs like *F. gilmorei* and *E. micropodia*. The acquisition of larger tooth pedicels in the common ancestor of *R. monbaroni* and Gymnophiona is likely associated with the reduction in tooth number in the labial and lingual rows of pseudodentary dentition.

**External pitting and internal microanatomy from histological section:** In *Funcusvermis gilmorei*, the external (lateral) surfaces of the pseudodentary and maxillopalatine are covered in circular to subcircular pits (Extended Data Fig. 4). Internal microanatomy observed in a histologically sectioned right pseudodentary (PEFO 44432) shows that these pits are the external expression of short canals that curve and sometimes branch within the bone of the pseudodentary; these canals terminate within the bone of the pseudodentary without connecting to the major neurovascular canals associated with the mandibular branches of CN V (Extended Data Fig. 4). Pits present on the external surfaces of the pseudodentary and maxillopalatine in *F. gilmorei* resemble similar pits on the same elements in *Eocaecilia micropodia* (Jenkins et al., 2007), *Rubricacaecilia monbaroni* (Evans and Sigogneau-Russel, 2001), and living gymnophionans (e.g., Wilkinson et al., 2011). Histological analysis of similar pit structures present on the lateral surfaces of anterior skull bones in the living gymnophionan *Siphonops annulatus* shows that these pits act as anchor sites for dermal collagen networks that facilitate strong skin-to-bone cohesion in the snout during the frictional stresses produced by subterranean burrowing (Jared et al., 2018). These collagen networks house glands that produce a lipid-rich mucus secretion, and this secretion is suggested to act as a friction-reducing lubricant aiding in subterranean burrowing (Jared et al., 2018). Although the internal anatomy and function of these pits have only been examined in *S. annulatus*, the similarity of their overall morphology to those of other living gymnophionans suggests that they are possibly homologous and serve similar functions throughout the clade. The internal morphology of these pits in *F. gilmorei* resembles those of *S. annulatus* (Jared et al., 2018; fig. 3a). Their presence in *F. gilmorei* could serve as an osteological correlate for tight skin-to-bone attachment and lubricating secretions as in living gymnophionans, but this cannot be tested until gymnophionomorph fossils are found with preserved soft tissue. Similar pits are not apparent in dissorophoids and early batrachians, suggesting these structures may be a gymnophionomorph synapomorphy. Albanerpetontids bear similar pitting, usually described as ‘nutrient foramina’ on the external surfaces of the dermal bones of the skull (e.g., Gardner & Averianov, 1998; Gardner, 1999; Gardner et al., 2003; Sweetman and Gardner, 2013).

**Post-atlantal vertebra***.* PEFO 45910 represents a posterior trunk or caudal vertebra (Extended Data Fig. 3). The notochordal and amphicoelous centrum bears a faint midventral keel and is broadly coossified dorsally to the neural arch. The neural arch is wide and flat with laterally flared zygopophyses, and a mediolateral constriction behind the level of the diapophysis gives the element an hourglass-shaped profile in dorsoventral view. The diapophysis is formed by an anterolaterally projecting process on the right lateral side of the vertebra at the junction of the centrum and prezygapophysis. The anteroventral and posteroventral ends of the centrum are broken, leaving the presence of robust parapophysis or basipophyses like those of living gymnophionans uncertain. PEFO 45910 is assigned to the Gymnophionomorpha based on the absence of a neural spine, presence of a midventral keel, and medially interconnected postzygaphophyses that form a posteriorly hyperextended lamina for overlapping the following vertebra. These same features are present in the trunk and caudal vertebra of *Eocaecilia micropodia,* suggesting they were acquired early in gymnophionomorph evolution. As in *E. micropodia*, PEFO 45910 lacks the elaborate parapophyseal and basipophyseal processes present in *Rubricacaecilia monbaroni* and other gymnophionans (Evans & Sigogneau-Russel, 2001), suggesting that *E. micropodia* and *Funcusvermis gilmorei* represent a stage in gymnophionan vertebral evolution prior to the derivation of these vertebral features. The rectangular (in dorsoventral view) and dorsoventrally compressed form of PEFO 45910 resembles the form of near-terminal vertebrae in living gymnophionans (Wake, 2003; figs. 19, 20), but differs from the form of caudal vertebra in tailed gymnophionans like *Epicrionops bicolor* where the neural arch is dorsally tall and the hypapophyses project ventrally (Wake, 2003; fig. 22). Given the disarticulation and dissociation of known material of *Funcusvermis gilmorei*, the presence of intercentra (as in *E*. *micropodia*) currently cannot be determined. The presence of cylindrical pleurocentra is shared with amphibamids such as *Doleserpeton annectens* and *Amphibamus grandiceps*. This feature is otherwise unknown in temnospondyls, although plagiosaurids exhibit a single elongate centrum of uncertain homology with the centra of other early tetrapods (e.g., Panchen, 1959; Shishkin, 1987, 1989; Warren & Snell, 1991; Danto et al., 2016). However, unlike the caudal and trunk vertebra of *D. annectens,* which bear tall neural spines (Sigurdsen and Bolt, 2010; fig. 7D), the neural arch is flat in *F. gilmorei.* The flattened neural arch and dorsoventrally compressed shape of PEFO 45910 may suggest that the body of *F. gilmorei* was tubular as in other gymnophionomorphs.

**Femur.** PEFO 43811 represents the proximal end of a right femur and is the only appendicular element referred to *Funcusvermis gilmorei* (Extended Data Fig. 3). The dorsoventrally oriented femoral head is bulbous and oval in dorsal view. A shallow depression occupies the lateral surface ventral to the femoral head, and a large concave fossa is present on the medial surface of the element ventral to the femoral head, separating the head from a prominent subtriangular medial trochanter (Extended Data Fig. 3). The femoral shaft below the medial trochanter is broken and missing. PEFO 43811 resembles femora referred to *Eocaecilia micropodia* and *Rubricacaecilia monbaroni,* and its presence in *F. gilmorei* suggests that it possessed limbs as in these non-gymnophionan gymnophionomorphs. The femoral head is bulbous and oriented dorsoventrally as in *E. micropodia*, differing from the femur of *R. monbaroni*, which is less bulbous and hooked posteriorly. As in *E. micropodia* and caudates, shallow depressions occupy the medial and lateral sides of the bulbous head. On the medioventral side of the proximal shaft is a rounded trochanter, less prominent and acute than that of *E. micropodia*, more closely resembling the trochanter in *R. monbaroni*. A trochanter is common to many early tetrapods (e.g., Sumida, 1997), but a rounded trochanter offset from the shaft is shared with only a few clades, including amphibamiforms (e.g., Daly, 1994; Schoch & Rubidge, 2005; Sigurdsen & Bolt, 2010); see also *Balanerpeton woodi* and *Lydekkerina huxleyi* (Milner & Sequeira, 1993; Pawley & Warren, 2005). In other temnospondyls, the trochanter may not be separated from the shaft (e.g., Schoch, 1999; Pawley, 2007; Sequeira, 2009) or may not form a ventrally convex rounded surface, instead forming a flat rugose surface (e.g., Sullivan et al., 2000; Sulej, 2007). The bulbous ovular head of the proximal femoral end in *E. micropodia*, *R. monbaroni*, and *F. gilmorei* differs from the more anteroposteriorly elongate form present in dissorophoids and caudates (Jenkins et al., 2007; Carroll, 2007).

**3. Comparisons to stereospondyl temnospondyls and *Chinlestegophis jenkinsi***

The recently described diminutive stereospondyl *Chinlestegophis jenkinsi* possesses several features possibly homologous to morphologies present in *Eocaecilia* *micropodia* and gymnophionans, leading to a novel hypothesis that *E. micropodia* and gymnophionans form a sister clade to *C. jenkinsi* and nested within stereospondyl temnospondyls (Pardo et al., 2017a)*.* Based on the phylogenetic results of Pardo et al. (2017a), ‘higher temnospondyls,’ including Stereospondyli, are included within total group Lissamphibia, recalibrating the estimated gymnophionan-batrachian divergence to at least 315 Ma. The results of this study have been contested by subsequent studies on differing grounds, which are detailed and expanded upon below.

**Phylogenetic analyses***.* Methodologically, Silva & Wilkinson (2021) used the dataset of Pardo et al. (2017a) as a case study for ‘large island bias’ in the majority rule consensus, the topology that Pardo et al. (2017a) used to summarize their most parsimonious trees (MPTs; figure S7 of Pardo et al., 2017a). Silva & Wilkinson (2021) identified five different islands, with the largest island comprising more than half of the 882 MPTs and thus strongly influencing the total 50%-majority rule consensus. Though the largest island’s consensus recovers the diphyletic origin of Lissamphibia for which Pardo et al. (2017a) advocated, three of the other islands of their analysis recover the traditional single origin of Lissamphibia from within Dissorophoidea (Silva & Wilkinson, 2021). These islands are smaller, but as demonstrated by Sumrall et al. (2001), island size is not a reliable proxy for support, and large island size may reflect a higher degree of instability. The traditional topology of a monophyletic lissamphibian origin from Dissorophoidea is therefore equally parsimonious with the novel diphyletic origin presented by Pardo et al. (2017a) in that study. Large island bias is particularly relevant for interpreting the results of Pardo et al. (2017a) because the majority-rule topology is the only one that they presented for their parsimony analysis; they did not include the strict consensus in either the text or in the supplemental information, but it can be derived from their reported nodal frequencies. The strict consensus topology is poorly resolved, with most major temnospondyl clades not recovered; those results differed from the topology found by Schoch (2013) based on the matrix used therein, the same matrix on which the dataset of Pardo et al. (2017a) was largely based. In a strict consensus topology of the matrix of Pardo et al. (2017a), *Chinlestegophis jenkinsi* would not be recovered as the sister taxon of gymnophionans or of brachyopoid. Instead, it is a single branch of a large polytomy of later-diverging rhachitomes alongside morphologically and temporally disparate taxa such as the early Permian amphibamiform *Doleserpeton annectens* (a close relative of at least batrachians in any hypothesis with a temnospondyl origin), the early Permian stereospondylomorph *Sclerocephalus haeuseri*, the Early Triassic lydekkerinid *Lydekkerina huxleyi*, the Early Triassic capitosaur *Benthosuchus sushkini*, the Early Jurassic chigutisaurid *Siderops kehli*, and *Eocaecilia micropodia*. The use of majority-rule consensus topologies for purposes other than strict evolutionary relationships is debated (e.g., Barrett et al., 1991; Swofford, 1991; Sharkey & Leathers, 2001; Holder et al., 2008; Wheeler & Pickett, 2008), but there is a broader consensus that this consensus method is not appropriate for inferring relationships (e.g., Nixon & Carpenter, 1996; Sumrall et al., 2001) and should be restricted to visualizing relationships found in some but not all MPTs. Therefore, treatment of the results of Pardo et al. (2017a) with the more rigorous consensus method would not confer stronger support for the diphyletic origin hypothesis over the traditional hypothesis of a monophyletic origin from within dissorophoid temnospondyls.

Other studies that used some form of the dataset of Pardo et al. (2017a) have also not recovered a diphyletic origin of Lissamphibia. In the redescription of the stem-caudate *Triassurus sixtelae* (Schoch et al. 2020), a slightly modified version of the character-taxon matrix of Pardo et al. (2017a) included revised character codings for *Chinlestegophis jenkinsi*, *Rileymillerus cosgriffi,* *Eocaecilia micropodia*, and *Gerobatrachus hottoni*. Results of their parsimony analysis of this modified dataset found *E. micropodia* and gymnophionans to be in a sister group relationship to a clade including albanerpetontids and batrachians, whereas *C. jenkinsi* and *R. cosgriffi* were nested within Brachyopoidea but only distantly related to *E*. *micropodia* and gymnophionans (Schoch et al., 2020). The return to the traditional topology is clearly influenced by reverted or altered codes for informative characters for these key taxa, but the results more broadly demonstrate that phylogenetic topologies resultant from analyses of this dataset are subject to significant labiality due to slight changes in the underlying dataset.

Daza et al. (2020) analyzed four different datasets, three in addition to the temnospondyl-focused matrix of Pardo et al. (2017a). The only changes to these matrices were the addition of the albanerpetontid *Yaksha perettii*; either the addition of, or some coding changes to, the terminal operational taxonomic unit (OTU) for Albanerpetontidae; and the use of implied weighting. Their reanalysis of the matrix of Pardo et al. (2017a), which densely sampled stereospondyls, also recovered a monophyletic lissamphibian originating within Amphibamiformes, that time in the strict consensus (fig. S14 in Daza et al. 2020). In the second reanalysis, *C. jenkinsi* was recovered as the sister taxon to Brachyopoidea, as in the original majority-rule consensus of Pardo et al. (2017a), but Gymnophiona clustered with other lissamphibians.

Marjanović & Laurin (2019) did not perform a revised analysis of the Pardo et al. (2017a) matrix, but they did examine the results and reported a few caveats. They noted that lissamphibian monophyly within Dissorophoidea or monophyly within Stereospondyli (the latter not previously hypothesized or recovered in the literature) were equally parsimonious based on a close examination of individual MPTs recovered by Pardo et al. (i.e., some MPTs recovered a monophyletic Lissamphibia within Stereospondyli as the sister taxon of *Chinlestegophis jenkinsi* + *Rileymillerus cosgriffi*, but this was not recovered in either the total majority-rule consensus, that of any individual island, or the strict consensus trees). As with Silva & Wilkinson (2021), the number of MPTs that recover the diphyletic origin of Lissamphibia relative to those recovering another topology cannot be directly tied to relative support; all MPTs are, by definition, equally parsimonious. Those authors also provided a slightly more detailed reanalysis of the Pardo et al. (2017a) matrix in a preprint of their final paper that was not carried forward (Marjanović & Laurin, 2018:57­­–58).

Finally, the results of both our parsimony (including all MPTs) and Bayesian analyses find gymnophionans to be the sister taxon to albanerpetontids + batrachians, suggesting that characters supporting the sister group relationship of *Chinlestegophis jenkinsi* with *Eocaecilia micropodia* + Gymnophionomorpha are homoplastic rather than homologous. Though our analysis is a derivate of the Schoch et al. (2020) phylogenetic dataset, we made substantial changes (see Supplementary Information 4), which include restoring of many character states introduced by Pardo et al. (2017a) that were changed by Schoch et al. (2020) for key taxa, such as coding the presence of a LEP in *C. jenkinsi* and *Rileymillerus cosgriffi* (changed to unknown by Schoch et al. 2020). Additionally, substantive changes were made to *Gerobatrachus hottoni* and *Doleserpeton annectens* that diminish their coded similarities with some or all lissamphibians (e.g., coding *D. annectens* as unknown (?) for the absence of an ectopterygoid and other associated characters). Therefore, our analysis represents a new, independent datapoint in support of the traditional temnospondyl hypothesis (in contrast to other largely unmodified derivates, which represent pseudoreplicates).

**Phenetic comparisons.** The interpretation of *Chinlestegophis jenkinsi* as a stem caecilian also were qualitatively challenged by Santos et al. (2020). The points raised there are repeated here in the context of a broader discussion that largely focuses on purported affinities of *C. jenkinsi* with brachyopoids and gymnophionans specifically; the temnospondyl and stereospondyl affinities, as well as the autapomorphies within the context of Stereospondyli, are not regarded as controversial by other workers. Like Santos et al. (2020), much of the discussion here emphasizes that purported synapomorphies are also found in other tetrapods, both extinct and extant, and could therefore be homoplastic. The original diagnosis is listed below for reference, and the comparisons proceed in order of listed features. As a nomenclatural note, Brachyopoidea here encompasses Brachyopidae, Chigutisauridae, and Plagiosauridae (following Schoch, 2013); this merely reflects the current state of temnospondyl phylogenetics and is not an explicit endorsement of this hypothesis over the traditional composition to the exclusion of plagiosaurids (e.g., Warren & Marsicano, 2000).

Diagnosis of *Chinlestegophis jenkinsi* from Pardo et al. (2017a: pg. E5389): “Small stereospondyl with a combination of brachyopoid and caecilian characteristics. Unique features include the following: lateral line sulcus restricted to suborbital margins of jugal and postorbital; parietal–tabular narrowly contact [may be shared with *Compsoceros*]; postfrontal anterior process long, forming the majority of the dorsomedial border of the orbit; finger-like process of prefrontal interlocks with notch on post-frontal. Shared features with stereospondyls include the following: parasphenoid strongly sutured to pterygoid, supratemporal excluded from otic notch, secondary upper tooth row. A shared feature with stereospondyls and caecilians is opisthotics coossified to exoccipitals. Shared features with brachyopoids and caecilians include lacrimal coossified to maxilla and two small posterior processes (‘horns’) on the occipital exposure of the tabular, just posterior to otic notch (as in chigutisaurids). Shared features with *Rileymillerus* and some other small temnospondyls comprise palatine exposed laterally in ventral margin of the orbit [LEP (lateral exposure of palatine)]. Shared features with *Rileymillerus* and caecilians include the following: orbits small and laterally directed. Shared features with caecilians include double tooth row on mandible; quadrate completely anterior to ear; broad, parallel-sided parasphenoid cultriform process >20% skull width; occipital condyles extend far beyond posterior edge of skull roof; and pterygoquadrate. Shared features with some other temnospondyls but not caecilians include large, laterally directed otic notch.”

**Opisthotics coossified to exoccipitals.** The developmental fate of the opisthotic is widely variable among stereospondyls. In some taxa like the iconic large capitosaur *Mastodonsaurus giganteus*, it definitively coossifies with the prootic to form a single otic element that in turn coossifies with the exoccipital (Schoch, 1999a, 2002). However, *M. giganteus* is not an appropriate representative for the generalized stereospondyl condition because it is unusually heavily ossified for a stereospondyl, which likely results from its abnormally large size (Schoch, 1999a). Other capitosaurs do not exhibit a coossified otic (e.g., Howie, 1970; Warren, 1980; Morales & Shishkin, 2002; Arbez et al., 2017) or have an otic that is not coossified with the exoccipital (e.g., Schoch, 1997). In general, the otic region remains very poorly described in stereospondyls, mostly due to a lack of exposure and perhaps a lack of ossification (e.g., Chernin, 1977; Warren & Hutchinson, 1983; Sengupta, 1995; Bjerring, 1999; Marsicano, 1999; Yates, 1999; Damiani & Kitching, 2003; Jeannot et al., 2006; Damiani et al., 2009; Maganuco & Pasini 2009; Warren et al., 2011; Marzola et al., 2017; Schoch, 2018).

In addition to the general paucity of described otic capsules among stereospondyls, many clades include members in which the opisthotic remained cartilaginous, such as trematosaurs (e.g., Sulej, 2007; Schoch, 2019b), capitosaurs (Welles & Cosgriff, 1965), and rhinesuchids (Dias & Schultz, 2003), and others exhibit distinct opisthotics not coossified with the exoccipitals or with the prootics (e.g., Wilson, 1941; Hewison, 2007). Furthermore, coossified otics that are not coossified with the exoccipitals occur in many large and/or terrestrial non-stereospondyls, such as *Edops craigi* (Romer & Witter, 1942), *Eryops megacephalus* (Sawin, 1941), and ‘*Dendrerpeton*’ (Robinson et al., 2005), as well as in the aquatic *Trimerorhachis insignis* (Schoch, 1999b). As discussed by Witzmann et al. (2012:46), the presence of a compound otic in many stem tetrapods may indicate that this is the plesiomorphic condition for crown tetrapods. Pardo et al. (2017a) also made no mention of the prootic or of a compound otic, and the coossification of the opisthotic with the exoccipital but not with the prootic would be a very peculiar condition. In short, the opisthotic and exoccipital are very rarely documented to become coossified in stereospondyls, and this feature should therefore not be treated as a stereospondyl synapomorphy or as a synapomorphy with caecilians.

**Lacrimal coossified with maxilla.** The interpretation of the circumorbital bones (i.e., the presence/absence of a lacrimal and presence/absence of a LEP) is both contentious and critical to the interpretation of *Chinlestegophis jenkinsi*. Pardo et al. (2017a) argued that the lacrimal is not a distinct ossification in *C. jenkinsi*, having coossified with the maxilla, with a corresponding anterior shift of the prefrontal and the postfrontal to frame the orbit anteromedially. The purported coossification of the lacrimal with the maxilla is based on the position of the nasolacrimal duct, typically housed within the lacrimal in temnospondyls (see visualization by Anderson & Bolt, 2013, for example), and which occurs within the dorsal lamina of the maxilla in *C. jenkinsi*. The lacrimal is absent in brachyopids and chigutisaurids (the historic Brachyopoidea; Warren & Marsicano, 2000) but is present in plagiosaurids (e.g., Jenkins et al., 2008; Damiani et al., 2009; Schoch & Witzmann, 2012; Schoch et al., 2014), which nest within Brachyopoidea in recent analyses (e.g., Schoch, 2013, and derivates thereof). A lacrimal is absent in most rhytidosteids, an early diverging stereospondyl clade with substantial cranial variation (e.g., Dias-da-Silva & Marsicano, 2011), as well as in some trematosaurs (e.g., Cosgriff & Garbutt, 1972; Steyer, 2002). The enigmatic *Laidleria gracilis* either has a very small lacrimal or has no lacrimal at all (Warren, 1998b). This taxon has often been considered a rhytidosteid if interpreted to lack a lacrimal (e.g., Dias-da-Silva & Marsicano, 2011) but has alternatively been classified as a trematosaur (e.g., Kitching, 1957; McHugh, 2012); in the clade Laidleriidae with *Uruyiella liminea* as the sister group to plagiosaurids but outside of Stereospondyli (Piñeiro et al., 2007a); and as the exclusive sister taxon of plagiosaurids within Stereospondyli (e.g., Yates & Warren, 2000; Schoch, 2013, and derivates thereof). Finally, the lacrimal is absent in tupilakosaurid dvinosaurs (e.g., Shishkin, 1967, 1973; Warren, 1998a).

In none of these taxa has it been demonstrated that the lacrimal has coossified with the maxilla via the proxy of the nasolacrimal duct that was utilized in *Chinlestegophis jenkinsi* (high-resolution CT analyses of temnospondyls are rare in general). Therefore, whereas the absence of a distinct lacrimal is indeed a shared feature, a lacrimal coossified with the maxilla is not an unequivocally shared feature (let alone a synapomorphy) with Brachyopoidea (as opposed to the diagnosis of *C. jenkinsi*). A dorsal/medial expansion of the maxilla (sometimes the ‘dorsal lamina’) posterior to the naris, as found in brachyopids and chigutisaurids, cannot be reliably assumed to represent the coossified lacrimal. This expansion also occurs in taxa with in which the lacrimal is both present and well-separated from the naris, as the maxilla frequently expands to meet the nasal such as in many capitosaurs and trematosaurs (e.g., Case, 1931; Dutuit, 1976; Damiani, 2001, 2008; Morales & Shishkin, 2002; Steyer, 2003; Liu & Wang, 2005; Sulej & Majer, 2005; Sulej, 2007; Novikov, 2012b; Liu, 2016; Lucas et al., 2016) or taxa with anteriorly narrowing lacrimals that meet the naris (e.g., some dissorophoids; *Dendrerpeton helogenes*; *Doleserpeton annectens*; Holmes et al., 1998; Reisz et al., 2009; Sigurdsen & Bolt, 2010; Holmes et al., 2013; Anderson et al., 2020).

**Presence of a lateral exposure of the palatine.** A lateral exposure of the palatine (LEP) is interpreted to occur at the anteroventral corner of the orbit of *Chinlestegophis jenkinsi*. The identification of the LEP is particularly controversial because it rarely occurs outside of Dissorophoidea, the longstanding candidate clade for a monophyletic lissamphibian origin. A similarly positioned but proportionately smaller element was identified as a LEP in *Rileymillerus cosgriffi* (Bolt & Chatterjee, 2000). A LEP is also found in the eobrachyopid dvinosaurs (Sequeira, 1998; Englehorn et al., 2008; Extended Data Fig. 9) and some tupilakosaurid dvinosaurs (e.g., Shishkin, 1967, 1973; Warren, 1998a); these exposures are more similarly sized to that of *C. jenkinsi* or dissorophoids (Extended Data Fig. 9). While dvinosaurs were once thought to be closely related to brachyopids (hence Eobrachyopidae), they are now considered to be only distantly related (e.g., Schoch, 2013, and derivates thereof; but see dissenting view of Milner on p. 95 of Schoch & Milner, 2014, for example). A LEP also occurs in the uncertainly-placed *Perryella olsoni* (historically Temnospondyli *incertae sedis* or a dvinosaur; e.g., Carlson, 1987; Ruta & Bolt, 2006), *Macrerpeton huxleyi* (historically an edopoid; e.g., Romer, 1947; Hook & Baird, 1986; Hook, 1993), and *Stegops newberryi* (historically a zatracheid; e.g., Romer, 1947). All three were considered indeterminate dissorophoids by Schoch & Milner (2014), based in part on the presence of a LEP (see also Schoch & Milner, 2021, for recent redescription of *M. huxleyi*).

The above discussion of the lacrimal and the LEP are meant to illustrate that each character has a more complicated distribution among Temnospondyli than is presented in either the main text or the supporting information of Pardo et al. (2017a). Even the putative combination of an absent lacrimal and a present LEP is not exclusive to *Chinlestegophis jenkinsi* and *Rileymillerus cosgriffi*. Tupilakosaurid dvinosaurs also exhibit this combination (e.g., Shishkin, 1967, 1973; Warren, 1998a; Milner & Sequeira, 2004), but no tupilakosaurid was sampled by Pardo et al. (2017a), even though two tupilakosaurids were originally sampled by Schoch (2013): *Thabanchuia oomie* and *Tupilakosaurus wetlugensis*. The size and anterior extent of the LEP is variable among the tupilakosaurids, whereas in the non-dvinosaurs, the LEP occupies a relatively small part of the space anteroventral to the orbit that is typically occupied by the lacrimal. Schoch (2008a:103) disputed the interpretation of a LEP in *R. cosgriffi* and instead interpreted it as an unusually short lacrimal, like that observed in some metoposaurids, trematosaurids, and the metoposauroid *Callistomordax kugleri*. This followed Schoch (2006:32), who identified a lacrimal overplated by the prefrontal, producing a short dorsal exposure, in the trematosaur *Trematolestes hagdorni*; he further suggested that complete overplating might explain the apparent loss in other trematosaurs (but made no comment on other temnospondyls without lacrimals). Schoch’s (2008a) analysis therefore recovered *Rileymillerus cosgriffi* as a trematosaur closely related to metoposaurids, and this interpretation and topology were then propagated (e.g., McHugh, 2012; Schoch, 2013). Schoch et al. (2020) recoded the lacrimal and the LEP as unknown for both *Ch. jenkinsi* and *R. cosgriffi* as part of their revisions to the matrix of Pardo et al. (2017a). These coding changes likely contributed (in part) to the return to the traditional temnospondyl monophyly hypothesis in the same way that their shared coding by Pardo et al. (2017a) contributed to their position as stem caecilians and the sister group to Brachyopoidea. Pardo et al. (2017a) stated that the lateral exposure is contiguous with a dentigerous palatal element, the most compelling line of evidence for interpretation as a LEP, but if so, the palatine is badly fractured based on the presented rendered volumes (movies S5 and S6 therein) and could be interpreted as lacking a dorsal exposure. The condition in *R. cosgriffi* is impossible to determine from the non-reconstruction figures of Bolt & Chatterjee (2000). As detailed in Supplementary Information 4, we restored these codes because we found no unequivocal reason to overturn them (the approach we took to recoding broadly), but the issue warrants further attention. Extended Data Fig. 9. illustrates the phylogenetic distribution and comparative morphology of a lateral exposure of the palatine (LEP) and the loss of the lacrimal in Temnospondyli.

**Presence of paired projections on the occipital face of the tabular.** Pardo et al. (2017a) stated that two small posterior processes (‘horns’) on the occipital face of the tabular of *Chinlestegophis jenkinsi*, posterior to the otic notch, are shared with brachyopoids and caecilians. The syntax of this part of the diagnosis suggests that these authors believe that these processes are common to brachyopoids, but only in chigutisaurids are they found posterior to the otic notch. However, this feature does not occur in any chigutisaurids, either posterior to the notch or anywhere else (e.g., *Compsocerops cosgriffi*, *Keratobrachyops australis*, *Kuttycephalus triangularis*, *Pelorocephalus mendozensis*, *Pelorocephalus tenax*; Warren, 1981; Sengupta, 1995; Marsicano, 1999). The condition is indeterminate and/or not reconstructed as such in *Koolasuchus cleelandi*, *Pelorocephalus cacheutensis*, and *Siderops kehli* (Warren & Hutchinson, 1983; Warren et al., 1997; Marsicano, 1999). The tabular horns of chigutisaurids do project well past the level of the relatively open otic notch, but this technically also occurs in any taxon in which the tabular horn posteriorly brackets the otic notch, including in many other stereospondyls (especially capitosaurs; e.g., Schoch & Milner, 2000:figs. 92–94) and in some morphologically and phylogenetically disparate clades such as olsoniform dissorophoids (e.g., Berman et al., 1985; Reisz et al., 2009). The chigutisaurid condition, in which the projection is confluent with the skull roof and thus ornamented dorsally, is not of clear homology to the condition of *C. jenkinsi* in which it is not even certain that one or both of the posterior projections are homologous with the classic temnospondyl tabular horn, as neither is confluent with the skull roof or ornamented. Neither are these processes from an occipital exposure of the tabular a typical plagiosaurid (e.g., Damiani et al., 2009; Schoch & Witzmann, 2012; Witzmann et al., 2012; Schoch et al., 2014) or brachyopid (e.g., Damiani & Warren, 1996; Warren & Marsicano, 1998; 2000; Warren et al., 2011) feature.

Pardo et al. (2017a) cited *Batrachosuchus* and *Vigilius*, both brachyopids, as having these processes. *Batrachosuchus* does sometimes have paired processes on the occipital face, but only one of these is on the tabular. In *Batrachosuchus concordi*, the only other posterior projection from the occiput is clearly laterally offset and on the squamosal (Chernin, 1977:fig. 1). In *Batrachosuchus henwoodi*, the quadrate ramus of the pterygoid probably projected posteriorly such that it is exposed in dorsal view (Warren & Marsicano, 1998:fig. 3); it seemingly lacks any posterior projection from the occipital surface of the tabular. This is similar to *Bathignathus watsoni* (formerly placed in *Batrachosuchus*; e.g., Watson, 1956:fig. 5; Welles & Estes, 1969:fig. 17), which was likely used as the basis for a reconstruction of *Bathignathus poikilops* that has a dorsally exposed quadrate ramus but no tabular processes (Damiani & Jeannot, 2002:fig. 3). The posterior projection of the quadrate ramus is also observed in *Compsocerops cosgriffi* (Sengupta, 1995:318, fig. 4) and *Vigilius wellesi* (e.g., Welles & Estes, 1969:fig. 26; Warren & Marsicano, 2000: fig. 3D). In *V. wellesi*, the only other occipital projection visible in dorsal view is laterally offset from the first (the quadrate ramus of the pterygoid) and therefore is from the squamosal, as in *Batrachosuchus concordi* and *Bath. watsoni*; there is no projection from the occipital surface of the tabular (Welles & Estes, 1969:fig. 27). In these taxa, the squamosal forms a posteriorly convex margin but not a distinct process. *Batrachosuchus browni*, the taxon depicted by Pardo et al. (2017a:fig. 3) has only one very underdeveloped process from the skull roof portion of the tabular, as evidenced from both their figure in dorsal view and earlier figures in all views by Chernin (1977:fig. 5).The dual projections from the occipital surface of the tabular in *Chinlestegophis jenkinsi* can therefore not be considered to be shared with any brachyopid, let alone with most brachyopids, most chigutisaurids, or most brachyopoids.

**Small, laterally facing orbits.** The small, laterally facing orbits of *Chinlestegophis jenkinsi* are a rare feature among temnospondyls and generically shared with caecilians in certain comparative contexts (i.e., the orbits of *C. jenkinsi* are still much bigger than those of caecilians but are certainly smaller than most other temnospondyls). Most small-bodied temnospondyls (either juveniles or diminutive adults) exhibit proportionately large orbits, like in amphibamiform dissorophoids (e.g., Anderson et al., 2008a, 2008b; Sigurdsen & Bolt, 2010), a feature also found in batrachians, especially anurans. Dissorophoids in general trended towards large, laterally facing orbits. Orbit size scales with negative allometry relative to skull size in temnospondyls, as in tetrapods at large, so very large taxa have orbits perhaps even proportionately smaller than *C. jenkinsi* (e.g., *Dasyceps bucklandi*, *Nigerpeton ricqlesi*, and *Saharastega moradiensis*; Paton, 1975; Sidor et al., 2005), but these are flat-headed taxa, and thus the orbits face dorsally.

**Second tooth row on the lower jaw.** Pardo et al. (2020) noted a double tooth row on the mandible to be shared between *Chinlestegophis jenkinsi* and caecilians. Conversely, Santos et al. (2020) argued that this occurred in other peripherally related temnospondyls (citing Yates & Warren, 2000; character state 101-2 therein). Yates & Warren (2000) code this state in five terminal OTUs: *Dvinosaurus* (coded based on *Dvinosaurus primus*), *Almasaurus*, Plagiosauridae, *Siderops*, and ‘Undescribed Genus’ (=*Thabanchuia oomie*). The condition in two of these taxa, *Thabanchuia* and *Siderops* is of dubious equivalency. There is no evidence for a row of coronoid teeth in *T. oomie*, only a few large ‘tusks’ (Warren, 1998a), which are atypical on the coronoids in temnospondyls. The original phylogenetic coding may be a matter of semantics regarding the differences between ‘teeth,’ ‘denticles,’ and ‘fangs/tusks,’ but as interpreted by Warren (1998a), there is no ‘row’. The condition of *Siderops kehli* is of also questionable, as the row is loosely organized and also includes a patch of denticles on the middle coronoid (Warren & Hutchinson, 1983). This condition of a ‘mixed’ dentition with loosely organized teeth and denticle fields seems to occur among several other stereospondyl lineages, such as lydekkerinids (Jeannot et al., 2006; Hewison, 2007) and brachyopids (Warren et al., 2011), and also occurs in the probable chigutisaurid *Keratobrachyops* *australis* (Warren, 1981).

In the other three taxa, *Dvinosaurus primus*, *Almasaurus habbazi*, and the plagiosaurid *Gerrothorax pulcherrimus* (Bystrow, 1938; Dutuit, 1976; Hellrung, 2003; Schoch & Witzmann, 2012), there are gaps of variable size between the row of each coronoid (Extended Data Fig. 8). The distribution within plagiosaurids also appears to be quite variable; *Plagiosuchus pustuliferus* lacks coronoid dentition altogether (Damiani et al., 2009), whereas *Megalophthalma ockerti* appears to have a row continuous across all three coronoids (Schoch et al., 2014). The posterior coronoid of *G. pulcherrimus* also consists of a patch of small teeth, not a single row (e.g., Schoch & Witzmann, 2012; Extended Data Fig. 8).

A sixth example of a row of coronoid teeth, unpublished at the times of Yates & Warren (2000) and not mentioned by Santos et al. (2020), occurs in lapillopsids; Yates & Sengupta (2002) listed this is a synapomorphy of the clade. In the best example, *Manubrantlia khaki*, there are small gaps between the row of each coronoid (Yates & Sengupta, 2002). A seventh example, recently reported by Dias et al. (2020), occurs in a Brazilian rhinesuchid, *Rastosuchus hammeri* (Extended Data Fig. 8). As best as can be determined from renderings of *Chinlestegophis jenkinsi*, the row of coronoid dentition is also not perfectly continuous (Pardo et al., 2017a:fig. S3; Extended Data Fig. 8). There are also a few places where projections appear outside of the tooth row in *Chinlestegophis jenkinsi*, particularly on the middle coronoid, but these are not illustrated as teeth in the interpretive line drawing, and it is unclear whether they are artifacts or in fact a partial shagreen. Only five teeth can be identified on the posterior coronoid of *Rileymillerus cosgriffi*, which is insufficient to differentiate it from many highly nested temnospondyls like capitosaurs, which restrict coronoid dentition to a row on the posterior coronoid.

A questionable eighth example might be the capitosaur *Edingerella madgascariensis*, which Steyer (2003:549) described as having “rows of denticles” across the coronoids. By Steyer (2003)’s comparison to *Lapillopsis*, this is interpreted to mean a row on each coronoid, rather than multiple rows (a shagreen) on each coronoid, and the characterization as ‘denticles’ may again be a matter of semantics (tooth smaller than the marginal dentition). This dentition was previously noted by Warren & Hutchinson (1988b). This condition might also occur in *Watsonisuchus aliciae* (sometimes placed in *Rewanobatrachus* sensu Schoch & Milner, 2000, or *Warrenisuchus* sensu Maganuco et al., 2009) in which a row of three teeth is found on the posterior coronoid with a row of two teeth on the poorly preserved middle coronoid (Warren & Hutchinson, 1988a). This could, however, be more like the very short row of the capitosaur *Mastodonsaurus giganteus* (Schoch, 1999a) in which only three teeth occur on the posterior coronoid or like the condition of the trematosaur *Benthosuchus gusevae* (Novikov, 2012b) in which there are three teeth on the middle coronoid and only one on the posterior coronoid (Extended Data Fig. 8). There is also the condition reported in an indeterminate capitosaur from Uruguay by Piñeiro et al. (2007b) in which one specimen has a double row of teeth on at least the middle and posterior coronoids. A double row only on the posterior coronoid also occurs in the trimerorhachid dvinosaur *Lafonius lehmani* (Berman, 1973), which has ‘tusks’ on the other two coronoids like in *Thabanchuia oomie*. A semi-continuous row of teeth across all three coronoids thus occurs in at least five different temnospondyl families with a bracket encompassing practically the entirety of Temnospondyli. As suggested by Piñeiro et al. (2007b) in comparing their capitosaur material with unspecified Brazilian rhinesuchid material that also displays a double row of coronoid teeth, the presence of a coronoid tooth row in temnospondyls might represent retention of the plesiomorphic tetrapod condition.

In our opinion, the more interesting observation that may conflict with the interpretation of homology is that most brachyopoids have a restricted set of coronoid dentition. Brachyopids usually have teeth only on the posterior coronoid (e.g., Welles & Estes, 1969; Warren & Davey, 1992; Damiani & Warren, 1996; Warren & Marsicano, 2000; Damiani & Kitching, 2003; Ruta & Bolt, 2008), and *Bathignathus poikilops* has lost coronoid teeth altogether (Damiani & Jeannot, 2002). Chigutisaurids may have teeth on the middle and the posterior coronoids, but these are patches, not a row (e.g., Marsicano, 1999); later diverging taxa often lack teeth altogether (Sengupta, 1995; Warren et al., 1997). The reduction of teeth from the plesiomorphic shagreen to a loose row of teeth typifies stereospondyl evolution. Most capitosaurs and trematosaurs have a short row of relatively large teeth only on the posterior coronoid, and metoposaurids have lost coronoid dentition altogether. The lower jaw is too poorly known in most plagiosaurids to characterize the distribution within the clade; only two taxa definitively have a semi-continuous row across all three coronoids (Schoch & Witzmann, 2012; Schoch et al., 2014). Early diverging stereospondyls like lydekkerinids and rhytidosteids have a shagreen of denticles on all three coronoids (e.g., Warren & Black, 1985; Jeannot et al., 2006), although a few present a mixture of differently sized dentition as in *Bothriceps* and *Siderops* (e.g., *Lydekkerina*; Hewison, 2007). Therefore, the neat, continuous row of *Chinlestegophis jenkinsi* would probably represent the reacquisition of teeth on at least the anterior coronoid if not also the middle coronoid if this taxon is highly nested as the sister group to Brachyopoidea as found by Pardo et al. (2017a). Finally, it should be noted that a single row of coronoid teeth also occurs in many stem tetrapods (e.g., Ahlberg & Clack, 1998; Clack et al., 2012; Porro et al., 2015; Rawson et al., 2021). This is not meant as a suggestion that gymnophionomorph origins lie among a taxon presently considered as a stem tetrapod but rather indicate that this feature is both plesiomorphic for tetrapods and is not restricted to certain temnospondyls. Extended Data Fig. 8. illustrates the phylogenetic distribution and comparative morphology of a single row of coronoid teeth in Temnospondyli.

**Broad, parallel-sided cultriform process.** Pardo et al. (2017a) emphasized a broad cultriform process (>20% of the skull width) with parallel margins as a feature shared between *Chinlestegophis jenkinsi* and caecilians. That comparison implies the acquisition of a feature associated with fossoriality, even though broad cultriform processes are also found in dvinosaurs and metoposaurids (e.g., Shishkin, 1973; Warren, 1998a; Milner & Sequeira, 2004; Sulej, 2007; Brusatte et al., 2015; Lucas et al., 2016), large-bodied aquatic taxa for which fossoriality has never been suggested (Extended Data Fig. 10). These taxa may not meet the arbitrary threshold of 20% of the skull width but nonetheless clearly deviate from the plesiomorphic temnospondyl condition of a slender, rod-like process, as in brachyopoids. Some brachyopids, plagiosaurids, and rhytidosteids also have widened cultriform processes, deviating from the plesiomorphic condition (e.g., Welles & Estes, 1969; Cosgriff, 1974; Warren & Marsicano, 2000; Yates, 2000; Damiani et al., 2009; Extended Data Fig. 10). That of *Rileymillerus cosgriffi* is wide at the base but otherwise slender throughout (Bolt & Chatterjee, 2000), closer to the plesiomorphic condition. The import of whether the margins are parallel or not has yet to be explored. The process is also incomplete in the holotype of *C. jenkinsi* (up to about the mid-length of the orbit) and is not preserved in the referred specimen; therefore, some caution should be exercised in assuming that it was of a consistent width throughout. As noted by Santos et al. (2020), a similarly broad process is also found in non-temnospondyls like the lysorophian *Brachydectes newberryi*, which suggests that broadening to this degree might be a common feature of, but not exclusive to, fossorial tetrapods (see other recumbirostran ‘microsaurs’; e.g., Anderson et al., 2009; Szostakiwskyj et al., 2015). Extended Data Fig. 10 illustrates the phylogenetic distribution and comparative morphology of a broad cultriform process in Temnospondyli.

**Position of jaw articulation relative to the ear.** Pardo et al. (2017a) stated that the anterior position of the jaw articulation relative to the ear is shared between *Chinlestegophis jenkinsi* and caecilians, but the landmark is a bit uncertain because the ‘ear’ is a vague anatomical term (e.g., does it refer to the stapes, the otic notch, or an individual bone of the otic capsule). There is no mention of the ‘ear’ in the extended text, only that the jaw articulation is completely anterior to the occiput (a more typical landmark used in phylogenetic characters). The language of “completely anterior” (Pardo et al., 2017a:E5390) suggests that it is specifically the posterior face of the occipital condyles that is being utilized (this is also a typical caveat to this landmark), which seems accurate since at least part of the jaw articulation would be level with the anteriormost parts of the occiput. However, if this is the correct landmark, this feature is not exclusive to *C. jenkinsi* + caecilians (Extended Data Fig. 10). A jaw articulation anterior to the posterior face of the condyles is found in many taxa with markedly posteriorly projecting condyles, such as some brachyopids (e.g., Watson, 1956; Chernin, 1977; Damiani & Kitching, 2003; Nonsrirach et al., 2021); some chigutisaurids (e.g., Warren, 1981; Sengupta, 1995; Warren & Hutchinson, 1983); at least two plagiosaurids (Damiani et al., 2009), including intraspecific variation within *Gerrothorax pulcherrimus* (Schoch & Witzmann, 2012); metoposaurids (e.g., Dutuit, 1976; Sulej, 2007; Buffa et al., 2019); dvinosaurs (e.g., Shishkin, 1973; Sequeira, 1998; Warren, 1998a; Englehorn et al., 2008); and the enigmatic *Almasaurus habbazi* (Dutuit, 1976) and *Laidleria gracilis* (Warren, 1998b). However, it also occurs in taxa without this prominent projection, including in at least one other rhytidosteid (*Nanolania anatopretia*; Yates, 2000), possibly in some dissorophids (e.g., DeMar, 1968; Schoch, 2012; Schoch & Sues, 2013; but see Dilkes, 2020), and possibly one branchiosaurid (Boy, 1987). As with a broad cultriform process, a posteriorly situated occiput is associated with fossoriality in some other tetrapod clades, such as ostodolepid and brachystelechid ‘microsaurs’ (e.g., Carroll & Gaskill, 1978; Carroll, 1990, 1991; Anderson et al., 2009; Maddin et al., 2011; Pardo et al., 2015). Extended Data Fig. 10 Illustrates the phylogenetic distribution and comparative morphology of a posteriorly situated occiput in Temnospondyli.

**Position of occipital condyles relative to skull roof.** Pardo et al. (2017a) listed occipital condyles extending far beyond the posterior margin of the skull roof as a shared feature of *Chinlestegophis jenkinsi* and caecilians. The condition of *C. jenkinsi* does not differ appreciably from a number of other stereospondyls of purported close relatedness, like brachyopoids and *Rileymillerus cosgriffi* (e.g., Watson, 1956; Panchen, 1959; Warren & Marsicano, 2000), but it is also shared with metoposauroids (e.g., Case, 1931; Sulej, 2007; Lucas et al., 2016; Buffa et al., 2019) and some dvinosaurs (e.g., Nielsen, 1967; Sequeira, 1998; Warren, 1998a; Englehorn et al., 2008). It appears to be intraspecifically variable in the trematosaur *Trematosaurus brauni* (Schoch, 2019b). Non-metoposaurid trematosaurs, rhytidosteids, and capitosaurs typically have a reduced version of this condition that is still distinct from the plesiomorphic state with no dorsal exposure of the condyles (e.g., Damiani, 2001; Damiani & Yates, 2003; Dias-da-Silva & Marsicano, 2011; Novikov, 2012b; Marzola et al., 2017). While this condition may have become progressively developed throughout stereospondyl evolution, its occurrence in dvinosaurs is convergent in the current topological context. Whether this homoplasy relates to the head-raising capability proposed in brachyopids and plagiosaurids (e.g., Watson, 1956; Panchen, 1959; Jenkins et al., 2008) remains to be determined. Santos et al. (2020) also noted the presence of this feature in non-temnospondyls and in other lissamphibians. It can, for example, be found in the same brachystelechid and ostodolepid ‘microsaurs’ noted in the previous section.

**Presence of a pterygoquadrate.** The final feature that Pardo et al. (2017a) identified as shared between *Chinlestegophis jenkinsi* and caecilians, a pterygoquadrate, was not positively identified in *C. jenkinsi* but rather was inferred from the apparent absence or substantial reduction of the quadratojugal, the absence of distinct quadrates, and the morphology of the quadrate ramus of the pterygoid. If indeed the quadrate had coossified with the pterygoid, it would be autapomorphic among temnospondyls and indicative of caecilian affinities, but the fragmentary nature of the jaw articulation and limited resolution of generated volumes render this interpretation equivocal at present. Unlike the use of the nasolacrimal duct as a proxy for determining coossification between the maxilla and the lacrimal, no similar proxy exists for the pterygoquadrate in early caecilian evolution.

To summate, many of the features that Pardo et al. (2017a) listed as being shared between *Chinlestegophis jenkinsi* and relative exclusive clades (e.g., brachyopoids or caecilians) appear in other distantly related clades and are therefore not as unequivocal as previously depicted. Some purported apomorphies, such as paired processes from the occipital surface of the tabular, have been demonstrated to be misinterpretations. Features such as an LEP or the loss of a lacrimal evolved several times within Temnospondyli, and even the joint presence of a LEP and absence of a lacrimal is not unique to *C. jenkinsi*.

Also worth consideration in this regard is *Almasaurus habbazi*, another small-bodied Late Triassic temnospondyl characterized by a tall skull with relatively small, laterally facing orbits and a posteriorly projecting occiput. It has teeth on all three coronoids, although these occur as separated rows on each coronoid (Dutuit, 1976:fig. 94b). Bolt & Chatterjee (2000) argued that this taxon was not closely related to *Rileymillerus cosgriffi*, noting differences such as the presence of a lacrimal, the absence of a LEP, and a large otic notch. The lacrimal of *A. habbazi* is well separated from the orbit and larger than the LEP in either *C. jenkinsi* or *R. cosgriffi*; it therefore can be more confidently characterized as a true lacrimal. With that said, it is interesting to note that the lacrimal of *A. habbazi* is nearly in the same position as the nasal in *R. cosgriffi*. It should be considered that the nasal of *R. cosgriffi* is actually misidentified given that the snout, including most of the nasal, is unknown in the only specimen; there are no particular defining attributes of the nasal other than its position (i.e., it has no equivalent to the nasolacrimal duct). Because material of *R. cosgriffi* has never been CT scanned or histologically sectioned, whether the nasolacrimal duct passes the through the dorsal lamina of the maxilla as in *C. jenkinsi* is unknown. The morphology of the complete nasal of *R. cosgriffi* would be quite aberrant if the preserved part currently interpreted as a partial nasal was correctly identified. The preserved region would represent a large separated posterior process in the lateral half to form an almost ‘L-shaped’ element when complete, something not observed in *A. habbazi*, *C. jenkinsi*, or other temnospondyls. Lateral expansions of the nasal posterior to the naris are common, but a subsequent posterior extension from this expansion is not. A short lacrimal separated from at least the orbit and sometimes also the naris is found in most metoposaurids (e.g., Case, 1931; Hunt, 1993; Sulej, 2007; Lucas et al., 2016) and is one feature that Schoch (2008a) cited as evidence for the close relationship of *A. habbazi* and *R. cosgriffi* to metoposaurids. A longer lacrimal, similarly separated from the nares and the orbits, is found in the proportionately longer skulls of non-metoposaurid trematosaurs (e.g., Damiani & Yates, 2003; Novikov, 2012b; Fernández-Collet al., 2019; Schoch, 2019b; Slodownik et al., 2021) and capitosaurs (e.g., Damiani, 2001, 2008; Schoch, 2008a; Sidor et al., 2014; Liu, 2016; Marzola et al., 2017), reflecting the plesiomorphic condition for stereospondylomorphs (e.g., Schoch & Witzmann, 2009a, 2009b).

The alternative (that the lacrimal of *Almasaurus habbazi* is misidentified and/or that it could have an unidentified LEP) could also be true. However, *A. habbazi* is known from many specimens with complete snouts, so it seems unlikely that the interpreted lacrimal was a consistently fractured part of the nasal or the maxilla. Sutures are quite evident in some of Dutuit’s (1976) photographs despite their datedness, so misidentification due to poor preservation seems unlikely. It is more plausible that an LEP is present but has yet to be identified. Though an LEP was not figured by Dutuit, some historical context should also be noted; LEPs were not widely recognized until the work of Bolt (1974) on dissorophoids, which formalized the term (but see DeMar, 1968; Bolt, 1969, for earlier identification), and they are typically quite small, therefore requiring good preservation and preparation to be identified. What is now a LEP was identified prior to that in dvinosaurs (e.g., Shishkin, 1967) but was misinterpreted as coossification of the palatine with the lacrimal, rather than as a dorsal exposure of the palatine (see Damiani & Warren, 1996:290, for comments). *Almasaurus habbazi* was described in 1976 and would fall within the time range during which LEPs were neither well-known nor widely documented within Temnospondyli. There are numerous examples (primarily of dissorophoids) in which reexamination of historic specimens, including those (re)described well after the 1960s, has led to identification of a LEP (e.g., Bolt, 1974; Boy, 1995; Milner, 2018; Gee et al., 2019; Gee & Reisz, 2020). If *A. habbazi* actually does have a LEP, this might indicate affinities with *Chinlestegophis jenkinsi* and *Rileymillerus cosgriffi* but could alternatively imply collective affinities with another clade or different relation to caecilians; or as yet, another independent acquisition of a LEP within Temnospondyli cannot be excluded.

*Almasaurus habbazi* also shares certain features (most of which are plesiomorphies) with *Rileymillerus cosgriffi* that are not found in *Chinlestegophis jenkinsi*. These include a narrow, rod-like cultriform process and corresponding large interpterygoid vacuities; a proportionately long, triangular skull; posteriorly open otic notches; a laterally extensive postfrontal that frames the orbit anteriorly; a quadratojugal; and a tightly articulated supratemporal (most of these are plesiomorphies). All three share features like the relatively tall skull, the sloping occiput, and relatively small orbits. *Almasaurus habbazi* has not been redescribed since Dutuit (1976), and it is typically excluded from phylogenetic analyses despite being represented by several complete skulls and postcrania. Schoch (2008a) recovered it as a close relative of *R. cosgriffi* but within Trematosauria. That was informed by an interpretation of the putative LEP of *R. cosgriffi* as a short lacrimal like that of *A. habbazi,* although if the small element below the orbit of *R. cosgriffi* is a lacrimal, it is very different from *A. habbazi*. McHugh (2012) similarly recovered *A. habbazi* as a relative of the Metoposauridae within Trematosauria. The omission of this taxon from most previous analyses is not surprising, but its uncertain relationships and general marginalization (it is not mentioned by Pardo et al., 2017a) urge caution in the interpretation of small-bodied Mesozoic temnospondyls.

If *Almasaurus habbazi* possesses a lacrimal and lacks an LEP (and the original interpretation of *Rileymillerus cosgriffi* as lacking a lacrimal and possessing an LEP is correct), then it is probably unrelated to *Chinlestegophis jenkinsi* and *R. cosgriffi* and may be a diminutive trematosaur as Dutuit (1976) and Schoch (2008a) suggested and as was recently recovered by Gee et al. (2021) based on codes generated from Dutuit’s description. However, it would then represent an independently convergent form characterized by many of the features that these taxa share with caecilians that Pardo et al. (2017a) asserted as early transformations in caecilian evolution and therein casts doubt on the robusticity of their inferred homologies (e.g., relatively small, laterally facing orbits, occiput projecting far posteriorly and exposed dorsally, single row of coronoid teeth). Alternatively, the three extinct taxa may be closely related, but it cannot be assumed that *A. habbazi* would merely nest with *R. cosgriffi* and *C. jenkinsi* without changes to the topology with respect to other stereospondyls or to lissamphibians. Small-bodied stereospondyls remain enigmatic, but it seems probable that they merely are undersampled due to both size and ecology and could prove to be more diverse and widespread than presently recognized, especially in North America, as Pardo et al. (2017a) suggested. Additional recovery and study of such taxa might confer additional support that their morphology is merely convergent with caecilians on account of their fossorial ecology (as with recumbirostran ‘microsaurs,’ longstanding candidates for lissamphibian origins more recently considered to be crown amniotes; e.g., Pardo et al., 2017b, and derivates thereof) or alternatively might provide a more compelling narrative in the stepwise acquisition of caecilian features from stereospondyls.

**Comparisons of *Funcusvermis gilmorei* to *Chinlestegophis jenkinsi*.** The evidence for a dissorophoid origin of Gymnophionomorpha and a broader monophyletic origin of Lissamphibia from Dissorophoidea means that it can be assumed that the anatomy of *Funcusvermis gilmorei*, not that of *Chinlestegophis jenkinsi*, approximates the plesiomorphic conditions for Gymnophionomorpha. The differing interpretations for caecilian evolution based on which taxon is considered as the plesiomorphic condition are summarized below.

- *Formation of the maxillopalatine:* The coossification of the lacrimal to the maxilla in *Chinlestegophis jenkinsi* was interpreted as incipient formation of the gymnophionan maxillopalatine (Pardo et al., 2017a), but the external and internal morphology of the *Funcusvermis gilmorei* maxillopalatine (Fig. 1, Extended Data Fig. 4) shows that the nasolacrimal duct was likely not enclosed by the maxillopalatine in ancestral gymnophionomorphs, suggesting that its closure within the maxillopalatine occurred later in the common ancestor of gymnophionans. This warrants additional examination of temnospondyls using CT methods to assess the passage of the nasolacrimal duct in taxa with various lacrimal morphologies as well as those lacking lacrimals.
- *Orbit size reduction:* The small, laterally directed orbits present in *Chinlestegophis jenkinsi* and *Rileymillerus cosgriffi* were suggested as homologous to similar orbital size and position in *Eocaecilia micropodia* and gymnophionans, however the incompletely preserved orbital margin of *Funcusvermis gilmorei* suggests relatively large orbits, more comparable to those in dissorophoids. This suggests that the orbits in ancestral gymnophionomorphans were initially large, later reducing in size in the common ancestor of *E. micropodia* and gymnophionans.
- *Consolidation of lower jaw elements:* The lower jaw of *Chinlestegophis jenkinsi* contains all the bones present ancestrally in temnospondyls, differing from the condition in *Funcusvermis gilmorei* and all other gymnophionomorphs where the lower jaw is formed by the pseudoangular and pseudodentary.
- *Secondary tooth row in the lower jaw:* The coronoid series in *Chinlestegophis jenkinsi* bears a continuous row of non-pedicellate teeth which form a parallel row to the marginal dentition of the dentary; this was suggested to represent an incipient pseudodentary prior to coossification of the coronoid series to the dentary forming the double row of dentition typical of gymnophionomorphs. However, the mesially restricted lingual tooth row in *Funcusvermis gilmorei* and *Eocaecilia micropodia* differs from the distally extending lingual tooth row of the coronoid series in *C. jenkinsi*, more closely resembling the mesially restricted tooth row of *Doleserpeton annectens*. As detailed in Supplementary Information 2, the lingual tooth row of *D. annectens* (and many temnospondyls) is likely homologous to the adsymphyseal (=parasymphyseal plate). If *C. jenkinsi* is the sister taxon of Gymnophionomorpha, multiple transformations in the dentigerous bones of the lower jaw would be required, including loss of the coronoid series, mesial expansion of the lingual tooth row to the mandibular symphysis, and acquisition of pedicellate teeth in coronoid 1.
- *Bicuspid pedicellate marginal dentition:* The monocuspid non-pedicellate conical dentition of *Chinlestegophis jenkinsi* differs from the clearly pedicellate dentition of *Funcusvermis gilmorei* and all other gymnophionomorphs. Given their phylogenetic results, Pardo et al. (2017a) argued that pedicellate dentition in gymnophionomorphs must have been acquired separately in amphibamids and gymnophionomorphs. Indeed, as they note, *Gerobatrachus hottoni* has monocuspid teeth (Anderson et al., 2008b), despite nesting higher than amphibamids with bicuspid dentition like *Amphibamus grandiceps* and *Doleserpeton annectens* (Sigurdsen & Bolt, 2010). Their points are not repeated here, but the possibility that bicuspid dentition is only acquired at hormonal maturity (as in many extant lissamphibians, e.g., Davit-Béal et al., 2007) warrants future consideration because the holotype and only specimen of *G. hottoni* was interpreted as a juvenile by Anderson et al. (2008). Immaturity might explain the present discrepancies in character evolution. However, this will require additional data from extinct taxa, which places a premium on improving ontogenetic resolution of temnospondyls and other candidate clades for lissamphibian origins. A monophyletic Lissamphibia originating from within Dissorophoidea supports a single origin of bicuspid pedicellate dentition.
- *Other dentition:* Unlike the high tooth counts present in the labial and lingual tooth rows of *Funcusvermis gilmorei* and *Eocaecilia micropodia* (>40)*,* which are also shared with many amphibamiforms (e.g., Huttenlocker et al., 2007; Clack & Milner, 2010; Sigurdsen & Bolt, 2010; Anderson & Bolt, 2013; Maddin et al., 2013) *Chinlestegophis jenkinsi* preserves at least 13 marginal teeth in the dentary and likely no more than twice as many based on the estimated length of the complete dentary. A symphyseal tusk is likely present in *C. jenkinsi*, as with other stereospondyls, whereas no tusk is present in *F. gilmorei*.
- *Dermal sculpturing:* Dermal bones of the cranium, as well as the angular in *Chinlestegophis jenkinsi*, bear external sculpturing, differing from the unsculptured maxillopalatine, pseudodentary, and pseudoangular in *Funcusvermis gilmorei.*

The limited skeletal material referred to *Funcusvermis gilmorei* precludes comparison with other skull roof and braincase features present in *Chinlestegophis jenkinsi* that were suggested to be homologs to similar gymnophionomorph features (Pardo et al., 2017a), however the strong support for an amphibamiform origin of Gymnophionomorpha found herein suggests that these features are homoplastic rather than homologous. The discovery of skeletons of *C. jenkinsi* encased within burrows strongly supports a fossorial life-habit for this taxon, suggesting that its similarity to gymnophionans may be a result of skeletal convergence driven by adaptation for fossoriality. This is similar to the longstanding hypothesis of an origin of Lissamphibia from within ‘Lepospondyli’ that was driven by features now regarded as convergence due to a fossorial ecology (e.g., Pardo & Anderson, 2016; Pardo et al., 2017b).

*Chinlestegophis jenkinsi* was recovered from the Upper Triassic Chinle Formation (middle-late Norian; Revueltian Estimated Holochron) in Garfield County, Colorado, USA (Pardo et al., 2017a). *Chinlestegophis jenkinsi* is only slightly younger than the middle Norian-aged *Funcusvermis gilmorei* (Adamanian Estimated Holochron), and the present-day provenance of these two taxa are separated spatially by approximately 400 km. *Rileymillerus cosgriffi* was recovered from the Upper Triassic Dockum Group (middle-late Norian; Adamanian Estimated Holochron) in Garza County, Texas (Martz et al., 2012). The proximal spatiotemporal occurrence of these taxa is evidence that fossorial caecilian-like stereospondyls such as *C. jenkinsi* and *R. cosgriffi* and stem-caecilians with uncertain life habits (*Funcusvermis gilmorei*) were all members of the overall Late Triassic vertebrate assemblage of equatorial Pangaea (present day Southwestern North America).

**4. Revisions and additions to phylogenetic dataset**

**Motivations**. The phylogenetic dataset built upon herein is derived from the dataset published in Schoch et al. (2020). The Schoch et al. (2020) matrix was selected because it has been previously employed to test the relationships of early-diverging lissamphibians (such as *Funcusvermis gilmorei*) because of its comprehensive sampling of temnospondyls, including the putative stem caecilian *Chinlestegophis jenkinsi* and other stereospondyls (Pardo et al., 2017a), and appreciable sampling of extant lissamphibians. For these aforementioned reasons this dataset was chosen for testing the relationships of*F. gilmorei*, however, during our coding of *F. gilmorei*into the matrix of Schoch et al. (2020), we encountered a number of codings that are in need of modification based on examination of published literature. A number of these codings were traced back to previous versions of the matrix and have the potential to greatly influence the results of the phylogenetic analysis, whether because they occur in focal taxa or in characters cited as important in the origin of lissamphibians. To avoid introducing asymmetry into the matrix by only modifying select codings, we undertook a systematic survey and revision of the character/taxon matrix of Schoch et al. (2020), leading to many changes to characters and character codings which we document in detail below. As a result, our revision is now the most up-to-date version of the Schoch et al. (2020) dataset and could be used for both future temnospondyl and lissamphibian studies. Our phylogenetic results (see Methods) using this revised matrix are shown in Fig. 3 and Extended Data Figs. 5-7.

**Matrix history.** The matrix of Schoch et al. (2020) represents a relatively minimally modified version of the matrix employed by Pardo et al. (2017a), who made substantive character modifications to the matrix of Schoch (2013), which was originally constructed to test the interrelationships of temnospondyls without consideration of lissamphibian origins. The Schoch (2013) matrix in turn represents the collation of smaller-scale analyses of various temnospondyl clades and sampled 70 temnospondyls (although eight were excluded from the final analysis for which results are discussed and figured in detail). Pardo et al. (2017a) excluded several other temnospondyls (*Thabanachuia oomie, Tupilakosaurus wetlugensis, Branchierpeton amblystomum, Branchiosaurus salamandroides, Watsonisuchus gunganji*, *Wetlugasaurus angustifrons, Eryosuchus garjainovi*, *Eocyclotosaurus wellesi*) but also sampled several of the taxa excluded by Schoch (e.g., *Lapillopsis nana*, *Peltobatrachus pustulatus*) and added *Chinlestegophis jenkinsi* and *Rileymillerus cosgriffi* to produce a total of 65 temnospondyls.

All temnospondyls other than *Chinlestegophis jenkinsi* and *Rileymillerus cosgriffi* were originally coded for characters 1–212 by Schoch (2013), for characters 213–345 by Pardo et al. (2017a), and for characters 346–360 by Schoch et al. (2020). It should be noted that although Pardo et al. did not mention or document any coding modifications, they incorporated most of the changes made by Dilkes (2015) to the original matrix of Schoch (2013), as noted by Marjanović & Laurin (2019). The matrix of Pardo et al. (2017a) therefore is not a first-order derivate of Schoch (2013), with modification, but rather a second-order derivate via Dilkes (2015), with modification. *Chinlestegophis jenkinsi* and *Rileymillerus cosgriffi* were coded for characters 1–345 by Pardo et al. (2017a) and for characters 346–360 by Schoch et al. (2020). The ‘lepospondyls’ *Rhynchonkos stovalli*, *Batropetes fritschi*, and *Brachydectes elongatus* were coded for characters 1–360 by Schoch et al. (2020). All lissamphibians were coded for characters 1–345 by Pardo et al. (2017a) and for characters 346–360 by Schoch et al. (2020). The albanerpetontid *Celtedens ibericus* (which was constructed as a chimeric operational taxonomic unit (OTU) per the supplementary information of Schoch et al., 2020) and the putative stem urodele *Triassurus sixtelae* were coded for characters 1–360 by Schoch et al. (2020). Changes to previous codes were noted by Schoch et al. (2020) in their supporting information. Note that there are 18 temnospondyls sampled by Schoch (2013) and Pardo et al. (2017a) that were excluded from Schoch et al. (2020).

**Recoding philosophy and methods.** Codes of certain taxa in the matrix of Schoch et al. (2020) cannot be replicated from the literature alone (i.e., they are either unsubstantiated or are predicated entirely on personal observations). This typically applies to taxa with historical descriptions, such as *Cyclotosaurus robustus, Metoposaurus diagnosticus*, and *Uranocentrodon senekalensis*, for which more recent published data may consist only of photographs of select skeletal regions or only of reconstructions. We applied a conservative approach to modification of the matrix; besides codings expressly based on personal observations, we applied the highest evidentiary standard (“beyond a reasonable doubt”) in assessing codes. Codes were adjusted only if the literature expressly contradicted the previous score, either by documenting a different condition or by indicating that a given skeletal region is unknown in a given taxon. If a feature or condition was not clearly documented in the literature but was previously coded, we kept that coding provided that the literature established that the general region is known for the taxon. An example is given below.

The trematopid *Phonerpeton pricei* was coded for five characters related to the ilium (198–202). The original (and only) descriptions of this taxon by Olson (1941:153–155) and Dilkes (1990:234–237) make no mention of the ilium, nor do they provide a description or a figure. This taxon is coded identically to other olsoniform dissorophoids in which the ilium is definitively known for these five characters, and it could be reasonably inferred (though not coded on this basis) that *Phonerpeton pricei* had the same apomorphic conditions of the olsoniform ilium. However, because Dilkes (1990:224) lists one specimen as consisting of “fragments of pectoral and pelvic girdles,” we did not change these codes because we are unable to conclude ‘beyond a reasonable doubt’ from the literature that the ilium is wholly unknown. This is not the most conservative approach from the perspective of *coding* (which would be to code it as unknown for these characters), but it is the most conservative approach from the perspective of *recoding* (which gives the benefit of the doubt to previous workers if the literature does not unequivocally contradict the codes). We opted for this approach to make our results as comparable as possible to previous iterations of this matrix, but there are a substantial number of unmodified codes that exist in this ‘gray area’ of being neither confidently substantiated nor invalidated by the literature, which will need to be addressed going forward. As a result of this standard, we were more likely to reexamine and to recode characters with distinct states (e.g., those with counts or presence/absence binaries) rather than those that are more ambiguous or undoubtedly subjective to a degree (e.g., ‘long’ vs. ‘short’).

The same burden of evidence was applied when considering whether to code previously uncoded cells. For example, the dendrerpetid *Dendrerpeton helogenes* (taxonomy following Arbez et al., 2022) was coded as unknown for dentigerous palatal ossicles (character 139), but Godfrey et al. (1987:800, fig. 1D) identified numerous small plates between the mandibles. They called these ‘gular’ osteoderms (character 149) but compared them to similar features in the holotype of *Platyrhinops lyelli* that were identified as by Carroll (1964:231) as “denticulate skin covering” and as dentigerous ossicles by Clack & Milner (2010:289), and the position is more in line with palatal plates rather than with gular osteoderms. On one hand, Godfrey et al. (1987) did not identify ornamentation on these plates that would provide positive evidence that they are osteoderms, but they also did not identify denticles that would provide positive evidence of a palatal or mandibular feature. Consequently, we did not code character 149 for this taxon, but other workers may consider a lower evidentiary standard (e.g., ‘more likely than not’) to be sufficient to code this. This approach applies also to entire characters that may require a high fidelity of preservational quality. For example, a narial flange is considered to be a dissorophoid synapomorphy and is coded as absent in all other sampled temnospondyls, even though most taxa do not appear to be preserved or prepared in a fashion that would actually permit assessment of this intranarial flange (e.g., in dorsoventrally compressed specimens).

We treated characters literally. For example, a number of characters actually capture multiple features, presumably because they frequently co-occur. An example is character 129 (palatine, ectopterygoid [width]). This is greatly reduced in many amphibamiforms (129–1), which is qualitatively cited as evidence for lissamphibian affinities. However, the prescription of the character clearly specifies both the palatine and the ectopterygoid, not one or the other. Therefore, any taxon without a palatine or an ectopterygoid (or both) cannot be coded for this character. Other examples include character 50 (postfrontal and postorbital either shorter than the supratemporal and the parietal or longer) and character 148 (anterior and middle coronoid dentition present or absent). This approach is superseded only by philosophical problems (e.g., non-homology, see ‘Terminology’ below).

In line with our conservative recoding approach, we also did not modify characters unless we felt that doing so was essential for avoiding redundancies or excessive dependencies or clearly failed to capture the full range of variation. For example, character 158 (presacral count) originally had three states: more than 28; 23–25; and fewer than 21. This character does not capture taxa with presacral counts of 21, 22, 26, 27, or 28 positions (which occur in some sampled taxa, e.g., *Callistomordax kugleri*, *Paracyclotosaurus davidi*; Watson, 1958; Schoch, 2008b), and the addition of a fourth character state for the further reduction observed in batrachians by Schoch et al. (2020) does not resolve this. This character was thus modified here to fully encompass all possible presacral counts. However, we did not attempt to modify characters with ambiguous qualitative distinctions (e.g., character 307, femur ‘long’ vs. ‘short’) to minimize the number of necessary recodings.

With the exception of personal observations of *Eocaecilia micropodia* made by several of the authors, recoding is restricted to data available from the literature.

**Terminology.** For brevity in the sections below and to remain consistent and transparent with respect to all of the changes we made to the matrix, we outline how specific terms are employed with respect to the coding changes that we made. The following terms are made with respect to the **original state coded.** Character numbering in this section refers to the original numbering by Schoch et al. (2020) regardless of the omission of several from the dataset used in our analyses (Characters 14, 78, 81, 115, 232, 253, 352); the new numbering scheme is detailed below in Supplementary Information 8.

- An **unsubstantiated code** is classified as one where the original coding was for a specific state (i.e., not unknown or inapplicable) and is in need of modification; the following terms define the three primary types of unsubstantiated codes.
  - A **mischaracterized code** is when the taxon was coded for one state but should be coded for another character state. An example is the original coding of the zatracheids *Acanthostomatops vorax* and *Zatrachys serratus* as having a prefrontal and postfrontal separated by the frontal (42–1). In fact, these taxa have a wide prefrontal-postfrontal contact (42–0, the plesiomorphic condition; e.g., Langston, 1953; Schoch, 1997; Witzmann & Schoch, 2006; Urban & Berman, 2007).
  - An **overgeneralized code** is when the taxon was coded for one state but should be coded for two or more as part of a polymorphism. In the absence of any previously specified protocol regarding non-ontogenetic intraspecific variation in this matrix, polymorphisms were coded across all scales within a species (between populations, within populations, within individuals), and no consideration is given to the relative frequency of either a polymorphism or of a specific state.
  - An **untestable code** is when the taxon cannot be coded for the character because the characterized feature is unknown in that taxon. An example is the original coding of the rhytidosteid *Sangaia lavina* as having four manual digits (197–1). However, as noted in the original description by Dias-da-Silva et al. (2006:382), there are only two specimens, one of which is a left half of the skull and the other of which is a palatal fragment. Two specimens previously published as Rhytidosteidae indet. (Dias-da-Silva, 2005) and subsequently listed as referred material (Dias-da-Silva & Marsicano, 2011) also do not consist of any postcranial material. No new material of this taxon has been published since then. Therefore, *S. lavina* cannot be coded for any postcranial features, let alone one that requires a high quality of preservation to assess like digit count. Untestable states are recoded as unknown (?) unless they are related to a dependency (see next point) and are termed as much because there is no way for the character to be reliably coded at present.
  - An **inapplicable code** is when the taxon should not be coded for a character because it is dependent on the presence of a feature that is known to be absent. Dependencies are extensively discussed in the literature (e.g., Maddison’s 1993 example of two characters: presence/absence of a tail and tail color; see also Strong & Lipscombe, 1993; Brazeau, 2011; Simões et al., 2017). There are two types of dependencies, although individual characters are not identified as such.
    - The first is a violation of two basic tenets of character sampling: independence and homology. For example, if the ectopterygoid is absent (346–1), it is technically true that ectopterygoid fangs are absent (88­–1); that the palatine ramus is only formed by the pterygoid (123–0) rather than by both the pterygoid and the ectopterygoid; and that a lateral exposure of the ectopterygoid is absent (132–0). Some of these binary characters are specifically stated in ‘presence/absence’ language, while others merely express it in a different fashion. For example, character 124, with states ‘pterygoid contacts only the palatine’ versus ‘pterygoid-contacts palatine and ectopterygoid,’ could alternatively be expressed as ‘pterygoid-ectopterygoid contact absent’ versus ‘pterygoid-ectopterygoid contact present.’ They all correspond to the same primary character (ectopterygoid), and their secondary absences are all intrinsically linked to the same primary condition (absence of the ectopterygoid). These characters are therefore non-independent and coding secondary characters like these will overweight this absence. In the example here, *Doleserpeton* has traditionally been coded as lacking an ectopterygoid (although this is equivocal at best based on the literature, see below), a feature shared with lissamphibians that is not found in any other temnospondyl. Considering that there are 10 secondary characters related to the ectopterygoid, it should be considered that this absence has overweighted this inferred homology in the debate over lissamphibian origins under the presumption that it lies within Temnospondyli. While the ‘intent’ of employing these secondary characters is not expressly stated, presumably it is intended as a means of differentiating between the subset of taxa that all have an ectopterygoid. Therefore, coding taxa without an ectopterygoid for these secondary presence/absence characters will obfuscate differentiation of taxa because there is a false equivalency between absence due to absence of ectopterygoid (a consequence) and absence in the presence of ectopterygoid (a condition).
    - The second type of dependency is when a dependent secondary character cannot be coded under any interpretation because it does not express presence/absence but rather characterizes a feature assumed to be present. Presence/absence may be explicitly defined in another character but does not need to be for a dependency to exist. An example in this matrix is character 211 (osteoderms, width), which has two states: ‘narrow median osteoderms’ or ‘transversely extended plates.’ Any taxon without any osteoderms (209–0), as is the condition of most temnospondyls, cannot be coded for character 211, as osteoderms do not exist. However, most taxa without osteoderms were consistently and originally coded for 211–0 (‘narrow’).
- An **omission** occurs when the original coding was listed as unknown (?), but available published literature allows for coding of the character. An example is the original coding of the trematopids *Acheloma cumminsi* and *Phonerpeton pricei* as being unknown for lacrimal-orbit contact (220). However, the lacrimal enters the orbit in these taxa (220–1), as in most dissorophoids and is well-documented (e.g., Olson, 1941; Dilkes & Reisz, 1987; Dilkes, 1990, 1993; Polley & Reisz, 2011). In the specific context of this study, we only coded these characters if the literature is sufficient to confidently do so; there may therefore be some missing data that other workers would be able to rectify through personal observation, access to relatively inaccessible publications (e.g., obscure journals, print-only materials).

The following terms are made with respect to the changes implemented by us to the original code:

- A **correction** is a coding change made based on the identification of an unsubstantiated code. All types of unsubstantiated codes are considered to be in need of correction.
  - A **correction to unknown** is a specific kind of correction where a previously coded cell is changed to unknown (?).
- A **recoding** is a coding change made based on a modification to a character, such as the addition of a new character state, or a coding change made based on a modification to a taxon, such as the restriction of the former composite OTU of *Cacops aspidephorus* + *Cacops morrisi* to strictly *Cacops aspidephorus*.
- A **change to inapplicable** refers specifically a change from ‘?’ to ‘-‘. This has no significance from an algorithmic perspective, as inapplicable cells are treated as missing data, but as with Pardo et al. (2017a), we prefer to explicitly express inapplicable cells as such. Our motivation here is for future use of this matrix; when inapplicable cells are left as unknown, the presence of this code cannot differentiate between a cell that was accidentally missed (an omission), a cell that was intentionally left as ‘?’ because the feature is unknown (missing data), and a cell that was intentionally left as ‘?’ because the character is inapplicable to the taxon (‘coded’).

**Comments on taxa.** The following section details relevant comments on the treatment of certain taxa sampled in our analysis. Character numbering in this section refers to the original numbering of Schoch et al. (2020) regardless of the omission of several characters from the dataset used in our analyses (Characters 14, 78, 81, 115, 232, 253, 352); the new numbering scheme is detailed below in Supplementary Information 8.

- ***Brachydectes elongatus:*** This taxon has been considered to be a junior synonym of *Brachydectes newberryi* by more recent ‘lepospondyl’ workers (Pardo & Anderson, 2016; Mann, 2018), a revision of its assignment by Wellstead (1991; the single source used by Schoch et al., 2020 to code this OTU). This more inclusive concept has little impact on existing codings of this OTU.
  - The following characters were newly coded based on Pardo & Anderson (2016): 39 (0), 46 (0), 60 (1), 76 (0), 222 (1), 247 (0), 255 (0), 256 (1), 264 (2), 271 (2), 272 (1), 273 (-), 290 (1), 291 (1), 293 (0), 336 (0), 338 (0), 339 (0), 340 (0).
  - Other changes are listed in the taxon-specific section and represent mischaracterized codes or omissions that need to be coded regardless of which species is sampled (e.g., coding an otic notch as present in this OTU; 51–0).
- ***Batropetes fritschi:*** The skull roof, palate, and braincase of *Batropetes fritschi* is poorly known, and therefore we elected to exchange *Batropetes fritschi* for a better-known member of the same genus, *Batropetes palatinus* (Carroll, 1991; Glienke, 2015), in order to maximize the scorable characters.
  - The following characters were newly coded based on the taxonomic shift for this OTU: 81 (-), 178 (1), 181 (-), 182 (1), 256 (1), 264 (1), 265 (0), 266 (2), 267 (-), 271 (3), 272 (-), 273 (-), 290 (1), 294 (0), 295 (0), 297 (0), 298 (0), 299 (1), 300 (2), 309 (1), 310 (-), 339 (0), 351 (0).
  - The following characters were recoded based on this taxonomic shift: 87 (1 🡪 0), 91 (0 🡪 -), 92 (0 🡪 -), 99 (0 🡪 ?), 101 (0 🡪 ?), 103 (0 🡪 1), 105 (0 🡪 ?), 110 (0 🡪 2), 138 (0 🡪 ?), 145 (0 🡪 -), 147 (0 🡪 -), 148 (0 🡪 -), 222 (0 🡪 1), 226 (0 🡪 1), 252 (0 🡪 ?), 259 (0 🡪 ?), 274 (0 🡪 ?), 325 (1 🡪 ?), 349 (0 🡪 ?), 353 (0 🡪 ?). Note that most of these probably represent unsubstantiated codes in the original coding of *Batropetes fritschi*, as they relate to palatal features that are not described, figured, or reconstructed by Glienke (2013), such as parasphenoidal dentition or choanal morphology, and that are unknown in this taxon based on the literature. Many of these codes may have been taken from *Batropetes niederkirchensis*, also described by Glienke (2013).
  - Other changes are listed in the taxon-specific section and represent mischaracterized codes or omissions that should have been coded previously regardless of which species is sampled (e.g., coding an otic notch as present in this OTU; 51–0).
- ***Rhynchonkos stovalli*:** Szostakiwskyj et al. (2015) demonstrated that the suite of material referred to this taxon by Carroll & Gaskill (1978), the source of codings by Schoch et al. (2020), represents a chimera of at least three taxa. Most of the original characterization by Carroll & Gaskill (1978) is based on a composite of specimens without skeletal overlap, which as Szostakiwskyj et al. (2015:3) noted, requires an assumption that this is not “an amalgam of caecilian-like characteristics drawn from several distinct taxa into an artificially caecilian-like form.” The codings are restricted to the holotype (a skull with lower jaws), which was redescribed by Szostakiwskyj et al. on the basis of new CT data.
  - The following characters were automatically recoded as unknown based on restriction to the holotype: 69, 99, 158­–162, 167–177, 179, 183–212, 214, 222, 278–284, 286, 288–289, 291–292, 296, 301–308, 325, 345, 347, 348.
  - The following characters were newly coded based on Szostakiwskyj et al. (2015): 19 (0), 76 (0), 247 (0), 256 (1), 264 (2), 266 (1), 267 (1), 271 (1), 272 (0), 273 (2), 336 (0), 338 (0), 339 (0), 340 (0).
- ***Dendrerpeton helogenes:*** The OTU long called ‘*Dendrerpeton acadianum*’ in temnospondyl analyses that derives from material described by Godfrey et al. (1987) and Holmes et al. (1998) is in fact *Dendrysekos helogenes* sensu Schoch & Milner (2014:25), which means that it is *Dendry. helogenes* and not *Dendre. acadianum* that has served as a common outgroup in temnospondyl analyses. Codings are restricted to the holotype (NHMUK 4149, a partial skull roof exposed ventrally; Steen, 1934) and to the two specimens described by Godfrey et al. (1987) and Holmes et al. (1998). A species concept restricted to these three specimens aligns with the *Dendrerpeton helogenes* of Arbez et al. (2022), who recently synonymized *Dendrerpeton* and *Dendrysekos.* Neither of these recent taxonomic acts would lead to any coding changes, although it should be questioned whether occipital features can be coded from these specimens (it is unclear from the literature) or if these could only be coded from what is now a taxonomically ambiguous specimen like the occiput described by Robinson et al. (2005) on the basis of CT data (the occiput does not contain any differential characters sensu Schoch & Milner, 2014).
- ***Cacops aspidephorus:*** This has always been a chimeric OTU representing a combination of *Cacops aspidephorus* and *Cacops morrisi* (but not *Cacops woehri*), as noted by Schoch (2013:table 1). The inferred rationale is that the cranial osteology of *Cacops aspidephorus* was essentially unknown until recently (Anderson et al., 2020), but the postcranial skeleton was entirely known (Williston, 1910; Dilkes & Brown, 2007; Dilkes, 2009, 2015), whereas the cranial osteology of *Cacops morrisi* was entirely known (Reisz et al., 2009), but the postcranial skeleton was almost entirely unknown until recently (Gee & Reisz, 2018). For this reason, the OTU has sometimes been displayed as ‘*Cacops aspidephorus morrisi*’ (e.g., Pardo et al., 2017a). No other taxon was coded from two or more species or as a terminal of a higher order clade, and now that *Cacops aspidephorus* can be more completely coded, we prefer to restrict the OTU to this single species, as there are differences between them that would be captured in phylogenetic codes (Anderson et al., 2020; Gee, 2021). Corrections are listed in the taxon-specific section and represent changes made to codes that were mischaracterized regardless of which species is sampled (e.g., coding the absence of orbital rims in this OTU; 27–0).
  - The following characters were recoded from one state to another: 87 (1 🡪 0).
  - The following characters were recoded from one state to unknown (?): 102, 104, 126.
  - The following characters were newly coded: 270 (0), 271 (0), 275 (1), 310 (1), 311 (1), 312 (0), 313 (0), 319 (-), 321 (0), 322 (1), 323 (0), 324 (0), 326 (1).
- ***Phonerpeton pricei:*** *Phonerpeton pricei*, as coded by Schoch (2013), was characterized following the concept of this taxon of Dilkes (1990) and not that of Schoch & Milner (2014) in which some of this material was considered to belong to a separate species of *Phonerpeton*, *Phonerpeton whitei*. Dilkes’ concept is followed here.
- ***Acheloma cumminsi:*** This taxon follows the concept of Gee (2020), in which material of ‘*Acheloma dunni*’ from the Richards Spur locality is considered to be synonymous with *Acheloma cumminsi*. Schoch (2013:table 1) noted personal observation of material from the AMNH (where only material of *Acheloma cumminsi* sensu Polley & Reisz, 2011, is held), but Schoch only cited Polley & Reisz (2011), who described material of ‘*Acheloma dunni*’ reposited at the BMRP and OMNH, as the literary source. The only difference between these two species was the purported absence of a LEP in *A. cumminsi* (e.g., Dilkes & Reisz, 1987; Polley & Reisz, 2011), which was subsequently identified in the holotype of this taxon (Gee et al., 2019). This OTU was originally coded as having a LEP. It is thus unclear whether this OTU may have originally been a chimeric OTU of two species like with *Cacops*.
- ***Sclerocephalus haeuseri*:** This taxon is complicated by the noted intraspecific variation within and between different stratigraphically separated lake populations (Schoch & Witzmann, 2009a). This variation reflects ecological differences, with some populations remaining aquatic and retaining features like a lateral line system, whereas others were terrestrial and have correspondingly robust postcrania (Schoch, 2009). Some of these differences are captured in phylogenetic characters (e.g., lateral line sulci). Schoch (2013:table 1) listed Schoch & Witzmann (2009a) as the only literary source of data for this taxon, and the latter specifically focused on characterizing the Lake Jeckenbach population (L–O 6; see p. 137, 145 therein), from which the largest sample of adults is known. We therefore interpret the existing states for this taxon to be exclusively for this population and do not expand the coding here as part of our conservative approach to minimizing coding changes.
- ***Batrachosuchus browni***: The single brachyopid sampled by Schoch (2013:table 1) was listed as *Batrachosuchus browni*, but the examined material refers to specimen(s) in the Natural History Museum London (NHMUK); there is only one specimen of *Batrachosuchus browni*, and it is reposited in the South African Museum (SAM). Conversely, *Bathignathus watsoni*, originally placed in *Batrachosuchus* until Damiani & Jeannot (2002), is known from one specimen reposited at the NHMUK, and the OTU was listed in the matrix as ‘*Batrachosuchus watsoni*’. The listed reference, Watson (1956), does not resolve this issue, as both species were redescribed in that work (though *Batr. browni* in much less detail). Both historic and recent diagnoses of these taxa (e.g., Welles & Estes, 1969:22–23; Damiani & Jeannot, 2002:61; Schoch & Milner, 2014:98) encompass differences that are not captured by the character sample (e.g., the presence of an interfrontal in *Batr. browni*). Because *Batr. browni* was more recently described and figured (Chernin, 1977) than *Bath. watsoni* (reassigned but not redescribed by Damiani & Jeannot, and last redescribed by Watson, 1956), we opted to use *Batr. browni* (which is also represented by slightly more complete material).
- ***Siderops kehli:*** The skull of *Siderops kehli*, despite being largely complete, is poorly known with respect to the skull roof. Virtually all of the cranial roof sutures are reconstructed (Warren & Hutchinson, 1983:fig. 2), and there is only one known specimen. The poor understanding of its cranial osteology is evidenced by diagnoses that rely primarily on qualitative cranial or postcranial features and an absence of propagated reconstructions compared to less reconstructed taxa (e.g., Schoch & Milner, 2000; Warren & Marsicano, 2000). A reconstruction might be acceptable for coding if it were formed as a composite from multiple specimens. However, because there is only one specimen, the reconstruction of sutures is not really that of *S. kehli* but rather of the concept of a generalized chigutisaurid skull roof in the 1980s; what sutures are known (e.g., paired frontals) are predominantly temnospondyl plesiomorphies. This means that there is no justifiable basis for coding based on the reconstruction compared to other taxa for which a composite from multiple specimens can be made.
- ***Gerrothorax pulcherrimus, Plagiosuchus pustuliferus*:** Among the notable features of plagiosaurids is the single centrum, with intersegmental neural arches. Compared to other taxa sampled in this family of matrices, the homology of the centrum is the most uncertain. Whether this centrum represents co-ossification of the pleurocentra and the intercentrum (Shishkin, 1987, 1989); an enlarged pleurocentrum and the loss of the intercentrum (Panchen, 1959); or an enlarged intercentrum with loss or fusion of the pleurocentra with the neural arch (Warren & Snell, 1991; Hellrung, 2003) remains unresolved (e.g., Witzmann et al., 2014; Danto et al., 2016, 2017). The first two conditions would be apomorphic among temnospondyls, whereas metoposaurids and perhaps *Laidleria gracilis* share large, dorsally closed intercentra in the absence of pleurocentra. There is further variability within the clade (e.g., Konietzko-Meier et al., 2014, suggested different development between *Gerrothorax* and *Plagiosuchus*). Original codes by Schoch (2013) for characters 163–170 are based on the hypothesis that the centra are strictly intercentra (i.e., the pleurocentra are coded as absent), but because the homology remains an open question, all characters related to specifically intercentra or pleurocentra (versus a generic ‘centra’) should not be coded.
  - Characters 163–168 and 170 were coded as unknown (?) for both taxa.
  - Characters 278, 287 were coded as unknown (?) for *Gerrothorax*.
- ***Trematosaurus brauni:*** The osteology of *Trematosaurus brauni* was outdated prior to Schoch’s (2019) revised study, so most characters added by Pardo et al. (2017a) were probably not coded for this taxon because the existing literature at the time was insufficient to do so. Following Schoch’s publication, many of these can now be coded.
  - The following characters were newly coded: 216 (0), 217 (0), 218 (0), 219 (1), 220 (0), 222 (0), 223 (0), 224 (0), 225 (1), 226 (0), 227 (0), 228 (0), 229 (0), 230 (0), 231 (0), 233 (0), 234 (0), 235 (0), 236 (0), 237 (0), 238 (0), 239 (0), 240 (0), 244 (0), 245 (0), 246 (0), 248 (0), 250 (0), 251 (1), 252 (1), 254 (0), 255 (1), 256 (0), 257 (1), 258 (1), 259 (0), 260 (0), 262 (0), 264 (0), 265 (0), 268 (0).
- ***Metoposaurus diagnosticus:*** *Metoposaurus diagnosticus* originally comprised two subspecies, *Metoposaurus diagnosticus diagnosticus* and *Metoposaurus diagnosticus krasiejowensis* (Sulej, 2002). This taxonomic framework was utilized until Brusatte et al. (2015) elevated the latter to species rank (*Metoposaurus krasiejowensis*) on the basis of their opinion that subspecies are not appropriate for extinct vertebrates, a framework subsequently adopted by other workers. Schoch (2013:table 1) indicated that the osteology of *Metoposaurus diagnosticus* was based solely on a combination of older literature (Fraas, 1889, which described only *M. diagnosticus diagnosticus* in Sulej’s framework) and personal observation, which are collectively prohibitive to coding by other workers (evidenced by the last amount of missing data for characters added by Pardo et al., 2017a). We changed this OTU to *Metoposaurus krasiejowensis* because it is more recently and comprehensively documented for the entire skeleton (e.g., Sulej, 2002, 2007). At present, there are no differences between these species that are captured in the sampled characters other than polymorphisms only known in *M. krasiejowensis*, and this change is made with the intent to make the coding more reproducible.
  - Characters recoded as a result of this taxonomic shift (most of which are newly coded polymorphisms for previous overgeneralized states): 20 (0 🡪 0&1), 24 (0 🡪 1), 39 (0 🡪 0&1), 40 (0 🡪 0&1), 44 (0 🡪 0&1), 45 (0 🡪 0&1), 58 (0 🡪 0&1), 141 (0 🡪 ?), 145 (2 🡪 1), 146 (1 🡪 0&1), 220 (1 🡪 0&1).
  - Characters newly coded as a result of this taxonomic shift: 214 (0), 255 (1), 257 (1), 260 (0), 261 (0), 262 (0), 263 (1), 264 (0), 265 (0), 266 (0), 268 (0), 269 (1), 270 (1), 271 (0), 272 (1), 273 (-), 274 (0), 275 (0), 276 (1), 278 (0), 279 (0), 280 (0), 281 (0), 282 (0), 283 (0), 284 (1), 285 (0), 286 (0), 287 (0), 288 (1), 289 (1), 290 (2), 291 (2), 292 (2), 296 (1), 299 (0), 300 (-), 301 (1), 302 (1), 305 (0), 306 (-), 307 (0), 313 (0), 314 (0), 315 (0), 316 (1), 317 (0), 318 (0), 321 (0), 322 (1), 323 (0), 324 (-), 326 (1), 327 (0), 328 (-), 329 (-), 330 (-), 332 (-), 334 (0), 335 (-), 341 (-), 342 (-), 343 (2), 344 (0).
- ***Cyclotosaurus robustus***: Like *Metoposaurus diagnosticus*, this taxon was almost entirely uncoded for characters added by Pardo et al. (2017a), which reflects the fact that the osteology of this taxon has only been depicted in simple photographs (e.g., Schoch & Milner, 2000:pl. 16) and reconstructions (e.g., Schoch & Milner, 2000:101; Schoch, 2008a:fig. 4D) in recent literature. However, Schoch (2013:table 1) cited Sulej & Majer (2005) as the literature source for this taxon. Sulej & Majer described a new species of *Cyclotosaurus* (*C. intermedius*), which despite some contention that it is synonymous with *Cyclotosaurus robustus* (Lucas et al., 2007), is widely accepted as a distinct taxon by temnospondyl workers (e.g., Schoch, 2008a; Witzmann et al., 2016; Marzola et al., 2017). Schoch (2013) also listed personal observation of material at Stuttgart (SMNS), but material of *Cyclotosaurus intermedius* is reposited at the Institute of Paleobiology in Warsaw (ZPAL). Therefore, the OTU is either chimeric (*Cyclotosaurus robustus* + *Cyclotosaurus intermedius*) and has not been stated as such, or one of these sources is in error. Because documentation of *Cyclotosaurus robustus* is poor, we restrict this OTU to *Cyclotosaurus intermedius.*
  - Characters recoded from one state to another as a result of this taxonomic shift: 71 (1 🡪 0), 81 (1 🡪 0), 193 (1 🡪 0).
  - Characters recoded from one state to unknown as a result of this taxonomic shift: 150–153, 160–170, 174, 202, 208, 212.
  - Characters newly coded as a result of this taxonomic shift: 175 (0), 176 (0), 177 (0), 178 (0), 213 (0), 215 (0), 216 (0), 217 (0), 218 (0), 219 (1), 220 (0), 222 (0), 223 (0), 224 (1), 225 (1), 226 (0), 227 (0), 228 (0), 229 (0), 230 (0), 231 (0), 233 (0), 234 (0), 235 (0), 236 (0), 237 (0), 238 (0), 239 (0), 240 (0), 241 (1), 242 (0), 243 (0), 244 (0), 245 (0), 246 (0), 248 (0), 249 (0), 250 (0), 251 (0), 252 (0), 254 (0), 258 (1), 259 (0), 260 (0), 261 (0), 262 (0), 263 (1), 264 (0), 265 (0), 268 (0), 269 (0), 270 (0), 272 (1), 273 (-), 275 (0), 299 (0), 301 (0), 302 (0), 310 (0), 311 (1), 312 (1), 313 (0), 314 (0), 315 (0), 316 (1), 317 (0), 318 (0), 319 (-), 321 (0), 323 (0), 324 (0), 326 (1), 334 (0), 335 (-), 343 (0), 344 (0).
- ***Rileymillerus cosgriffi*:** As noted in Supplementary Information 3, we question the validity of the original interpretation of the nasal in this taxon by Bolt & Chatterjee (2000), as it would invoke an aberrant morphology in which the nasal extends along the lateral edge of the frontal. This is not observed in any other taxon regardless of the presence or absence of either an LEP or the lacrimal. Instead, the partial ‘nasal’ is in the same position as the lacrimal of *Almasaurus habbazi* (Dutuit, 1976), among some other later-diverging stereospondyls. Because we do not have positive evidence that the ‘nasal’ is a lacrimal, we opted to recode characters related to the nasal as unknown but did not code previously unknown characters for the lacrimal or restore the presence of a lacrimal. This had a minimal influence because it only led to the recoding two characters (20, 38) to unknown (?); most nasal characters require the entire element to be known.
- ***Hynobius japonicus:*** There is no such species within *Hynobius*, although there is a hynobiid with the same species epithet, *Onychodactylus japonicus.* Based on Pardo et al.’s (2017a) analysis, the OTU labeled as ‘*Hynobius japonicus*’ was sourced from Maddin et al. (2012:2), who coded an unspecified species of *Hynobius* from personal observations of one specimen (in the earlier study, it was coded as a terminal “salamanders” OTU). We treated this OTU as a genus-level OTU for *Hynobius*.

**Partial uncertainty.** In PAUP* and Mesquite, partial uncertainty is coded with the use of a slash (/), and this is algorithmically differentiated from polymorphism (indicated with ‘&’). TNT scripts cannot accommodate partial uncertainty, and these codings are converted to a polymorphism encompassing the possible states (e.g., partial uncertainty of states 0 and 1 becomes a polymorphism of 0 and 1) when Mesquite exports a TNT script. Pardo et al. (2017a), who analyzed the matrix in PAUP*, included several partial uncertainties, although most of these may have been intended as polymorphisms because they are largely for binary characters (except characters 248 and 321). We did not adjust codes to differentiate between intended partial uncertainty and intended polymorphism because our analysis was conducted in TNT. Because these cells are visually distinctive using Mesquite’s display option to color cells by character state, we noticed that none of these appear in the matrix of Schoch et al. (2020), in which the corresponding cells were coded as unknown. However, Schoch et al. (2020) did not note changing these codings, and it may be an error in translation between software or scripts (their analysis was conducted in TNT). Note that none of these cells were present in Schoch (2013) or Dilkes (2015) and therefore originated in Pardo et al. (2017a). The following codings are restored from Pardo et al.:

- **Character 10:** restored to ‘0/1’ from ‘?’ for *Ichthyophis* and *Epicrionops*.
- **Character 17:** restored to ‘0/1’ from ‘?’ for *Hynobius*.
- **Character 106:** restored to ‘0/1’ from ‘0’ for *Eocaecilia*.
- **Character 160:** restored to ‘0/1’ from ‘?’ for *Triadobatrachus*.
- **Character 246:** restored to ‘0/1’ from ‘?’ for *Cacops*.
- **Character 248:** restored to ‘1/2’ from ‘?’ for *Xenopus*.
- **Character 253:** originally coded as ‘0/1’ by Pardo et al. and changed here to ‘0&1’ for *Gerrothorax* (also noted in the taxon-specific changes).
- **Character 267:** restored to ‘0/1’ from ‘?’ for *Cochleosaurus*, *Lydekkerina*, *Siderops*, and *Callistomordax*.
- **Character 321:** restored to ‘0/1’ from ‘?’ for *Chinlestegophis*.
- **Character 324:** restored to ‘0/1’ from ‘?’ for *Eryops* and *Gerrothorax*.

**Unspecified character states.** A number of multistate characters occur in the matrix where the numerically highest state is not listed in the character list of previous studies. As may be noted, all of these characters are characters from the original Schoch (2013) matrix, but most of the ambiguous character states first appeared in the matrix of Pardo et al. (2017a). Pardo et al. (2017a)’s typeset character list does not reflect these apparent added states, and there is no comment in either the main text or supplemental information about character changes, but these added states were mostly gleaned from their NEXUS script in the supplemental information. Character numbering in this section refers to the original numbering of Schoch et al. (2020) regardless of the omission of several from the dataset used in our analyses (Characters 14, 78, 81, 115, 232, 253, 352); the new numbering scheme is detailed below in Supplementary Information 8. Note that characters discussed in this section are written without modification from those listed by Schoch et al., (2020); modifications to these characters are detailed below, and the final characters used in our analyses are reported in Supplementary Information 8.

- **3 (ornament (elements)). Reticulate ridges of various sizes (0), or isolated pustules (1).**
  - State 2 is defined herein as ‘absent.’
- **9 (preorbital region (length)). Less than twice the length of posterior skull table (0), or more (1).**
  - State 2 is defined herein as ‘twice as long.’
- **26 (orbit location). Medial, framed by wide jugals laterally (0), or lateral emplacement, framed by very slender jugals (1).**
  - State 2 is not stated and was introduced by Pardo et al. (2017a). It was coded for *Amphibamus, Doleserpeton, Gerobatrachus,* and *Platyrhinops* among temnospondyls; for *Ambystoma, Cryptobranchus, Hynobius*, *Leptodactylus*, and *Xenopus* among extant lissamphians; and for *Eocaecilia, Karaurus,* and *Triadobatrachus* among extinct lissamphibians. This is apparently a feature intended to differentiate lissamphibians and putative close dissorophoid relatives from other in-group taxa. Personal communication with J. Pardo for another study clarified that state 2 refers to enlarged orbits that are framed by both slender jugals and slender frontals. *Eocaecilia* is therefore changed to state 1. Furthermore, it is unclear why other taxa with proportionately large orbits that can be said to have both slender jugals and slender frontals were not recoded to state 2, including the early diverging amphibamiform *Micropholis*, micromelerpetids, the highly nested branchiosaurid amphibamiforms, and *Lapillopsis* (see Schoch & Milner, 2014:figs. 25–27, for example). The orbits of *Platyrhinops* in particular are proportionately much smaller than in other amphibamiforms coded for state 2, which might relate to its large size; the interorbital region is wider than the orbits, and therefore the frontals are not slender, and the orbits are more laterally situated (Clack & Milner, 2010). In other words, if *Platyrhinops* is coded as state 2, all of the aforementioned taxa should be as well. We recoded *Platyrhinops* to state 1 and recoded *Apateon* to state 2.
- **112 (cultriform process (width)). Base not wider than rest, clearly set off from basal plate (0), or merging continuously into plate (1).**
  - State 2 is defined herein as ‘flaring anteriorly.’
- **158 (presacral count). More than 28 (0), or 23-27 vertebrae (1), or less than 23 (2).**
  - State 3 was introduced by Schoch et al. (2020). Schoch et al. did not provide a full character list or a script with character names and definitions, and their list of character changes does not include any mention of character alterations. State 3 was coded for *Amphibamus* (18? presacrals) and *Gerobatrachus* (17 presacrals) among temnospondyls and for *Ambystoma opacum* (14 presacrals)*, Cryptobranchus allegheniensis* (9 presacrals)*, Hynobius* (15–18 presacrals), *Leptodactylus* (8 presacrals), and *Xenopus* *tropicalis* (8 presacrals) among extant lissamphians, and for *Karaurus* (12–13 presacrals)*, Triadobatrachus* (15 presacrals), and *Triassurus* (16 presacrals) among extinct lissamphibians (number in parentheses represents presacral count). *Amphibamus* represents the upper bound for taxa coded for state 3, with a presumed 18 presacral positions (e.g., Clack & Milner, 2010; Schoch & Milner, 2014, as opposed to Schoch et al., 2020, in which they state it has 24). If state 3 is ‘<19 presacral positions,’ this would also align with why *Apateon* (19–22 positions) was coded for state 2.
- **170 (pleurocentrum (ventral extension)). Wedged between successive intercentra and not reaching ventral margin of intercentra (0), or pleurocentra ventrally expanded to near each other (1), or ventrally fused to form a single cylindral element (2).**
  - State 3 was introduced by Schoch et al. (2020) without a detailed description. It was coded only in *Brachydectes* and *Batropetes* and is inferred here to represent the condition where the pleurocentra fuse with the intercentra to form a single centrum (holospondyly). It remains to be tested whether this indeed occurs in these taxa specifically, or whether all early tetrapods with this external condition share a homologous condition (see previous discussion of the centra of plagiosaurids). Codings for this character are unchanged herein.
- **171 (ribs (length)).** Moderately elongate thoracic ribs curved distoventrally (0), or such ribs foreshortened without distal curvature (1).
  - State 2 was introduced by Schoch et al. (2020) without a detailed description. It was coded only in *Brachydectes* and *Batropetes* (this character was not coded for the third ‘lepospondyl’ added by these authors, *Rhynchonkos*). Both of these taxa have ribs with distoventral curvature (e.g., Carroll & Gaskill, 1978:figs. 95–96; Wellstead, 1991:fig. 1; Carroll, 1991:fig. 3). Their relative length differs greatly in perception at least because lysorophians like *Brachydectes* have greatly elongated trunks at least twice as long most temnospondyls, while *Batropetes* has a shortened trunk similar to that of some early diverging lissamphibians. The ribcage of *Brachydectes* also expands notably in width towards the mid-length of the trunk before tapering again towards the pelvis. The only immediate hypothesis for what state 2 represents is an intermediary state between the two existing states: either moderately elongate ribs that are not curved distally (not observed in these taxa) or short ribs with distal curvature (‘short’ being arbitrary). The character is not modified in light of this uncertainty and because it apparently only serves to differentiate some ‘lepospondyls’ from other taxa, but this will require future redress if the taxon sample is expanded.
- **201 (ilium (orientation)). Main axis of shaft inclined posterodorsally (0), or vertical (1).**
  - State 2 is defined herein as ‘anteriorly.’

**Character specific changes.** The following comments pertain to characters in their entirety, including but not limited to, issues with character construction, character dependencies, and character redundancies. Character numbering in this section refers to the original numbering of Schoch et al. (2020) regardless of the omission of several from the dataset used in our analyses (Characters 14, 78, 81, 115, 232, 253, 352); the new numbering scheme is detailed in Supplementary Information 8. Taxa are listed only by the genus for readability, as no genus has more than one species sampled in this matrix. Lists of taxa are ordered following their order in the matrix.

- **4 (ornament, snout):** a third state (numerous circular to subcircular pits) is added to capture the condition found in gymnophionomorphs.
  - *Ichthyophis, Epicrionops*, and *Eocaecilia* were recoded to the new state 4–2.
- **13 (premaxillary foramen); 14 (premaxillary fontanelle):** character 13 refers to a small opening at the tip of the snout typically called the ‘internarial fenestra’, ‘internarial pit,’ or ‘internarial fontanelle’ in temnospondyls (e.g., Dilkes & Reisz, 1987:3; Clack & Milner, 2010:279; Milner, 2018:217; Schoch, 2019a:144). Character 14 appears to refer to the enlarged opening found in zatracheids that is convergent with a large fontanelle found in many lissamphibians that is larger than the ‘foramen of temnospondyls but smaller than the ‘fontanelle’ of zatracheids.
  - **13. Premaxillary foramen.** Premaxilla and nasal completely sutured (0), or medially bearing an opening separating the two alary processes of the premaxilla (1). [*added by Schoch, 2013*]
  - **14. Premaxillary fontanelle.** Premaxillae with or without small opening (0), or encircling large fenestra extending posteriorly between the nasals (1). [*added by Schoch, 2013*]

All taxa coded for state 14–1 (fontanelle present) were coded for state 13–1 (fenestra present), but in none of these taxa are there two separate openings framed between the premaxilla and nasal (the internasal opening of *Kokartus* is considered to be a separate condition not homologous to 14–1 because it is entirely within the nasals; Skutschas & Martin, 2011). This is therefore a partial dependency (there must be a premaxillary foramen whenever there is a premaxillary fontanelle; or conversely, there cannot be a fontanelle if there is no foramen). There is also a question of whether *Acanthostomatops* should be coded the same as *Zatrachys.* While a large fontanelle diagnoses the clade (e.g., Schoch & Milner, 2014), the opening in *Acanthostomatops* is substantially smaller than in either *Zatrachys* or in *Dasyceps* (the latter is not sampled). It barely divides the nasals posteriorly in *Acanthostomatops* (it fully divides them in *Zatrachys* and divides them for about half the length in *Dasyceps*), and it can be argued that *Acanthostomatops* has a condition closer to non-zatracheids like *Cacops*. That *Acanthostomatops* has a slightly larger fontanelle than *Cacops* is a combination of premaxillary elongation (for which there is already a character) and overall skull proportions.

- - Characters 13 and 14 were first combined into a three-state character for the median rostral opening. The new 13–1 is defined as relative small opening (more ‘foramen’ than ‘fontanelle’); examples include *Dendrerpeton*, *Acheloma*, *Phonerpeton*, and *Benthosuchus*. The new 13–2 is defined as a large opening, with any taxon originally coded for 14–1 newly coded for this; the best example is *Zatrachys*. Character 14 is omitted.
    - We consider the condition of *Acanthostomatops* (originally 14–1) to be closer to that of *Cacops* (present and relatively small in *C. morrisi* and *C. woehri* but uncertain in the sampled *C. aspidephorus*) than to *Zatrachys*; the fact that the opening in *Acanthostomatops* is slightly larger than in *Cacops* has more to do with the elongated premaxillae (captured in other characters) than to a prominent posterior expansion between the nasals, as in *Zatrachys*.
  - *Xenopus* was corrected from 13–1 to 13–0 because the genus has a fused nasal and there is no opening within it (e.g., Henderson, 2002:24; Goodman et al., 2021:fig. 2A). *Ambystoma* and *Karaurus* were also corrected from 13–1 to 13–0 because they have no apparent opening between the unfused nasals or premaxillae in the most mature specimens (e.g., Parker, 1887:pl. 27.6; Skutschas & Martin, 2011:825, 828; Schoch et al., 2020:fig. 3).
  - *Micromelerpeton* was corrected from 13–0 to 13–1 because this taxon has an oval internarial fenestra (Boy, 1995; Schoch & Milner, 2014).
  - *Cochleosaurus* was corrected from 13–0 to 13–0&1. Sequeira (2004:25) noted the presence of a small foramen in some specimens (it is not reconstructed in that paper or subsequent reconstructions).
- **20 (nasal, lateral margin):** state 1 specified a lateral expansion “anterior to lacrimal.” This definition is problematic because (1) it should not be coded for taxa lacking a lacrimal (but has been previously; e.g., *Siderops, Batrachosuchus*); (2) state 20–1 is redundant with state 219–1 (lacrimal excluded from naris) because the anterolateral expansion will partially or fully contribute to that exclusion. Previously, taxa with a clearly stepped nasal that does not exclude the lacrimal have been coded as 20–0 (e.g., *Amphibamus, Balanerpeton*), which is discordant with how Pardo et al. (2017a) coded *Hynobius* and *Karaurus* and which indicates that the character was coded more as a “nasal with lateral excursion anterior to lateral” character than as a “nasal with stepped lateral margin” character. We prefer to remove the mention of the lacrimal because the lacrimal-naris relationship is accounted for in character 219, and as noted above, some taxa lack lacrimals.
  - Codes were recoded from 20–0 to 20–1 for *Balanerpeton, Trimerorhachis, Zatrachys, Micromelerpeton, Micropholis, Gerobatrachus, Lydekkerina, Plagiosuchus, Gerrothorax, Sangaia, Edingerella,* and *Callistomordax.*
  - Codes were corrected to polymorphisms from previously coded single states (i.e., these should have been coded as such prior to the character modification) for *Cochleosaurus*, *Platyrhinops*, *Onchiodon*, *Trematosaurus*, and *Mastodonsaurus*.
- **22 (lacrimal, lateral suture); 23 (lacrimal, position); 24 (lacrimal, posterior extension); 43 (prefrontal, process); 59 (jugal-lacrimal):** any taxon coded for state 21–1 (lacrimal absent) cannot be coded for these characters.
  - Character 22: Codes were corrected from 22–0 (lateral suture parallel to medial suture) for *Siderops, Batrachosuchus,* and *Celtedens.*
  - Character 23: Codes were corrected from 23–0 (extending anterior to orbit) for *Celtedens*.
  - Character 24: Codes were corrected from 24–0 (restricted to the antorbital region) for *Siderops, Batrachosuchus,* and *Celtedens.*
  - Character 43: Codes were corrected from 43–0 (prefrontal-lacrimal suture simple) for *Siderops, Batrachosuchus, Celtedens* and *Ichthyophis*.
  - Character 59: Codes were corrected from 59–0 (jugal-lacrimal separated by orbit or palate bones) for *Celtedens* and *Chinlestegophis*.
  - For all five characters, all taxa originally coded as unknown were updated to inapplicable for consistency.
- **25 (orbit and naris); 35 (infraorbital sulcus); 44 (prefrontal-jugal); 45 (prefrontal-maxilla):** modified along the lines of character 20 to remove reference specifically to the lacrimal. No codes were changed.
- **25 (orbit and naris):** state 25–1 (separated only by narrow gap of bone) was only coded for the dvinosaurs *Isodectes* and *Acroplous* (among temnospondyls) in previous derivates (neither was retained by Schoch et al., 2020). However, it is applicable to all trematopids, in which a posterior expansion of the naris incises into the lacrimal, creating a narrow separation of orbit and naris that is proportionately narrower than in those dvinosaurs (e.g., Berman et al., 1985, 1987, 2010, 2011; Dilkes, 1990; Sumida et al., 1998; Polley & Reisz, 2011; Milner, 2018; Gee & Reisz, 2020). The sampled trematopids were previously coded for 25–0 (well separated).
  - *Acheloma* and *Phonerpeton* were recoded to inapplicable from 25–0 to avoid redundancy because the elongate naris that typifies this clade is the result of posterior expansion towards the orbit and is captured in character 18.
  - State 25–1 is also applicable to some branchiosaurids (e.g., *Apateon pedestris*; Schoch & Milner, 2008:fig. 1; Schoch & Milner, 2014:fig. 26) and plagiosaurids (e.g., *Plagiosuchus*; Damiani et al. (2009:fig. 7A). In this matrix, *Apateons*, *Gerrothorax*, and *Plagiosuchus* were changed to 25–1 from 25–0.
- **28 (palpebral ossifications):** this is a complicated character to code because these ossifications, as well as the scleral ring, may be predisposed to be dislodged post-mortem. The previous codings are peculiar because nearly all temnospondyls, including some known from singletons and those without any known scleral rings, were coded (for 28­–0), but four dissorophoids were not: *Gerobatrachus*, *Cacops, Acheloma,* and *Phonerpeton*. This is conspicuous because most dissorophoids do have palpebral ossifications (28­­–1). There is clearly some discrepancy in coding philosophy here where this character was probably left uncoded for these four taxa because palpebral ossifications are unknown from any specimen, but it could be reasonably inferred that they were present in life (i.e. coding them as ‘absent’ would produce hypothetically false conflicting information). There are two approaches that can be taken here: either to code the character as preserved, without considering the possibility of post-mortem loss; or to only code the character when sclerotic ring plates are at least partially preserved, as the absence of these plates would indicate that preservation was likely insufficient for palpebral ossifications to be preserved if present. The latter approach is taken here for two reasons: (1) it is more conservative and accounts for taphonomy; and (2) both states technically require scleral ring ossicles to be present to be coded.
  - The following taxa were uncoded from 28–0 (no palpebral ossifications) because the literature does not establish the presence of sclerotic ring plates in these taxa: *Proterogyrinus, Greererpeton, Brachydectes, Batropetes, Rhynchonkos, Trimerorhachis, Zatrachys*, *Eryops, Glanochthon,* *Lydekkerina,* *Siderops, Batrachosuchus, Plagiosuchus, Sangaia, Gerrothorax*, *Edingerella, Benthosuchus, Trematosaurus, Trematolestes, Lyrocephaliscus, Callistomordax*, *Metoposaurus, Parotosuchus, Paracyclotosaurus, Mastodonsaurus*, and *Celtedens*.
- **29 (pineal foramen); 33 (lateral line sulci); 154 (ceratobranchials); 155 (basibranchial); 156 (hypobranchial elements); 188 (scapula); 191 (humerus, adult shaft); 356 (palatine):** all of these characters specifically invoke the condition of adults. This means any taxon represented only by juvenile individuals cannot be coded for these characters.
  - Codes were corrected to unknown for characters 29, 154, 156, 188, 191, and 356 for *Gerobatrachus*, which Anderson et al. (2008:515) interpreted as represented by a holotype skeleton (the only specimen) that “belonged to a juvenile individual.”
- **34 (sulci, skull roof); 35 (infraorbital sulcus); 36 (supraorbital sulcus):** any taxon coded for state 33–1 (lateral line sulci absent) cannot be coded for any of these characters. Codes for characters 34–36 are strange; for example, nearly all taxa coded for 33–1 were left uncoded for character 34, except for *Micropholis stowi* (34–0). All temnospondyls that were coded for 33–1 were also previously coded for both 35–0 and 36–0, but the three ‘lepospondyls’ coded for 33–1 were left uncoded for both characters.
  - Character 34: Codes were corrected from 34–0 (sulci present throughout skull roof if present) for *Micropholis*.
  - Character 35: Codes were corrected from 35–0 (infraorbital sulcus with simple curve) for *Edops, Cochleosaurus, Dendrerpeton, Balanerpeton, Acanthostomatops, Zatrachys, Apateon, Micropholis, Platyrhinops, Doleserpeton, Amphibamus*, *Dissorophus, Cacops, Broiliellus, Acheloma, Phonerpeton, Onchiodon,* and *Eryops*.
  - Character 36: Codes were corrected from 36–0 (supraorbital sulcus passing entirely through nasal) for *Edops, Cochleosaurus, Dendrerpeton, Balanerpeton, Acanthostomatops, Zatrachys, Apateon, Micropholis, Platyrhinops, Doleserpeton, Amphibamus*, *Dissorophus, Cacops, Broiliellus, Acheloma, Phonerpeton, Onchiodon,* and *Eryops*.
  - *Gerobatrachus hottoni* was originally coded for 36–0 but uncoded for 33, 34, and 35. This was corrected to ‘?’ for character 36 (see above comments on coding of character 33 only for adults; note also that the single specimen is only exposed ventrally; Anderson et al., 2008b).
  - For all three characters, all taxa originally coded as unknown were updated to inapplicable for consistency.
- **36 (supraorbital sulcus):** this character cannot be modified like characters 25 and 35 to omit the lacrimal as a reference point because there is a genuine difference between some taxa in which the groove passes onto the nasal and prefrontal but not the lacrimal (some capitosaurs) and others in which it passes onto these elements and the lacrimal (e.g., *Mastodonsaurus*). Additionally, the state in which it contacts the nasal and prefrontal is technically not captured. The character is modified to add a state for this. However, this character therefore cannot be coded for taxa without either lacrimals or prefrontals.
  - Codes were corrected from 36–0 (supraorbital sulcus passing entirely through nasal) for *Siderops, Batrachosuchus,* and *Plagiosuchus*, which either lack lacrimals (21–1) or prefrontals (314–1). *Chinlestegophis, Leptodactylus, Xenopus, Celtedens, Ambystoma, Cryptobranchus, Ichthyophis, Epicrionops,* and *Eocaecilia* were updated from unknown to inapplicable for consistency.
  - Codes were recoded to 36–1 for *Greererpeton*, *Micromelerpeton*, *Archegosaurus*, *Uranocentrodon*, *Lydekkerina*, *Gerrothorax*, and *Parotosuchus,*
  - Codes were recoded to 36–2 for *Sangaia*, *Edingerella*, *Benthosuchus*, *Trematosaurus*, *Lyrocephaliscus*, *Metoposaurus*, *Paracyclotosaurus*, and *Mastodonsaurus.*
  - *Trimerorhachis* was recoded as ‘1&2’ (Milner & Schoch, 2013:fig. 2).
  - *Glanochthon* was uncoded as there is no explicit description or figuring of this specific sulcus (Schoch & Witzmann, 2009b:126, fig. 2C).
- **39 (prefrontal, frontal):** this character did not encompass the state where the prefrontal terminates posterior to the anterior extent of the frontal. A third state was added for this. The language for 39­–0 was changed to more clearly indicate that this refers to the anterior extent, not the element’s length, which may have caused some previous confusion based on examination of codes added by Pardo et al. (2017a); because this is not certain, these corrections are listed in the taxon-specific section.
  - Codes were updated to 39–2 for *Rileymillerus* and *Cryptobranchus*.
- **36 (supraorbital sulcus); 39 (prefrontal, frontal); 40 (prefrontal, anterior end); 41 (prefrontal, lateral suture); 42 (prefrontal-postfrontal); 43 (prefrontal, process); 44 (prefrontal-jugal); 45 (prefrontal-maxilla); 226 (prefrontal contributes to external naris)**: these characters cannot be coded for taxa coded as 314–1 (prefrontals absent).
  - Codes were corrected from 36–0 (see above); 39–0 (prefrontal substantially longer than frontal); 40–1 (prefrontal anterior end wide and blunt); 41–0 (prefrontal lateral suture straight or concave); 42–1 (prefrontal-postfrontal separated by frontal); 43–0 (prefrontal-lacrimal suture simple); 44–1 (prefrontal-jugal in contact); and 45–0 (prefrontal-maxilla separated) for *Plagiosuchus*; the code for character 226 was changed from unknown to inapplicable for consistency for this taxon.
  - Codes for all eight characters were updated from unknown to inapplicable for *Leptodactylus* and *Xenopus* for consistency.
- **42 (prefrontal-postfrontal):** a large number of taxa originally coded for this character by Schoch (2013) were coded for the wrong state. This does not seem to be a random typographic error, but there is also no evidence for the character states having been merely inverted because some taxa were correctly coded. State 42–1 (separated by frontal) occurs in the majority of dissorophoids (but not in early-diverging Carboniferous taxa) and most capitosaurs. All dissorophoids were originally coded for 42–1, even though Carboniferous taxa like *Platyrhinops* have a prefrontal-postfrontal contact (e.g., Clack & Milner, 2010). Conversely, all capitosaurs were originally coded for 42–0, even though taxa like *Parotosuchus* and *Mastodonsaurus* lack this contact (e.g., Schoch, 1999, 2018). These observations lend further credence to the hypothesis that the errors are not random typographic errors.
  - The following taxa were corrected from 42–0 (prefrontal-postfrontal contact) to 42–1: *Edingerella*, *Parotosuchus*, *Paracyclotosaurus*, *Cyclotosaurus*, and *Mastodonsaurus*.
  - The following taxa were corrected from 42–1 (prefrontal-postfrontal separated) to 42–0: *Greererpeton*, *Trimerorhachis*, *Acanthostomatops*, *Zatrachys*, *Platyrhinops*, *Amphibamus*, *Onchiodon*, *Eryops*, *Siderops*, and *Batrachosuchus*.
- **42 (prefrontal-postfrontal); 50 (postorbital, postfrontal); 61 (intertemporal and postorbital); 218 (postfrontal shape)**: these characters cannot be coded for taxa coded for 315–1 (postfrontals absent).
  - Codes were corrected from 42–1 (see above); 50–0 (postorbital and postfrontal shorter than supratemporal and parietal); and 61–0 (postorbital lateral to postfrontal) for *Plagiosuchus*; the code for character 218 was changed from unknown to inapplicable for consistency for this taxon.
  - Codes for all four characters were updated from unknown to inapplicable for consistency in *Brachydectes, Celtedens*, *Triadobatrachus, Leptodactylus, Xenopus, Celtedens, Karaurus, Ambystoma, Hynobius,* and *Cryptobranchus*.
- **47 (postorbital, shape); 48 (postorbital, end); 49 (postorbital); 50 (postorbital, postfrontal); 61 (intertemporal and postorbital); 231 (shape of postorbital); 232 (parietal-postorbital contact):** these characters cannot be coded for any taxon coded as 230–1 (postorbital absent).
  - Codes were corrected from 47–0 (postorbital long triangular, wedged deeply between squamosal and supratemporal); 48–0 (posterior end acutely triangular); 49–0 (not wider than orbit); 50–0 (see above); 61–0 (see above) for *Plagiosuchus*; the codes for characters 231 and 232 were changed from unknown to inapplicable for consistency for this taxon*.*
  - Codes were corrected from 47–1 for *Ichthyophis* and *Epicrionops.*
  - Codes were corrected from 50–0 for *Ichthyophis*, *Epicrionops*, and *Eocaecilia*.
  - Codes for these seven characters were updated from unknown to inapplicable for *Triadobatrachus, Leptodactylus, Xenopus, Celtedens, Karaurus, Ambystoma, Hynobius, Cryptobranchus,* and *Eocaecilia* for consistency.
- **50 (postorbital, postfrontal); 53 (supratemporal); 54 (supratemporal, width); 56 (semilunar flange); 217 (supratemporal exposure on occiput)**: these characters cannot be coded for any taxon coded as 216–1 (supratemporal absent).
  - Character 50: Codes were corrected from 50–0 (postorbital and postfrontal shorter than supratemporal and parietal) for *Batropetes* and *Rhynchonkos*.
  - Character 56: Codes were corrected from 56–0 (supratemporal without ventral projection into otic notch) for *Batropetes, Rhynchonkos, Leptodactylus*, and *Xenopus.*
  - Character 217: Codes were corrected from 217–0 (supratemporal exposure on occiput absent) for *Batropetes, Brachydectes, Rhynchonkos*, and *Celtedens.*
  - Codes for these five characters were updated from unknown to inapplicable for the above taxa (when originally coded as unknown) as well as for *Triadobatrachus, Ichthyophis*, and *Epicrionops* for consistency.
- **52 (otic notch, position); 55 (supratympanic flange); 56 (semilunar flange); 229 (large otic notch approaching orbit):** any taxon coded for 51–1 (otic notch absent, straight transverse posterior skull margin) cannot be coded for these characters (as was properly applied originally for character 52: otic notch position).
  - Character 55: Codes were corrected from 55–0 (squamosal continuously ornamented around margin of otic notch) for *Greererpeton*, *Siderops, Batrachosuchus, Plagiosuchus, Sangaia,* and *Gerrothorax*.
  - Character 56: Codes were corrected from 56–0 (supratemporal without ventral projection into otic notch) for *Greererpeton, Siderops, Batrachosuchus, Plagiosuchus, Sangaia,* and *Gerrothorax*.
  - Character 229: Codes were corrected from 229–0 (large otic notch approaching orbit absent) for *Greererpeton*, *Brachydectes, Batropetes, Rhynchonkos, Siderops, Batrachosuchus, Plagiosuchus, Sangaia*, *Gerrothorax, Rileymillerus, Celtedens, Ambystoma, Hynobius, Cryptobranchus, Ichthyophis,* and *Epicrionops*. Note that state 0 here is not a redundant state with 51–1; 229–0 is coded for many taxa with otic notches, including most temnospondyls.
  - Codes for these four characters were updated from unknown to inapplicable for the above taxa (when originally coded as unknown) as well as for *Karaurus* and *Kokartus* for consistency.
- **61 (intertemporal and postorbital); 232 (parietal-postorbital):** the language for character 61 is somewhat vague, but it expresses the same fundamental feature as character 232 –postorbital-parietal contact (original definitions shown below).
  - **61. Intertemporal and postorbital.** Postorbital lateral to postfrontal (0), or expanding medially to replace intertemporal, contacting parietal (1). [*added by Schoch, 2013*]
  - **232. Parietal-postorbital contact.** Absent (0), present (1). [*added by Pardo et al., 2017*]

Character 61 is maintained in its position but is replaced by the wording of character 232 for clarity, and character 232 was not modified or replaced by a new character (its position is omitted). The only coding change was to the newly substituted *M. krasiejowensis*, which is now coded as polymorphic. All other codes were unchanged as the character states are directly equivalent (i.e., both 61–1 and 232–1 were for parietal-postorbital contact).

- **62 (squamosal-tabular); 63 (tabular and squamosal); 65 (tabular, horn); 66 (tabular, extension); 67 (tabular, ventral crest); 71 (posterior skull rim); 234 (parietal-tabular):** these characters cannot be coded for taxa coded for 239–1 (tabular absent; exclusive to lissamphibians).
  - Character 63: Codes were corrected from 63–0 (tabular and squamosal forming either squamosal embayment or straight posterior margin) for *Karaurus*, *Kokartus*, *Ambystoma*, *Hynobius*, and *Cryptobranchus*.
  - Character 71: Codes were corrected from 71–0 (quadrate trochea posterior to tabular horns) for *Triadobatrachus.* Codes were corrected from 71–1 (quadrate trochlea at one level or anterior to tabular horns) for *Leptodactylus, Xenopus, Celtedens, Karaurus, Kokartus, Ambystoma, Hynobius,* and *Cryptobranchus.*
  - Codes for these seven characters were updated from unknown to inapplicable for the above taxa (when originally coded as unknown) as well as for *Ichthyophis* and *Epicrionops* for consistency.
- **65 (tabular, horn); 66 (tabular, extension)**: character 66 cannot be coded for taxa coded for 65–1 (tabular horn absent).
  - Character 66: Codes were corrected from 66–0 (tabular horn pointing posteriorly if present) for *Greererpeton, Brachydectes, Batropetes, Rhynchonkos, Batrachosuchus, Plagiosuchus,* and *Gerrothorax*.
  - *Chinlestegophis* was recoded to 65–1 because Pardo et al. (2017) expressly questioned whether this was homologous with the tabular horn of other temnospondyls given its occipital position, as with brachyopoids (they cited *Batrachosuchus*, which was coded as lacking a tabular horn [65–1] in their matrix). It was then recoded as inapplicable for character 66.
  - The code for character 66 was updated from unknown to inapplicable for *Rileymillerus*.
  - The treatment of rhytidosteids for these characters is complicated. Some workers consider there to be a tabular horn when the tabular extends posterolaterally (e.g., in *Sangaia*; Dias-da-Silva et al., 2006) such that the posterior skull margin is not straight or posteriorly convex (in contrast to brachyopoids and plagiosaurids, which definitively lack tabular horns). In this interpretation, the tabular horn is present and merely not ‘separate’; instead, it is jointly formed by the squamosal, precluding an otic notch. *Laidleria* (sampled in previous derivates but not by Schoch et al., 2020) also has this condition, but Warren (1998b) did not consider it to have a tabular horn. Here, *Sangaia* is considered to have tabular horns and is recoded to 65–0 and 66–0.
- **69 (quadratojugal-maxilla):** codes for this binary character appear to be reversed because taxa without this contact (separation by jugal) are largely coded for 69–0 (in contact), such as the majority of stereospondyls, while taxa with this contact are largely coded for 69–1 (contact absent), such as *Greererpeton* and dissorophoids. This pattern only appeared among taxa first sampled by Schoch (2013); taxa subsequently added to the matrix have no pattern of unsubstantiated codes. Because some taxa were correctly coded originally (e.g., *Gerrothorax, Trematolestes*), the codes in the matrix were checked and subsequently reversed for most taxa, rather than automatically reversing codes for all taxa originally coded by Schoch (2013).
  - Codes were reversed (corrected) for *Proterogyrinus*, *Greererpeton, Edops, Cochleosaurus, Dendrerpeton, Balanerpeton, Trimerorhachis, Acanthostomatops, Zatrachys, Micromelerpeton, Apateon, Micropholis, Platyrhinops, Doleserpeton, Amphibamus, Gerobatrachus, Dissorophus, Cacops, Broiliellus, Acheloma, Phonerpeton, Onchiodon, Eryops, Sclerocephalus, Glanochthon, Archegosaurus, Uranocentrodon, Lydekkerina, Plagiosuchus, Edingerella, Metoposaurus, Paracyclotosaurus,* and *Cyclotosaurus.*
  - Codes were changed to unknown (corrected) for *Rhynchonkos* (no quadratojugal is known; Szostakiwskyj et al., 2015:10); *Siderops* (lateral and palatal surfaces of the quadratojugal are unknown; Warren & Hutchinson, 1983:6); and *Batrachosuchus* (no maxillary sutures are discernible or preserved; Chernin, 1977:102).
- **71 (posterior skull rim):** this character originally specified two reference points: the tabular horns and the quadrate condyles. However, not all taxa have tabular horns (65–1), yet such taxa were coded for this character (e.g., ‘lepospondyls’, *Batrachosuchus*, *Plagiosuchus*). The original codes were correct if the ‘tabular horn’ reference point was changed to the ‘posteriormost extent of the tabular’; in taxa with horns, the horn is the posteriormost extent, and taxa without horns could then be properly coded. This change was implemented and did not require any coding modifications beyond those noted above for other dependencies.
- **72 (occipital flange); 73 (postparietal-exoccipital); 236 (postparietal-squamosal); 237 (postparietal length):** these characters cannot be coded for any taxon coded for 235–2 (postparietals absent).
  - Character 72: Codes were corrected from 72–0 (descending flange of occipital portion of postparietals forming a bulge) for *Batropetes*.
  - Character 73: Codes were corrected from 73–0 (no postparietal-exoccipital contact) for *Batropetes*.
  - Character 236: Codes were corrected from 236–0 (postparietal-squamosal contact absent) for *Batropetes*.
  - Codes for these four characters were updated from unknown to inapplicable for *Batropetes* (for character 237) as well as for *Triadobatrachus, Leptodactylus, Xenopus, Karaurus, Ambystoma, Hynobius, Cryptobranchus, Ichthyophis, Epicrionops* for consistency.
- **78 (dentition, upper jaw):** The two states of this character were not clearly differentiated. State 78–1 (caniniform) was coded only for the trematopids *Acheloma* and *Phonerpeton*. Caniniform dentition in the form of a region of proportionately enlarged teeth on the anterior part of the maxilla is common to trematopids and differentiates them from their sister group (dissorophids), but short regions of enlarged teeth also occur in taxa like eryopoids and *Sclerocephalus* (e.g., Sawin, 1941; Boy, 1990; Schoch & Witzmann, 2009a). This accounts for the morphology captured in state 37–1 (maxilla anterior margin laterally convex due to enlarged teeth). Based on this redundancy, it might be presumed that ‘caniniform’ refers to a morphology, not to size, as 78–0 is ‘conical to slightly curved inwards.’ This does not clearly invoke a contrasting morphology like a compressed tooth with a keel or with carinae (which occurs in some stereospondyls, like *Metoposaurus* and *Siderops*; Warren & Hutchinson, 1983; Milner & Schoch, 2004), and in any event, trematopids do have conical teeth that curve slightly inwards (e.g., Gee et al., 2019:fig. 3). This character is therefore omitted.
- **81 (dentition, vomer); 251 (denticles on vomer):** these characters refer to the same feature, although worded differently.
  - **81. Dentition (vomer).** Tooth patches present at least in small specimens (0), or dentition entirely restricted to vomerine fangs (1). [*added by* Schoch, 2013]
  - **251. Denticles on vomers.** Present (0), absent (1). [*added by Pardo et al., 2017*]
  - *Gerobatrachus* was uncoded from 81–1; Anderson et al. (2008) mention a few rows of teeth medial to the choana, which based on their figure, appear closer to the size of typical denticles than other forms of vomerine dentition. *Sangaia* was uncoded from 81–1 because there is no indication that the vomers are known from any specimen (Dias-da-Silva et al., 2006).

It is interpreted that “tooth” in character 81 refers only to a shagreen of denticles, as this character was coded as 81–1 (dentition entirely restricted to vomerine fangs) in *Limnogyrinus* and *Apateon* (the former sampled in previous derivates but not retained by Schoch et al., 2020), which have teeth larger than denticles, but smaller than the marginal teeth, on the vomer, and in most stereospondyls, which frequently have transvomerine and parachoanal dentition that is of a similar relative size. Character 251 is maintained as is, and character 81 is omitted.

- **87 (parasphenoid, shagreen); 253 (denticles on parasphenoid):** characters 87 and 253 are clearly redundant and both express binary ‘presence/absence’ conditions, with 87–0 and 253–0 as ‘present’ and 87–1 and 253–1 as ‘absent.’
  - **87. Parasphenoid (shagreen).** Tooth patches present (0), or absent (1). [*added by Schoch, 2013*]
  - **253. Denticles on parasphenoid.** Present (0), absent (1). [*added by Pardo et al., 2017a*]

That most taxa are coded the same for both characters (e.g., 87–0 and 253–0) indicates that both have been consistently interpreted as referring to specifically denticles (rather than character 87 referring to non-denticle dentition). Character 87 is retained simply because it occurs earlier in the matrix. However, coded states differ for several taxa between them:

- - *Trimerorhachis* was most recently described by Milner & Schoch (2013:107), who stated that “the presence of a denticle field on the ventral side of the basal plate of the parasphenoid is not clear; if present, it must have been small and faintly developed.” The conservative way to reconcile the differences (87–0, 253–1) is to code it as unknown, but with the addition of a third state (see below for character 352), this can be coded as partial uncertainty for states 0 and 1.
  - *Dissorophus multicinctus* has not been redescribed since DeMar (1968:1217) who stated that only vomerine palatal teeth could be confidently identified. Schoch (2012:fig. 3D) reconstructed this taxon without parasphenoidal dentition, as with most dissorophids, but no primary data have been newly provided to substantiate this (Milner’s 2003 redescription of a junior synonym, “*Longiscitula houghae*” does not address this). The conservative way to reconcile the differences (87–0, 253–1) is to code it as unknown.
  - *Cacops aspidephorus* was most recently described by Anderson et al. (2020:7) who stated that a small surface area between the basal tubera and the basicranial articulation is covered by a shagreen of denticles, in contrast to other species of *Cacops*. This code is corrected to 87–0 from 87–1 and is considered a mischaracterization.
  - *Sclerocephalus haeuseri* was most recently described by Schoch & Witzmann (2009a:148), who made no comment on dentition of the parasphenoid but who did remark that there are denticles on the rest of the palatal bones. Schoch (2003) noted a decrease in the presence of denticles on this element throughout early ontogeny, which was visually shown by Boy (1988:figs. 3–4). We feel that this is sufficient to correct the code to 87–1 from 87–0 and is considered a mischaracterization.
  - *Uranocentrodon senekalensis* has not been appreciably redescribed since Broom (1930:fig. 2), who depicted denticles on the parasphenoid. This is in agreement with both earlier work (e.g., Haughton, 1925:228) and the phylogenetic coding by Marsicano et al. (2017), the most recent revision of Rhinesuchidae. This is considered sufficient to maintain the code as 87–0.
  - *Edingerella madagascariensis* was most recently described by Maganuco et al. (2009:18) who stated that there are no denticles on the parasphenoid, only a granular surface. This is considered sufficient to maintain the code as 87–1.
  - *Benthosuchus sushkini* has not been appreciably redescribed since work by Bystrow & Efremov (1940:figs. 67–69), in which it is repeatedly figured with denticle fields on the parasphenoid. This follows how previous workers have coded the taxon in phylogenetic analyses (e.g., Damiani, 2001; Schoch, 2008a), although it is not always figured as such. Novikov (2012b:402–403), in describing a new species of *Benthosuchus*, lists parasphenoid denticles as being present on the parasphenoid. This is considered sufficient to maintain the code as 87–0.
  - *Trematosaurus brauni* was most recently described by Schoch (2019b:51) who compared the topography of the basal plate to other stereospondyls with fine pitting and denticles; this taxon has also been previously reconstructed as having denticles (e.g., Schoch & Milner, 2000:fig. 77). This is considered sufficient to maintain the code as 87–0.
  - *Lyrocephaliscus euri* was most recently described by Mazin & Janvier (1983:16,19), who stated that denticles are found on the parasphenoid, as also depicted by Schoch & Milner (2000:fig. 83) and described by Save-Söderbergh (1935:23). This is considered sufficient to maintain the code as 87–0.
  - The following taxa were coded for character 87 but not for character 253 and are left as is for character 87: *Micromelerpeton, Platyrhinops, Broiliellus, Acheloma, Phonerpeton, Eryops, Sangaia, Celtedens, Kokartus*, and *Triassurus*.
  - The following taxa were coded for character 253 but not for character 87, and the code of the former (denticles absent) is newly applied to the latter: *Karaurus*.
- **87 (parasphenoid, shagreen); 115 (cultriform process, dentition); 352 (denticle field on parasphenoid):** character 352 is meant to capture the expanded denticle field of some taxa, but it is already captured in character 115 (cultriform process, dentition). States 115–1 (with elongate tooth patch) and 352–1 (greatly expanded anteriorly along cultriform process) are synonymized and subsequently appended to character 87 as a new state 2, and characters 115 and 352 are omitted.
  - **87. Parasphenoid (shagreen).** Tooth patches present (0), or absent (1). [*added by Schoch, 2013*]
  - **115. Cultriform process (dentition).** Main shaft edentulous except for base (0), or with elongate tooth patch (1). [*added by Schoch, 2013*]
  - **352. (*no character name*).** Denticle field on basal plate present or not (0), or greatly expanded anteriorly along cultriform process (1). [*added by Schoch et al., 2020*]
  - All taxa coded for 352–1 were coded as 87–2 (*Micropholis, Amphibamus, Platyrhinops, Eocaecilia*). *Acanthostomatops* and *Zatrachys* were changed from state 0 to the new state 2, as these taxa also have extensive denticle fields (e.g., Schoch, 1997; Witzmann & Schoch, 2006; originally coded for state 115­–1). These are treated as unsubstantiated codes. *Micromelerpeton* is coded as polymorphic for states 0 and 2 (Boy, 1995:fig. 3).
- **88 (ectopterygoid, fangs); 123 (pterygoid, ectopterygoid); 124 (pterygoid-palatine-ectopterygoid); 125 (palatine, ectopterygoid); 128 (palatine, ectopterygoid, ontogeny); 129 (palatine, ectopterygoid, width); 130 (palatine, ectopterygoid, continuous tooth row); 131 (ectopterygoid length); 132 (laterally exposed ectopterygoid, LEE); 133 (ectopterygoid, Y-shaped):** any taxon coded for state 346–1 (ectopterygoid absent) cannot be coded for this character.
  - Character 88: Codes were corrected from 88–1 (ectopterygoid fangs absent) for *Triadobatrachus*, *Celtedens*, and *Eocaecilia*.
  - Character 123: Codes were corrected from 123–0 (palatine ramus exclusively formed by pterygoid) for *Brachydectes*, *Triadobatrachus, Leptodactylus, Xenopus, Celtedens, Ambystoma, Hynobius*, *Ichthyophis*, *Epicrionops*, and *Eocaecilia.*
  - Character 124: Codes were corrected from 124–0 for (pterygoid contacting both ectopterygoid and palatine) for *Leptodactylus*, *Ichthyophis*, and *Epicrionops*. Codes were corrected from 124–1 (pterygoid only contacting ectopterygoid) for *Triadobatrachus*.
  - Character 125: Codes were corrected from 125–0 (palatine and ectopterygoid with simple, transverse suture) for *Brachydectes* and *Triadobatrachus*.
  - Character 128: Codes were corrected from 128–0 (palatine and ectopterygoid maintain their width throughout ontogeny) for *Brachydectes*, *Ichthyophis* and *Epicrionops*.
  - Character 129: Codes were corrected from 129–0 (palatine and ectopterygoid much wider than maxilla) for *Brachydectes, Ichthyophis, Epicrionops*, and *Eocaecilia*. Codes were corrected from 129–1 (palatine and ectopterygoid as narrow as maxilla) *Triadobatrachus* and *Leptodactylus*.
  - Character 130: Codes were corrected from 130–0 (palatine and ectopterygoid without continuous tooth row) for *Brachydectes, Triadobatrachus, Leptodactylus*, and *Eocaecilia*. Codes were corrected from 130–1 (palatine and ectopterygoid with continuous tooth row) for *Ichthyophis* and *Epicrionops*.
  - Character 131: Codes were corrected from 131–1 (ectopterygoid markedly shorter than palatine) for *Triadobatrachus*.
  - Character 132: Codes were corrected from 132–0 (lateral exposure of ectopterygoid absent) for *Triadobatrachus*.
  - Character 133: Codes were corrected from 133–0 (ectopterygoid with continuous maxillary suture) for *Triadobatrachus*.
  - Codes for these 10 characters were updated from unknown to inapplicable for the above taxa (when originally coded as unknown) as well as for *Karaurus, Kokartus,* and *Cryptobranchus* for consistency.
- **92 (anterior palatal opening(s)):** this character requires at least one palatal opening to be present between the vomer and premaxilla; the identical name to character 91 evidences that they are linked. Taxa coded for state 91–0 (vomer and premaxilla with continuous suture) cannot be coded for this character.
  - Codes were changed corrected 92–0 (opening unpaired if present) for *Proterogyrinus, Brachydectes, Rhynchonkos, Edops, Cochleosaurus, Dendrerpeton, Balanerpeton, Acanthostomatops, Zatrachys, Micromelerpeton, Apateon, Micropholis, Platyrhinops, Doleserpeton*, *Amphibamus, Gerobatrachus, Dissorophus, Cacops, Broiliellus, Acheloma, Phonerpeton, Onchiodon, Eryops, Sclerocephalus, Glanochthon, Archegosaurus, Plagiosuchus, Gerrothorax, Paracyclotosaurus, Leptodactylus, Ambystoma,* and *Hynobius,*
  - *Xenopus* and *Kokartus* were coded as unknown for character 91, so they should be left as unknown for character 92 as well.
  - *Cryptobranchus, Ichthyophis, Epicrionops,* and *Eocaecilia* were updated to inapplicable from unknown for character 92.
- **95 (anterior palatal depression):** this character requires an anterior palatal depression to be present. Taxa that lack such a depression cannot be coded for this character.
  - Codes were corrected from 95–0 (posterior rim round if present) for *Brachydectes, Batropetes, Rhynchonkos, Dendrerpeton*, and *Balanerpeton*.
- **99 (choana, lateral):** the coding of this character is perplexing. Most taxa are coded for 99–0 (choana anterolaterally expanded with triangular outline), but most taxa lack this expansion. Additionally, the few taxa originally coded for 99–1 (choana oval) have markedly disparate morphology. The trematosaur *Lyrocephaliscus* genuinely has a simple oval choana, like most other temnospondyls and *Greererpeton*, whereas *Dendrerpeton* has the marked lateral expansion that is captured in state 0, but both *Lyrocephaliscus* and *Dendrerpeton* are coded for 99–1. This indicates that there is not merely a reversal of codes, similar to character 69, and codes were systematically checked.
  - Codes were reversed (corrected) from 99–0 to 99–1 for *Proterogyrinus*, *Greererpeton*, *Balanerpeton, Trimerorhachis, Micromelerpeton, Apateon, Micropholis, Platyrhinops, Doleserpeton, Amphibamus, Gerobatrachus, Dissorophus, Cacops, Broiliellus, Acheloma, Phonerpeton, Onchiodon, Eryops, Sclerocephalus, Glanochthon, Archegosaurus, Uranocentrodon, Lydekkerina, Siderops, Batrachosuchus, Plagiosuchus, Gerrothorax, Edingerella, Benthosuchus, Trematosaurus, Trematolestes, Lyrocephaliscus, Callistomordax, Metoposaurus, Parotosuchus, Paracyclotosaurus, Cyclotosaurus, Mastodonsaurus,* and *Rileymillerus*.
  - Codes were reversed (corrected) from 99–1 to 99–0 for *Edops, Cochleosaurus,* and *Dendrerpeton*.
  - Codes were corrected from 99­–0 to unknown for *Rhynchonkos* (reconstructed palate with the lateral margin of the choana shown has not been presented, and present disarticulation precludes determination; Carroll & Gaskill, 1978; Szostakiwskyj et al., 2015).
- **102 (vomer-pterygoid); 124 (pterygoid-palatine-ectopterygoid); 125 (palatine, ectopterygoid); 126 (palatine, vomer); 127 (LEP); 128 (palatine, ectopterygoid, ontogeny); 129 (palatine, ectopterygoid, width); 130 (palatine, ectopterygoid, continuous tooth row); 131 (ectopterygoid length); 248 (anterior palatine); 252 (denticles on palatines); 355; palatine with distinct tusk pair):** any taxon coded for 248–2 (palatine absent) cannot be coded for these characters.
  - State 248–2 (palatine absent) is redundant with state 356–1 (palatine absent). Because we wanted to add a character state to differentiate between ‘palatine present and distinct from maxilla’ (the plesiomorphic state) versus ‘palatine present but co-ossified with maxilla’ (as in gymnophionomorphs), we added a third state to character 356 (a general presence/absence character) and reduced character 248 (which only refers to the anterior process of the palatine) to states 0 and 1.
  - Character 127: Codes were corrected from 127–0 (LEP absent) for *Ambystoma*.
  - Character 252: Codes were corrected from 252–0 (denticles on palatine present) for *Karaurus.* Codes were corrected from 252–1 (denticles on palatine absent) for *Ambystoma, Hynobius,* and *Cryptobranchus.*
  - Character 355: Codes were corrected from 355–0 (palatine with distinct tusk pair) for *Karaurus, Kokartus, Ambystoma, Hynobius*, and *Cryptobranchus.*
  - Codes for these 11 characters were updated from unknown to inapplicable (when originally coded as unknown) for the above taxa.
- **104 (vomer, extensions):** state 104–0 specifies a contact with the pterygoid; any taxon coded for state 102–1 (vomer-pterygoid separated by palatine) cannot be coded for this character. It is also inferred that 104–0 should be ‘vomer contacts pterygoid medial to choana,’ not ‘lateral to choana,’ as the pterygoid never extends onto or along the lateral side of the choana in the sampled taxa.
  - Codes were corrected from 104–0 (vomer contacts pterygoid lateral to choana) for *Micromelerpeton, Apateon, Micropholis, Platyrhinops, Doleserpeton, Amphibamus, Gerobatrachus, Dissorophus, Broiliellus*, *Lydekkerina, Siderops, Batrachosuchus, Plagiosuchus, Gerrothorax, Edingerella, Benthosuchus, Trematosaurus, Trematolestes, Lyrocephaliscus, Callistomordax, Metoposaurus, Parotosuchus, Paracyclotosaurus, Cyclotosaurus,* and *Mastodonsaurus*.
  - Codes were updated from unknown to inapplicable for *Triadobatrachus, Leptodactylus, Xenopus, Celtedens, Triassurus, Ichthyophis, Epicrionops, Eocaecilia,* and *Rileymillerus*.
- **106 (basicranium, contact), 107 (basicranium, suture):** character 107 requires a sutural contact between the parasphenoid and the pterygoid to be present and cannot be coded for any taxa coded for 106–0 (joint between parasphenoid and pterygoid).
  - Codes were corrected from 107–0 (suture [if present] much shorter than basal plate, reaching at best 40% its length) for *Proterogyrinus, Greererpeton, Brachydectes, Batropetes, Rhynchonkos, Dendrerpeton, Balanerpeton, Trimerorhachis, Micromelerpeton, Apateon, Micropholis, Platyrhinops, Doleserpeton, Amphibamus, Gerobatrachus, Sclerocephalus, Glanochthon,* and *Archegosaurus.*
  - All lissamphibians were updated from unknown to inapplicable because they were coded for 106–0.
- **112 (cultriform process width); 343 (parasphenoid, cultriform process):** these characters refer to the same general feature but with drastically different character states (shown below for reference). These are both maintained here because of the different states, but future workers may wish to consolidate them, or conversely, to further differentiate them (e.g., to restrict one to anterior width and one to posterior width).
  - **112. Cultriform process (width).** Base not wider than rest, clearly set off from basal plate (0), merging continuously into plate (1), flaring anteriorly (2). [*added by Schoch, 2013*]
    - 112–0 and 112–1 refer to the posterior portion where the cultriform process merges with the basal plate and whether this is smoothly confluent (as in stereospondyls) or more abrupt (as in non-stereospondyls). 112–2 refers to the anterior portion only, is exemplified by the condition seen in somewhat aberrant taxa like *Zatrachys* (e.g., Schoch, 1997), and would be better placed with character 343, which refers to changes to the width. We make this change here (moving state 112–2 to character 343) to make character 112 encapsulate mutually exclusive states referring only to the posterior region and to make character 343 more inclusive, as taxa like *Zatrachys* could not be previously coded for either state (but were). The new 343–2 is slightly reworded for clarity, and 343–1 removes reference to ‘spatulate,’ as this “flatness” is already captured in character 113 (cultriform process, structure), and 343–0 makes no mention of morphology, only changes in width.
    - Recoded from 112–2 to 112–0: *Balanerpeton, Zatrachys,* and *Hynobius, Cryptobranchus.*
    - Recoded from 112–2 to 112–1: *Platyrhinops, Kokartus,* and *Ambystoma.*
    - Uncoded for 112: *Gerobatrachus* (no basal plate is preserved, and the cultriform process is largely absent).
  - **343. Parasphenoid cultriform process.** Narrow, tapering anteriorly (0), spatulate and parallel-sided (1). [*added by Pardo et al., 2017a*]
    - Recoded from 343–0 to 343–2: *Edops, Acanthostomatops, Zatrachys, Platyrhinops, Amphibamus, Sclerocephalus, Glanochthon*, *Archegosaurus*, *Lydekkerina, Edingerella, Rileymillerus,* and *Triadobatrachus*.
    - Recoded from 343–0 to 343–1: *Micropholis*, *Cacops*, *Plagiosuchus, Benthosuchus*, *Lyrocephaliscus*, and *Callistomordax*.
    - Recoded from 343–1 to 343–2: *Trimerorhachis, Karaurus,* and *Kokartus.*
    - Recoded from 343–1 to 343–0: *Epicrionops*
    - Newly coded for 343: *Micromelerpeton* (Boy, 1995:fig. 3), *Broiliellus* (Williston, 1914:fig. 2).
    - Coding changed to unknown for 343: *Gerobatrachus* (as with 112); *Paracyclotosaurus* (the posterior part of the process is not defined; Watson, 1958:fig. 1).
- **119 (pterygoid, basioccipital); 138 (basioccipital, length):** any taxon without an ossified basioccipital cannot be coded for these characters. Because the absence of an ossified basioccipital may be phylogenetically relevant, a third state is added to character 138 for ‘basioccipital unossified.’
  - Character 119: Codes were corrected from 119–0 (no pterygoid-basioccipital contact) for *Acanthostomatops, Apateon, Micropholis, Platyrhinops, Doleserpeton, Amphibamus, Siderops, Batrachosuchus, Sangaia, Gerrothorax, Benthosuchus, Metoposaurus, Parotosuchus, Triadobatrachus, Leptodactylus, Xenopus, Karaurus, Kokartus, Ambystoma, Hynobius,* and *Cryptobranchus.* Codes were corrected from 119–1 (pterygoid-basioccipital sutured lateral to parasphenoid) for *Rileymillerus.*
  - Character 138: Codes were updated to 138–2 from 138–1 (basioccipital foreshortened to a narrow posterior rim of the palatal bone) for *Acanthostomatops, Apateon, Micropholis, Doleserpeton, Amphibamus, Platyrhinops, Siderops, Batrachosuchus, Sangaia, Gerrothorax, Benthosuchus, Metoposaurus, Parotosuchus, Triadobatrachus*, and *Rileymillerus.*
  - Codes were updated from unknown to 138–2 for *Leptodactylus, Xenopus, Karaurus, Kokartus, Ambystoma, Hynobius,* and *Cryptobranchus*.
- **124 (pterygoid-palatine-ectopterygoid); 125 (palatine, ectopterygoid):** any taxon coded for state 125–1 (palatine with posterolateral process excluding the ectopterygoid from interpterygoid vacuity and contacting pterygoid) is assured of state 124–0 (pterygoid contacting both ectopterygoid and palatine) unless either the palatine or the ectopterygoid is absent, in which case these characters cannot be coded.
  - To remove this dependency, the language of character 125 was altered to remove any mention of the palatine contacting the pterygoid in state 1; a posteromedial process can be present yet not contact the pterygoid, as in *Lydekkerina* (Hewison, 2007). This prevented us from having to change any existing codes for 124–0 to inapplicable. We also clarified state 0 as referring to a straight suture; many taxa have sutures without a posteromedial process that are not transverse but set at sharply oblique angles.
- **128 (palatine, ectopterygoid (ontogeny)); 180 (interclavicle, ontogeny); 190 (humerus, ontogeny):** these characters clearly express ontogeny. Therefore, any taxon for which no ontogenetic trajectory is available (i.e. there is only one specimen with the preserved feature) should not be coded.
  - Character 128: Codes were corrected for *Rhynchonkos, Gerobatrachus, Siderops, Batrachosuchus,* and *Sangaia.*
  - Character 180: Codes were corrected for *Siderops*.
  - Character 190: Codes were corrected for *Trematolestes.*
- **141 (postglenoid area, types):** the classification of postglenoid areas was originally developed by Warren & Black (1985) and Jupp & Warren (1986), specifically for Triassic stereospondyls. The extensive modification of the gymnophionomorph jaw into the pseudoangular means that this classification scheme is inappropriate for such taxa. Future workers may also want to consider whether this binary scheme is inappropriate for non-stereospondyls as well.
  - *Ichthyophis*, *Epicrionops*, and *Eocaecilia* were uncoded from 141–1 (type 2 PGA).
- **144 (preglenoid process):** all taxa coded for 262–2 (surangular absent) cannot be coded for this character because it specifies the labial side of the surangular.
  - Codes were corrected from 144–0 (labial side of surangular with straight dorsal margin anterior to glenoid) for *Triadobatrachus* and *Celtedens*. Codes were changed from 144–1 (labial side of surangular forming dorsal projection well above the level of the glenoid articulation) for *Ambystoma, Hynobius*, and *Cryptobranchus*. Codes were updated from unknown to inapplicable for *Leptodactylus* and *Xenopus*.
- **146 (symphyseal teeth):** a third state was added to differentiate between a parasphymphyseal row set an angle relative to the dentary tooth row and one set parallel to the dentary tooth row.
  - *Ichthyophis, Epicrionops*, and *Eocaecilia* were recoded from 146–1 to the new 146–2.
  - *Doleserpeton* was originally coded as lacking adsymphyseal teeth, but there is clearly a second, lingually situated row on the dentary (Sigurdsen & Bolt, 2010:1366, fig. 6A) that is parallel to the labial row. This taxon is corrected to the new 146–2.
- **147 (posterior coronoid teeth), 148 (anterior, middle coronoid teeth):** as originally coded, both characters were coded for ‘teeth present’ in taxa without “true teeth” on the coronoids, only denticles. The name of these characters is changed to reflect this broader characterization of dentition, as different authors apply the terms ‘teeth’ and ‘denticles’ differently.
- **147 (posterior coronoid dentition):** this character should not be coded for any caecilian because the single coronoid is probably the anteriormost one (or the adsymphyseal (=parasymphyseal plate), as we suggest in Supplementary Information 2 herein) based on embryological data. Since the coronoid itself is absent (captured in character 271, number of coronoids), all caecilians should be coded as inapplicable for this character. Codes were changed from 147–1 (posterior coronoid dentition absent) for *Ichthyophis, Epicrionops*, and *Eocaecilia*.
- **148 (anterior, middle coronoid dentition):** this character follows in a similar vein as above. One of these two coronoids is absent in caecilians (if not both), and since the character specifies both coronoids, the presence of teeth on one coronoid (even if it is the only coronoid) is insufficient to code the presence of teeth. Because these coronoids are more likely candidates for the retained coronoid in caecilians than the posterior coronoid, codes were changed from 148–0 (anterior and middle coronoid dentition present) to inapplicable for *Ichthyophis, Epicrionops*, and *Eocaecilia*.
- **150 (stapes, quadrate process):** this appears to be another character in which codes were reversed for most taxa; *Sclerocephalus* and *Archegosaurus*, which have a well-figured stapes with a quadrate process (Witzmann, 2005:fig. 30; Schoch & Witzmann, 2009a:fig. 6F-G) are coded as lacking one (150–0), whereas taxa like *Edops* and the majority of stereospondyls that lack this process (Schoch, 2017) are coded as having one (150–1). However, some taxa were originally coded correctly (e.g., *Glanochthon* lacks a process and was coded for 150–0 by Schoch, 2013; Schoch & Witzmann, 2009b).
  - Codes were reversed (corrected) from 150–1 to 150–0 for *Edops, Dendrerpeton, Balanerpeton, Trimerorhachis, Acanthostomatops, Micromelerpeton, Apateon, Micropholis, Platyrhinops, Doleserpeton, Amphibamus, Dissorophus, Cacops, Broiliellus, Acheloma, Phonerpeton, Onchiodon, Eryops, Uranocentrodon, Lydekkerina, Siderops, Edingerella, Benthosuchus, Trematosaurus, Trematolestes, Lyrocephaliscus, Metoposaurus, Parotosuchus, Paracyclotosaurus,* and *Mastodonsaurus*.
  - Codes were reversed (corrected) from 150–0 to 150–1 for *Sclerocephalus* and *Archegosaurus.*
  - *Batrachosuchus* was uncoded as there is no mention of the stapes or other parts of the otic capsule (Chernin, 1977).
- **152 (stapes, shape):** mention of the presence of a quadrate process for state 0 was removed, as most taxa coded for state 0 do not have this process (state 150–0).
- **153 (stapes, curvature):** as indicated by the name of this character, the feature being highlighted is curvature of the stapes. 153–1, however, prescribes at least three different attributes: curvature (no curvature), length (“abbreviated”), and the presence of a vertically aligned and laterally situated otic notch. This is problematic because state 0 prescribes only curvature, and there are long, non-curved stapes (e.g., *Mastodonsaurus*) and long, curved stapes not directed towards a vertically aligned otic notch (e.g., *Trimerorhachis*). The character is therefore restricted to merely curvature.
  - Codes were recoded from 153–0 (curvature) to 153–1 (no curvature) for *Edops, Acanthostomatops, Lydekkerina, Siderops, Edingerella, Benthosuchus, Metoposaurus, Parotosuchus,* and *Mastodonsaurus.*
  - Codes were recoded from 153–0 to unknown for *Batrachosuchus* (see character 150); *Trematosaurus* (Schoch, 2019b:53; “besides its presence, there are no further details preserved”).
  - Codes were recoded to 153­–1 (no curvature) for *Brachydectes, Batropetes*, and *Rhynchonkos*.
- **158 (presacral count):** the original three character states (>28; 23–25; <21) do not encompass the full range of possible presacral counts; any taxon with 22, 26, 27, or 28 presacral vertebrae was not captured here. The addition of a state 3 for less than 19 positions does not resolve this. Considering that these states are arbitrarily differentiated, there is no compelling reason to merely leave taxa with these non-captured counts as uncoded (e.g., *Trematolestes* with 22 presacral vertebrae). We therefore modify state 1 to be ‘23–28’ and state 2 to be ‘19–22.’
  - *Batropetes* is recoded from 158–2 to 158–3 (17 positions per Glienke, 2013).
  - *Acanthostomatops* is recoded from 158–1 to 158–2. Witzmann & Schoch (2006:374) stated that “has a rather short trunk composed of 21–22 presacral vertebrae.” This range would not fit within a single character state in the original character (and is thus an unsubstantiated code), but it fits within state 2 of the reformulated version.
  - *Platyrhinops* is largely known from incomplete specimens, but the most complete one has 25–26 presacral positions (Carroll, 1964:234; Clack & Milner, 2010:289). Therefore, this taxon is recoded here to 158–1, rather than 158–2.
  - *Siderops* has 23 presacral positions (Warren & Hutchinson, 1983:26), which means is recoded here to 158–1, rather than 158–2.
  - *Trematolestes* is recoded from 158–1 to 158–2. Regardless of our reformulation, it was originally an unsubstantiated code; Schoch (2006a:36) stated that approximately 22 presacral vertebrae are present.
  - *Callistomordax* is recoded from 158–0 to 158–1. Schoch (2008a:92) states that the presacral count is 26–28, which is not applicable to the original or the new (unchanged) state 0.
  - *Paracyclotosaurus* is recoded from 158–0 to 158–1. Watson (1958:246) identified position 28 as the sacral position, which means there are between 25 and 27 presacral positions depending on whether the atlas and axis are counted; either is not applicable to state 0.
  - *Mastodonsaurus* has long had an uncertain presacral count; historical workers’ estimates ranged from 27–30, which does not fit within a single character state in either the original or revised formulation of this character. Schoch (1999a:93) stated that at least 27 were present and suggested a range of 27–29 (which still does not fall within one character state); he described two specimens, one with 28 positions and one with 30 positions. It is recoded here from 158–0 to 158–0/1, which represents a correction.
- **171 (ribs, length); 296 (ribs anterior to sacrum):** both of these characters relate to rib length, although it is formalized somewhat differently between them (original definitions shown below).
  - **171. Ribs (length).** Moderately elongate thoracic ribs curved distoventrally (0), or such ribs foreshortened without distal curvature (1). [*added by Schoch, 2013*]
  - **296. Ribs anterior to sacrum.** Short (0), long (1). [*added by Pardo et al., 2017a*]

Character 171 is named as if it addresses length, but really it addresses both length and curvature, assuming that they are always correlated. Character 296 only addresses length. Codes for these characters are not the same, either before or after other scoring changes that were made to both; this reflects the inherent subjectivity in discerning ‘long’ versus ‘short’ (or similar distinctions) and that these characters were coded by different workers. Other versions of this character (e.g., character 88 in Schoch, 2018) have invoked a reference point: the length of three vertebral positions. However, presacral rib length is typically variable, being broadest in the anterior half of the trunk in most taxa and tapering sharply towards the sacrum. This is only further complicated by the fact that while most taxa are coded, most are unknown from complete presacral columns or from partial columns that can be precisely positioned. Character 171 is here restricted to curvature, and character 296 is retained for relative length. Both characters were recoded (see further comments below on a pattern of discrepancies within character 296).

- - Codes for character 296 seem to have been inverted; *Gerobatrachus*, for example, has relatively short ribs like in lissamphibians (Anderson et al., 2008b) but was coded for ‘long’ ribs, whereas lissamphibians were coded for ‘short’ ribs. Most temnospondyls have much longer ribs than in lissamphibians, but they were coded for ‘short’ ribs. In order to maintain consistency, the character was recoded for all taxa.
- **179 (interclavicle, length); 180 (interclavicle, ontogeny); 181 (interclavicle, central ornamented area); 182 (interclavicle, proportions); 183 (interclavicle, posterior margin); 184 (interclavicle, anterior margin); 185 (interclavicle, anterior stylus); 186 (interclavicle, posterior stylus); 187 (interclavicle, clavicles):** any taxon coded for 347–1 (interclavicle absent) cannot be coded for these characters.
  - Character 179: Codes were corrected from 179–0 (interclavicle shorter than posterior skull) for *Triadobatrachus* and *Leptodactylus*.
  - Codes for these nine characters were updated from unknown to inapplicable (when originally coded as unknown) for the above taxa as well as for *Xenopus, Celtedens, Karaurus, Kokartus, Ambystoma, Hynobius, Cryptobranchus, Triassurus, Ichthyophis, Epicrionops,* and *Eocaecilia.*
- **186 (interclavicle, posterior stylus):** this character cannot be coded if a stylus is absent (183–1). This holds true even if 186–0 (posterior end rounded or blunt) is intended to be the same as 183–1 (posterior margin transversely straight / without posterior process).
  - Codes were corrected from 186–0 (posterior end rounded or blunt) for *Plagiosuchus* and *Gerrothorax*.
- **203 (pubis):** 203–0 is prescribed for an unossified pubis, with 203–1 for an ossified pubis, but the codes in the matrix were largely reversed for taxa originally coded by Schoch (2013): taxa coded as having an ossified pubis (e.g., *Cacops*, *Eryops*) were coded for 203–0, and taxa described as lacking an ossified pubis (e.g., *Acanthostomatops*, *Glanochthon*) were coded for 203–1, and a few taxa coded were coded correctly (e.g., *Mastodonsaurus*). Conversely, the codes were correct for taxa coded by Pardo et al. (2017a), such as *Cryptobranchus* (pubis unossified, coded for 203–0) and for those coded by Schoch et al. (2020), such as *Celtedens* (pubis ossified, coded for 203–1) and *Triassurus* (pubis unossified, coded for 203–0).
  - Codes were reversed (corrected) for *Proterogyrinus, Greererpeton, Brachydectes, Batropetes, Cochleosaurus, Dendrerpeton, Balanerpeton, Trimerorhachis, Acanthostomatops, Micromelerpeton, Apateon, Micropholis, Doleserpeton, Amphibamus, Gerobatrachus, Dissorophus, Cacops, Broiliellus, Acheloma, Phonerpeton, Onchiodon, Eryops, Sclerocephalus, Glanochthon, Archegosaurus, Uranocentrodon, Lydekkerina, Siderops, Plagiosuchus, Gerrothorax, Trematolestes, Callistomordax,* and *Metoposaurus*.
- **208 (squamation):** this character requires the presence of dermal scales, and taxa without these ossifications cannot be coded. Because these ossifications could hypothetically be lost from even complete skeletons post-mortem, the codes are changed to unknown rather than to inapplicable when they were changed.
  - Codes were recoded from 208–0 (dermal scales oval to spindle-shaped) for *Dissorophus, Cacops, Broiliellus, Siderops, Trematosaurus, Trematolestes, Callistomordax,* and *Metoposaurus* on the basis that no scales are described for these taxa, all of which are known from at least one relatively complete skeleton.
  - Codes are changed from 208–0 for *Benthosuchus* and *Mastodonsaurus* on the basis that scales of these taxa, for which the entire skeleton is known (albeit in disarticulation), are unknown. Less well-represented taxa for which scales have not been positively identified among at least partially known postcrania (e.g., *Edops, Phonerpeton*) are not adjusted following our conservative approach to adjusting codes.
- **210 (osteoderms, articulation); 211 (osteoderms, width):** any taxon coded for state 209–0 (osteoderms absent) cannot be coded for these characters. Corrected codes include taxa coded for 209-0 and as unknown for character 209.
  - Codes were corrected from both 210–0 (simple set of osteoderms arranged in one layer if present) and 211–0 (narrow median osteoderms) for *Proterogyrinus, Greererpeton, Brachydectes, Batropetes*, *Cochleosaurus, Dendrerpeton, Balanerpeton, Trimerorhachis, Acanthostomatops, Micromelerpeton, Apateon, Micropholis, Platyrhinops, Doleserpeton, Amphibamus, Gerobatrachus, Onchiodon, Eryops, Sclerocephalus, Glanochthon, Archegosaurus, Uranocentrodon, Lydekkerina, Siderops, Batrachosuchus, Edingerella, Trematolestes, Callistomordax, Metoposaurus, Paracyclotosaurus, Mastodonsaurus,* and *Celtedens.*
  - *Acheloma* and *Phonerpeton* were coded unsubstantially for 209–1 and should be 209–0; therefore, their codes were also corrected for 210 and 211.
  - *Sangaia* was unsubstantially coded for 209–0 and should be 209–?; therefore, this code was also corrected for 210 and 211.
  - Coded as unknown for 209 but coded for 210–0 and 211–0 and corrected to unknown for 210 and 211: *Edops*, *Zatrachys, Benthosuchus, Trematosaurus, Parotosuchus,* and *Cyclotosaurus.*
- **257 (sphenethmoid); 324 (sphenethmoid); 328 (dorsomedial process of the sphenethmoid); 329 (dorsomedial process of the sphenethmoid); 330 (dorsal sutural surface of the sphenethmoid); 332 (length of lateral walls of sphenethmoid); 333 (floor of sphenethmoid); 341 (anterior wall of sphenethmoid); 342 (anterolateral process of sphenethmoid):** all of these characters require identification of a sphenethmoid in the contemporary framework of tetrapod homologies. Especially in recumbirostran ‘microsaurs,’ this has been debated on the basis of CT analyses (e.g., Maddin et al., 2011; Pardo et al., 2015; Szostakiwskyj et al., 2015; Pardo & Anderson, 2016), and favoring the identification of the paired lateral ossifications in these taxa as orbitosphenoids, not as an unpaired sphenethmoid (as was often done historically; e.g., Carroll & Gaskill, 1978). Therefore, *Brachydectes* and *Rhynchonkos* are recoded as unknown for all characters related to the sphenethmoid (previously they were coded for state 0 for all of the above characters except 330) because CT studies of these taxa have identified paired orbitosphenoids (Szostakiwskyj et al., 2015; Pardo & Anderson, 2016). The more anteriorly extensive ossification found in the brachystelechid *Batropetes* was assumed by Glienke (2015) to be a more anteriorly extensive sphenethmoid, but CT studies of other brachystelechids have shown that there is a short ossification of the mesethmoid anterior to the orbitosphenoid (Maddin et al., 2011; Pardo et al., 2015). Therefore, this taxon is also recoded as unknown for these sphenethmoid characters (previously it was coded for all of the above characters except 330).
- **265 (splenial exposed laterally); 267 (ventral border of Meckel’s fossa); 274 (symphysis); 310 (dorsal margin of splenial); 322 (splenial teeth)**: any taxon without a splenial (264–2) cannot be coded for these characters.
  - *Rhynchonkos* was corrected from 265–0, 267–1, and 274–0 based on restriction of the OTU to the holotype.
  - Character 274: Codes were corrected from 274–1 (symphysis formed by dentary alone) for *Triadobatrachus, Leptodactylus, Xenopus, Celtedens, Ambystoma, Hynobius,* and *Cryptobranchus.*
  - Character 322: Codes were corrected from 322–0 (splenial teeth present) for *Ichthyophis* and *Epicrionops*. Codes were corrected from 322–1 (splenial teeth absent) for *Triadobatrachus, Leptodactylus, Celtedens, Ambystoma, Hynobius,* and *Cryptobranchus*.
  - Codes for these five characters were updated from unknown to inapplicable (when originally coded as unknown) for the above taxa as well as for *Eocaecilia* for consistency.
- **266 (Meckelian fossae):** this character is confusing largely because of the terminology; the openings on the lingual surface of the lower jaw are not typically referred to as ‘fossae’ in temnospondyls but more commonly as ‘fenestrae,’ ‘foramina,’ or ‘windows.’ This can then be complicated by inconsistent use between workers. Some differentiate the large opening found anteroventral to the adductor fossa that is usually framed by some combination of the postsplenial, the angular, and the prearticular in temnospondyls, from additional, smaller openings that are often entirely within the postsplenial (e.g., Sawin, 1941:429–430; Milner & Sequeira, 1993:338; Hewison, 2007:36), whereas others refer to any opening on this surface by the same name regardless of relative size or position (e.g., Langston, 1953:387). Based on how the character was originally coded (e.g., for ‘2 or more’ in taxa with only one relatively large opening but several smaller ones), it seems that any opening on the lower half of the lingual surface (i.e., excluding more dorsally situated ones near the glenoid) is counted as a Meckelian fossa in this instance, and this definition is applied here in newly coding the following taxa:
  - Codes were newly added for *Acanthostomatops, Zatrachys*, *Micromelerpeton, Phonerpeton*, *Glanochthon, Plagiosuchus*, *Trematosaurus, Parotosuchus,* and *Paracyclotosaurus*. Many of these are coded for partial uncertainty, as there is at least one large posterior opening, but the possible presence of additional small openings anteriorly is unclear due to limited exposure or poor preservation.
- **300 (coracoid foramina):** state 2 was changed from ‘2’ to ‘2 or more’ to account for the presence of up to three foramina in *Sclerocephalus* (Boy, 1988).
- **316 (squamosal-frontal contact):** the original coding for this character is peculiar, as this contact is absent in all temnospondyls in which these two elements are defined, but the character was largely recoded as unknown for temnospondyls except for the majority of capitosaurs and trematosaurs. These codes were updated for all taxa where these two elements are defined.
- **318 (quadrate-maxilla):** this is another character for which the omissions are strange. State 1 is only found in gymnophionans, but several ‘lepospondyls’ and temnospondyls were left as unknown: *Brachydectes, Batropetes, Zatrachys, Micromelerpeton, Cacops, Broiliellus*, and *Uranocentrodon*. Of these, the condition is figured or described in all but *Broiliellus,* the holotype of the type species of which has tightly occluded mandibles that obscure the quadrates. All other taxa were newly coded to state 0.
- **319 (quadrate-maxillopalatine):** this is a caecilian character that should not be coded for taxa lacking a maxillopalatine, even if they retain separate maxillae and palatines. A few temnospondyls with known palates were also previously left as uncoded (e.g., *Zatrachys, Micromelerpeton*). Codes were therefore changed to inapplicable for all non-lissamphibians, including *Chinlestegophis,* which lacks a maxillopalatine regardless of the validity of the interpreted fusion of the maxilla and lacrimal in this taxon.
  - Codes were corrected from 319–0 (quadrate and maxillopalatine contact laterally) for *Brachydectes, Batropetes*, and *Rhynchonkos*. Codes were corrected from 319–1 (quadrate and maxillopalatine do not contact laterally) for *Proterogyrinus, Greererpeton, Edops, Cochleosaurus, Dendrerpeton, Balanerpeton, Trimerorhachis, Acanthostomatops, Apateon, Micropholis, Platyrhinops, Doleserpeton, Amphibamus, Gerobatrachus, Dissorophus, Acheloma, Phonerpeton, Onchiodon, Eryops, Sclerocephalus, Glanochthon, Archegosaurus, Lydekkerina, Siderops, Batrachosuchus, Plagiosuchus, Sangaia, Gerrothorax, Edingerella, Benthosuchus, Trematolestes, Lyrocephaliscus, Callistomordax, Parotosuchus, Paracyclotosaurus, Mastodonsaurus, Rileymillerus*, and *Chinlestegophis*.
- **321 (tentacle):** the presence of an osteological correlate for the tentacular organ is a derived condition found in all extant gymnophionans, *Eocaecilia*, and perhaps in *Chinlestegophis*. Extant gymnophionans provide the only examples of the soft-tissue tentacular organ and its osteological correlate; therefore, coding of this feature for fossils in which soft tissue features representing the tentacular organ are not preserved requires the presence of morphology similar to the osteological correlate of this feature in extant gymnophionans. In extant gymnophionans, the osteological correlate for the tentacular organ occupies a single fossa transversing the anteroventral orbital margin of the maxillopalatine or a single aperture in the maxillopalatine anterior to and separate from the orbit; therefore, coding the presence of an osteological correlate in a fossil lacking soft tissue preservation requires the following: the presence of only a single fossa or aperture; and, presence of that fossa or aperture on the anterior orbital margin or anterior to the orbital margin. The presence of multiple branches of the nasolacrimal duct exiting the skull just anterior to the orbital margin in taxa like *Pasawioops mayi* should not be regarded as an osteological correlate for the tentacular organ based on the presence of only a single fossa or aperture for the tentacular organ in extant gymnophionomorphs (Anderson and Bolt, 2013; Pardo et al., 2017a). The character was coded for 321–0 (tentacle absent) in the majority of temnospondyls, but not for any non-metoposaurid trematosaurs or early diverging stereospondyls. This presented as a strange pattern, and there were instances where one member of a clade was coded (for 321–0, absent) but another member of no poorer quality was not (e.g., *Acanthostomatops* was coded but *Zatrachys* was not). The possible correlate of the tentacle that Pardo et al. (2017a) identified in *Chinlestegophis* is a sulcus below the orbit in the position where the nasolacrimal duct normally passes through the lacrimal. Uncoded taxa were generally, but not always, aquatic taxa with lateral line systems, and the coding of some taxa with lateral lines (e.g., *Mastodonsaurus*) and the lack of coding for some without (e.g., *Cacops*) suggests that these cells were not left uncoded because of uncertainty about whether a groove represented a lateral line, the nasolacrimal duct, or a tentacle. The identification of a tentacular correlate in *Chinlestegophis* remains to be further explored (Pardo et al., 2017a, equivocated on the issue and coded it for partial uncertainty).
  - All taxa with sufficiently preserved suborbital regions were newly coded for 321–0 in the absence of the identification of a similar sulcus. This includes *Zatrachys, Micromelerpeton, Cacops, Broiliellus, Uranocentrodon, Lydekkerina, Siderops, Batrachosuchus, Plagiosuchus, Sangaia, Gerrothorax, Edingerella, Benthosuchus, Trematosaurus, Trematolestes, Lyrocephaliscus, Callistomordax, Metoposaurus,* and *Rileymillerus*.
- **324 (sphenethmoid); 328 (dorsomedial process of the sphenethmoid); 329 (dorsomedial process of the sphenethmoid); 330 (dorsal sutural surface of the sphenethmoid); 332 (length of the lateral walls of the sphenethmoid); 341 (anterior wall of sphenethmoid); 342 (anterolateral process of sphenethmoid)**: any taxon coded for 257–1 (sphenethmoid unossified) cannot be coded for these characters.
  - Character 324: Codes were corrected from 324–0 (sphenethmoid covered dorsally) for *Acanthostomatops, Apateon,* and *Amphibamus*.
  - Codes for these seven characters were updated from unknown to inapplicable (when originally coded as unknown) for the above taxa as well as for *Siderops, Batrachosuchus, Trematolestes, Lyrocephaliscus, Callistomordax*, *Rileymillerus,* and *Chinlestegophis* for consistency.
- **325 (larval stage):** the original coding for this character is difficult to interpret and therein to apply to newly added taxa. Pardo et al. (2017a) originally coded most lissamphibians and a number of European temnospondyls with well-sampled ontogenies (e.g., *Micromelerpeton*, *Sclerocephalus*) as having larval stages (325–0). Conversely, *Gerrothorax, Trematolestes, Callistomordax*, and *Mastodonsaurus* were coded as having direct development (325–1). Schoch et al. (2020) coded direct development in the three ‘lepospondyls’ as well. The stereospondyls coded for 325–1 appear entirely random. At least one, *Trematolestes*, is actually stated to have a larval form (Schoch, 2006:38). Literature on the other three makes no mention of direct development, which would be unexpected in these obligately aquatic, paedomorphic taxa, and arguably it cannot be well-assessed in at least *Callistomordax* based on the narrow known ontogenetic range, (Schoch, 2008b). The character does not prescribe anatomical features considered to be unequivocal evidence of larval stages (e.g., Boy & Sues, 2000).
  - *Trematolestes* is recoded to 325­–0, and the other three stereospondyls are recoded as unknown.
  - *Batropetes* was modified in line with the substitution of *B. palatinus*, and *Rhynchonkos* was modified in line with the restriction of the OTU to the holotype; these changes were noted in the taxon-specific section.

The following are characters with partial dependencies that did not result in any coding changes but for which cells coded as ‘?’ were updated to ‘-‘ for consistency.

- **175–177 (cleithrum):** any taxon coded for 348–1 (cleithrum absent) cannot be coded for these characters. Codes updated for: *Triadobatrachus, Leptodactylus, Xenopus, Celtedens, Karaurus, Kokartus, Ambystoma, Hynobius, Cryptobranchus, Triassurus, Ichthyophis, Epicrionops,* and *Eocaecilia.*
- **309 (number of distal tarsals):** any taxon coded for 206–1 (tarsals all unossified) cannot be coded for this character. Codes updated for: *Trimerorhachis, Acanthostomatops, Micromelerpeton, Apateon, Sclerocephalus, Glanochthon, Siderops, Plagiosuchus, Gerrothorax, Trematolestes, Callistomordax, Metoposaurus, Paracyclotosaurus, Mastodonsaurus,* and *Karaurus*.

**Taxon-specific changes.** As with the above section, taxa are referred to only by the genus and should be assumed to be the species sampled in the matrix (for multi-species genera) unless otherwise stated (e.g., a reference to *Eryops* is specifically to *Eryops megacephalus*).

- ***Proterogyrinus scheelei***
  - **24 (lacrimal, posterior extension):** 0 → ?. Holmes (1984:447, fig. 3) indicated that the suture between the lacrimal and the jugal is entirely unknown, and there is no comment on the anterior extent of the latter in the text. As reconstructed by Holmes, the lacrimal would extend past the antorbital region (24–1).
  - **35 (infraorbital sulcus):** 0 → ?. Holmes (1984:440–441) noted that the grooves are poorly developed and only appear on the “the postfrontal, postorbital, the suborbital ramus of the jugal, and on the lateral edge of the nasal.” In taxa with a lacrimal, like *Proterogyrinus*, any flexure of the infraorbital sulcus would occur on the lacrimal, which does not have any groove per Holmes’ description.
  - **57 (jugal, ventral process):** 0 → 1. Holmes (1984:453) stated that “the relation between the posterior end of the ectopterygoid and the jugal is not clear in any of the articulated specimens. However, in CMNH 11067 the right jugal clearly shows a modest processus alaris that would almost certainly have made contact with the pterygoid mesially and excluded the ectopterygoid from the rim of the adductor chamber, as it does in *Eogyrinus* and *Palaeoherpeton*.” Although a ventral exposure of the jugal is typically thought of as a stereospondyl feature, it occurs also in *Cochleosaurus* and *Eryops* (and was originally coded as such).
  - **58 (jugal, anterior extension):** 0 → ?. As with character 24.
  - **59 (jugal-lacrimal):** 0 → ?. As with character 24. While a LEP (or LEE) would be highly unexpected in an embolomere, it cannot be assumed that one was absent because this taxon is an embolomere (circular logic).
  - **74 (posttemporal fenestra):** 0 → -. There are no openings homologous with the posttemporal fenestrae of temnospondyls in which it is enclosed by the tabular, the postparietal, and the exocciptal.
  - **85 (transvomerine tooth row):** 0 → ?. Holmes (1984:452) stated that there is no identifiable vomer in any specimen, and the only putative fragment of a vomer confers practically no information.
  - **86 (additional vomerine fangs):** 0 → ?. As with character 85. Alternatively, this could be coded as ‘-‘ based on Holmes’ inference that there were no vomerine fangs because these are often large in early tetrapods, and there was no evidence for such fangs in material of this taxon.
  - **93 (vomer):** 0 → ?. As with character 85.
  - **94 (vomer, paired anterior depressions):** 0 → ?. As with character 85.
  - **96 (vomerine ridges):** 0 → ?. As with character 85.
  - **97 (vomerine pit and fontanelle):** 0 → ?. As with character 85.
  - **98 (vomerine septum):** 0 → ?. As with character 85.
  - **100 (choana, medial):** 0 → ?. As with character 85.
  - **101 (choana, width):** 0 → ?. As with character 85.
  - **103 (vomer, anterior part):** 0 → ?. As with character 85.
  - **120 (pterygoid, squamosal):** 0 → -. Holmes (1984:453–454, fig. 4) showed that it is the epipterygoid, not the pterygoid, that sutures to the skull roof. There is therefore neither pterygoid-squamosal contact (120–0) nor an open fissure (120–1).
  - **126 (palatine, vomer):** 0 → ?. As with character 85.
  - **127 (LEP):** 0 → ?. As with character 24.
  - **144 (preglenoid process):** 0 → ?. This refers to the dorsal margin of the coronoid process as formed by the surangular (i.e. is it mostly flat or convex); Holmes (1984:460, figs. 14–15) stated that there is a prominent dorsal process and reconstructed a markedly convex margin (144–1), but the latter morphology is only inferred.
  - **145 (Meckelian window):** 0 → 1/2. Holmes (1984:461–462, fig. 15) indicated that this region of the lingual surface is not well-preserved, but it was reconstructed based on partial preservation of a “large” window.
  - **150 (stapes, quadrate process):** 0 → ?. Holmes (1984) did not describe any stapes, fragmentary or otherwise, and Clack et al. (2016:84) noted that stapes are found in only two embolomeres: *Palaeoherpeton* and *Pholiderpeton*.
  - **151 (stapes, ventral process):** 0 → ?. As with character 150.
  - **152 (stapes, shape):** 0 → ?. As with character 150.
  - **153 (stapes, curvature):** 0 → ?. As with character 150.
  - **173 (ribs, uncinate blades):** 0 → -. This character requires the presence of uncinate blades, which are absent.
  - **174 (ribs, uncinate spines):** 0 → -. As with character 173.
  - **196 (carpals):** 0 → 1. Holmes (1984:481, fig. 28) depicted two ossified distal carpals.
  - **266 (Meckelian fossa):** ? → 0/1. Holmes (1984:461–462) noted one anterior fossa near the anterior end of the prearticular. He tentatively suggested there was a second one more posteriorly, which is typical for early tetrapods, but the degree of confidence expressed is not sufficient to code it exclusively for 266–0 (two fossae).
  - **312 (parietal width):** ? → 0. As shown by Holmes (1984:figs. 5–6).
  - **349 (vomer):** 0 → ?. As with character 85.
- ***Greererpeton burkemorani***
- 15 (snout, internarial distance): 0 🡪 1. The nares of this taxon are not exposed dorsally in adults (this is an ontogenetic shift; Godfrey, 1989), and consequently, the interorbital distance is much narrower (see also, Smithson, 1982).
- **24 (lacrimal, posterior extension):** 0 → 1. As shown and described by Romer (1969:figs. 2–3) and Smithson (1982:44, figs. 5, 7), the lacrimal forms a substantial portion of the lateral orbital margin. This is slightly reduced throughout ontogeny (Godfrey et al., 1989:fig. 4), but the posterior terminus of the lacrimal remains well posterior to the level of the anterior orbital margin.
- **45 (prefrontal-maxilla):** 0 → 1. There is a prominent contact between these elements (Romer, 1969:figs. 2–3; Smithson, 1982:44, fig. 9; Godfrey, 1989:fig. 4).
- **120 (pterygoid, squamosal):** 0 → -. It is the epipterygoid, not the pterygoid, that sutures to the skull roof. There is therefore neither pterygoid-squamosal contact nor an open fissure (Smithson, 1982:42, 44, fig. 12).
- **196 (carpals):** 0 → 1. Godfrey (1989:109) made brief mention of ossified carpals.
- **267 (ventral border of Meckel’s fossa):** 0 → 0&1. This taxon has an unusually large Meckelian fossa, which is largely bordered ventrally by the postsplenial, with contributes on the ends by both the splenial (267–0) and the angular (267–1) (e.g., Smithson, 1982:fig. 19; Bolt & Lombard, 2001:figs. 2, 4–5).
- ***Brachydectes newberryi***

*Note that these changes are considered to be applicable regardless of whether the more restrictive concept of ‘*B. elongatus*’ (sensu Wellstead, 1991) or the more expansive concept of *B. newberryi* (sensu Pardo & Anderson, 2016) is employed.

- **15 (internarial distance):** 1 🡪 0. The nares approach each other very closely on the dorsal surface of the skull (e.g., Pardo & Anderson, 2016:fig. 4B).
- **17 (naris, position):** 0 🡪 1. As with character 15.
- **24 (lacrimal, posterior extension):** 0 → 1. As shown by Pardo & Anderson (2016:figs. 3C, 4A), the lacrimal forms the anterolateral corner of the orbit and extends beyond the antorbital region.
- **26 (orbit location):** 1 → -. This taxon has no jugal.
- **30 (interorbital distance):** 0 → 1. The interorbital region is wider than the orbits, even when accounting for the laterally facing orientation (e.g., Pardo & Anderson, 2016:figs. 3–4).
- **44 (prefrontal-jugal):** 0 → -. As with character 26.
- **51 (otic notch):** 0 → 1. ‘Microsaurs’ lack any form of otic notch or squamosal embayment (Wellstead, 1991; Pardo & Anderson, 2016).
- **59 (jugal-lacrimal):** 1 → -. As with character 26.
- **74 (posttemporal fenestra):** 0 → -. There are no openings homologous with the posttemporal fenestrae of temnospondyls.
- **89 (interpterygoid vacuities):** 0 🡪 -. There are no interpterygoid vacuities in this taxon.
- **90 (interpterygoid vacuities, pterygoid):** 0 🡪 -. As with character 89.
- **105 (basipterygoid ramus):** 0 🡪 -. As with character 89.
- **106 (basicranium, contact):** 0 🡪 1. The pterygoid and parasphenoid have a long abutting contact.
- **120 (pterygoid-squamosal):** 0 → 1. The pterygoid is not a vaulted element in ‘microsaurs,’ and therefore there is a large separation between the pterygoid and the skull roof.
- **147 (posterior coronoid teeth):** 0 → 1. Pardo & Anderson (2016:18–19) described only one coronoid (of uncertain homology among the three present in most temnospondyls) and it has no teeth. Here, the coronoid of this taxon is treated as the posterior coronoid to capture the edentulous state.
- **148 (anterior, middle coronoid teeth):** 0 → -. As with character 147; these coronoids are considered to be entirely absent here and are therefore not codeable.
  - **173 (ribs, uncinate blades):** 0 → -. This character requires the presence of uncinate blades, which are absent.
  - **174 (ribs, uncinate spines):** 0 → -. As with character 173.
  - **188 (scapula, glenoid facet):** 0 🡪 1. Wellstead (1991:12) listed the absence of an ossified glenoid as an apomorphy of lysorophians.
- **192 (humerus, entepicondylar foramen):** 0 → 1. Wellstead (1991:43, fig. 21) indicated the absence of this foramen.
- **206 (tarsals):** 0 🡪 1. No tarsals have been previously described, and given the lack of an ossified glenoid facet and an ossified pubis, it can be reasonably inferred that they were biologically absent from articulated skeletons.
- **225 (septomaxilla):** ? → 0. Considering that nearly every other taxon was originally coded, there is no reason to leave this taxon uncoded when numerous nearly complete skulls are known, with no reported or reconstructed septomaxilla.
- **238 (squamosal-jugal):** 1 🡪 -. As with character 26.
- **246 (supraoccipital):** 0 → 1. Pardo & Anderson (2016:15, figs. 3–7) described a supraoccipital, which was previously identified by Case (1908:fig. 1); Williston (1908:fig. 2); Sollas (1920:493–494, figs. 5–7, 12–13); Bolt & Wassersug (1975:fig. 4) and Wellstead (1991:12, 14, 17–18, 61, figs. 7–10, 13, 15), among others.
- **251 (vomer, denticles):** 0 🡪 1. There are only a series of large teeth on the vomer (Pardo & Anderson, 2016:16).
- **254 (cultriform process-vomer):** 0 🡪 1. These elements have a broad overlapping contact.
- **258 (pterygoids, contact):** 0 🡪 1. The pterygoids are well-separated.
- **261 (dentary, coronoid process):** 0 🡪 1. The dentary forms the anterior portion of the process (Wellstead, 1991; Pardo & Anderson, 2016).
- **267 (ventral border of Meckel’s fossa):** 1 → -. There is no splenial and no Meckelian fossa on the lingual surface, contrary to other tetrapods sampled here.
- **271 (number of coronoids):** ? → 2. As with character 147.
- **272 (coronoid teeth):** ? → 1. As with character 147.
- **274 (symphysis):** 1 → -. As with character 267.
- **298 (caudal ribs, count):** ? 🡪 3. There is no evidence for caudal ribs in articulated caudal sequences (Wellstead, 1991).
- **299 (scapula, supraglenoid foramen):** ? 🡪 1. As described by Wellstead (1991:24).
- **300 (coracoid, number of coracoid foramina):** ? 🡪 0. As with character 299.
- **306 (basale commune):** 0 🡪 -. Following character 196.
- **308 (elongate tibiale and fibulare):** 0 🡪 -. As with character 206.
- **335 (os basale, dorsal exposure):** 0 🡪 -. There is no os basale in this taxon.
- **355 (palatine with distinct tusk pair):** 0 → -. Pardo & Anderson (2016) stated that no teeth or denticles occur on the palatine.
- **359 (squamosal, shape):** 0 🡪 ?. The squamosal of lysorophians is highly modified around the temporal opening such that it does not cleanly fit into either of the prescribed states.
- ***Batropetes palatinus***

*****Note that the following coding changes are made with respect to *Batropetes palatinus* but are noted here because they were unsubstantiated when treated as *Batropetes fritschi*.

- **1 (skull outline):** 0 🡪 1. The skull of this taxon is distinctly triangular (e.g., Carroll, 1991; Glienke, 2013, 2015).
- **8 (spines):** 0 → -. Spines, as in zatracheids, occur on the quadratojugal (as specified by 8–1), which is determinable but not properly codeable in this taxon in the absence of a quadratojugal (Carroll & Gaskill, 1978:140; Carroll, 1991:234).
- **12 (premaxilla, outline):** 0 → ?. The anterior margin in *Batropetes fritschi* was entirely reconstructed by Glienke (2013:fig. 6), in part because these elements readily detach from the roof in taxa with recumbent snouts. As reconstructed for *Batropetes palatinus* (Glienke, 2015:fig. 1), it favors characterization as 12–1 (box-like and anteriorly blunt).
- **30 (interorbital distance):** 0 → 1. The orbits are proportionately narrow, even when viewed laterally (Carroll, 1991:figs. 4–5; Glienke, 2013:fig. 6; Glienke, 2015:fig. 1).
- **51 (otic notch):** 0 → 1. ‘Microsaurs’ lack any form of otic notch or squamosal embayment (e.g., Carroll & Gaskill, 1978; Carroll, 1991; Glienke, 2013, 2015).
- **59 (jugal-lacrimal):** 1 🡪 0. These elements share a broad contact in all species of *Batropetes*.
- **60 (intertemporal):** 0 → 1. An intertemporal is definitively absent, as in all ‘microsaurs.’
- **61 (parietal-postorbital):** 0 🡪 1. There is a broad contact associated with the laterally expanded parietal.
- **69 (quadratojugal-maxilla):** 1 → -. As with character 8; note also that character 68 (quadratojugal, medial process) was originally uncoded.
- **74 (posttemporal fenestra):** 0 → -. There are no openings homologous with the posttemporal fenestrae of temnospondyls.
- **80 (palatal tusks, cross-section):** 0 → ?. The palate of *Batropetes fritschi* was stated to be poorly preserved, is described in two sentences, is not reconstructed, and makes no comments on dentition (Glienke, 2013:86). It is slightly better preserved in *Batropetes palatinus* but not sufficiently so to code this character (Glienke, 2015:9–10, fig. 3).
- **83 (bicuspidity):** 1 🡪 -. Brachystelechids are well-known for having tricuspid dentition, which is not covered by this character.
- **86 (additional vomerine fangs):** 0 → ?. There is no comment on whether any pair of vomerine fangs are present, so it cannot be determined whether “additional” fangs were present (Carroll & Gaskill, 1978; Carroll, 1991; Glienke, 2013, 2015).
- **88 (ectopterygoid, fangs):** 0 → ?. No ectopterygoid is described.
- **98 (vomer, septum):** 0 🡪 ?. In addition to the poor preservation of the vomer in this particular taxon, the compression and disarticulation of all specimens of *Batropetes* precludes confident determination of this feature.
- **102 (vomer, pterygoid):** 0 → ?. These elements are not sufficiently preserved to code this.
- **104 (vomer, extension):** 0 → ?. As with character 102.
- **120 (pterygoid-squamosal):** 0 → ?. Crushing of the skulls prevents any confident interpretation of this condition, although the pterygoid is not typically dorsally vaulted in ‘microsaurs.’
- **123 (pterygoid, ectopterygoid):** 0 → ?. As with character 88.
- **124 (pterygoid-palatine-ectopterygoid):** 0 → ?. As with character 88.
- **125 (palatine, ectopterygoid):** 0 → ?. As with character 88.
- **126 (palatine, vomer):** 0 → ?. This suture is not preserved.
- **128 (palatine, ectopterygoid, ontogeny):** 0 → ?. As with character 88.
- **129 (palatine, ectopterygoid, width):** 0 → ?. As with character 88.
- **130 (palatine, ectopterygoid, continuous tooth row):** 0 → ?. As with character 88.
- **131 (ectopterygoid, length):** 0 → ?. As with character 88.
- **133 (ectopterygoid, Y-shaped):** 0 → ?. As with character 88.
- **173 (ribs, uncinate blades):** 0 → -. There are no uncinate blades.
- **174 (ribs, uncinate spines):** 0 → -. There are no uncinate spines.
  - **187 (interclavicle, clavicles):** 0 🡪 1. The reconstruction by Glienke (2015) depicted a very narrow separation of the clavicles.
- **212 (rib cage):** 0 → 1. Brachystelechids consistently display wide trunks (e.g., Carroll, 1991:fig. 3).
- **240 (maxilla into external naris):** 0 → 1. Reconstruction by Glienke (2013, 2015) with a septomaxilla inferred from other species indicates the maxilla does not enter the naris.
- **241 (maxilla forms entire ventral naris):** 0 → ?. As with character 240.
- **246 (supraoccipital):** 0 → 1. Carroll (1991:235) and Glienke (2015:10) described a supraoccipital in *Batropetes palatinus*; this ossification is also present in *Batropetes fritschi* (Glienke, 2013:86, fig. 6).
- **248 (anterior palatine):** 0 → ?. As with character 126; the palatine is also essentially undefined.
- **249 (denticles on pterygoid):** 0 → 1. Glienke (2015:9) stated that there appear to be no denticles except on the parasphenoid; as with character 80 for *Batropetes fritschi*.
- **251 (denticles on vomers):** 0 → 1. As with character 249.
- **270 (angular extends to lateral view):** 0 → -. Glienke (2015:10) stated that the angular does not extend to the posterior end of the tooth row, which is also evident in her reconstruction of *Batropetes fritschi* (Glienke, 2013:fig. 7Q).
- **304 (radius:humerus length):** 0 🡪 2. The radius is much shorter than the humerus in *Batropetes*. For this particular species, Glienke (2015:12) noted that the ulna was about half three length of the humerus, and the radius is shorter than the ulna.
- **305 (olecranon process):** 0 → 1. Glienke (2013, 2015) noted the presence of a short triangular olecranon process in both species.
- **313 (skull table):** 0 🡪 1. By comparison with other taxa coded for state 1, this state is more appropriate for this taxon.
- **326 (anterior dentary teeth):** 1 → -. The dentary teeth are tricuspid (Glienke, 2013, 2015).
  - **327 (nasal septum ossification):** 0 🡪 2. Previous workers identified what appeared to be an anteriorly extensive sphenethmoid that has a palatal exposure in the snout region (e.g., Carroll, 1991; Glienke, 2013, 2015), but CT analysis of the brachystelechids *Carrolla* and *Quasicaecilia* has identified separate ossifications present in the nasal region (Maddin et al., 2011; Pardo et al., 2015). Based on this interpretation, the sphenethmoid is unknown in this taxon, which results in the associated coding changes:
    - **328 (sphenethmoid, dorsomedial process, length):** 0 🡪 ?
    - **329 (sphenethmoid, dorsomedial process, thickness):** 0 🡪 ?
    - **331 (sola nasi):** 0 🡪 ?
    - **332 (sphenethmoid, lateral walls, length):** 0 🡪 ?
    - **333 (sphenethmoid, floor):** 0 🡪 ?
    - **341 (sphenethmoid, anterior wall):** 0 🡪 ?
    - **342 (sphenethmoid, anterolateral process):** 0 🡪 ?
- **346 (ectopterygoid):** 0 → ?. As with character 88.
- **351 (continuous tooth row lateral to choana):** 0 → ?. The lateral margins are not preserved.
- **354 (squamosal posterior extent relative to quadrate and quadratojugal):** 0 → -. As with character 8.
- **355 (palatine with distinct tusk pair):** 0 → ?. As with character 80.
- **358 (neurocranium):** 0 → 1. Brachystelechids are well-known for cranial consolidation into an os basale (Carroll, 1990; 1991; Maddin et al., 2011; Glienke, 2013, 2015; Pardo et al., 2015).
- ***Rhynchonkos stovalli***
- **24 (lacrimal, posterior extension):** 1 → 0. The lacrimal only forms the anterior orbital margin without a suborbital process, with the maxilla forming the entire ventral/lateral margin (Carroll & Gaskill, 1978:fig. 63A; Szostakiwskyj et al., 2015:9, fig. 1).
- **51 (otic notch):** 0 → 1. ‘Microsaurs’ lack any form of otic notch or squamosal embayment (Carroll & Gaskill, 1978; Szostakiwskyj et al., 2015).
- **60 (intertemporal):** 0 → 1. An intertemporal is definitively absent, as in all ‘microsaurs.’
- **74 (posttemporal fenestra):** 0 → -. There are no openings homologous with the posttemporal fenestrae of temnospondyls.
- **98 (vomer, septum):** 0 🡪 1. Szostakiwskyj et al. (2015:9) described a median vaulted septum.
- **120 (pterygoid-squamosal):** 0 → 1. The pterygoid is not a vaulted element in ‘microsaurs,’ and therefore there is a large separation between the pterygoid and the skull roof.
- **147 (posterior coronoid dentition):** 0 → 1. As with *Brachydectes*, the reduction in number of coronoids creates uncertain homology with the three coronoids present in most temnospondyls, and neither of the coronoids in *Rhynchonkos* have teeth. Here, the posterior coronoid of this taxon is treated as the homologue of the posterior coronoid in temnospondyls to capture the edentulous state.
- **148 (anterior, middle coronoid dentition):** 0 → -. As with character 147. The anterior coronoid of this taxon cannot be homologized with both coronoids.
- **213 (basal skull length):** ? → 3. This is a very small taxon (Carroll & Gaskill, 1976; Szostakiwskyj et al., 2015).
- **222 (quadratojugal):** 0 🡪 1. Contrary to the reconstruction by Carroll & Gaskill (1978), no quadratojugal is found in the nearly complete and undistorted holotype; this is considered sufficient for coding it as unknown. Quadratojugals have also not been confidently identified in specimens previously referred to this taxon (e.g., holotypes of *Aletrimyti gaskillae* and *Dvellecanus carrolli*).
- **246 (supraoccipital):** 0 → 1. Carroll & Gaskill (1978:107:fig. 63) and Szostakiwskyj et al. (2015:13–14, figs. 1, 4, 6) described and figured a supraoccipital.
- **265 (splenial, lateral exposure):** 0 🡪 -. There is no splenial (Szostakiwskyj et al., 2015).
- **267 (Meckelian window, ventral border):** 1 🡪 -. As with character 265.
- **274 (symphysis):** 0 🡪 -. As with character 265.
- **329 (sphenethmoid, dorsomedial process, thickness):** 0 🡪 -.
- **335 (os basale, dorsal exposure):** 0 🡪 -. There is no os basale in this taxon.
- **355 (palatine, fangs):** 0 🡪 ?. There is no dentition in the region of the palatine where ‘fangs’ occur in temnospondyls (anteromedial to the choana).
- ***Edops craigi***
- **31 (frontal-nasal, length):** 0 → 1. Whether measured along the midline or as the greatest length of the respective elements, the nasal is longer than the frontal (Romer & Witter, 1942:figs. 1–2; Schoch & Milner, 2014:fig. 13A).
- **146 (symphyseal teeth):** ? → 0. Romer & Witter (1942:949, fig. 3) depicted only a pair of symphyseal tusks posterior to the dentary row.
- **147 (posterior coronoid dentition):** ? → 0. Romer & Witter (1942:949) noted a covering of denticles all on three coronoids.
- **148 (anterior, middle coronoid dentition):** ? → 0. As with character 147.
- **168 (pleurocentrum, presence):** ? → 0. Romer & Witter (1942:952) described pleurocentra.
- **192 (humerus, entepicondylar foramen):** 0 → 1. A foramen is absent (Romer & Witter, 1942:fig. 13).
- **214 (skull to trunk):** 1 → ?. Vertebral material of this taxon is isolated, and the length of the trunk is unknown (Romer & Witter, 1942:951–952). This code may have been accidentally entered for this taxon instead of for *Cochelosaurus bohemicus* (one line below), which has a well-characterized skeleton and which was not originally coded.
- ***Cochleosaurus bohemicus***
  - **121 (pterygoid, flange):** 0 🡪 1. Sequeira (2004:30, fig. 7) made note of a prominent posterolateral flange.
- **192 (humerus, entepicondylar foramen):** 0 → 1. The loss of the foramen is an ontogenetic feature (Sequeira, 2009:147).
- **205 (femur, trochanter):** ? → 0. Sequeira (2009:148) notes a small trochanter.
- **214 (skull to trunk):** ? → 1. Reconstructions of the skeleton have been published by Schoch & Milner (2014:fig. 8B).
- **278 (trunk intercentra):** ? → 0. As described by Sequeira (2009:142).
- **292 (atlas neural arch):** ? → 0. As described by Sequeira (2009:142).
- **293 (proatlantes):** ? → 0. As described by Sequeira (2009:142).
- **305 (olecranon process):** ? → 1. As described by Sequeira (2009:147).
- **311 (parietal, anterior waisting):** ? → 0&1. As figured by Sequeira (2003:figs. 1, 4–6).
- **312 (parietal width):** ? → 1. As with character 311.
- ***Dendrerpeton helogenes***
- **147 (posterior coronoid dentition):** ? → 0. Godfrey et al. (1987:801) stated that the coronoids are covered by denticles.
- **148 (anterior, middle coronoid dentition):** ? → 0. As with character 147.
- ***Balanerpeton woodi***
  - **121 (pterygoid, flange):** 0 🡪 1. The posterolateral margin is distinctly convex (Milner & & Sequeira, 1993:fig. 5A).
- ***Trimerorhachis insignis***
- **30 (interorbital distance):** 0 → 0&1. There is apparently intraspecific variation in this feature (e.g., Milner & Schoch, 2013:fig. 2); reconstructions depict an interorbital distance that is wider than the orbit.
- **39 (prefrontal, frontal):** 1 → 0&1. This is polymorphic (e.g., Milner & Schoch, 2013:figs. 1–2).
- **52 (otic notch, position):** 0 → 2. The otic notch of this taxon is not well-developed and is closer to the condition observed in stereospondyls (dorsally facing and forming a relatively short incision into the squamosal) than to other taxa coded for 52­–0, such as dissorophoids or dendrerpetids, and it is clearly not “slit-like” as in eryopoids (52–1).
- **148 (anterior, middle coronoid teeth):** 1 → ?. While teeth are absent on the anterior coronoid, Milner & Schoch (2013:107) stated that there is a raised denticle field on the second (middle) coronoid. Based on how other taxa were coded, the presence of denticles is considered to be valid for presence of ‘teeth.’ This character is coded as unknown rather than as polymorphic because of how the character is constructed.
- **244 (maxillary tooth count):** 1 → 0. Schoch & Milner (2013:106), citing Case (1935:237), noted room for up to 60 maxillary teeth.
- **281 (haemal arches):** ? → 0. Pawley (2007:876) made brief mention of these features.
- **286 (transverse processes):** ? → 0. Pawley (2007:fig. 5) illustrated these processes on the arch.
- **290 (atlas neural arch):** ? → 0. Pawley (2007:875) indicated there is no fusion between components of the atlas.
- **292 (atlas neural arch):** ? → 0/1. Pawley (2007:875) indicated the two counterparts are “not fused” to each other.
- **295 (cervical rib distal shape):** ? → 0. Pawley (2007:876, fig. 4) indicated distally expanded ribs to form a more ‘spatulate’ shape.
- **302 (deltopectoral crest):** ? → 1/2. Termed the ‘deltoid’ and ‘pectoral’ crests by Pawley (2007:881), these are “well-developed” and “rugose.”
- **305 (olecranon process):** ? → 1. Pawley (2007:883, fig. 10) described and figured a moderately developed process.
- **307 (femur):** ? → 0. As figured by Pawley (2007:figs. 12–13). This is clearly a subjective feature with ‘long’ and ‘short’ states; the coding here is by comparison with previously coded taxa and is at least more elongate than other taxa coded for 307–0 like *Acanthostomatops*.
- **322 (splenial teeth):** 0 → 1. No previous worker has described or figured teeth on the splenial (e.g., Williston, 1913; Case, 1935; Milner & Schoch, 2013).
- ***Acanthostomatops vorax***
- **39 (prefrontal, frontal):** 1 → 0. The prefrontal terminates well anterior to the frontal (Boy, 1989:fig. 1; Witzmann & Schoch, 2006:figs. 2–3).
- **73 (postparietal-exoccipital):** 1 → - Witzmann & Schoch (2006:373) indicated the total absence of ossified exoccipitals and basioccipitals.
- **74 (postfenestral window):** 0 → ?. As with character 73.
- **75 (quadrate and occipital condyles):** 1 → -. As with character 73.
- **76 (epipterygoid):** 0 → -. Witzmann & Schoch (2006:373) indicated the absence of an ossified epipterygoid.
- **108 (parasphenoid):** 0 → -. As with character 73.
- **118 (pterygoid, exoccipital):** 0 → -. As with character 73.
- **121 (pterygoid, flange):** 0 → 1. Boy (1989:fig. 2) and Witzmann & Schoch (2006:fig. 4) figured pterygoids with a prominent posterolateral expansion of the palatine ramus into the subtemporal fenestra.
- **137 (exoccipital condyles):** 0 → -. As with character 73.
- **145 (Meckelian window):** 0 → 1. Witzmann & Schoch (2006:374) estimated the length of the posterior Meckelian window to be one-sixth of the length of the lower jaw, which is certainly much larger than the smaller openings found in taxa like *Balanerpeton* or *Trimerorhachis* (145–0).
- **266 (Meckelian fossae):** ? → 0/1. As with character 145. This is expressed as partial uncertainty because the anteromedial surface is not as well-preserved (Boy, 1989:fig. 3B), so the presence of additional fossae is unclear.
- **305 (olecranon process):** ? → 0. Witzmann & Schoch (2006:378) stated that an olecranon process is absent.
- ***Zatrachys serratus***
- **11 (prenarial region):** 0 → 1. As also seen in the larger zatracheid *Dasyceps* (not sampled here; Paton, 1975:figs. 1, 5), the prenarial region is at least as long if not longer than the frontal (contrary to *Acanthostomatops* and to most other temnospondyls) (Langston, 1953:fig. 13; Schoch, 1997:fig. 2).
- **24 (lacrimal, posterior extension):** 0 → 1. As shown by Langston (1953:fig. 13) and Schoch (1997:fig. 1), the lacrimal forms a moderate portion of the lateral orbital margin.
- **39 (prefrontal, frontal):** 1 → 0. The prefrontal terminates well anterior to the frontal (Schoch, 1997:fig. 2).
- **102 (vomer-pterygoid):** 0 → 0&1. Schoch (1997:233, fig. 3) noted a specimen in which the elements are separated, but other specimens appear to feature this contact (Langston, 1953:fig. 13) .
- **121 (pterygoid, flange):** 0 → 1. Langston (1953:fig. 13) and Schoch (1997:fig. 3) figured pterygoids with a prominent posterolateral expansion of the palatine ramus into the subtemporal fenestra.
- **147 (posterior coronoid dentition):** ? → 0. Langston (1953:386–388, fig. 15) described the lower jaw and its dentition.
- **148 (anterior, middle coronoid dentition):** ? → 0. As with character 147.
- **149 (mandibular osteoderms):** 0 → 1. Schoch (1997:fig. 4) depicted “ventral osteoderms” on the palate of a skull, which Schoch & Rubidge (2005:510) cite as the same gular osteoderms that are found in *Micropholis*.
- **153 (stapes, curvature):** ? → 0. Langston (1953:388) stated that this bone is long and curved.
- **202 (ilium, tip):** 1 → ?. Postcrania are entirely unknown for this taxon, as reflected in other postcranial codes (Langston, 1953; Schoch & Milner, 2014).
- **208 (squamation):** 0 → ?. As with character 202.
- **214 (skull to trunk):** 0 → ?. As with character 202.
- **258 (pterygoids contact anteriorly):** ? → 1. Langston (1953:fig. 13) and Schoch (1997:fig. 3) figured wide separation.
- **260 (dentary):** ? → 0. As with character 147.
- **261 (dentary, coronoid process):** ? → 0. As with character 147.
- **262 (surangular, normal):** ? → 0. As with character 147.
- **264 (number of splenials):** ? → 0. As with character 147.
- **266 (Meckelian fossae):** ? → 0. Langston (1953:387) identified two Meckelian foramina, one within the first third of the length of the postsplenial, the posterior Meckelian foramen bordered by the angular and the prearticular, and another “smaller opening” adjacent to the postsplenial-angular suture.
- **267 (ventral border of Meckel’s fossa):** ? → 1. As with character 266.
- **269 (articulation to tooth row):** ? → 0. As with character 147.
- **271 (number of coronoids):** ? → 0. As with character 147.
- **272 (coronoid teeth):** ? → 1. As with character 147.
- **275 (jaw sculpture):** ? → 0. As with character 147.
- **311 (parietals, anterior waisting):** 0 → 0&1. As shown to be variable by Schoch (1997:fig. 1).
- **312 (parietal width):** ? → 1. As shown by Langston (1953:fig. 13) and Schoch (1997:figs. 1–2).
- **314 (prefrontals):** ? → 0. As with character 312.
- **315 (postfrontals):** ? → 0. As with character 312.
- **317 (zygokrotaphy):** ? → 0. As with character 312.
- **345 (dorsolateral osteoderms fused to ribs):** 0 → ?. As with character 202.
- **347 (interclavicle):** 0 → ?. As with character 202.
- **348 (cleithrum):** 0 → ?. As with character 202.
- ***Micromelerpeton credneri***
- **39 (prefrontal-frontal):** 0 → 0&1. This is variable within a single individual (Boy, 1995:fig. 2B; Schoch & Milner, 2014:fig. 25D).
- **121 (pterygoid, flange):** 1 → 0&1. This is polymorphic (Boy, 1995:fig. 3).
- **131 (ectopterygoid length):** 0 → 0&1. This is polymorphic (Boy, 1995:fig. 3).
- **154 (ceratobranchials):** 0 → ?. This character prescribes the adult condition, and Boy (1995) and Witzmann (2013) noted that the hyobranchium is unknown in adults.
- **155 (basibranchial):** 1 → ?. As with character 154.
- **156 (hypobranchial):** 1 → ?. As with character 154.
- **250 (teeth on pterygoid):** ? → 0. Boy (1995) did not make any mention of teeth on the pterygoid (or any other part of the palate), nor did he reconstruct it or indicate it in figures showing the ventral surface of the pterygoid.
- **251 (denticles on vomers):** ? → 0. Boy (1995:435, fig. 3) stated that all palatal surfaces are covered in denticles.
- **252 (denticles on palatines):** ? → 0. As with character 251.
- **258 (pterygoids contact anteriorly):** ? → 1. Boy (1995:fig. 3) figured wide separation.
- **266 (Meckelian fossae):** ? → 0/1. Boy (1995:fig. 4) identified at least one fossa entirely within the postsplenial and indicates the posterior fossa is either absent or reduced to a small foramen.
- **271 (number of coronoids):** ? → 0/1. Boy (1995:fig. 4) identified as least two coronoids; there is a possible greatly truncated third coronoid (anterior coronoid) near the symphysis.
- **272 (coronoid teeth):** ? → 1. Boy (1995:438, fig. 4) indicated the coronoids are only covered in denticles.
- **311 (parietal, waisting):** ? 🡪 0&1. As shown by Boy (1995) among others.
- **312 (parietal, width):** ? 🡪 0. As shown by Boy (1995) among others.
- **314 (prefrontals):** ? → 0. As shown by Boy (1995) among others.
- **315 (postfrontals):** ? → 0. As with character 314.
- **317 (zygokrotaphy):** ? → 0. As with character 311.
- ***Apateon pedestris***
- **39 (prefrontal, frontal):** 1 → 0. This taxon has been repeatedly illustrated with a prefrontal well anterior to the frontal (e.g., Schoch & Milner, 2008:fig. 3D), which separates it from other species that have in-line anterior margins (e.g., *A. caudacus*).
- **121 (pterygoid, flange):** 1 🡪 0. There is no posterolateral process from the palatine or quadrate ramus of the pterygoid (e.g., Schoch & Milner, 2014:fig. 27G).
- ***Micropholis stowi***
- **31 (frontal-nasal, length):** 0 → 0&1. This variation is associated with the two morphs (broad- and slender-headed) that are noted by Schoch & Rubidge (2005:506).
- **62 (squamosal-tabular, dorsal):** 1 → 0&1. This variation is noted to occur on at least the dorsal surface of the roof (which is what the character refers to) by Schoch & Rubidge (2005:507).
- **65 (tabular, horn):** 0 → 0&1. Schoch & Rubidge (2005:507) noted the absence of a tabular horn in at least one large specimen.
- **86 (additional vomerine fangs):** 1 → 0&1. Schoch & Rubidge (2005:507) noted this as a variable feature between morphs; 86–0 (only one fang pair) occurs in the slender-headed morph, while 86–1 (three pairs) occurs in the broad-headed morph (see also their comparative reconstructions in figure 2 therein).
- **88 (ectopterygoid, fangs):** 1 → 0. Schoch & Rubidge (2005:507) stated that “the ectopterygoid has one fang-pair that is smaller than the palatine one,” which is reflected in their reconstruction (fig. 2 therein); this might be polymorphic (it is not reconstructed for the broad-headed morph), but they note that the ectopterygoid is not well-known from this morph.
- **109 (basicranium, carotids):** 0 → ?. Schoch & Rubidge (2005:508) confirmed the absence of foramina for the carotids on the ventral surface of the parasphenoid that was reported by Boy (1985). They speculated that the carotids might enter more posteriorly or simply never passed through the parasphenoid; this ambiguity does not support coding the character as 109–0.
- **110 (parasphenoid plate):** 2 → 1&2. This is a polymorphic condition differing between morphs (Schoch & Rubidge, 2005:fig. 2). The slender-headed morph has a proportionately narrower basal plate.
- **124 (pterygoid-palatine-ectopterygoid):** 1 → 0. Schoch & Rubidge (2005:508, fig. 2) noted substantial contact of the pterygoid with the palatine in the slender-headed morph. They indicated that the contact in the broad-headed morph was likely “faint,” as in their reconstruction, which is why this is corrected to 124­–0 rather than as polymorphic.
- **148 (anterior, middle coronoid teeth):** 1 → ?. Schoch & Rubidge (2005:509) stated that “the possession of anterior and middle coronoids and the morphology of the symphysis are not determinable because the area is obscured by the preorbital region.” Therefore, this feature should be coded as unknown.
- **149 (mandibular osteoderms):** 0 → 1. Schoch & Rubidge (2005:509–510) noted the presence of “oval, polygonal, or irregularly shaped ossicles emplaced between the basal plate of the parasphenoid and the ventral elements of the dermal pectoral girdle.” They also indicate that these are “true osteoderms and not dislocated tooth plates” and term them as “osteoderms of the throat region.”
- **205 (femur, trochanter):** 1 → 0. Schoch & Rubidge (2005:513) noted “a prominent, boss-like internal trochanter” on the femur, although this is not clear from their single-profile illustration. They compared it favorably to *Eoscopus*, which has a trochanter like other non-branchiosaurid amphibamiforms (e.g., *Doleserpeton*) based on Daly’s (1994:fig. 15) illustrations and description.
- **224 (frontal-orbit):** 0 → 1. Entry of the frontal was documented by Boy (1985:fig. 2) and Schoch & Rubidge (2005:figs. 1–3).
- **242 (number of premaxillary teeth):** ? → 0/1. Schoch & Rubidge (2005:509) stated that there is room for 8–10 premaxillary teeth.
- **243 (number of maxillary teeth):** ? → 0. Schoch & Rubidge (2005:509) stated that there is room for 31–42 maxillary teeth (variation between morphs).
- ***Platyrhinops lyelli***
- **100 (choana, medial):** 1 → 0. The choanae form elongate ovals without any broadening anteromedially (Clack & Milner, 2010:figs. 5, 9).
- **110 (parasphenoid plate):** 2 → 1. The condition of this taxon (Clack & Milner, 2010) is much closer to taxa coded for 110­–1, such as olsoniforms and zatracheids, than to amphibamids and salientians (coded for 110­–2).
- **203 (pubis):** ? → 1. Carroll (1964:238) noted a well-ossified pubis.
- **250 (teeth on pterygoid):** ? → 0. Clack & Milner (2010) did not make any mention of teeth on the pterygoid (or any other part of the palate), nor do they reconstruct it or indicate it in figures showing the ventral surface of the pterygoid.
- **251 (denticles on vomers):** ? → 0. Clack & Milner (2010:288) stated all palatal surfaces are covered in denticles.
- **252 (denticles on palatines):** ? → 0. As with character 251.
- **264 (splenials):** 2 🡪 0. Clack & Milner (2010:286) described both the splenial and the postsplenial, as with other temnospondyls.
- **272 (coronoid teeth):** ? → 1. Clack & Milner (2010:285) noted the coronoids are covered with denticles.
- ***Doleserpeton annectens***
- **148 (anterior, middle coronoid teeth):** 0 → ?. As noted by Sigurdsen & Bolt (2010:1366), “the presence of an anterior coronoid is uncertain.”
- **271 (number of coronoids):** 0 → 0/1. As with character 148; at least two coronoids have been identified.
- **346 (ectopterygoid):** 1 → ?. As noted by Schoch (2019a:148), whether an ectopterygoid is lost or merely very reduced in *D. annectens* is uncertain; the small size of specimens and any possible ectopterygoid greatly complicates this. The most recent study of *D. annectens* (Sigurdsen & Bolt, 2010:1365) state that “the ectopterygoid is either absent or greatly reduced.” To be conservative, this code is changed to unknown. Associated coding changes (88–133):
  - - **88 (ectopterygoid, fangs):** 1 → ?
    - **124 (pterygoid-palatine-ectopterygoid):** 1 → ?
    - **125 (palatine, ectopterygoid):** 0 → ?
    - **128 (palatine, ectopterygoid (ontogeny)):** 0 → ?
    - **129 (palatine, ectopterygoid (width)):** 1 → ?
    - **130 (palatine, ectopterygoid (continuous tooth row)):** 0 → ?
    - **131 (ectopterygoid (length)):** 1 → ?
    - **133 (ectopterygoid (Y-shaped)):** 0 → ?
- **143 (hamate process):** 0 → ?. The lower jaws of this taxon are very poorly known because of the frequent disarticulation of material, which is further complicated by the presence of several amphibamiforms at the type locality. Previous workers have barely described or figured any part of the lower jaw (e.g., Bolt, 1969:889; Bolt, 1977; Sigurdsen & Bolt, 2010:1366, fig. 3C). The character is uncoded here.
- **156 (hypobranchials):** 0 → 1. Witzmann’s (2013:153) review of the hyobranchial apparatus cited Sigurdsen & Bolt (2010) for evidence of ossified hypobranchials.
- **252 (denticles on palatine):** 0 → 1. This taxon has neither a pair of fangs nor any denticles; Sigurdsen & Bolt (2010:1365) stated that “a shagreen of denticles covers the vomers, the lateral shelf of the pterygoids, and a diamond-shaped field at the base of the cultriform process of the parasphenoid” without mention of the palatines.
- **266 (number of Meckelian fossae):** 0 → 0/1. Sigurdsen & Bolt (2010:1366) only described the posteriorly situated large opening; there is no reference to additional qualifying openings, but their figures are insufficient to determine whether smaller foramina might be present anteriorly within the postsplenial or splenial.
- ***Amphibamus grandiceps***
- **143 (hamate process):** 0 → ?. The lower jaws of this taxon are very poorly known because of the typical articulation with the skull and the overall compression of Mazon Creek specimens. Previous workers have barely described or figured any part of the lower jaw (e.g., Carroll, 1964:232–233, fig. 22; Bolt, 1979; Milner, 1982; Daly, 1994). The character is uncoded here.
- **317 (zygokrotaphy):** ? → 0. As with character 42 regarding documentation of the skull roof configuration.
- ***Gerobatrachus hottoni***
- **3 (ornament, elements):** 0 → ?. The only known specimen is only exposed ventrally (Anderson et al., 2008b:515).
- **4 (ornament, snout):** 0 → ?. As with character 3.
- **5 (ornament, general):** 0 → ?. As with character 3.
- **6 (ornament, intensive growth):** 0 → ?. As with character 3.
- **7 (ornament, preorbital):** 0 → ?. As with character 3.
- **24 (lacrimal, posterior extension):** 0 → ?. The posterior terminus of the lacrimal and the lateral margins of the orbit are not preserved (Anderson et al., 2008b:fig. 2).
- **27 (orbit margins):** 1 → ?. As with character 3.
- **39 (prefrontal-frontal):** ? → 1. The prefrontal’s anterior margin is sufficiently defined to code this character (as with character 40, which was originally coded).
- **47 (postorbital, shape):** 1 → ?. The posterior end of the postorbital is unknown (Anderson et al., 2008b:fig. 2).
- **48 (postorbital, end):** 0 → ?. As with character 47.
- **55 (supratympanic flange):** 0 → ?. As with character 3.
- **58 (jugal, anterior extension):** 0 → ?. The lateral orbital margins are not defined (Anderson et al., 2008b:fig. 2).
- **73 (postparietal-exoccipital):** 1 → ?. The parabasisphenoid is barely preserved and only at the basicranial articulation, let alone posteriorly (Anderson et al., 2008b:fig. 2), and there was no mention of the exoccipitals.
- **74 (postfenestral window):** 0 → ?. The occiput is not sufficiently preserved, and what part is preserved is too compressed to identify these openings.
- **75 (quadrate and occipital condyles):** 1 → ?. As with character 73.
- **86 (additional vomerine fangs):** 0 → -. There are no vomerine fangs.
- **100 (choana, medial):** ? → 0. Both characters 99 and 101, related to the choana’s entire shape, were previously coded. It is somewhat questionable whether the entire choana can actually be discerned given the disarticulation in the one specimen, but if those characters can be coded (they are not confidently overturned here), then this character must be scorable as well. The vomer gives no indication of an anteromedial expansion.
- **108 (parasphenoid):** 0 → ?. As with character 73.
- **118 (pterygoid, exoccipital):** 0 → ?. As with character 73.
- **119 (pterygoid, basioccipital):** 0 → ?. As with character 73; no basioccipital is identified or described.
- **136 (occipital condyle):** 1 → ?. As with character 73.
- **137 (exoccipital condyles):** 0 → ?. As with character 73.
- **138 (basioccipital, length):** 1 → ?. As with characters 73 and 119.
- **142 (postglenoid area, dorsal):** 0 → ?. This refers to the dorsal surface, which is obscured due to articulation with the compressed skull.
- **216 (supratemporal):** 1 → 0. A supratemporal was figured.
- **230 (postorbital):** ? → 0. A postorbital is present.
- **243 (number of maxillary teeth):** 0 → ?. In contrast to the premaxillary tooth count, no maxillary count or estimate was given. The tooth row appears incomplete posteriorly (markedly so), and if it were interpreted as being nearly complete, it is clearly much shorter than the premaxilla and would thus contain fewer than 21 positions.
- **245 (occipital profile):** 0 → ?. The specimen is clearly compressed and not exposed in occipital view.
- **296 (ribs anterior to sacrum):** 1 → 0. As with lissamphibians, this taxon has much shorter ribs than in other temnospondyls (Anderson et al., 2008b:fig. 1).
- ***Cacops aspidephorus***

*Note that these changes are considered to be applicable regardless of whether only *C. aspidephorus* or a chimera of *C. aspidephorus* + *C. morrisi* is employed.

- **27 (orbit margins):** 1 → 0. All three species of *Cacops* have prominent orbital rims (Reisz et al., 2009:fig. 2; Fröbisch & Reisz, 2012:fig. 2; Anderson et al., 2020:5, fig. 2A).
- **264 (splenials):** 1 🡪 0. There is a splenial and postsplenial in this species (Anderson, 2005; Anderson et al., 2020).
- **272 (coronoid teeth):** ? → 1. Anderson (2005:15) noted denticles on the three coronoids.
- **278 (trunk intercentra):** ? → 0. As noted by Williston (1910:261).
- **279 (trunk neural arch to centrum):** ? → 0. As noted by Williston (1910:261–262, pl. 9), Dilkes & Brown (2007:401, figs. 4–5), and Dilkes (2009:1014, fig. 1).
- **281 (haemal arches):** ? → 0. As noted by Williston (1910:265, pl. 9).
- **282 (haemal arches):** ? → 0. As with character 281.
- **283 (haemal arches):** ? → 0. As with character 281.
- **286 (transverse process):** ? → 0. As noted by Williston (1910:262, pl. 9).
- **287 (atlas-axis intercentra):** ? → 0. Williston (1910:262–263) described intercentra of both positions.
- **288 (atlas anterior centrum):** ? → 1. As with character 287.
- **289 (atlas centrum):** ? → 1. As with character 287.
- **290 (atlas neural arch):** ? → 1/2. Whether the arch is co-ossified or merely tightly sutured with the centrum is unclear in light of the preservation of most material of this taxon.
- **292 (atlas, neural arch):** ? → 0. The arch forms two separate prongs that are separated medially by an anterior expansion of the axial neural arch (Williston, 1910:263, pl. 14).
- **293 (proatlantes):** ? → 1. The completeness with which the skeleton is known confers confidence in the absence of proatlantes.
- **294 (second cervical arch):** ? → 0. As noted by Williston (1910:263).
- **299 (supraglenoid foramen):** ? → 0. As noted by Williston (1910:268).
- **301 (scapulocoracoid ossification):** ? → 0. As described by Williston (1910:268–269).
- **302 (deltopectoral crest):** ? → 1/2. As figured by Williston (1910:pl. 11).
- **307 (femur):** ? → 0. As figured by Williston (1910:pl. 13).
- **343 (parasphenoid cultriform process):** ? → 0. As figured by Williston (1910:pl. 7).
- **344 (dentary marginal dentition):** ? → 0. As figured by Williston (1910:pl. 8).
- ***Broiliellus texensis***
- **311 (parietals, anterior waisting):** ? → 1. As figured by Williston (1914:fig. 1) and DeMar (1966:fig. 4).
- **312 (parietal width):** ? → 0. As with character 311.
- **313 (skull table):** ? → 0. As with character 311.
- **314 (prefrontals):** ? → 0. As with character 311.
- **315 (postfrontals):** ? → 0. As with character 311.
- **317 (zygokrotaphy):** ? → 0. As with character 311.
- ***Dissorophus multicinctus***
- **47 (postorbital shape):** 1 → 0. Figures by DeMar (1968:fig. 2A) and reconstructions by Schoch (2012:figs. 1C, 3C) show a triangular postorbital that divides the squamosal and supratemporal.
- **75 (quadrate and occipital condyles):** 2 → 1. Schoch (2012:fig. 3D) reconstructed this taxon for 75–2, but the most recent description of this taxon by DeMar (1968:fig. 2B) depicted a condition closer to 75–1, which is what Dilkes (2020:22) advocated for in a reanalysis of a dissorophid-focused matrix.
- ***Acheloma cumminsi***

*Note that these changes are considered to be applicable regardless of whether *A. cumminsi* (sensu Polley & Reisz, 2011) or ‘*A. dunni*’ (sensu Polley & Reisz, 2011) is employed.

- **37 (maxilla, anterior margin):** 0 → 1. Lateral swelling in this region is characteristic of this taxon due to its caniniform dentition (e.g., Williston, 1909:fig. 1; Dilkes & Reisz, 1987:figs. 2A, 3A; Polley & Reisz, 2011:fig. 1).
- **100 (choana, medial):** 0 → 1. The anteromedial expansion of the choana (e.g., Polley & Reisz, 2011:figs. 1, 7) is autapomorphic for this taxon among trematopids (Gee, 2020).
- **209 (osteoderms):** 1 → 0. Osteoderms are not found in this taxon (e.g., Olson, 1941; Dilkes & Reisz, 1987).
- **214 (skull to trunk):** ? → 0. Williston’s (1909:fig. 6) reconstruction is based on the holotype of ‘*Trematops milleri*’, which has an essentially complete presacral column and skull.
- **220 (lacrimal-orbit):** ? → 1. This contact is well-documented (Olson, 1941:figs. 2A, 6; Dilkes & Reisz, 1987:fig. 3; Polley & Reisz, 2011:figs. 1–5).
- **249 (denticles on pterygoid):** ? → 0. As reported by Polley & Reisz (2011:803).
- **251 (denticles on vomers):** ? → 0. Polley & Reisz (2011:801) stated that the vomer is covered in denticles.
- **252 (denticles on palatines):** ? → 0. Polley & Reisz (2011:fig. 1) did not comment on the presence or absence of denticles in the text but did figure a loose covering.
- **287 (atlas-axis intercentra):** ? → 0. Williston (1909:645–646) and Polley & Reisz (2011:805) described intercentra of both positions.
- **288 (atlas anterior centrum):** ? → 1. As with character 287.
- **289 (atlas centrum):** ? → 1. As with character 287.
- **290 (atlas neural arch):** ? → 1/2. Whether the arch is co-ossified or merely tightly sutured with the centrum is unclear.
- **292 (atlas, neural arch):** ? → 0. The arch forms two separate prongs (Polley & Reisz, 2011:fig. 12C).
- **293 (proatlantes):** ? → 1. The completeness with which the skeleton is known confers confidence in the absence of proatlantes.
- **305 (olecranon process):** ? → 1. This process was documented in the most mature specimens by Olson (1941:168).
- **309 (number of distal tarsals):** ? → 1. The number of tarsals was documented by Dilkes (2014).
- ***Phonerpeton pricei***
- **37 (maxilla, anterior margin):** 0 → 1. Lateral swelling in this region is characteristic of this taxon due to its caniniform dentition (Dilkes, 1990:224, fig. 1A-B).
- **179 (interclavicle, length):** 0 → ?. There is no documented interclavicle for this taxon (Dilkes, 1990), and other interclavicle characters (180–187) were not originally coded.
- **209 (osteoderms):** 1 → 0. Osteoderms are not found in this taxon (Dilkes, 1990).
- **220 (lacrimal-orbit):** ? → 1. This contact is well-documented (Dilkes, 1990:figs. 1, 3–4).
- **251 (denticles on vomers):** ? → 0. Dilkes (1990:231, fig. 1B) stated all palatal surfaces are covered in denticles.
- **252 (denticles on palatines):** ? → 0. As with character 251.
- **305 (olecranon process):** ? → 1. This process is documented by Dilkes (1990:237).
- ***Onchiodon labyrinthicus***
- **121 (pterygoid, flange):** 0 → 1. A prominent flange is found in large individuals (Boy, 1990:fig. 3B).
- **243 (maxillary tooth count):** 2 → 0. Boy (1990:296) stated that there is room for 30–34 teeth on the maxilla.
- **272 (coronoid teeth):** 0 → 1. Boy (1990:298, fig. 4) indicated that the posterior coronoid is only covered by denticles, and the other two coronoids appear to be smooth. Based on how this character was originally coded (272–1, teeth absent) in taxa with denticulate coronoids like the outgroups, *Balanerpeton*, and edopoids, it is interpreted that denticles are differentiated from teeth for this character.
- **273 (coronoid teeth):** 2 → -. Correspondent with character 272.
- ***Eryops megacephalus***
  - **49 (postorbital, lateral process):** 0 🡪 0&1. As depicted by Sawin (1941:fig. 1), there is a slender lateral process from the postorbital that extends past the lateral margin of the orbit on at least one side of the skull. This could also be coded strictly for state 1.
- **121 (pterygoid, flange):** 0 → 1. A prominent flange is found in large individuals (Sawin, 1941:pl. 2).
- **272 (coronoid teeth):** ? → 1. Sawin (1941:430–431) noted denticles on the coronoids.
- **287 (atlas-axis intercentra):** ? → 0. Moulton (1974:26–27; figs. 1, 12) described these ossifications.
- **288 (atlas anterior centrum):** ? → 1. As with character 287.
- **289 (atlas centrum):** ? → 1. As with character 287.
- **290 (atlas neural arch):** ? → 0. As with character 287.
- **292 (atlas neural arch):** ? → 0&2. Moulton (1974:26–27) noted that the arches may be separate or coosified.
- **293 (proatlantes):** ? → 0. Moulton (1974:27) noted the presence of the proatlas.
- **305 (olecranon process):** ? → 1. A well-developed process was documented by Miner (1925:figs. 7, 21) and Pawley & Warren (2006:572–573, fig. 7.7–7.10).
- ***Sclerocephalus haeuseri***
- **33 (lateral line sulci, adults):** 0 → 0&1. Schoch & Witzmann (2009a:146) noted specimens from L–O 6 that have lateral line grooves but also mention a specimen from L–O 6 that was first noted by Boy (1988) to lack grooves.
- **121 (pterygoid, flange):** 0 → 1. A prominent flange comparable to the transverse flange reported in *Archegosaurus* is found in this taxon (Schoch & Witzmann, 2009a:fig. 4B).
- **156 (hypobranchials):** 0 → 1. Witzmann (2013:155, fig. 5F) identified a pair of elements as hypobranchials.
- **159 (caudal count):** 0 → 1. Schoch & Witzmann (2009a:153) only provided a caudal count for one specimen, but it is from L–O 6 and has 27 caudal positions, compared to 24–25 presacral positions.
- **203 (pubis):** 0 → 0&1. The code was originally adjusted as part of the recoding of this character (see character-specific comment) but was subsequently changed to a polymorphism because Schoch & Witzmann (2009a:157) stated that “the pubis is only present in some of the largest adults of *S. haeuseri* from L–O 6 […], whereas it is definitely absent in others (SMNS 90055).”
- **250 (teeth on pterygoid):** 1 → 0. Schoch & Witzmann (2009a:148) made no mention of teeth on the pterygoid, nor do they reconstruct or illustrate any, in contrast to teeth on the palatine and ectopterygoid.
- **251 (denticles on vomers):** 1 → 0. Schoch & Witzmann (2009a:148) stated that all palatal bones (exclusive of the parasphenoid) are covered in denticles.
- **252 (denticles on palatines):** 1 → 0. As with character 251.
- **272 (coronoid teeth):** 0 → 1. Schoch & Witzmann (2009a:150, fig. 4E) indicated the coronoids are only covered by denticles. Based on how this character was originally coded (272–1, teeth absent) in taxa with denticulate coronoids like the outgroups, *Balanerpeton*, and edopoids, it is interpreted that denticles are differentiated from teeth for this character.
- **273 (coronoid teeth):** 2 → -. Correspondent with character 272.
- **281 (haemal arches):** ? → 0. Schoch & Witzmann (2009a:153, fig. 7E) noted these features.
- **282 (haemal arches):** ? → 0. As with character 284.
- **283 (haemal arches):** ? → 0. As with character 284.
- **284 (haemal arch shape):** ? → 0. As with character 284.
- **287 (atlas-axis intercentra):** ? → 0. Schoch & Witzmann (2009a:151, fig. 7) described and figured most of this complex.
- **288 (atlas anterior centrum):** ? → 1. As with character 287.
- **290 (atlas neural arch):** ? → 0. As with character 287.
- **292 (atlas neural arch):** ? → 0/1. As with character 287; Schoch & Witzmann stated that they were “at best loosely attached.”
- **294 (second cervical arch):** ? → 0. As figured by Schoch & Witzmann (2009a:fig. 7A-B).
- **295 (cervical rib distal shape):** ? → 0. As figured by Schoch & Witzmann (2009a:fig. 7C).
- **296 (ribs anterior to sacrum):** ? → 0. As with character 295.
- **298 (number of caudal rib pairs):** ? → 0. Schoch & Witzmann (2009a:153) noted the presence of six pairs.
- **299 (supraglenoid foramen):** ? → 0. As described and figured by Boy (1988:122, fig. 8).
- **300 (number coracoid foramina):** ? → 2. As with character 300.
- **301 (scapulocoracoid ossification):** ? → 0. As with character 300.
- **302 (deltopectoral crest):** ? → 2. Schoch & Witzmann (2009a:154, fig. 8A-C) depicted a well-developed thickened crest, which is better developed than in *Glanochthon* (previously coded for 302–1).
- **306 (basale commune):** ? → 0. As determined by figures of the pes (Schoch & Witzmann, 2009a:fig. 8E).
- **307 (femur):** ? → 1. As described by Schoch & Witzmann (2009a:157).
- **308 (elongate tibiale and fibulare):** ? → 0. As with character 306.
- ***Glanochthon latirostre***
- **37 (maxilla, anterior margin):** 0 → 1. Schoch & Witzmann (2009b:12) listed “locally differentiated teeth in the snout, with larger teeth producing lateral excursions of margin (*Intasuchus*, *Sclerocephalus*)” as a feature shared between *Glanochthon* and the aforementioned stereospondylomorphs.
- **121 (pterygoid, flange):** 0 → 1. A prominent flange comparable to the transverse flange reported in *Archegosaurus* is found in this taxon (Boy, 1993:fig. 3; Schoch & Witzmann, 2009b:fig. 4D).
- ***Archegosaurus decheni***
- **15 (internarial distance):** 1 → 0. This condition is ontogenetically variable (Witzmann, 2005:134), with state 15­–1 found in juveniles (e.g., Witzmann, 2005:fig. 2a), but 15–0 occurs in adults (Witzmann, 2005:fig. 5a).
- **39 (prefrontal, frontal):** 0 🡪 0&2. This is variable within a single individual (e.g., Witzmann, 2005:fig. 15).
- **85 (transverse tooth row, transvomerine):** 1 → 0. Witzmann (2005:145) only noted the presence of a tooth row along the medial edge of the choana, which may be continuous with, but is not equivalent to, the transvomerine row. Witzmann (2005)’s ‘type B’ palate (fig. 17B therein) does have a trio of teeth near the midline, but this is not a row, and the slight anteromedial angling of the parachoanal teeth also does not form a row; even if either could be considered a ‘row’, this character would have to be coded as polymorphic.
- **88 (ectopterygoid fangs):** 0 → 1. Witzmann (2005:146) stated that no fangs are present on the ectopterygoids.
- **121 (pterygoid, flange):** 0 → 1. A prominent flange is found in large individuals (Witzmann, 2005:146, fig. 17).
- **272 (coronoid teeth):** 0 → 1. Witzmann (2005:149, fig. 21) indicated the coronoids are only covered by denticles. Based on how this character was originally coded (272–1, teeth absent) in taxa with denticulate coronoids such as the outgroups, *Balanerpeton*, and edopoids, it is interpreted that denticles are differentiated from teeth for this character.
- **273 (coronoid teeth):** 2 → -. Correspondent with character 272.
- ***Uranocentrodon senekalensis***
- **107 (basicranium, suture):** 0 → 1. The extent of the suture is certainly more than 40% of the length of the basal plate and does not differ substantially from other stereospondyls (e.g., Broom, 1930:fig. 2).
- **219 (lacrimal-naris):** 0 → 1. The lacrimal is widely separated from the naris (e.g., Marsicano et al., 2017:fig. 4).
- **249 (denticles on pterygoid):** ? → 0. Broom (1930:fig. 2) depicted denticles on the pterygoid. This agrees with both earlier work (e.g., Haughton, 1925:228) and the phylogenetic coding by Marsicano et al. (2017), the most recent revision of Rhinesuchidae.
- **251 (denticles on vomer):** ? → 0. As with character 249.
- **311 (parietals, anterior waisting):** ? → 1. As most recently documented by Marsicano et al. (2017:fig. 4).
- **312 (parietal width):** ? → 1. As with character 311.
- **313 (skull table):** ? → 0. As with character 311.
- **314 (prefrontals):** ? → 0. As with character 311.
- **315 (postfrontals):** ? → 0. As with character 311.
- **317 (zygokrotaphy):** ? → 0. As with character 311.
- ***Lydekkerina huxleyi***
- **38 (maxilla-nasal):** 1 → 0&1. This condition is intraspecifically variable per Jeannot et al. (2006:fig. 5).
- **75 (quadrate and occipital condyles):** 1 → 0. Illustrations and reconstructions (Shishkin et al., 1996:fig. 7; Schoch & Milner, 2000:fig. 57; Jeannot et al., 2006:figs. 2, 7; Hewison 2007:figs. 30, 32) depict occipital condyles anterior to the quadrate condyles.
- **88 (ectopterygoid, fangs):** 1 → 0&1. Jeannot et al. (2006), citing Shishkin et al. (1996:1646), stated this character is polymorphic.
- **107 (basicranium, suture):** 0 → 1. The extent of the suture is certainly more than 40% of the length of the basal plate and does not differ substantially from other stereospondyls (Shishkin et al., 1996:fig. 7; Jeannot et al., 2006:figs. 2, 7; Hewison 2007:fig. 32).
- **124 (pterygoid-palatine-ectopterygoid):** 0 → 0&1. Jeannot et al. (2006:829) noted that the ectopterygoid is excluded from the interpterygoid vacuity by a point contact between the pterygoid and the palatine (as with Shishkin et al., 1996:fig. 7), whereas Hewison (2007:22) noted the absence of this suture in other specimens.
- **145 (Meckelian window):** 1 → 0. The size of this opening is proportionately in line with the plesiomorphic condition observed in most non-stereospondyls (e.g., Jeannot et al., 2006:fig. 4; Hewison, 2007:fig. 34)
- **148 (anterior, middle coronoid teeth):** 1 → 0. While this taxon lacks “teeth” in the sense of similarly sized features to the marginal teeth, a covering of denticles on all three is the plesiomorphic condition, and many non-stereospondyls with denticle coverings but no coronoid teeth were coded for state 0. Jeannot et al. (2006:831) confirmed the presence of denticles on all three coronoids.
- **155 (basibranchials):** ? → 1. Witzmann (2013:155) noted the presence of basibranchials.
- **196 (carpals):** ? → 1. Pawley & Warren (2005:289, fig. 5) described and figured carpals.
- **219 (lacrimal-naris contact):** 1 → 0&1. Jeannot et al. (2006:828, fig. 5) cited this as an example of non-taphonomic intraspecific variation.
- **225 (septomaxilla):** ? → 0. Septomaxillae are often absent in specimens of this taxon (Jeannot et al., 2006:826, 828, fig. 5), but an ossified septomaxillae has been documented by numerous workers (e.g., Jeannot et al., 2006; Hewison, 2007:9–10), and it is felt that the balance favors post-mortem loss or immaturity in specimens without this bone (as with Jeannot et al., 2006:fig. 5).
- **243 (maxillary tooth count):** 1 → 0. Jeannot et al. (2006:830) were able to identify at least 29 teeth on the left maxilla of the holotype and 38 teeth on the right. Hewison (2007:12) only identified 31 teeth on the right maxilla but reconstructed several additional positions.
- **287 (atlas-axis centra):** ? → 0. Jeannot et al. (2006:832) reported the presence of intercentra associated with these two positions.
- **293 (proatlantes):** ? → 1. Pawley & Warren (2005:282), citing Broili and Schröder (1937), considered the absence of a proatlas among the abundance of material to be genuine.
- **312 (parietal, width):** 1 🡪 0. Jeannot et al. (2006) and Hewison (2007) depict parietals that are wider than the frontals.
- ***Siderops kehli***
- **10 (premaxilla, alary process):** 1 → ?. The reconstruction of the premaxillary-nasal suture is entirely conjectural (Warren & Hutchinson, 1983:9–10, fig. 2).
- **11 (premaxilla, prenarial portion):** 0 → ?. As with character 10.
- **12 (premaxilla, outline):** 0 → ?. As with character 10.
- **13 (premaxillary foramen):** 0 → ?. As with character 10. Though Warren & Hutchinson (1983:10) stated that none was found, because the opening can be variably sized, and the tip of the premaxilla is not preserved on the skull roof, this is best left as unknown.
- **20 (nasal, lateral margin):** 0 → ?. The lateral suture is too incomplete on both sides to assess its shape (Warren & Hutchinson, 1983:fig. 2).
- **31 (frontal-nasal, length):** 0 → ?. The reconstruction of the frontal-nasal suture is entirely conjectural (Warren & Hutchinson, 1983:fig. 2).
- **32 (frontal-nasal, suture**): 0 → ?. As with character 31.
- **35 (infraorbital sulcus):** 0 → ?. The sensory groove canals are both asymmetrical and interrupted (Warren & Hutchinson, 1983:9–10, figs. 1–2); it is not clear that this groove is sufficiently posteriorly extensive to capture any flexure like that found in some other taxa. If it is considered to be sufficient, then it would be state 1 (S-shaped flexure) based on a prominent curve from the left maxilla to the left prefrontal; the right side does not preserve a groove in the same region.
- **38 (maxilla-nasal):** 1 → ?. Despite the absence of a lacrimal, this does not ensure maxilla-nasal contact, and this suture nor the anterior terminus of the prefrontal are confidently known (Warren & Hutchinson, 1983:fig. 2).
- **41 (prefrontal, lateral suture):** 0 → ?. Only a small portion of the anterolateral margin is preserved, and the region around the orbit (referenced in state 1) is unknown (Warren & Hutchinson, 1983:fig. 2).
- **44 (prefrontal-jugal):** 1 → ?. the anterior extent of the jugal is unknown, and the anterolateral and lateral orbital margins are not well-preserved (Warren & Hutchinson, 1983:fig. 2).
- **47 (postorbital, shape):** 0 → ?. Only the anterolateral and medial sutures are known (Warren & Hutchinson, 1983:fig. 2), which is insufficient to determine whether it is “wedged deeply between squamosal and supratemporal.”
- **48 (postorbital, end):** 0 → ?. As with character 47.
- **50 (postorbital, postfrontal):** 1 → ?. None of the four elements specified for relative length here (postorbital, postfrontal, supratemporal, parietal) are completely defined at both anterior and posterior ends (Warren & Hutchinson, 1983:fig. 2).
- **51 (otic notch):** 1 → 0. Warren & Hutchinson (1983:7, 9, fig. 2) expressly reconstructed an otic notch (so coding off of the reconstruction, as would have to be done for other previously coded characters, would produce this coding change) and described features of the occiput that indicate support for a notch (when ignoring the reconstruction in coding).
- **53 (supratemporal):** 0 → ?. As with character 50.
- **56 (semilunar flange):** 0 → ?. It is not possible to discern whether the supratemporal reached the otic notch (Warren & Hutchinson, 1983:fig. 2), so it cannot be determined that it had no ventral projection.
- **58 (jugal, anterior extension):** 0 → ?. The anterior extent of the jugal is unknown (Warren & Hutchinson, 1983:fig. 2).
- **61 (intertemporal and postorbital):** 0 → ?. Because character 60 (presence/absence of intertemporal) was originally uncoded, which we prefer to follow here, this character cannot be coded.
- **62 (squamosal-tabular, dorsal):** 1 → ?. This sutural relationship is entirely conjectural (Warren & Hutchinson, 1983:fig. 2).
- **71 (posterior skull rim):** 1 → ?. Although there is sufficient evidence for a tabular horn, its posterior extent is unknown, and thus this feature cannot be assessed (Warren & Hutchinson, 1983:fig. 2).
- **85 (transverse tooth row, transvomerine):** 0 → 1. Warren & Hutchinson (1983:15, fig. 9) described and figured these teeth.
- **127 (LEP):** 0 → ?. The predicted region of the LEP is not resolved suturally (Warren & Hutchinson, 1983:fig. 2).
- **146 (symphyseal teeth):** 0 → 1. Warren & Hutchinson (1983:25) stated that “posterior to the tusk there is a further short tooth row.”
- **168 (pleurocentra):** 0 🡪 1. Warren & Hutchinson (1983:31) stated that there were no ossified pleurocentra in association with the skeleton. Considering that practically the entire axial skeleton was recovered, the absence of pleurocentra suggests a biological absence (as in *Pelorocephalus*, as noted by Warren & Hutchinson); this may be more widespread in chigutisaurids or brachyopoids at large, but the paucity of postcranial remains for most taxa remains a hindrance.
- **170 (pleurocentrum, ventral extension):** 0 🡪 -. As with character 168; note that character 169 (pleurocentrum, lateral surface) was originally uncoded.
- **207 (gastral squamation):** 0 → 1. There are no scales reported anywhere on the skeleton (Warren & Hutchinson, 1983; Witzmann, 2007).
- **214 (skull to trunk):** ? → 1. The presacral column and skull are nearly complete in this taxon, sufficiently to estimate this ratio.
- **222 (quadratojugal):** ? → 0. A quadratojugal is confidently identified (Warren & Hutchinson, 1983:6, 11).
- **226 (prefrontal-naris**): 1 → ?. As with character 38.
- **231 (shape of postorbital):** 0 → ?. As with characters 47 and 48.
- **237 (squamosal-jugal):** 0 → ?. This sutural relationship is entirely conjectural (Warren & Hutchinson, 1983:fig. 2).
- **239 (tabular):** ? → 0. Warren & Hutchinson’s (1983:7, 9) justification is considered sufficient for the inference of a tabular based on the occipital anatomy; other characters related to the tabular that were added by Pardo et al. (2017a) were coded by those workers (e.g., character 234, parietal-tabular contact).
- **268 (retroarticular process):** 0 → ?. Warren & Hutchinson (1983:21) stated that the process is lost in both lower jaws.
- **350 (premaxilla):** 0 → ?. As with character 10.
- ***Batrachosuchus browni***

* Note that corrections for characters 140–159, 174–175, 177–178, 208, 260–263, 269–272, 322, 326, 344, 347, 348, and 360 (all mandibular or postcranial) are considered to be applicable regardless of whether the more restrictive concept of *Batrachosuchus browni* or *Bathignathus watsoni* is employed; neither taxon is known from more than a partial skull.

- **10 (premaxilla, alary process):** 1 → ?. Chernin (1977:102) stated that “no premaxillary or maxillary sutures are discernible and only the posterior half of the right narial border is preserved as an impression.” The reconstruction of the premaxilla is expressly based on other species of *Batrachosuchus* (p. 99–100 therein).
- **11 (premaxilla, prenarial portion):** 0 → ?. As with character 10.
- **12 (premaxilla, outline):** 0 → ?. As with character 10.
- **13 (premaxillary foramen):** 0 → ?. As with character 10.
- **19 (naris, flange):** 0 → ?. As with character 10 regarding the narial preservation.
- **35 (infraorbital sulcus):** 0 → ?. The only specimen lacks the anterolateral margins where this sulcus typically occurs, and there was no discussion of the groove in the text (Chernin, 1977).
- **57 (jugal, ventral exposure):** 1 → ?. There was no comment or figuring of a ventral exposure by Chernin (1977). Neither was this reconstructed or reported in *Bathignathus watsoni* (Watson, 1956:fig. 6; Schoch & Milner, 2014:fig. 47B).
- **58 (jugal, anterior extent):** 0 → ?. The anterolateral margins of the orbits are not preserved, there is no comment on the anterior extent of the jugal, and the maxillary sutures are not identifiable. Schoch & Milner (2014:fig. 46E) reconstructed this taxon (and *Bathignathus watsoni*) as having jugals extending anterior to the orbit (58–1), but we prefer to leave this uncoded to be conservative.
- **91 (anterior palatal opening(s)):** 1 → ?. As with character 10 regarding the absence of premaxillae. The preserved anteriormost edge of the vomers is straight and without indicate of a pair of fossae or foramina in this position. The foramen situated entirely within the vomers (Chernin, 1977:fig. 5b) is not homologous with the paired anterior perforations for symphyseal fangs that are found in other temnospondyls and based on the specific inclusion of language regarding the fangs in this character, the median vomerine foramen is best regarded as being irrelevant to this character. Chernin (1977:104) also suggested that this foramen might be the same as a poorly ossified region in *Hadrokkosaurus bradyi* (Welles & Estes, 1969:fig. 26b), which is well posterior to the vomer-premaxilla suture and the anterior palatal opening.
- **92 (anterior palatal opening(s)**: 0 → ?. As with character 91.
- **140 (postglenoid area):** 1 → ?. There is no lower jaw material for this taxon (Chernin, 1977:102; Warren & Marsicano, 2000:465; Schoch & Milner, 2014:98).
- **141 (postglenoid area (types):** 0 → ?. As with character 140.
- **142 (postglenoid area (dorsal):** 0 → ?. As with character 140.
- **143 (hamate process):** 0 → ?. As with character 140.
- **144 (preglenoid process):** 0 → ?. As with character 140.
- **145 (Meckelian window):** 0 → ?. As with character 140.
- **146 (symphyseal teeth):** 0 → ?. As with character 140.
- **147 (posterior coronoid teeth):** 0 → ?. As with character 140.
- **148 (anterior, middle coronoid teeth):** 0 → ?. As with character 140.
- **149 (mandibular osteoderms):** 0 → ?. Based on the definition of state 1 (between mandible and dermal pectoral girdle), this character cannot be assessed in the absence of both a mandible and the pectoral girdle.
- **174 (ribs, uncinate spines):** 0 → ?. No postcrania are known for this taxon (Chernin, 1977:102; Warren & Marsicano, 2000:465; Schoch & Milner, 2014:98), as reflected in the absence of codes for most other postcranial characters.
- **175 (cleithrum):** 0 → ?. As with character 174.
- **177 (cleithrum):** 0 → ?. As with character 174.
- **178 (clavicle, ventral blade):** 0 → ?. As with character 174.
- **208 (gastral scales):** 0 → ?. As with character 174.
- **209 (osteoderms):** 0 🡪 ?. As with character 174.
- **222 (quadratojugal):**? → 0. The element is not exposed dorsally but is exposed in occipital view (Chernin, 1977:fig. 5C).
- **260 (dentary):** 0 → ?. As with character 140.
- **261 (dentary, coronoid process):** 0 → ?. As with character 140.
- **262 (surangular):** 0 → ?. As with character 140.
- **263 (angular):** 0 → ?. As with character 140.
- **268 (retroarticular process):** 0 → ?. As with character 140.
- **269 (articulation to tooth row):** 1 → ?. As with character 140.
- **270 (angular):** 0 → ?. As with character 140.
- **271 (number of coronoids):** 2 → ?. As with character 140.
- **272 (coronoid teeth):** 1 → ?. As with character 140.
- **322 (splenial teeth):** 1 → ?. As with character 140.
- **326 (anterior dentary teeth):** 1 → ?. As with character 140.
- **344 (dentary marginal dentition):** 0 → ?. As with character 140.
- **347 (interclavicle):** 0 → ?. As with character 174.
- **348 (cleithrum):** 0 → ?. As with character 174.
- **350 (premaxilla):** 0 → ?. As with character 10.
- ***Plagiosuchus pustuliferus***
- **88 (ectopterygoid fangs):** 1 → 0. Damiani et al. (2009:359) identified two large tooth sockets at the anterior end of the ectopterygoid that they interpreted as fangs; these are substantially larger than either teeth posterior or marginal teeth.
- **147 (posterior coronoid teeth):** 0 → 1. There is no mention of any coronoid teeth, nor do any appear on figures or reconstructions by Damiani et al. (2009:figs. 6–7).
- **148 (anterior, middle coronoid teeth):** 1 → -. There is only one coronoid, which Damiani et al. (2009:351,364) interpreted as the posterior coronoid. Therefore, this character is inapplicable.
- **209 (osteoderms):** 1 → 2. The osteoderms cover the entire body (Hellrung, 2003; Witzmann & Soler-Gijón, 2010).
- **219 (lacrimal-naris):** 0 → 1. The lacrimal is well-separated from the naris (Damiani et al., 2009:fig. 7).
- ***Sangaia lavina***
- **10 (premaxilla, alary process):** 1 → ?. The premaxillae are not preserved in either the holotype of referred specimen (Dias-da-Silva et al., 2006:383, fig. 3), and the reconstruction of this element is entirely conjectural.
- **11 (premaxilla, prenarial portion):** 0 → ?. As with character 10.
- **12 (premaxilla, outline):** 0 → ?. As with character 10.
- **13 (premaxillary foramen):** 0 → ?. As with character 10.
- **58 (jugal, anterior extent):** 0 → 1. The jugal extends anterior to the orbit (Dias-da-Silva et al., 2006:384, figs. 3, 6).
- **65 (tabular horn):** 1 → 0. Dias-da-Silva et al. (2006:385) described a tabular horn, noting that it is partially formed by the squamosal. Because the tabular turn sharply posterolaterally as in a “free” tabular horn, we consider it applicable here (as with Dias-da-Silva & Marsicano, 2011).
- **89 (interpterygoid vacuities):** 1 → ?. Partial preservation of the parasphenoid and pterygoid (Dias-da-Silva et al., 2006:fig. 4) is insufficient to discern the shape of these openings.
- **91 (anterior palatal opening(s)):** 1 → ?. The vomer is not preserved in either specimen (Dias-da-Silva et al., 2006:385). Character 85 (transvomerine teeth) was originally left uncoded.
- **92 (anterior palatal opening(s)):** 0 → ?. As with character 85.
- **93 (vomer):** 1 → ?. As with character 85.
- **94 (vomer, paired anterior depressions):** 0 → ?. As with character 85.
- **95 (anterior palatal depression):** 0 → ?. As with character 85.
- **96 (vomerine ridges):** 0 → ?. As with character 85.
- **97 (vomerine pit and fontanelle):** 0 → ?. As with character 85.
- **98 (vomerine septum):** 0 → ?. As with character 85.
- **99 (choana, lateral):** 0 → ?. The choana is not preserved.
- **100 (choana, medial):** 0 → ?. As with character 99.
- **101 (choana, width):** 0 → ?. As with character 99.
- **102 (vomer-pterygoid):** 1 → ?. As with character 85.
- **103 (vomer, anterior part):** 0 → ?. As with character 85.
- **104 (vomer, extension):** 0 → ?. As with character 85.
- **109 (basicranium (carotids)):** 1 → ?. There is no mention of the carotid foramina or any other foramina on the parasphenoid (Dias-da-Silva et al., 2006:385, fig. 4B).
- **112 (cultriform process, width):** 1 → ?. The cultriform process is not preserved (Dias-da-Silva et al., 2006:385, fig. 4B).
- **113 (cultriform process, structure):** 0 → ?. As with character 112.
- **114 (cultriform process, outline):** 0 → ?. As with character 112.
- **124 (pterygoid-palatine-ectopterygoid**): 0 → ?. Neither the ectopterygoid nor the palatine are exposed, if they are even preserved, so while Dias-da-Silva et al. (2006:385) conjectured that the pterygoid contacted the palatine, this is not documented in any specimen, and it is unclear whether the pterygoid is fully complete anteriorly.
- **129 (palatine, ectopterygoid (width)):** 0 → ?. As with character 124.
- **130 (palatine, ectopterygoid (continuous tooth row)):** 1 → ?. As with character 124.
- **131 (ectopterygoid (length):** 0 → ?. As with character 124.
- **133 (ectopterygoid (Y-shaped):** 0 → ?. As with character 124.
- **141 (postglenoid area (types):** 0 → ?. There is no lower jaw material, let alone a preserved PGA, as indicated by the lack of coding for character 140 (postglenoid area, presence/absence).
- **143 (hamate process):** 0 → ?. As with character 141.
- **144 (preglenoid process):** 0 → ?. As with character 141.
- **145 (Meckelian window):** 0 → ?. As with character 141.
- **149 (mandibular osteoderms):** 0 → ?. Based on the definition of 149–1 (between mandible and dermal pectoral girdle), this character cannot be assessed in the absence of both a mandible and the pectoral girdle.
  - **167 (parapophysis):** 0 → ?. No postcrania are known for this taxon (Dias-da-Silva et al., 2006), and other postcranial characters were largely uncoded.
  - **178 (clavicle, ventral blade):** 0 → ?. As with character 167.
  - **197 (manual digit count):** 1 → ?. As with character 167.
  - **202 (ilium, tip)**: 1 → ?. As with character 167.
  - **208 (squamation):** 0 → ?. As with character 167.
  - **209 (osteoderms)**: 0 → ?. As with character 167.
  - **212 (rib cage)**: 0 → ?. As with character 167.
  - **258 (pterygoids contact anteriorly):** 1 → ?. As with character 124.
  - **346 (ectopterygoid)**: 0 → ?. As with character 124.
  - **347 (interclavicle):** 0 → ?. As with character 167.
  - **348 (cleithrum):** 0 → ?. As with character 167.
  - **349 (vomer)**: 0 → ?. As with character 85.
  - **350 (premaxilla)**: 0 → ?. As with character 10.
  - **351 (choanal tooth row):** 0 → ?. As with character 99.
  - **355 (palatine, dentition)**: 0 → ?. As with character 124.
  - **356 (palatine)**: 0 → ?. As with character 124.
- ***Gerrothorax pulcherrimus***
- **58 (jugal, anterior extent):** 0 → 0&1. Schoch & Witzmann (2012:fig. 1) indicated that this condition is variable within a single specimen.
- **87 (parasphenoid, shagreen):** 0 → 0&1. Schoch & Witzmann (2012:377–378, 382, fig. 4) indicated that this condition is variable.
- **146 (symphyseal teeth):** 0 → 2. Schoch & Witzmann (2012:378) stated that “the symphyseal region lacks fangs but bears 2–5 tiny teeth posterior to the dental arcade, which recall the situation in some capitosaurs.” They indicated that this tooth row is a continuation of the coronoid dentition, and therefore it is considered to be parallel to the dentary row (state 2).
- ***Edingerella madagascariensis***
- **57 (jugal, ventral exposure):** 1 → 0. There is no figuring or description of this exposure (Lehman, 1961:fig. 9; Steyer, 2003:fig. 2B; Maganuco et al., 2009:fig. 10).
- **75 (quadrate and occipital condyles):** 1 → 0. The occipital condyles terminate well before the posterior face of the quadrate condyles (e.g., Maganuco et al., 2009:figs. 9–10).
- **87 (parasphenoid, shagreen):** 0 → 1. Steyer (2003) and Maganuco et al. (2009) both described the surface as “granular,” but the latter expressly noted the absence of denticles. The same feature appears work by in Lehman (1961:fig. 9), but all three of these studies referred to it as an “area aspera.”
- **125 (palatine, ectopterygoid):** 1 → 0. As shown by Maganuco et al. (2009:fig. 10), the palatine-pterygoid contact is mediated by an anteriorly extensive pterygoid overlapping onto the palatine (which has a transverse suture with the ectopterygoid), not by a posteromedial process of the palatine (as in most other capitosaurs).
- **145 (Meckelian window):** 1 → 0. The size of this opening is proportionately in line with the plesiomorphic condition observed in most non-stereospondyls (e.g., Lehman, 1961:fig. 11) in which state 0 is coded (e.g., *Eryops, Sclerocephalus*).
- **202 (ilium tip):** 1 → ?. No pelvic material is known for this taxon (Steyer, 2003:545; Maganuco et al., 2009:9), and the four other iliac characters were not coded originally.
- **237 (postparietal, length):** 1 → 0. The element is nearly a square and more anteriorly extensive than the tabular (Steyer, 2003:figs. 1–2, 6; Maganuco et al., 2009:fig. 6), as with most other stereospondyls.
- **251 (vomer, denticles):** 0 🡪 1. There are no denticles in this taxon per the common usage of ‘denticle’; Maganuco et al. (2009:18) describe a single row of denticles that represent the transvomerine tooth row found in other stereospondyls; one of the other authors of that study (Steyer) has used the term ‘denticle’ for any tooth smaller than the marginal dentition in an earlier study of this taxon (Steyer, 2003).
- ***Benthosuchus sushkini***
- **75 (quadrate and occipital condyles):** 1 → 0. The occipital condyles terminate before the posterior face of the quadrate condyles (Bystrow & Efremov, 1940:figs. 64, 66–67; Damiani, 2001:fig. 11; Schoch, 2008a:fig. 5B).
- **145 (Meckelian window):** 1 → 0. The size of this opening is proportionately in line with the plesiomorphic condition observed in most non-stereospondyls (e.g., Bystrow & Efremov, 1940:fig. 26; Schoch, 2000:fig. 7B; Damiani, 2001:fig. 12B) in which state 0 is coded (e.g., *Eryops, Sclerocephalus*).
- **148 (anterior, middle coronoid teeth):** 0 → 1. Figures by Bystrow & Efremov (1940:fig. 26), Schoch (2000:fig. 7B), Schoch & Milner (2000:fig. 71), and Damiani (2001:fig. 12) clearly show coronoid dentition is restricted to the posterior coronoid.
- **168 (pleurocentra):** 0 🡪 1. Bystrow & Efremov (1940) did not identify or describe any pleurocentra (contra Romer, 1947, and as with Howie, 1970). Given the large size range and large sample size, the lack of articulated skeletons is not considered prohibitive to recoding this taxon.
- **169 (pleurocentrum, lateral surface):** 0 🡪 -. As with character 168.
- **170 (pleurocentrum, ventral extension):** 0 🡪 -. As with character 168.
- **203 (pubis):** ? → 0. Bystrow & Efremov (1940:66) commented on the absence of an ossified pubis, which was reaffirmed by Romer (1947:209) and Warren & Snell (1991:table 1).
- ***Trematosaurus brauni***
- **31 (frontal, nasal):** 0 → 0&1. Schoch (2019b:53, fig. 6) noted variability in this feature.
- **58 (jugal, anterior extent):** 0 → 0&1. This condition is clearly variable (Schoch, 2019b:figs. 4–5).
- **137 (exoccipital condyles):** 0 → 0&1. Schoch (2019b:53, fig. 6) noted variability in this feature.
- **147 (posterior coronoid teeth):** 0 → ?. There is no mention of coronoid teeth (Schoch, 2019b), and the lingual surface is exceedingly poorly known in this taxon (“the coronoids are not preserved well enough in any specimen as to expose the sutures; Schoch, 2019b:53). Therefore, even if teeth were apparent on some of the inferred coronoid surface, it is unknown which coronoid they are on.
- **148 (anterior, middle coronoid teeth):** 0 → ?. As with character 147.
- **348 (cleithrum):** 0 → ?. Schoch (2019b:53–54) noted that definitively referable postcranial material is very rare because multiple temnospondyls occur in the type locality (Merkel’s Quarry), where postcrania are isolated. He mentioned only the interclavicle, clavicle, and one lost scapulocoracoid from another site with respect to this taxon.
- ***Trematolestes hagdorni***
- **44 (prefrontal-jugal):** 0 → 0&1. This condition is clearly variable within a referred specimen (Schoch, 2006:fig. 3A).
- **57 (jugal, ventral exposure):** 1 → 0. This exposure is not described or reconstructed by Schoch (2006:fig. 4B).
- **58 (jugal, anterior extent):** 0 → 0&1. This variability is related to character 44; a more anteriorly extensive jugal contacts the prefrontal.
- **88 (ectopterygoid fangs):** 1 → 0. Schoch (2006) made no definitive statements either way, but the reconstruction in that study (fig. 4B therein) depicted teeth at the anteriormost region of the ectopterygoid that are larger than the posteriormost teeth of the palatine. Based on other taxa with this condition that are coded for state 0 (e.g., *Glanochthon*, *Lyrocephaliscus*), the code is changed.
- **137 (exoccipitals):** 0 → 1. Figures and reconstruction based on the holotype (Schoch, 2006:figs. 1–2, 4) indicate well-exposed condyles in dorsal view.
- **237 (postparietal, length):** 1 → 0. The element is nearly square, more anteriorly extensive than the tabular (Schoch, 2006:figs. 2–4), and in stark contrast to the condition of zatracheids or highly nested amphibamiforms in which it is an anteroposteriorly short, tranvsersely elongate rectangle (e.g., Witzmann & Schoch, 2006:fig. 3; Clack & Milner, 2010:fig. 9a; Sigurdsen & Bolt, 2010:fig. 3A).
- **305 (olecranon process):** ? → 0. Schoch (2006:37) stated that this is unossified.
- ***Lyrocephaliscus euri***
- **85 (transverse tooth row, transvomerine):** 2 → 1. Save-Söderbergh (1935:fig. 5) and Schoch & Milner (2000:fig. 83) depicted only two transvomerine teeth. These cannot form a ‘V-shaped’ row.
- **161 (transverse process, length):** ? → 0. This feature can be coded based on Mazin & Janvier (1983:24–27, figs. 9–10).
- **162 (neural spine, height):** ? → 1. As with character 161.
- **163 (intercentrum, shape):** ? → 0. As with character 161.
- **164 (intercentrum, width):** ? → 1. As with character 161.
- **168 (pleurocentrum, presence):** ? → 0. As with character 161.
- **169 (pleurocentrum, lateral surface):** ? → 1. As with character 161.
- **170 (pleurocentrum, ventral extension):** ? → 0. As with character 161.
- **171 (ribs, length):** ? → 0. As with character 161.
- **172 (ribs, ventral extension):** ? → 1. As with character 161.
- **173 (ribs, uncinate blades):** ? → 0. As with character 161.
- **174 (ribs, uncinate spines):** ? → 0. As with character 161.
- **178 (clavicle, ventral blade):** ? → 0. This feature can be coded based on Mazin & Janvier (1983:27, fig. 11).
- **185 (interclavicle, anterior stylus):** ? → 1. This feature can be coded based on Mazin & Janvier (1983:27, fig. 12).
- ***Callistomordax kugleri***
- **24 (lacrimal, posterior extension):** 0 → 1. The lacrimal is not confined to the anterior orbital margin or the preorbital region and extends for a similar extent to *Batropetes*, originally coded for 24–1.
- **30 (interorbital distance):** 0 → 1. The interorbital distance is wider in specimens and reconstructions figured by Schoch (2008a:figs. 1–5).
- **66 (tabular, extension):** 0 → 1. Schoch (2008a:89, figs. 1–2) stated that “its [the tabular’s] blunt posterolateral end never extends posteriorly.”
- **75 (quadrate and occipital condyles):** 1 → 2. Figures and reconstruction by Schoch (2008a:91, figs. 1–2, 5) show the occipital condyles projecting beyond the quadrates, as in most metoposaurids, which is further substantiated by the text (“the condyles reach a level well posterior to the occipital rim of the dermal skull and behind the quadrate condyles”).
- **87 (parasphenoid, shagreen):** 0 → 1. Schoch (2008a:90) stated that the area aspera is heavily ornamented but edentulous.
- **88 (ectopterygoid, fangs):** 1 → 0. Schoch (2008a:90) stated that this element “bears a pair of small tusks, succeeded posteriorly by a row of 12 much smaller teeth.” This is reflected in the corresponding illustrations and reconstructions (figs. 1–2 therein).
- **146 (symphyseal teeth):** 0 → 1. Schoch (2008a:91) described at least one tooth posterior to the symphyseal tusk “similar in position to the postsymphyseal dentition of other temnospondyls,” and he coded the presence of symphyseal teeth in the matrix used in that study’s analysis (table A1 therein).
- **148 (anterior, middle coronoid teeth):** 0 → ?. This taxon is unusual in having dentition only on the middle coronoid; Schoch (2008a:92) stated that “the anterior edentulous coronoid is entirely smooth […].” We prefer to code this as unknown (inapplicable is another option) rather than as polymorphic here.
- **237 (postparietal, length):** 1 → 0. The element is nearly square, more anteriorly extensive than the tabular (Schoch, 2008a:figs. 1–5), and in stark contrast to the condition of zatracheids or highly nested amphibamiforms in which it is an anteroposteriorly short, tranvsersely elongate rectangle (e.g., Witzmann & Schoch, 2006:fig. 3; Clack & Milner, 2010:fig. 9a; Sigurdsen & Bolt, 2010:fig. 3A).
- **287 (atlas-axis intercentra):** ? → 0. Schoch (2008a:92) described this complex.
- **288 (atlas anterior centrum):** ? → 1. As with character 287.
- **289 (atlas centrum):** ? → 1. As with character 287.
- **293 (proatlantes):** ? → 1. The completeness of the holotype confers support for the absence of the proatlas.
- ***Metoposaurus krasiejowensis***

*Note that these changes are considered to be applicable regardless of whether *M. diagnosticus* or *M. krasiejowensis* (sensu Brusatte et al., 2015) is employed.

- **66 (tabular, extension):** 0 → 1. like in the metoposauroid *Callistomordax*, the tabular horn of *Metoposaurus* points far more laterally than it does posteriorly (e.g., Fraas, 1889:taf. XII; Sulej, 2002:figs. 3–4; Sulej, 2007:figs. 1B, 5, 13; Brusatte et al., 2015:figs. 2B, 11).
- **168 (pleurocentra):** 0 → 1. Pleurocentra are widely recognized to be absent in all metoposaurids except perhaps *Dutuitosaurus ouazzoui* (e.g., Dutuit, 1972, 1976; Hunt, 1993, but see Sulej, 2007).
- **237 (postparietal length):** 1 → 0. The postparietals are typically longer than they are wide (e.g., Fraas, 1889:145, taf. XII; Sulej, 2002:figs. 3–4; Sulej, 2007:figs. 1B, 4–14; Brusatte et al., 2015:figs. 2B, 3, 11).
- **242 (number of premaxillary teeth):** 1 → 0. Like other highly nested stereospondyls, metoposaurids have a relatively high tooth count, with more than 15 positions on the premaxilla (e.g., Sulej, 2007:34).
- ***Parotosuchus nasutus***
- **113 (cultriform process, structure):** 0 → 1. Schoch (2018:118) noted a median ventral ridge extending from the basal plate onto the cultriform process and extending for the posterior half of the process.
- **168 (pleurocentra):** 0 🡪 ?. Schoch (2018:119) stated that postcrania are difficult to confidently identify due to disarticulation at the type locality and that the vertebrae are entirely unknown.
- **170 (pleurocentrum, ventral extension):** 0 🡪 ?. As with character 168; note that character 169 (pleurocentrum, lateral surface) was originally uncoded.
- ***Paracylotosaurus davidi***
- **75 (quadrate and occipital condyles):** 1 → 0. Figures by Watson (1958:fig. 1) and Damiani (2001:fig. 21) show the occipital condyles situated slightly anterior to the quadrate condyles, contrary to Watson’s description that the condyles lie “essentially in the same transverse plane” (p. 244 therein).
- **91 (anterior palatal opening):** 1 → 0. There is no indication of any perforations for the symphyseal fangs (Watson:1958:fig. 1).
- **168 (pleurocentra):** 0 🡪 1. Watson (1958:247) stated that the pleurocentra were entirely cartilaginous. While their position can be ascertained from the articulated holotype, this does not allow them to be coded for any pleurocentra character given the unossified state.
- **169 (pleurocentrum, lateral surface):** 1 🡪 -. As with character 168.
- **170 (pleurocentrum, ventral extension):** 0 🡪 -. As with character 168.
- **197 (manual count):** 1 → 0. Watson (1958:251) noted five manual digits. Konietzko-Meier et al. (2020:1152–1153) also commented on this with respect to the present mounting of the holotype with four fingers as perhaps a modification from the original mounting with five fingers in light of the now disproven perception that all temnospondyls have four manual digits.
- **287 (atlas-axis intercentra):** ? → 0. Watson (1958:245–246, fig. 8) described and figured this complex.
- **288 (atlas anterior centrum):** ? → 1. As with character 287.
- **289 (atlas centrum):** ? → 0. As with character 287.
- ***Cyclotosaurus intermedius***

*Note that these changes are considered to be applicable regardless of whether *C. robustus* or *C. intermedius* is employed.

- **75 (quadrate and occipital condyles):** 1 → 2. The occipital condyles extend beyond the quadrates (Schoch, 2008a:fig. 5C).
- ***Mastodonsaurus giganteus***
- **15 (internarial distance):** 0 → 1. The narrow interorbital distance of this taxon is well-documented (Schoch, 1999:25, fig. 1)
- **75 (quadrate and occipital condyles):** 1 → 2. Schoch (1999:fig. 13) showed the occipital condyles extending beyond the level of the quadrates. Schoch (1999)’s statement about the condyles being at the same level (p. 56 therein) may refer to the dorsoventral plane (as they are indeed at the same level in this axis).
- **141 (postglenoid area (types)):** 0 → ?. Schoch (1999:80) stated that the PGA of this taxon is highly variable and displays characteristics of both types within a single jaw (i.e. it cannot be discretized into this binary); for this reason, we code the character as unknown rather than as polymorphic.
- **196 (carpals):** 0 → 1. Schoch (1999:130, fig. 45) described and figured at least two proximal carpals, which are known from a partial articulated skeleton from Gaildorf.
- **287 (atlas-axis intercentra):** ? → 0. Schoch (1999:98–99, figs. 29–30) described and figured this complex.
- **288 (atlas anterior centrum):** ? → 1. As with character 287.
- **289 (atlas centrum):** ? → 1. As with character 287.
- **290 (atlas neural arch):** ? → 2. As with character 287.
- **291 (atlas parapophyses):** ? → 2. As with character 287.
- **292(atlas neural arch):** ? → 1. As with character 287.
- **293 (proatlantes):** ? → 1. Schoch (1999:95) states that the proatlas is absent.
- ***Chinlestegophis jenkinsi***
- **21 (lacrimal):** ? → 0. Regardless of whether the lacrimal indeed fused with the maxilla or was ‘lost’ through some other process, there is no dorsal expression of the lacrimal or identification of a distinct lacrimal through tomography. This is considered sufficient here to restore the original code of Pardo et al. (2017a), which was changed by Schoch et al. (2020). The same character is not restored for *Rileymillerus cosgriffi* because CT analysis has not been performed for this taxon and because of suspicions over identity of the ‘nasal’ in this taxon (see Supporting Information). Correspondent with this change, codes for characters 22–24 (related to the lacrimal) were updated to ‘-‘ from ‘?’.
- **39 (prefrontal, frontal):** 1 → 0. The prefrontal clearly extends well beyond the frontal (Pardo et al., 2017a:fig. 1I).
- **56 (semilunar flange):** 1 → 0. The supratemporal is entirely excluded from the otic notch, let alone with a ventral flange (Pardo et al., 2017a:fig. 1I-J).
- **58 (jugal, anterior extent):** ? → 0. The presence of an LEP clearly truncates the jugal anteriorly (Pardo et al., 2017a:fig. 1I-J).
- **127 (LEP):** ? → 1. This code is restored from that of Pardo et al. (2017a), following their original interpretation. This was changed to unknown by Schoch et al. (2020), who stated that it might be a lacrimal, as has previously suggested for similar exposures in some trematosaurs (Schoch, 2006, 2008), but those studies have not demonstrated that an element in the same position is either definitively a lacrimal or definitively not a LEP (e.g., via CT analyses to identify the nasolacrimal duct passage or to assess the articulation of the palate with the roof). Furthermore, the aforementioned studies did not address the possibility that other temnospondyl clades without lacrimals might have similar overplating, and if *Chinlestegophis jenkinsi* were to be left coded as unknown for this character, all other taxa without lacrimals should have been treated the same. We do not opt for this approach here and restore the original code on the basis that there was no unequivocal basis for overturning it.
- **237 (postparietal, length):** 1 → 0. The element is nearly square, more anteriorly extensive than the tabular (Pardo et al., 2017a:fig. 1I), and in stark contrast to the condition of zatracheids or highly nested amphibamiforms in which it is an anteroposteriorly short, transversely elongate rectangle (e.g., Witzmann & Schoch, 2006:fig. 3; Clack & Milner, 2010:fig. 9a; Sigurdsen & Bolt, 2010:fig. 3A).
- **346 (ectopterygoid):** 0 → ?. The supporting information of Pardo et al. (2017a) indicated that the ectopterygoids are not preserved.
- **348 (cleithrum):** 0 → ?. The supporting information of Pardo et al. (2017a) indicated that a limited amount of postcrania is preserved (e.g., only one limb element), and only the dermal components of the pectoral girdle are present.
- **349 (vomer):** 0 → 1. The vomer is very fragmentary in this taxon (Pardo et al., 2017a), and both its sutures and the shape of the choana are entirely conjectural (as reflected in the ‘?’ codes for characters 93–104 and 323 that relate to these features and that were coded by Pardo et al., 2017).
- **351 (continuous tooth row lateral to choana):** 0 → ?. As with character 349.
- **355 (palatine dentition):** 0 → ?. There is no description or figuring of palatine dentition (Pardo et al., 2017a).
- ***Rileymillerus cosgriffi***
- **57 (jugal, ventral process):** ? → 1. Bolt & Chatterjee (2000:676) mentioned a “jugal processus alaris” several times and code this feature as present in their matrix (appendix 1 therein).
- **75 (quadrate and occipital condyles):** 1 → 2. The occipital condyles clearly extend past the level of the quadrate condyles (Bolt & Chatterjee, 2000:figs. 2.1–2.2).
- **82 (transverse tooth row, transvomerine):** 0 → 2. Bolt & Chatterjee (2000:678) described two sets of vomerine teeth (exclusive of ‘fangs’): one that extends posteriorly from a position medial to the anterior fang position and that “continues near the intervomerine suture” and the other than borders the medial margin of the internal naris. The former is vaguely illustrated in their reconstruction (fig. 2.2 therein) and forms a sharp V-shaped profile, similar to the condition seen in some trematosaurs (including *Almasaurus habbazi*; Dutuit, 1976:figs. 83–84), with the same point of origination and the same orientation towards the midline suture.
- **107 (basicranium, suture):** 1 → ?. Bolt & Chatterjee (2000:676) stated that crushing of this region results in the inability to identify any sutures between the parasphenoid, pterygoid, and exoccipital. Because the exoccipitals can have variable exterior extent (truncating the parasphenoid-pterygoid suture) and may contact each other medially (truncating the basal plate), this feature cannot be reliably coded.
- **108 (parasphenoid):** 0 → ?. As with character 107.
- **109 (basicranium, carotids):** 1 → ?. As with character 107. There is no mention of any carotid foramina.
- **114 (cultriform process, outline):** 1 → 0. Based on how the character was originally coded by Schoch (2013), it refers to the condition of eryopoids where there is a prominent and abrupt lateral expansion anterior to the base of the cultriform process; it does not refer to a gradual broadening onto the base, as in this taxon.
- **118 (pterygoid, exoccipital):** 1 → ?. As with character 107.
- **120 (pterygoid, squamosal):** 0 → 1. Bolt & Chatterjee (2000:675-676) discussed whether the term ‘palatoquadrate fissure’ was applicable to this taxon, but since the character refers only to a ‘fissure,’ it is coded as state 1 since there is a wide separation between the pterygoid and the squamosal.
- **127 (LEP):** ? → 1. This code is restored from that of Pardo et al. (2017a), following the original interpretation of Bolt & Chatterjee (2000:674, fig. 2). This was reversed by Schoch et al. (2020), and it was previously argued that it is merely a short lacrimal (Schoch, 2008b:83–84) but compelling evidence to overturn the original interpretation (e.g., CT data) or to prove that exposures in similar positions in other taxa are lacrimals and not LEPs (e.g., identifying the nasolacrimal duct) have not been provided. The position is in line with where an LEP occurs in dissorophoids and dvinosaurs, which already represent two independent acquisitions of this feature. Furthermore, past authors have not addressed the possibility that other temnospondyl clades without lacrimals might have similar overplating, and if *Chinlestegophis jenkinsi* were to be left uncoded for this character, all other taxa without lacrimals (e.g., *Siderops, Laidleria*) should have been treated the same. We do not opt for this approach here and restore the original code on the basis that there was no unequivocal basis for overturning it.
- **145 (Meckelian window):** 0 → ?. Bolt & Chatterjee, 2000:677, fig. 4) noted that they were unable to locate any Meckelian opening and that only the posterior quarter of the lower jaw is exposed and well-preserved.
- **237 (postparietal, length):** 1 → ?. The element’s lateral margins are not discernible on the roof (Bolt & Chatterjee, 2000:675, figs. 2.1, 3), but it was clearly a relative anteroposteriorly long element, concurrent with the skull’s general proportions. Mapping the postparietal-tabular suture from the occiput onto the skull roof would indicate an element far longer than it is wide, but because it cannot be assumed that the postparietal’s lateral margin was longitudinally oriented, this character is recoded as unknown.
- **251 (vomer, denticles):** 0 🡪 1. Bolt & Chatterjee (2000:678) stated that no palatal denticles were identified.
- **350 (premaxilla):** 0 → ?. There is no premaxilla preserved in either the holotype or the referred specimen (Bolt & Chatterjee, 2000; Martz et al., 2013).
- **360 (parietal):** 0 → ?. The parietal is only defined along the midline and is insufficient to determine its shape (Bolt & Chatterjee, 2000:672, fig. 2.1)
- ***Triadobatrachus massinoti***
- **39 (prefrontal, frontal):** 1 → 0. The prefrontal extends beyond the frontal (e.g., Rage & Roček, 1989:figs. 2, 4; Ascarrunz et al., 2016:fig. 4). Alternatively, the character could be coded as inapplicable because the frontal and parietal are co-ossified in this taxon.
- ***Celtedens ibericus***
- **39 (prefrontal, frontal):** 1 → 0. The prefrontal clearly extends beyond the frontal (e.g., McGowan, 2002:fig. 5). Alternatively, the character could be coded as unknown or inapplicable because the element lateral to the nasal is sometimes interpreted as the lacrimal or as a co-ossified lacrimal and prefrontal (McGowan, 2002), and other authors have expressed general uncertainty over whether this region can be resolved (e.g., Matsumoto & Evans, 2018).
- ***Ichthyophis bannanicus***
- **114 (cultriform process, outline):** 1 → 0. Based on how the character was originally coded by Schoch (2013), it refers to the condition of eryopoids where there is a prominent and abrupt lateral expansion anterior to the base of the cultriform process; it does not refer to a gradual broadening onto the base, as in this taxon.
- **272 (number of coronoids):** 0 → 2. Based on the embryological data suggesting that there is only a single coronoid in gymnophionomorphs (that does not clearly represent fusion of the plesiomorphic three coronoids found in most temnospondyls), we recoded this character (e.g., Wake & Hanken, 1982; Müller et al., 2005; Müller, 2006; Theska et al., 2019). Alternatively, the character could be coded as unknown since this specific taxon has not been studied developmentally.
- ***Epicrionops bicolor***
- **114 (cultriform process, outline):** 1 → 0. Based on how the character was originally coded by Schoch (2013), 114–1 refers to the condition of eryopoids where there is a prominent and abrupt lateral expansion anterior to the base of the cultriform process (e.g., Sawin, 1941:pls. 2–3; Boy, 1990:fig. 3B); it does not refer to a gradual broadening onto the base, as in this taxon (e.g., Reiss, 1996:fig. 2D).
- **272 (number of coronoids):** 0 → 2. Based on the embryological data suggesting that there is only a single coronoid in gymnophionomorphs (that does not clearly represent fusion of the plesiomorphic three coronoids found in most temnospondyls), we recoded this character (e.g., Wake & Hanken, 1982; Müller et al., 2005; Müller, 2006; Theska et al., 2019). Alternatively, the character could be coded as unknown since this specific taxon has not been studied developmentally.
- ***Eocaecilia micropodia***
- **39 (prefrontal, frontal):** 1 → 0. The prefrontal clearly extends beyond the frontal.
- **266 (number of Meckelian fossae):** ? → 1. Jenkins et al. (2007:319, 322, figs. 24, 27) identified one opening that they termed the “intramandibular foramen” and which they suggested was occupied by a remnant of Meckel’s cartilage, as in extant gymnophionans (coded for 266–1). More anteriorly situated foramina all occur just below the lingual tooth row and likely relate to tooth replacement, not to Meckel’s cartilage.
- **272 (number of coronoids):** 0 → 2. Based on the embryological data suggesting that there is only a single coronoid in gymnophionomorphs (that does not clearly represent fusion of the plesiomorphic three coronoids found in most temnospondyls), we recoded this character (e.g., Wake & Hanken, 1982; Müller et al., 2005; Müller, 2006; Theska et al., 2019). Alternatively, the character could be coded as unknown since this specific taxon cannot be studied developmentally.

**5. Supplementary Tables**

**Supplementary Table 1: Adductor fossa to pseudoangular length ratios**

| **Taxon** | **Specimen number (source)** | **Ratio:** (anteroposterior length of adductor fossa in dorsal view) / (anteroposterior length of pseudoangular*) |
| --- | --- | --- |
| *Caecilia tentaculata* | MW 5138 (Wilkinson et al., 2011) | 0.13 |
| *Dermophis mexicanus* | NHMUK 64.1.26.397 (Wilkinson et al., 2011) | 0.13 |
| *Doleserpeton annectens* | FMNH UR1335 (Sigurdsen & Bolt, 2010) | 0.42 |
| *Eocaecilia micropodia* | MNA V8066 (Jenkins et al., 2007) | 0.17 |
| *Epicrionops petersi* | USNM 160360 (Nussbaum, 1977) | 0.13 |
| *Funcusvermis gilmorei* | PEFO 46480 (Wilkinson et al., 2011) | 0.32 |
| *Herpele squalostoma* | MW 4532 (Wilkinson et al., 2011) | 0.10 |
| *Ichthyophis glutinosus* | MW 1773 (Wilkinson et al., 2011) | 0.15 |
| *Indotyphlus battersbyi* | AMNH 89788 (Wilkinson et al., 2011) | 0.13 |
| *Rhinatrema bivittatum* | MW 2051 (Wilkinson et al., 2011) | 0.19 |
| *Scolecomorphus kirkii* | NHMUK 1946.9.5.58 (Wilkinson et al., 2011) | 0.19 |
| *Siphonops annulatus* | NHMUK 2005.9 (Wilkinson et al., 2011) | 0.10 |
| *Typhlonectes compressicauda* | MW 5820 (Wilkinson et al., 2011) | 0.12 |

*In the case of *Funcusvermis*, the anteroposterior length of the pseudoangular was measured using the referred pseudoangular specimen PEFO 46480 and the extent of the facet for attachment of the pseudoangular to the pseudodentary in pseudodentary specimen PEFO 46284 (given PEFO 46480 is missing its anterior end).; In the case of *Doleserpeton*, the anteroposterior length of the postdentary bones measured from the anterior terminus of the prearticular to the posterior terminus of the articular was used in absence of the compound pseudoangular.

**Supplementary Table 2. Fossil gymnophionomorph occurrence data**

|  | **Taxon** | **Reference** | **Age** | **Present day location** | **Paleogeography** | **Paleolatitude** | **Minimum number of individuals (MNI)** | **Collecting Method** |
| --- | --- | --- | --- | --- | --- | --- | --- | --- |
| 1 | *Funcusvermis gilmorei* | This study | Middle Norian, ~221 Ma | Arizona, USA | Equatorial Central Pangaea (North American Plate) | ~6° N (Kent & Irving, 2010) | 76 | Screenwashing |
| 2 | *Eocaecilia micropodia* | Jenkins et al., 1993, 2007 | Pliensbachian, ~183.7 Ma | Arizona, USA | Equatorial Central Pangaea (North American Plate) | ~23° N (Bazard & Butler, 1991) | 11 | Quarrying in-situ skeletons |
| 3 | *Rubricacaecilia monbaroni* | Evans & Sigogneau-Russell, 2001 | Berriasian, ~145 – 140 Ma | Figuig, Morocco | Equatorial Gondwana (Africa plate) | ~16° N | 2 | Screenwashing |
| 4 | Gymnophiona indet. | Werner, 1994 | Campanian, ~79.2 Ma | Ash Shamaliyah, Sudan | Equatorial Africa | ~ 3° S | 1 | Screenwashing |
| 5 | Gymnophiona indet. | Gayet et al., 2001 | Maastrichtian, ~68.4 Ma | Cochabamba, Bolivia | Equatorial South America | ~22° S* | 1 | Screenwashing |
| 6 | Gymnophiona indet. | Rage, 1991 | Paleocene, ~64-62 Ma | Mizque, Bolivia | Equatorial South America | ~20° S | 1 | Screenwashing |
| 7 | *Apodops pricei* | Estes and Wake, 1972 | Eocene, ~53-50 Ma | Rio de Janeiro, Brazil | Equatorial South America | ~27° S | 1 | Screenwashing |
| 8 | Gymnophiona indet. | Rage et al., 2021 | Eocene, ~50-45 Ma | Bechar district, Algeria | Equatorial Africa | ~20° N* | 1 | Screenwashing |
| 9 | Teresomata indet. | Rage & Pickford, 2011 | Miocene, ~19-20 Ma | Napak, Uganda | Equatorial Africa | ~5° S* | 1 | Quarrying in-situ skeletons |
| 10 | Gymnophiona indet. | Hecht & LaDuke, 1997 | Miocene, ~13.8-11.6 Ma | Huila, Columbia | Equatorial South America | ~4° S | 1 | Screenwashing |
| 11 | *Dermophis mexicanus* | Wake et al., 1999 | 1200-1350 B.C. to recent | Chiapas, Mexico | Equatorial Central America | ~16° N | 1 | Screenwashing |

Age data derived from Santos et al. (2020). Unless a citation is provided, paleolatitude estimates are derived from the GPlates model (Müller et al., 2018) accessed through the Paleobiology Database (https://paleobiodb.org/#/).

*****Paleolatitude estimated using Paleolatitude.org (Hinsbergen et al., 2015) using the Torsvik et al (2012) paleomagnetic reference frame.

**Supplementary Table 3. Triassic batrachian occurrences**

|  | **Taxon** | **Reference** | **Age** | **Present day location** | **Paleogeography** | **Paleolatitude** |
| --- | --- | --- | --- | --- | --- | --- |
| 1 | *Triadobatrachus massinoti* | Rage & Rocek, 1989 | Late Induan – early Olenekian  252.3 – 247.2 Ma | Diana, Madagascar | Southern Pangaea | ~44° S |
| 2 | *Czatkobatrachus polonicus* | Evans & Borsuk-Bialynicka, 1989 | Olenekian  251.3 to 247.2 Ma | Malopolskie, Poland | Northern Pangaea | ~18° N |
| 3 | *Triassurus sixtelae* | Schoch et al., 2020 | Ladinian  242.0 – 221.5 Ma | Batken, Kyrgystan | Northern Pangaea | ~40° N |
| 4 | Batrachia indet. | Heckert et al., 2012 | Norian  ~225 Ma (Whiteside et al., 2011). | North Carolina, USA | Equatorial Pangaea | ~10° N |
| 5 | Salientia indet. | Stocker et al., 2019 | Norian  223.036 ± 0.059 - 220.123 ± 0.068 Ma (Ramezani et al., 2011) | Arizona, USA | Equatorial Pangaea | ~6° N (Kent & Irving, 2010) |
| 6 | Salientia indet. | Stocker et al., 2019 | Norian   219.39 ± 0.16 Ma (Ramezani et al., 2014) | Arizona, USA | Equatorial Pangaea | ~6° N (Kent & Irving, 2010) |
| 7 | Salientia indet. | Stocker et al., 2019 | Norian  ∼217.7 Ma - 213.870 ± 0.078 Ma (Ramezani et al., 2011) | Arizona, USA | Equatorial Pangea | ~6° N (Kent & Irving, 2010) |

Age and palaeolatitudinal data derived from the Paleobiology Database (https://paleobiodb.org/#/) unless citation is provided.

**Supplementary Table 4: Molecular clock estimates of Gymnophionomorpha-Batrachia divergence without *Gerobatrachus* calibration**

| **Age (Ma)** | **Reference** |
| --- | --- |
| 314.8 | Shen et al., 2012 |
| 316.9 | San Mauro, 2010 |
| 318.1 | Kumazawa, 2007 |
| 319.7 | Shen et al., 2011 |
| 320.9 | Irisarri et al., 2012 |
| 322.0 | Hugall et al., 2007 |
| 323.4 | Okajima & Kumazawa, 2010 |
| 325.6 | Okajima & Kumazawa , 2009 |
| 335.0 | Igawa et al., 2008 |
| 337.0 | Zhang et al., 2005a |
| 341.0 | Zhang et al., 2005b |
| 351.6 | Roelants et al., 2007 |
| 352.0 | Santos et al., 2009 |
| 356.9 | Crottini et al., 2012 |
| 367.0 | San Mauro et al., 2005 |
| **Mean age** = 333.5 Ma | |

Only studies using a monophyletic Lissamphibia topology without the use of *Gerobatrachus hottoni* as a node calibration shown. Reported ages derived from timetree.org.

**Supplementary Table 5: Gymnophionomorpha-Batrachia divergence data with *Gerobatrachus* calibration**

| **Age (Ma)** | **Reference** |
| --- | --- |
| 270.9 | Pyron, 2014 |
| 282 | Marjanović et al., 2007 |
| 294 | Hedges & Kumar, 2009 |
| 295 | Zhang & Wake, 2009a |
| 297.3 | Feng et al., 2017 |
| 297.9 | Laurin et al., 2019 |
| 305.8 | Pyron, 2011 |
| 308 | Zhang & Wake, 2009b |
| 314.8 | Shen et al., 2012 |
| 316.9 | San Mauro, 2010 |
| 317.5 | Bonett et al., 2013 |
| 318.1 | Kumazawa, 2007 |
| 319.7 | Shen et al., 2011 |
| 320.1 | Alfaro et al., 2009 |
| 320.9 | Irisarri et al., 2012 |
| 322 | Hugall et al., 2007 |
| 323.4 | Okajima & Kumazawa, 2010 |
| 325.6 | Okajima & Kumazawa, 2009 |
| 335 | Igawa et al., 2008 |
| 337 | Zhang et al., 2005a |
| 338.1 | Pyron, 2010 |
| 341 | Zhang et al., 2005b |
| 351.6 | Roelants et al., 2007 |
| 352 | Santos et al., 2009 |
| 356.9 | Crottini et al., 2012 |
| 367 | San Mauro et al., 2005 |
| Mean Age = 320.3 Ma |  |

Reported ages derived from timetree.org.

**Supplementary Table 6: Molecular clock estimates of Salientia-Caudata divergence**

| **Age (Ma)** | **Reference** |
| --- | --- |
| 181.5 | Kieren et al., 2018 |
| 232.8 | Alfaro et al., 2009 |
| 247.4 | Blackburn et al., 2010 |
| 262.5 | Pyron, 2014 |
| 263 | Marjanović et al., 2007 |
| 264 | Hedges & Kumar, 2009 |
| 265 | Zhang & Wake, 2009a |
| 266.6 | Ruane et al., 2011 |
| 270.7 | Feng et al., 2017 |
| 271.7 | Laurin et al., 2019 |
| 274 | Hugall et al., 2007 |
| 280.3 | Zhang et al., 2013 |
| 281.9 | Shen et al., 2012 |
| 290.9 | Irisarri et al., 2012 |
| 292 | Pyron, 2011 |
| 292.5 | San Mauro, 2010 |
| 294.9 | Shen et al., 2011 |
| 300 | Bonett et al., 2013 |
| 302.5 | Roquet et al., 2014 |
| 305 | Aris-Brosou & Yang, 2013 |
| 305.7 | Pyron, 2010 |
| 308 | Zhang et al., 2005a |
| 318 | Igawa et al., 2008 |
| 321 | Zhang et al., 2005b |
| 322 | Vieites et al., 2007 |
| 325.8 | Delsuc et al., 2018 |
| 332.9 | Roelants et al., 2007 |
| 339.1 | Santos et al., 2009 |
| 344.4 | Crottini et al., 2012 |
| 357 | San Mauro et al., 2005 |
| **Mean Age** = 290.4 Ma | |

Reported ages derived from timetree.org.

**Supplementary Table 7: Rhinatrematidae-Stegokrotaphia divergence data**

| 87.9 | Alfaro et al., 2009 |
| --- | --- |
| 97.9 | Pyron, 2011 |
| 108.7 | Pyron, 2014 |
| 115 | Hugall et al., 2007 |
| 125.1 | Pyron, 2010 |
| 188.4 | Kamei et al., 2012 |
| 212.7 | San Mauro, 2010 |
| 214 | San Mauro et al., 2005 |
| 217.8 | Roelants et al., 2007 |
| 224.7 | Hedges et al., 2015 |
| 226.4 | Hedges & Kumar, 2009 |
| 228 | Zhang & Wake, 2009b |
| 239.1 | San Mauro et al., 2014 |
| 242 | Zhang & Wake, 2009a |
| 246.5 | Santos et al., 2009 |
| 267 | Igawa et al., 2008 |
| **Mean Age** = 190.1 Ma | |

Reported ages derived from timetree.org.

**Supplementary Table 8. Micro-computed tomographic scan parameters**

| Specimen | Resolution | Source Voltage | Source Current | Scanner |
| --- | --- | --- | --- | --- |
| PEFO 44432 | x, y, and z = 3.83 μm | 62 kV | 159 μA | Skyscan 1172 (VT ICTAS) |
| PEFO 43811 | x, y, and z = 5.92 μm | 71 kV | 131 μA | Skyscan 1172 (VT ICTAS) |
| PEFO 45800 | x, y, and z = 5.92 μm | 80 kV | μA 100 | Skyscan 1172 (VT ICTAS) |
| PEFO 45910 | x, y, and z = 5.57 μm | 55 kV | 179 μA | Skyscan 1172 (VT ICTAS) |
| PEFO 46284 | x, y, and z = 5.92 μm | 71 kV | 131 μA | Skyscan 1172 (VT ICTAS) |
| PEFO 46480 | x, y, and z = 3.66 μm | 100 kV | 100 μA | Skyscan 1172 (VT ICTAS) |
| PEFO 46481 | x, y, and z = 3.83 μm | 58 kV | 171 μA | Skyscan 1172 (VT ICTAS) |
| PEFO 43891 | x, y, and z = 10.2 μm | 201 kV | 50 μA | Nikon XTH 225 ST (Duke SMIF) |

**Supplementary Table 9. Character and character state coding for *Funcusvermis gilmorei***

| Character Number | Character Name | Character State |
| --- | --- | --- |
| 76 | Dentition (marginal) | 1: homogeneous, small teeth, equidistant |
| 77 | Dentition (marginal, tooth bases). | 0: round or oval |
| 79 | Pedicely | 1: at least some teeth with two separate mineralization centers (pedicellate) |
| 81 | Labyrinthodonty | 1: never labyrinthodont |
| 136 | Postglenoid area | 0: absent or present as very faint outgrowth |
| 138 | Postglenoid area (dorsal) | 0: plain |
| 139 | Hamate process | 1: present but lower than postglenoid portion is long |
| 140 | Preglenoid process | 0: labial side of surangular with straight dorsal margin anterior to glenoid |
| 141 | Meckelian window | 0: small round or oval opening |
| 142 | Symphyseal teeth | 2: a row aligned parallel to the labial tooth row |
| 164 | Pleurocentrum (presence) | 0: ossified |
| 166 | Pleurocentrum (ventral extension) | 2: ventrally fused to form a single cylindrical element |
| 201 | Femur (trochanter) | 0: internal trochanter present as discrete process |
| 239 | Teeth laterally compressed | 0: no |
| 247 | Denticles on palatines | 1: absent |
| 254 | Dentary | 0: long |
| 255 | Dentary forms coronoid process | 0: absent |
| 258 | Number of splenials | 2: 0 |
| 260 | Meckelian fossae | 1: 1 |
| 263 | Articulation to tooth row | 0: above |
| 266 | Coronoid teeth | 0: present |
| 269 | Jaw sculpture | 1: absent |
| 273 | Trunk neural arch to centrum | 2: fused |
| 315 | Tentacle | 0: absent |
| 338 | Dentary marginal dentition | 1: multiple rows |
| 345 | Continuous tooth row lateral to choana. | 0: absent |
| 349 | Palatine in adults | 1: present but coossified to maxilla to form maxillopalatine |
| 354 | Cranio-mandibular articulation surface of the mandible | 0: dorsally facing planar circular or subcircular pad |
| 355 | Pseudoangular | 1: present |

**6. Institutional abbreviations**

AMNH, American Museum of Natural History, New York, New York, U.S.A.; FMNH, Field Museum of Natural History, Chicago, Illinois, U.S.A.; MCZ, Museum of Comparative Zoology, Harvard University, Cambridge, Massachusetts, U.S.A.; MNA, Museum of Northern Arizona, Flagstaff, Arizona, U.S.A.; NHMUK, Natural History Museum, London, England; PEFO, Petrified Forest National Park, Arizona, U.S.A.; MW, field series (NHMUK); SMNS, Staatliches Museum für Naturkunde, Stuttgart, Germany; USNM, United States National Museum, Smithsonian Institution, Washington D.C., U.S.A.; ZPAL, Institute of Paleobiology of the Polish Academy of Science, Warsaw, Poland.

**7. Supplementary References**

Ahlberg, P. E. & Clack, J. A. Lower jaws, lower tetrapods–a review based on the Devonian genus *Acanthostega*. *Earth and Environmental Science Transactions of The Royal Society of Edinburgh* **89**, 11-46 (1998).

Alfaro, M. E. *et al.* Nine exceptional radiations plus high turnover explain species diversity in jawed vertebrates. *Proceedings of the National Academy of Sciences* **106**, 13410-13414 (2009).

Anderson, J. S. On the skull of *Cacops aspidephorus* Williston (Tetrapoda; Temnospondyli; Dissorophidae) from the Lower Permian of Texas. Part 1: lower jaw anatomy. *The Nonmarine Permian, New Mexico Museum of Natural History & Science Bulletin* **30**, 15 (2005).

Anderson, J. S. & Bolt, J. R. New information on amphibamids (Tetrapoda, Temnospondyli) from Richards Spur (Fort Sill), Oklahoma. *Journal of Vertebrate Paleontology* **33**, 553-567 (2013).

Anderson, J. S., Henrici, A. C., Sumida, S. S., Martens, T. & Berman, D. S. *Georgenthalia clavinasica*, a new genus and species of dissorophoid temnospondyl from the Early Permian of Germany, and the relationships of the family Amphibamidae. *Journal of Vertebrate Paleontology* **28**, 61-75 (2008a).

Anderson, J. S., Reisz, R. R., Scott, D., Fröbisch, N. B. & Sumida, S. S. A stem batrachian from the Early Permian of Texas and the origin of frogs and salamanders. *Nature* **453**, 515-518 (2008b).

Anderson, J. S., Scott, D. & Reisz, R. R. *Nannaroter mckinziei*, a new ostodolepid ‘microsaur’(Tetrapoda, Lepospondyli, Recumbirostra) from the Early Permian of Richards Spur (Ft. Sill), Oklahoma. *Journal of Vertebrate Paleontology* **29**, 379-388 (2009).

Anderson, J. S., Scott, D. & Reisz, R. R. The anatomy of the dermatocranium and mandible of *Cacops aspidephorus* Williston, 1910 (Temnospondyli: Dissorophidae), from the Lower Permian of Texas. *Journal of Vertebrate Paleontology* **40**, e1776720 (2020).

Arbez, T., Dahoumane, A. & Steyer, J.-S. Exceptional endocranium and middle ear of *Stanocephalosaurus* (Temnospondyli: Capitosauria) from the Triassic of Algeria revealed by micro-CT scan, with new functional interpretations of the hearing system. *Zoological Journal of the Linnean Society* **180**, 910-929 (2017).

Arbez, T., Atkins, J.B., & Maddin, H.C. Cranial anatomy and systematics of *Dendrerpeton* cf. *helogenes* (Tetrapoda, Temnospondyli) from the Pennsylvanian of Joggins, revisited through micro‐CT scanning. *Papers in Palaeontology* **8**, e1421 (2022).

Aris-Brosou, S. & Yang, Z. Effects of models of rate evolution on estimation of divergence dates with special reference to the metazoan 18S ribosomal RNA phylogeny. *Systematic Biology* **51**, 703-714 (2002).

Ascarrunz, E., Rage, J.-C., Legreneur, P. & Laurin, M. *Triadobatrachus massinoti*, the earliest known lissamphibian (Vertebrata: Tetrapoda) re-examined by μCT scan, and the evolution of trunk length in batrachians. *Contributions to Zoology* **85**, 201-234 (2016).

Barrett, M., Donoghue, M. J. & Sober, E. Against consensus. *Systematic Zoology* **40**, 486-493 (1991).

Bazard, D. R. & Butler, R. F. Paleomagnetism of the Chinle and Kayenta Formations, New Mexico and Arizona. *Journal of Geophysical Research: Solid Earth* **96**, 9847-9871 (1991).

Bemis, W. E., Schwenk, K. & Wake, M. Morphology and function of the feeding apparatus in *Dermophis mexicanus* (Amphibia: Gymnophiona). *Zoological Journal of the Linnean Society* **77**, 75-96 (1983).

Berman, D. S. A trimerorhachid amphibian from the Upper Pennsylvanian of New Mexico. *Journal of Paleontology* **47**, 932-945 (1973).

Berman, D. S., Reisz, R. R. & Eberth, D. A. *Ecolsonia cutlerensis*, an Early Permian dissorophid amphibian from the Cutler Formation of north-central New Mexico. *New Mexico Bureau of Mines & Mineral Resources Circular* **191**, 31 (1985).

Billo, R. & Wake, M. H. Tentacle development in *Dermophis mexicanus* (Amphibia, Gymnophiona) with an hypothesis of tentacle origin. *Journal of Morphology* **192**, 101-111 (1987).

Bjerring, H. C. A new amphibious tetrapod from the Greenlandic Eotriassic. *Meddelelser om Grønland, Geosciences* **38**, 1-42 (1999).

Blackburn, D. C., Bickford, D. P., Diesmos, A. C., Iskandar, D. T. & Brown, R. M. An ancient origin for the enigmatic flat-headed frogs (Bombinatoridae: *Barbourula*) from the islands of Southeast Asia. *PLoS One* **5**, e12090 (2010).

Bolt, J. in *Origins of the Higher Groups of Tetrapods. Controversy and Consensus* (eds H. P. Schultze and L. Trueb) 194-222 (Comstock Publishing Associates, 1991).

Bolt, J. R. Lissamphibian origins: possible protolissamphibian from the Lower Permian of Oklahoma. *Science* **166**, 888-891 (1969).

Bolt, J. R. A trematopsid skull from the Lower Permian, and analysis of some characters of the dissorophoid (Amphibia: Labyrinthodontia) otic notch. *Fieldiana: Geology* **30**, 67-79 (1974).

Bolt, J. R. Dissorophoid relationships and ontogeny, and the origin of the Lissamphibia. *Journal of Paleontology* **51**, 235-249 (1977).

Bolt, J. R. in *Mazon Creek fossils* (ed M H Nitecki) 529-563 (Academic Press, 1979).

Bolt, J. R. & Chatterjee, S. A new temnospondyl amphibian from the Late Triassic of Texas. *Journal of Paleontology* **74**, 670-683 (2000).

Bolt, J. R. & Lombard, R. E. The mandible of the primitive tetrapod *Greererpeton*, and the early evolution of the tetrapod lower jaw. *Journal of Paleontology* **75**, 1016-1042 (2001).

Bolt, J. R. & Lombard, R. E. *Sigournea multidentata*, a new stem tetrapod from the Upper Mississippian of Iowa, USA. *Journal of Paleontology* **80**, 717-725 (2006).

Bolt, J. R. & Wassersug, R. J. Functional morphology of the skull in *Lysorophus*: a snake-like Paleozoic amphibian (Lepospondyli). *Paleobiology* **1**, 320-332 (1975).

Bonett, R. M., Trujano-Alvarez, A. L., Williams, M. J. & Timpe, E. K. Biogeography and body size shuffling of aquatic salamander communities on a shifting refuge. *Proceedings of the Royal Society B: Biological Sciences* **280**, 20130200 (2013).

Boulenger, G. No. XVI.-A list of the freshwater fishes, batrachians, and reptiles obtained by Mr. J. Stanley Gardiner’s expedition to the Indian Ocean. *Transactions of the Linnean Society of London. 2nd Series: Zoology* **12**, 291-300 (1909).

Boy, J. & Sues, H. in *Amphibian biology* Vol. 4 (eds H Heatwole & RL Carroll) 1150-1197 (2000).

Boy, J. A. Über *Micropholis*, den letzten Überlebenden der Dissorophoidea (Amphibia, Temnospondyli; Unter-Trias). *Neues Jahrbuch für Geologie und Paläontologie-Monatshefte* **1985**, 29-45 (1985).

Boy, J A. Studien über die Branchiosauridae (Amphibia: Temnospondyli; Ober-Karbon-Unter-Perm). 2. Systematische Übersicht. *Neues Jahrbuch für Geologie und Paläontologie. Abhandlungen* **174**, 75-104 (1987).

Boy, J. A. Über einige Vertreter der Eryopoidea (Amphibia: Temnospondyli) aus dem europäischen Rotliegend (? höchstes Karbon—Perm) 1. *Sclerocephalus*. *Paläontologische Zeitschrift* **62**, 107-132 (1988).

Boy, J. A. Über einige Vertreter der Eryopoidea (Amphibia: Temnospondyli) aus dem europäischen Rotliegend (? höchstes Oberkarbon—Perm) 2. *Acanthostomatops*. *Paläontologische Zeitschrift* **63**, 133-151 (1989).

Boy, J. A. Über einige Vertreter der Eryopoidea (Amphibia: Temnospondyli) aus dem europäischen Rotliegend (? höchstes Karbon-Perm) 3. *Onchiodon*. *Paläontologische Zeitschrift* **64**, 287-312 (1990).

Boy, J. A. Über einige vertreter der Eryopoidea (Amphibia: Temnospondyli) aus dem europäischen Rotliegend (? höchstes Karbon-Perm) 4. *Cheliderpeton latirostre*. *Paläontologische Zeitschrift* **67**, 123-143 (1993).

Boy, J. A. Über die Micromelerpetontidae (Amphibia: Temnospondyli). 1. Morphologie und Paläoökologie des *Micromelerpeton credneri* (Unter-Perm; SW-Deutschland). *Paläontologische Zeitschrift* **69**, 429-457 (1995).

Brazeau, M. D. Problematic character coding methods in morphology and their effects. *Biological Journal of the Linnean Society* **104**, 489-498 (2011).

Broili, F. & Schröder, J. Beobachtungen an wirbeltieren der Karrooformation. *Sitzungsberichte der Bayerischen Akademie der Wissenschaften, Mathematisch-Naturwissenschaftliche Abteilung* **1937**, 19-37 (1937).

Broom, R. Notes on some labyrinthodonts in the Transvaal Museum. *Annals of the Transvaal Museum* **14**, 1-10 (1930).

Brusatte, S. L., Butler, R. J., Mateus, O. & Steyer, J. S. A new species of *Metoposaurus* from the Late Triassic of Portugal and comments on the systematics and biogeography of metoposaurid temnospondyls. *Journal of Vertebrate Paleontology* **35**, e912988 (2015).

Buffa, V., Jalil, N. E. & Steyer, J. S. Redescription of *Arganasaurus* (*Metoposaurus*) *azerouali* (Dutuit) comb. nov. from the Upper Triassic of the Argana Basin (Morocco), and the first phylogenetic analysis of the Metoposauridae (Amphibia, Temnospondyli). *Papers in Palaeontology* **5**, 699-717 (2019).

Bystrow, A. *Dvinosaurus* als neotenische Form der Stegocephalen. *Acta Zoologica* **19**, 209-295 (1938).

Bystrow, A. & Efremov, J. *Benthosuchus sushkini* Efr.—a labyrinthodont from the Eotriassic of Sharzhenga River. *Trudy Paleontologicheskogo Instituta* **10**, 1-152 (1940).

Carlson, K. J. *Perryella*, a new temnospondylous amphibian from the Lower Permian of Oklahoma. *Journal of Paleontology* **61**, 135-147 (1987).

Carroll, R. L. Early Evolution of the Dissorophid Amphibians. *Bulletin of the Museum of Comparative Zoology* **131**, 161-250 (1964).

Carroll, R. L. A tiny microsaur from the Lower Permian of Texas: size constraints in Palaeozoic tetrapods. *Palaeontology* **33**, 893-909 (1990).

Carroll, R. L. *Batropetes* from the Lower Permian of Europe—a microsaur, not a reptile. *Journal of Vertebrate Paleontology* **11**, 229-242 (1991).

Carroll, R. L. The Palaeozoic ancestry of salamanders, frogs and caecilians. *Zoological Journal of the Linnean Society* **150**, 1-140 (2007).

Carroll, R. L. & Gaskill, P. The Order Microsauria *Memoirs of the American Philosophical Society* **126**, 1-211 (1978).

Carroll, R. L. & Holmes, R. The skull and jaw musculature as guides to the ancestry of salamanders. *Zoological Journal of the Linnean Society* **68**, 1-40 (1980).

Case, E. C. Notes on the skull of *Lysorophus tricarinatus* Cope. . *Bulletin of the American Museum of Natural History* **24**, 1-26 (1908).

Case, E. C. Description of a new species of *Buettneria*, with a discussion of the brain case. *Contributions from the Museum of Paleontology, University of Michigan* **3**, 18-206 (1931).

Case, E. C. Description of a collection of associated skeletons of *Trimerorhachis*. *Contributions from the Museum of Paleontology, University of Michigan* **4**, 227-274 (1935).

Casey, J. & Lawson, R. A histological and scanning electron microscope study of the teeth of caecilian amphibians. *Archives of Oral Biology* **26**, 49-58 (1981).

Chernin, S. A new brachyopid, *Batrachosuchus concordi* sp. nov. from the Upper Luangwa Valley, Zambia with a redescription of *Batrachosuchus browni* Broom, 1903. *Palaeontologia Africana* **20**, 87-109 (1977).

Chuliver, M. & Scanferla, A. Morphology and postnatal ontogeny of the dentition of *Chthonerpeton indistinctum* (Gymnophiona: Typhlonectidae). *Amphibia-Reptilia* **40**, 327-336 (2019).

Clack, J. A., Ahlberg, P. E., Blom, H. & Finney, S. M. A new genus of Devonian tetrapod from North‐East Greenland, with new information on the lower jaw of *Ichthyostega*. *Palaeontology* **55**, 73-86 (2012).

Clack, J. A. & Anderson, J. S. in *Evolution of the Vertebrate Ear* (eds J. A. Clack, R. R. Fay, & A. N. Popper) 71-105 (Springer, 2016).

Clack, J. A. & Milner, A. R. Morphology and systematics of the Pennsylvanian amphibian *Platyrhinops lyelli* (Amphibia: Temnospondyli). *Earth and Environmental Science Transactions of the Royal Society of Edinburgh* **100**, 275-295 (2010).

Cosgriff, J. Lower Triassic Temnospondyli from the Triassic of Western Australia. *Geological Society of America, Special Paper* **14**, 1-131 (1974).

Cosgriff, J. & Garbutt, N. *Erythrobatrachus noonkanbahensis*, a trematosaurid species from the Blina Shale. *Journal of the Royal Society of Western Australia* **55**, 5-18 (1972).

Crottini, A. *et al.* Vertebrate time-tree elucidates the biogeographic pattern of a major biotic change around the K–T boundary in Madagascar. *Proceedings of the National Academy of Sciences* **109**, 5358-5363 (2012).

Daly, E. Amphibamidae (Amphibia: Temnospondyli), with a description of a new genus from the upper Pennsylvanian of Kansas. *University of Kansas Museum of Natural History Miscellaneous Publications* **85**, 1-59 (1994).

Damiani, R. A giant skull of the temnospondyl *Xenotosuchus africanus* from the Middle Triassic of South Africa and its ontogenetic implications. *Acta Palaeontologica Polonica* **53**, 75-84 (2008).

Damiani, R., Schoch, R. R., Hellrung, H., Werneburg, R. & Gastou, S. The plagiosaurid temnospondyl *Plagiosuchus pustuliferus* (Amphibia: Temnospondyli) from the Middle Triassic of Germany: anatomy and functional morphology of the skull. *Zoological Journal of the Linnean Society* **155**, 348-373 (2009).

Damiani, R. J. A systematic revision and phylogenetic analysis of Triassic mastodonsauroids (Temnospondyli: Stereospondyli). *Zoological Journal of the Linnean Society* **133**, 379-482 (2001).

Damiani, R. J. & Jeannot, A. M. A brachyopid temnospondyl from the lower Cynognathus Assemblage Zone in the northern Karoo Basin, South Africa. *Palaeontologia Africana* **38**, 57-69 (2002).

Damiani, R. J. & Kitching, J. W. A new brachyopid temnospondyl from the Cynognathus Assemblage Zone, Upper Beaufort Group, South Africa. *Journal of Vertebrate Paleontology* **23**, 67-78 (2003).

Damiani, R. J. & Warren, A. A new look at members of the Superfamily Brachyopoidea (Amphibia, Temnospondyli) from the Early Triassic of Queensland and a preliminary analysis of brachyopoid relationships. *Alcheringa* **20**, 277-300 (1996).

Damiani, R. J. & Yates, A. M. The Triassic amphibian *Thoosuchus yakovlevi* and the relationships of the Trematosauroidea (Temnospondyli: Stereospondyli). *Records-Australian Museum* **55**, 331-342 (2003).

Danto, M., Witzmann, F. & Fröbisch, N. B. Vertebral development in Paleozoic and Mesozoic tetrapods revealed by paleohistological data. *PloS One* **11**, e0152586 (2016).

Danto, M., Witzmann, F., Pierce, S. E. & Fröbisch, N. B. Intercentrum versus pleurocentrum growth in early tetrapods: A paleohistological approach. *Journal of Morphology* **278**, 1262-1283 (2017).

Davit‐Béal, T., Chisaka, H., Delgado, S. & Sire, J. Y. Amphibian teeth: current knowledge, unanswered questions, and some directions for future research. *Biological Reviews* **82**, 49-81 (2007).

Daza, J. D. *et al.* Enigmatic amphibians in mid-Cretaceous amber were chameleon-like ballistic feeders. *Science* **370**, 687-691 (2020).

Delsuc, F. *et al.* A phylogenomic framework and timescale for comparative studies of tunicates. *BMC biology* **16**, 1-14 (2018).

DeMar, R. The Permian labyrinthodont amphibian *Dissorophus multicinctus*, and adaptations and phylogeny of the family Dissorophidae. *Journal of Paleontology* **42**, 1210-1242 (1968).

Dias, E. V., Dias-da-Silva, S. & Schultz, C. L. A new short-snouted rhinesuchid from the Permian of southern Brazil. *Revista Brasileira de Paleontologia* **23**, 98-122 (2020).

Dias, E. V. & Schultz, C. L. The first Paleozoic temnospondyl postcranial skeleton from South America. *Revista Brasileira de Paleontologia* **6**, 29-42 (2003).

Dias-da-Silva, S., Marsicano, C. & Schultz, C. L. . Rhytidosteid temnospondyls in Gondwana: a new taxon from the Lower Triassic of Brazil. *Palaeontology* **49**, 381-390 (2006).

Dias-da-Silva, S. & Marsicano, C. Phylogenetic reappraisal of Rhytidosteidae (Stereospondyli: Trematosauria), temnospondyl amphibians from the Permian and Triassic. *Journal of Systematic Palaeontology* **9**, 305-325 (2011).

Dias-da-Silva, S., Marsicano, C. & Schultz, C. L. Early Triassic temnospondyl skull fragments from southern South America (Paraná Basin, Brazil). *Revista Brasileira de Paleontologia* **8**, 165-172 (2005).

Dilkes, D. Carpus and tarsus of Temnospondyli. *Vertebrate Anatomy Morphology Palaeontology* **1**, 51-87 (2015).

Dilkes, D. & Brown, L. E. Biomechanics of the vertebrae and associated osteoderms of the Early Permian amphibian *Cacops aspidephorus*. *Journal of Zoology* **271**, 396-407 (2007).

Dilkes, D. W. A new trematopsid amphibian (Temnospondyli: Dissorophoidea) from the Lower Permian of Texas. *Journal of Vertebrate Paleontology* **10**, 222-243 (1990).

Dilkes, D. W. Comparison and biomechanical interpretations of the vertebrae and osteoderms of *Cacops aspidephorus* and *Dissorophus multicinctus* (Temnospondyli, Dissorophidae). *Journal of Vertebrate Paleontology* **29**, 1013-1021 (2009).

Dilkes, D. W. Revision of the Early Permian Dissorophid ‘*Dissorophus*’ *angustus* (Temnospondyli: Dissorophoidea). *Journal of Vertebrate Paleontology* **40**, e1801704 (2020).

Dilkes, D. W. & Reisz, R. *Trematops milleri* Williston, 1909, identified as a junior synonym of *Acheloma cumminsi* Cope, 1882: with a revision of the genus. *American Museum Novitates* **2902** (1987).

Dutuit, J. Découverte de pleurocentres dans les vertébrés de stégocephales métoposaurides. *Compte Rendu de l'Academie des*

*Sciences* **274**, 536-537 (1972).

Dutuit, J.-M. Introduction à l'étude paléontologique du Trias continental marocain. Description des premiers stegocephales recueillis dans le couloir d'Argana (Atlas Occidental). *Mémoires du Muséum National d’Histoire* **36**, 1-253 (1976).

Eltink, E., Schoch, R. R. & Langer, M. C. Interrelationships, palaeobiogeography and early evolution of Stereospondylomorpha (Tetrapoda: Temnospondyli). *Journal of Iberian Geology* **45**, 251-267 (2019).

Englehorn, J., Small, B. J. & Huttenlocker, A. A redescription of *Acroplous vorax* (Temnospondyli: Dvinosauria) based on new specimens from the Early Permian of Nebraska and Kansas, USA. *Journal of Vertebrate Paleontology* **28**, 291-305 (2008).

Estes, R. & Wake, M. H. The first fossil record of caecilian amphibians. *Nature* **239**, 228-231 (1972).

Evans, S. E. & Borsuk-Białynicka, M. A stem-group frog from the Early Triassic of Poland. *Acta Palaeontologica Polonica* **43**, 573-580 (1998).

Evans, S. E. & Sigogneau‐Russell, D. A stem‐group caecilian (Lissamphibia: Gymnophiona) from the Lower Cretaceous of North Africa. *Palaeontology* **44**, 259-273 (2001).

Feng, Y.-J. *et al.* Phylogenomics reveals rapid, simultaneous diversification of three major clades of Gondwanan frogs at the Cretaceous–Paleogene boundary. *Proceedings of the National Academy of Sciences* **114**, E5864-E5870 (2017).

Fernández-Coll, M., Arbez, T., Bernardini, F. & Fortuny, J. Cranial anatomy of the Early Triassic trematosaurine *Angusaurus* (Temnospondyli: Stereospondyli): 3D endocranial insights and phylogenetic implications. *Journal of Iberian Geology* **45**, 269-286 (2019).

Fraas, E. Die Labyrinthodonten der Schwäbischen Trias. *Palaeontographica* **36**, 1–158 (1889).

Fröbisch, N. B. & Reisz, R. R. A new species of dissorophid (*Cacops woehri*) from theLower Permian Dolese quarry, near Richards Spur, Oklahoma. *Journal of Vertebrate Paleontology* **32**, 35-44 (2012).

Gao, K.-Q. & Shubin, N. H. Late Jurassic salamanders from northern China. *Nature* **410**, 574-577 (2001).

Gardner, J. D. New albanerpetontid amphibians from the Albian to Coniacian of Utah, USA—bridging the gap. *Journal of Vertebrate Paleontology* **19**, 632-638 (1999).

Gardner, J. D. Revised taxonomy of albanerpetontid amphibians. *Acta Palaeontologica Polonica* **45**, 55-70 (2000).

Gardner, J. D. Monophyly and affinities of albanerpetontid amphibians (Temnospondyli;Lissamphibia). *Zoological Journal of the Linnean Society* **131**, 309-352 (2001).

Gardner, J. D. & Averianov, A. O. Albanerpetontid amphibians from the Upper Cretaceous of Middle Asia. *Acta Palaeontologica Polonica* **43**, 453-476 (1998).

Gardner, J. D., Evans, S. E. & Sigogneau-Russell, D. New albanerpetontid amphibians from the Early Cretaceous of Morocco and Middle Jurassic of England. *Acta Palaeontologica Polonica* **48** (2003).

Gayet, M. *et al.* Middle Maastrichtian vertebrates (fishes, amphibians, dinosaurs and other reptiles, mammals) from Pajcha Pata (Bolivia). Biostratigraphic, palaeoecologic and palaeobiogeographic implications. *Palaeogeography, Palaeoclimatology, Palaeoecology* **169**, 39-68 (2001).

Gee, B. M. Returning to the roots: resolution, reproducibility, and robusticity in the phylogenetic inference of Dissorophidae (Amphibia: Temnospondyli). *PeerJ* **9**, e12423 (2021).

Gee, B. M., Bevitt, J. J. & Reisz, R. R. A juvenile specimen of the trematopid *Acheloma* from Richards Spur, Oklahoma and challenges of trematopid ontogeny. *Frontiers in Earth Science* **7**, 38 (2019).

Gee, B. M. & Reisz, R. R. Cranial and postcranial anatomy of *Cacops morrisi*, a eucacopine dissorophid from the early Permian of Oklahoma. *Journal of Vertebrate Paleontology* **38**, e1433186 (2018).

Gee, B. M. & Reisz, R. R. A redescription of the late Carboniferous trematopid *Actiobates peabodyi* from Garnett, Kansas. *The Anatomical Record* **303**, 2821-2838 (2020).

Glienke, S. A taxonomic revision of *Batropetes* (Amphibia, Microsauria) from the Rotliegend (basal Permian) of Germany. *Neues Jahrbuch für Geologie und Paläontologie-Abhandlungen* **269**, 73-96 (2013).

Glienke, S. Two new species of the genus Batropetes (Tetrapoda, Lepospondyli) from theCentral European Rotliegend (basal Permian) in Germany. *Journal of Vertebrate Paleontology* **35**, e918041 (2015).

Godfrey, S. J., Fiorillo, A. R. & Carroll, R. L. A newly discovered skull of the temnospondyl amphibian *Dendrerpeton acadianum* Owen. *Canadian Journal of Earth Sciences* **24**, 796-805 (1987).

Goodman, C. M. *et al.* A Case of Mistaken Identity: Genetic and Anatomical Evidence Reveals the Cryptic Invasion of *Xenopus tropicalis* in Central Florida. *Journal of Herpetology* **55**, 62-69 (2021).

Haughton, S. Descriptive catalogue of the Amphibia of the Karroo system. *Annals of the South African Museum* **12**, 65-77 (1925).

Hecht M. K., & Laduke, T. C. in *Vertebrate paleontology in the Neotropics: The Miocene fauna of La Venta, Colombia* (eds Madden R H Kay R F, Cifelli R L, Flynn J J) 95–99 (Smithsonian Institution Press, 1997).

Heckert, A. B., Mitchell, J. S., Schneider, V. P. & Olsen, P. E. Diverse new microvertebrate assemblage from the Upper Triassic Cumnock Formation, Sanford Subbasin, North Carolina, USA. *Journal of Paleontology* **86**, 368-390 (2012).

Hedges, S. B. & Kumar, S. *The timetree of life*. (Oxford University Press, 2009).

Hedges, S. B., Marin, J., Suleski, M., Paymer, M. & Kumar, S. Tree of life reveals clock-like speciation and diversification. *Molecular biology and evolution* **32**, 835-845 (2015).

Hellrung, H. *Gerrothorax pustuloglomeratus*, ein Temnospondyle (Amphibia) mit knöcherner Branchialkammer aus dem Unteren Keuper von Kupferzell (Süddeutschland). *Stuttgarter Beiträge zur Naturkunde Ser. B* **330**, 1-130 (2003).

Henderson, A. C. *Studies on Interactions Among Xenopus Taxa Using Comparative Osteology and Other Methods: An Evolutionary Perspective*, University of Bristol, (2002).

Hewison, R. The skull and mandible of the stereospondyl *Lydekkerina huxleyi*, (Tetrapoda: Temnospondyli) from the Lower Triassic of South Africa, and a reappraisal of the family Lydekkerinidae, its origin, taxonomic relationships and phylogenetic importance. *Journal of Temnospondyl Palaeontology* **1**, 1-80 (2007).

Holder, M. T., Sukumaran, J. & Lewis, P. O. A justification for reporting the majority-rule consensus tree in Bayesian phylogenetics. *Systematic biology* **57**, 814-821 (2008).

Holmes, R. The Carboniferous amphibian *Proterogyrinus scheelei* Romer, and the early evolution of tetrapods. *Philosophical Transactions of the Royal Society of London. B, Biological Sciences* **306**, 431-524 (1984).

Holmes, R., Berman, D. S. & Anderson, J. S. A new dissorophid (Temnospondyli, Dissorophoidea) from the Early Permian of New Mexico (United States). *Comptes Rendus Palevol* **12**, 419-435 (2013).

Holmes, R. B., Carroll, R. L. & Reisz, R. R. The first articulated skeleton of *Dendrerpeton acadianum* (Temnospondyli, Dendrerpetontidae) from the Lower Pennsylvanian locality of Joggins, Nova Scotia, and a review of its relationships. *Journal of Vertebrate Paleontology* **18**, 64-79 (1998).

Hook, R. W. *Chenoprosopus lewisi*, a new cochleosaurid amphibian (Amphibia: Temnospondyli) from the Permo-Carboniferous of north-central Texas. *Annals of the Carnegie Museum* **62**, 273-291 (1993).

Hook, R. W. & Baird, D. The diamond coal mine of Linton, Ohio, and its Pennsylvanian-age vertebrates. *Journal of Vertebrate Paleontology* **6**, 174-190 (1986).

Howie, A. A. A new capitosaurid labyrinthodont from East Africa. *Palaeontology* **13**, 210-253 (1970).

Hugall, A. F., Foster, R. & Lee, M. S. Calibration choice, rate smoothing, and the pattern of tetrapod diversification according to the long nuclear gene RAG-1. *Systematic Biology* **56**, 543-563 (2007).

Hunt, A. P. Revision of the Metoposauridae (Amphibia: Temnospobdyli) and description of a new genus from Western North America. *Museum of Northern Arizona Bulletin* **59**, 67-97 (1993).

Huttenlocker, A. K., Pardo, J. D. & Small, B. J. *Plemmyradytes shintoni*, gen. et sp. nov., an Early Permian amphibamid (Temnospondyli: Dissorophoidea) from the Eskridge Formation, Nebraska. *Journal of Vertebrate Paleontology* **27**, 316-328 (2007).

Igawa, T., Kurabayashi, A., Usuki, C., Fujii, T. & Sumida, M. Complete mitochondrial genomes of three neobatrachian anurans: a case study of divergence time estimation using different data and calibration settings. *Gene* **407**, 116-129 (2008).

Irisarri, I. *et al.* The origin of modern frogs (Neobatrachia) was accompanied by acceleration in mitochondrial and nuclear substitution rates. *BMC Genomics* **13**, 1-19 (2012).

Ivachnenko, M. Urodelans from the Triassic and Jurassic of Soviet central Asia. *Paleontological Journal* **12**, 362-368 (1978).

Jared, C. *et al.* Skin gland concentrations adapted to different evolutionary pressures in the head and posterior regions of the caecilian *Siphonops annulatus*. *Scientific reports* **8**, 1-7 (2018).

Jeannot, A. M., Damiani, R. & Rubidge, B. S. Cranial anatomy of the Early Triassic stereospondyl *Lydekkerina huxleyi* (Tetrapoda: Temnospondyli) and the taxonomy of South African lydekkerinids. *Journal of Vertebrate Paleontology* **26**, 822-838 (2006).

Jenkins, F. A., Walsh, D. M. & Carroll, R. L. Anatomy of *Eocaecilia micropodia*, alimbed caecilian of the Early Jurassic. *Bulletin of the Museum of Comparative Zoology* **158**, 285-365 (2007).

Jenkins Jr, F. A., Shubin, N. H., Gatesy, S. M. & Warren, A. *Gerrothorax pulcherrimus* from the Upper Triassic Fleming Fjord Formation of East Greenland and a reassessment of head lifting in temnospondyl feeding. *Journal of Vertebrate Paleontology* **28**, 935-950 (2008).

Jenkins, P. A. & Walsh, D. M. An Early Jurassic caecilian with limbs. *Nature* **365**, 246-250 (1993).

Jessen, H. Die Crossopterygier des Oberen Plattenkalkes (Devon) der Bergisch-Gladbach-Paffrather Mulde (Rheinisches Schiefergebirge) unter Berucksichtigung von Amerikanischem und Europaischem *Onychodus*-material. *Arkiv för Zoologi* **18**, 305-389 (1965).

Jia, J. & Gao, K.-Q. A new hynobiid-like salamander (Amphibia, Urodela) from Inner Mongolia, China, provides a rare case study of developmental features in an Early Cretaceous fossil urodele. *PeerJ* **4**, e2499 (2016).

Jia, J. & Gao, K.-Q. A new basal salamandroid (Amphibia, Urodela) from the Late Jurassic of Qinglong, Hebei province, China. *PloS One* **11**, e0153834 (2016).

Jia, J. & Gao, K.-Q. A new stem hynobiid salamander (Urodela, Cryptobranchoidea) from the Upper Jurassic (Oxfordian) of Liaoning Province, China. *Journal of Vertebrate Paleontology* **39**, e1588285 (2019).

Johnston, P. Cranial muscles of the anurans *Leiopelma hochstetteri* and *Ascaphus truei* and the homologies of the mandibular adductors in Lissamphibia and other gnathostomes. *Journal of morphology* **272**, 1492-1512 (2011).

Jupp, R. & Warren, A. The mandibles of the Triassic temnospondyl amphibians. *Alcheringa* **10**, 99-124 (1986).

Kamei, R. G. *et al.* Discovery of a new family of amphibians from northeast India with ancient links to Africa. *Proceedings of the Royal Society B: Biological Sciences* **279**, 2396-2401 (2012).

Kent, D. V. & Irving, E. Influence of inclination error in sedimentary rocks on the Triassic and Jurassic apparent pole wander path for North America and implications for Cordilleran tectonics. *Journal of Geophysical Research: Solid Earth* **115** (2010).
[truncated: 132,827 more chars]
