# Supplementary material for: Triassic stem caecilian supports dissorophoid origin of living amphibians
Source: Nature. 2023 Jan 25;614(7946):102–7. doi: 10.1038/s41586-022-05646-5 (PMC9892002; doi:10.1038/s41586-022-05646-5)
Supplement: Supplementary file 2 — Reporting Summary [file 41586_2022_5646_MOESM2_ESM.pdf]

nature portfolio

Corresponding author(s): Ben T. Kligman

Last updated by author(s): Sep 27, 2022

Reporting Summary

Nature Portfolio wishes to improve the reproducibility of the work that we publish. This form provides structure for consistency and transparency in reporting. For further information on Nature Portfolio policies, see our Editorial Policies and the Editorial Policy Checklist.

Statistics

For all statistical analyses, confirm that the following items are present in the figure legend, table legend, main text, or Methods section.

n/a	Confirmed	
☒	☐	The exact sample size (*n*) for each experimental group/condition, given as a discrete number and unit of measurement
☒	☐	A statement on whether measurements were taken from distinct samples or whether the same sample was measured repeatedly
☒	☐	The statistical test(s) used AND whether they are one- or two-sided<br>*Only common tests should be described solely by name; describe more complex techniques in the Methods section.*
☒	☐	A description of all covariates tested
☒	☐	A description of any assumptions or corrections, such as tests of normality and adjustment for multiple comparisons
☒	☐	A full description of the statistical parameters including central tendency (e.g. means) or other basic estimates (e.g. regression coefficient) AND variation (e.g. standard deviation) or associated estimates of uncertainty (e.g. confidence intervals)
☒	☐	For null hypothesis testing, the test statistic (e.g. *F*, *t*, *r*) with confidence intervals, effect sizes, degrees of freedom and *P* value noted<br>*Give P values as exact values whenever suitable.*
☐	☒	For Bayesian analysis, information on the choice of priors and Markov chain Monte Carlo settings
☒	☐	For hierarchical and complex designs, identification of the appropriate level for tests and full reporting of outcomes
☒	☐	Estimates of effect sizes (e.g. Cohen's *d*, Pearson's *r*), indicating how they were calculated

Our web collection on statistics for biologists contains articles on many of the points above.

Software and code

Policy information about availability of computer code

Data collection	Reconstruction of CT scan data slice reconstruction was conducted in NRecon Reconstruction Software V 1.6.9.4 (Micro Photonics Inc.). CT scan data segmentation and creation of 3D meshes was conducted using Dragonfly (v.2020.2). Images of 3D meshes were produced using Meshlab 2021.07 (https://www.meshlab.net/). Composite skull reconstructoin produced using Meshmixer 3.5 (https://www.meshmixer.com). Image stacking was conducted in Adobe Photoshop CC 23.0.0 (https://www.adobe.com/products/photoshop.html). Figures were produced using Adobe Illustrator CC V. 26.5 (https://www.adobe.com/products/illustrator.html).
Data analysis	The data was compiled in Mesquite (v.3.04); our analyses were conducted in the phylogenetic sofware TNT (v.1.5) and MrBayes (v.3.2.6). All code used in the phylogenetic analyses herein are available in Supplementary Information 8 (Phylogenetic Datasets) and are also available for download under project 4166 on Morphobank.org (http://morphobank.org/permalink/?P4166).

For manuscripts utilizing custom algorithms or software that are central to the research but not yet described in published literature, software must be made available to editors and reviewers. We strongly encourage code deposition in a community repository (e.g. GitHub). See the Nature Portfolio guidelines for submitting code & software for further information.

Data

Policy information about availability of data

All manuscripts must include a data availability statement. This statement should provide the following information, where applicable:

- Accession codes, unique identifiers, or web links for publicly available datasets
- A description of any restrictions on data availability
- For clinical datasets or third party data, please ensure that the statement adheres to our policy

The holotype, paratype, and referred specimens of Funcusvermis gilmorei are cataloged and available for study to qualified researchers upon request at Petrified

Forest National Park. Computed tomography scan data, including surface volume files (3D meshes) and raw CT data of specimens mentioned in the main text and extended data figures (including the holotype, paratypes, & referred specimens), as well as a surface volume file of the composite skull reconstruction are available for download under project 000382289 on Morphosource.org (https://www.morphosource.org/projects/000382289?locale=en). Full phylogenetic datasets are available for download under project 4166 on on Morphobank.org (http://morphobank.org/permalink/?P4166).

Field-specific reporting

Please select the one below that is the best fit for your research. If you are not sure, read the appropriate sections before making your selection.

☐ Life sciences ☐ Behavioural & social sciences ☒ Ecological, evolutionary & environmental sciences

For a reference copy of the document with all sections, see nature.com/documents/nr-reporting-summary-flat.pdf

Ecological, evolutionary & environmental sciences study design

All studies must disclose on these points even when the disclosure is negative.

Study description	Description of new species of stem caecilian based on data and images derived from photographs, histology, and CT scans of fossil specimens collected from the Late Triassic strata of Arizona, U.S.A. A new phylogenetic hypothesis of lissamphibian evolution revealing new information regarding the monophyletic nature of living amphibians; the origins of the caecilian musculoskeletal apparatus and the lissamphibian mandibular ramus and their transition from the condition of dissorophoid ancestors; the evolutionary timing of lissamphibian origins; the biogeographic and ecological history of living amphibians.
Research sample	The new species is represented by a single holotype specimen, 60 paratype specimens, and 10 referred specimens. The phylogenetic dataset includes 357 discrete morphological characters obtained from 63 extinct and living terminal taxa including stem and crown amniotes, stereospondyl and dissorophoid temnospondyl amphibians, batrachians, gymnophionomorphs, and albanerpetontids. Phylogenetic data builds on that of previous studies (refs. 6, & 14), and new data added is described in detail in the Supplementary Information.
Sampling strategy	No statistical methods were used to determine sample size. Sample size was limited to the specimens collected in this study.
Data collection	Detailed description of field and lab methods for collection of specimens in Methods. Digital photographs acquired using a Leica MZ67 stereomicroscope and a Sony NEX-5T digital camera, and a Sony NEX-5T digital camera mounted on a Nikon OPTIPHOT-POL Polarizing microscope. Micro-computed tomographic scans acquired using a Skyscan 1172 Microfocus X-radiographic Scanner, and a Nikon XTH 225 ST High-Resolution X-ray Computed Tomography Scanner. New phylogenetic data derived from peer-reviewed published literature, and personal observations.
Timing and spatial scale	Specimens were collected between 2018 and 2021. Information on the location of specimens described herein detailed in the Main Text, Methods and Extended Data Fig. 1.
Data exclusions	No data were excluded.
Reproducibility	Code for reproduction of our phylogenetic results presented herein are publicly available in the Supplementary Information and in an online repository (see Data Availability and Code Availability statements).
Randomization	Specimens were determined to belong to the same, new species due to the presence of a unique combination of characters unknown in any other extinct or living taxon. Randomization was not relevant for phylogenetic data sampling conducted herein.
Blinding	Blinding was not relevant to the morphological study of palaeontological specimens or the analyses conducted herein.

Did the study involve field work? ☒ Yes ☐ No

Field work, collection and transport

Field conditions	The fossil specimens were collected from a single outcrop (PFV 456) in the badland landscape of Petrified Forest National Park, Arizona, U.S.A. Annual average precipitation for this location is 24.1 cm. Its climate is classified as cold semi-arid under the Köppen climate classification system.
Location	The fossiliferous horizon (PFV 456) is located at approximately 1,630 meters above sea level, in Petrified Forest National Park, Arizona, U.S.A. (N34° 59’, W109° 42’). Detailed locality information available to qualified researchers upon request at Petrified Forest National Park.
Access & import/export	All fossil specimens used in this study were collected from Petrified Forest National Park as part of the United States National Park Service project PEFO-00030. 2018-2020 specimens permit: PEFO-2017-SCI-0003, issued on March 7, 2017. 2021-2022 specimens permit: PEFO-2021-SCI-0003, issued on January 1, 2021.
Disturbance	The study did not produce any environmental disturbance.

Reporting for specific materials, systems and methods

We require information from authors about some types of materials, experimental systems and methods used in many studies. Here, indicate whether each material, system or method listed is relevant to your study. If you are not sure if a list item applies to your research, read the appropriate section before selecting a response.

Materials & experimental systems

n/a	Involved in the study
☒	☐ Antibodies
☒	☐ Eukaryotic cell lines
☐	☒ Palaeontology and archaeology
☒	☐ Animals and other organisms
☒	☐ Human research participants
☒	☐ Clinical data
☒	☐ Dual use research of concern

Methods

n/a	Involved in the study
☒	☐ ChIP-seq
☒	☐ Flow cytometry
☒	☐ MRI-based neuroimaging

Palaeontology and Archaeology

Specimen provenance	All Palaeontological specimens used in this study were collected from Petrified Forest National Park (PEFO) as part of the United States National Park Service project PEFO-00030. 2018-2020 specimens permit: PEFO-2017-SCI-0003, issued on March 7, 2017. 2021-2022 specimens permit: PEFO-2021-SCI-0003, issued on January 1, 2021.
Specimen deposition	The holotype, paratype, and referred specimens of Funcusvermis gilmorei are catalogued and available for study to qualified researchers at Petrified Forest National Park (PEFO).
Dating methods	No new dates were provided.

☒ Tick this box to confirm that the raw and calibrated dates are available in the paper or in Supplementary Information.

Ethics oversight	Petrified Forest National Park, Virginia Tech Department of Geosciences

Note that full information on the approval of the study protocol must also be provided in the manuscript.
